# Supplementary material for: In situ functional dissection of RNA cis-regulatory elements by multiplex CRISPR-Cas9 genome engineering
Source: Nat Commun. 2017 Dec 13;8:2109. doi: 10.1038/s41467-017-00686-2 (PMC5727397; doi:10.1038/s41467-017-00686-2)
Supplement: Supplementary file 10 — Supplementary Information [file 41467_2017_686_MOESM10_ESM.pdf]

Description of Supplementary Files

File Name: Supplementary Information
Description: Supplementary Figures and Supplementary Notes

File Name: Supplementary Data 1
Description: **GenERA CRISPR/Cas9 sgRNA libraries corresponding to data in Fig. 1-5**. Detailed representation all sgRNA spacer sequences together with corresponding protospacer genomic coordinates, forward/reverse oligonucleotides used for cloning in pAc-Cas9-sgRNA vector, and PAM identity/position.

File Name: Supplementary Data 2
Description: **NGS library primers and amplification conditions used for generation of GenERA amplicons**. Targeted PCR conditions including annealing temperature, minimal non-saturating cycle number and amplicon-specific primer sequences are displayed for each amplicon in the study

File Name: Supplementary Data 3
Description: **Top expressed S2 miRNAs**. Seed sequence identity of most abundant S2 miRNA families is shown together with their ranked expression levels as reported by Ruby et al.

File Name: Supplementary Data 4
Description**: Assembled miR-184 target network**. TargetScanFly 6.2 prediction of conserved 8mer and 7mer-m8 sites and miRanda prediction of conserved 8mer and 7mer-m8 sites with good miRSVR scores were included. * = putative lincRNA.484 MRE was excluded from further analysis due to genome misannotation. miRNA-MRE base pairing interaction are shown for miR-184 and most abundant S2 miRNAs.

File Name: Supplementary Data 5
Description: **Expression levels of putative miR-184 targets in S2R+ cells**. FPKM data from two previously published studies (Berkely Drosophila Genome Project (SRR070279) http://sra.dnanexus.com/runs/SRR070279/experiments; Berkely Drosophila Genome Project (SRR124149) http://sra.dnanexus.com/runs/SRR124149/studies) and an independent profiling performed in this study were used to compile an average FPKM value for each miR-184 target gene (genes with FPKM < 0.015 were not included in the analysis).

File Name: Supplementary Data 6
Description**: Putative RNA regulatory elements (RRE) and their occurrence within selected miR-184 targets**. The count, sequence and position of each RRE encoded within a 200 bp window centred on the miR-184 MRE seed sequence is shown.

File Name: Supplementary Data 7
Description: **GenERA CRISPR/Cas9 sgRNA libraries used for miR-184 MRE network analysis**. Detailed representation all sgRNA spacer sequences together with corresponding protospacer genomic coordinates, forward/reverse oligonucleotides used for cloning in pAc-Cas9-sgRNA vector, and PAM identity/position.

File Name: Supplementary Data 8
Description: Synthetic reference genome sequences underlying the miR-184 MRE network.

File Name: Supplementary Data 9
Description: **MRE-score distribution across miR-184 targets**. Weighted normalized MREscore (MRE-score), corresponding standard deviations, UDP counts and MRE-score group partitions are shown. miR-184 MREs reported by (Kertesz et al.,)31 and (Hong et al.,)32 were used for validation purposes.

File Name: Peer Review File

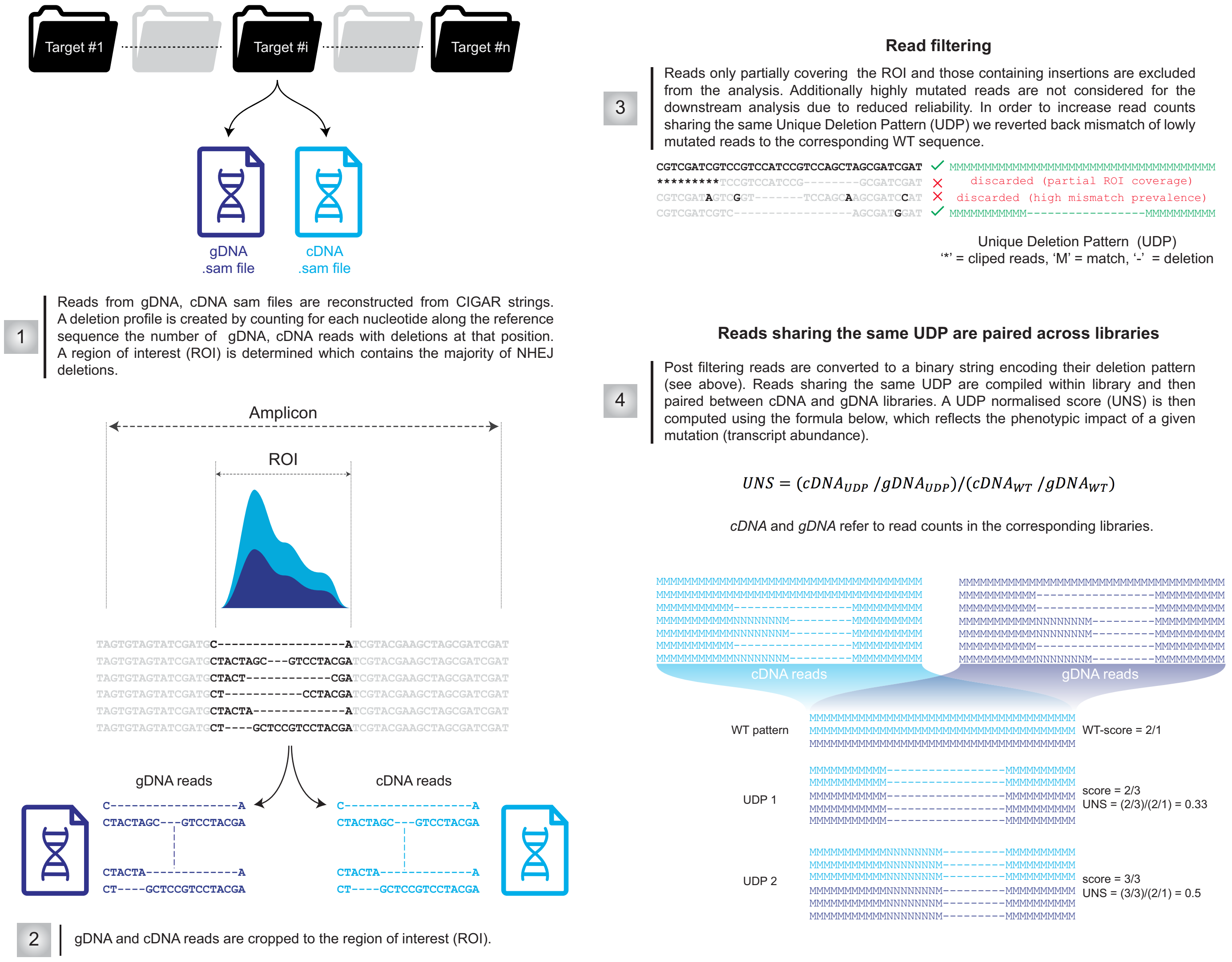

Supplementary Figure 1. GenERA computational pipeline . Stepwise overview of the computational pipeline used to derive the UDP normalised scores (UNS) from gDNA and cDNA library sam files containing mapped sequencing reads.

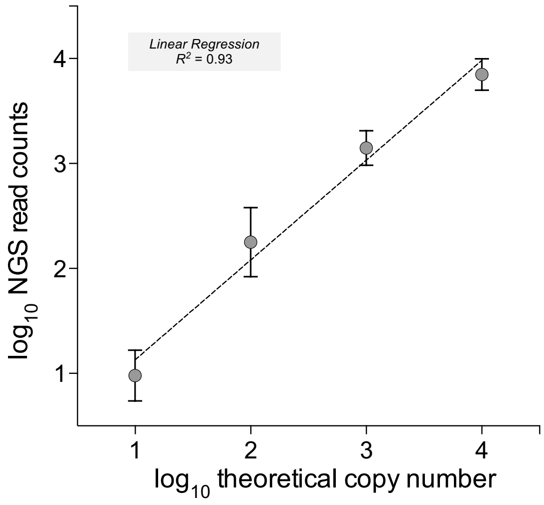

Supplementary Figure 2. Reliability of deep sequencing datasets. Estimation of NGS copy number resolution and dynamic range. Each dot represents average barcoded RP49 read counts following serial dilutions in gDNA and cDNA libraries. For each dilution, 3 barcoded RP49 triplicates were generated and sequenced. (Error bars = SEM).

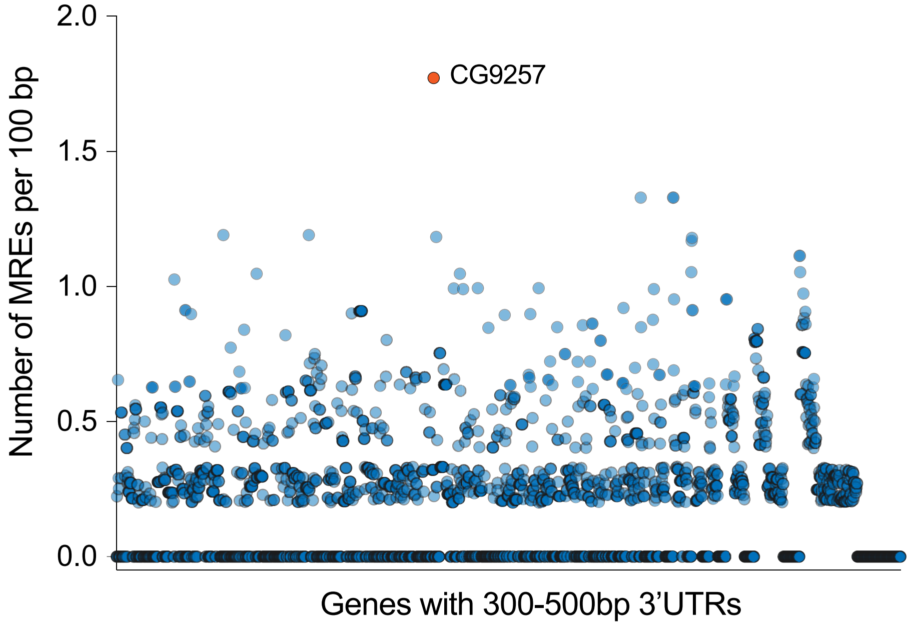

Supplementary Figure 3. Selection of a candidate 3'UTR for GenERA-based unbiased survey of regulatory activity. An MRE enrichment score, defined as the number of MREs per 100bp, was calculated for all S2R+ expressed genes containing 3'UTRs between 300 – 500 bp. The MRE predictions included the top 10 most abundantly expressed miRNAs in S2R+ cells (see Supplementary Data 3). The 3'UTR encoded in CG9257 (orange dot) displayed the highest MRE enrichment score among the 2311 genes subjected to this analysis.

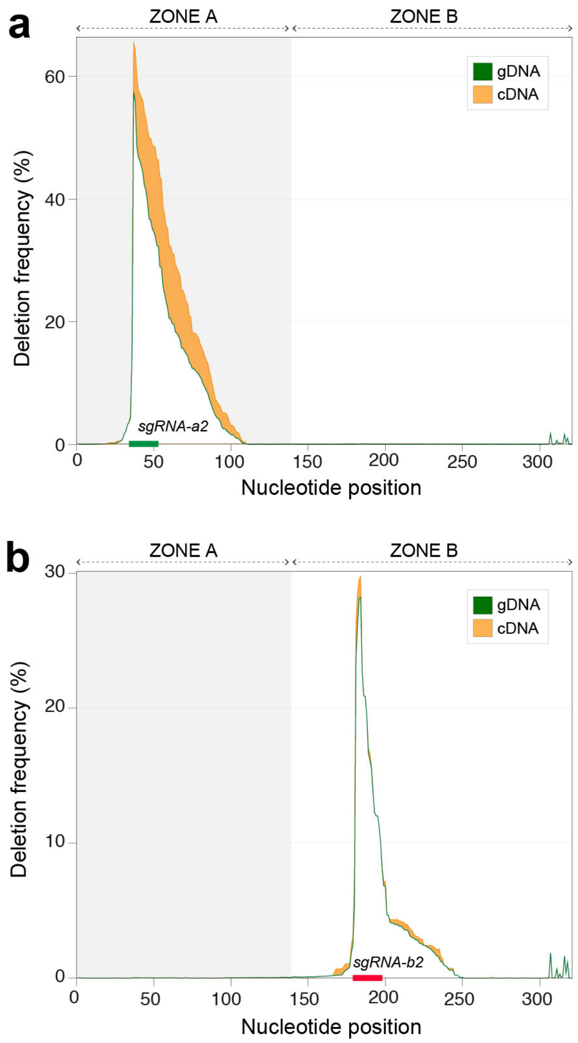

Supplementary Figure 4. Validation of differential regulatory potential associated with CG9257 3'UTR zone A and zone B . (a, b) Second set of individual sgRNAs separately targeting zone A **(a)** and zone B **(b)** of CG9257 3'UTR (relevant to Fig. 3 d, e). The cDNA and gDNA deletion frequencies across the 3'UTR are shown in orange and green, respectively. The precise positions of the sgRNA protospacers targeting zone A (*sgRNA-a2,* green) and zone B (*sgRNA-b2,* red) are displayed on the x axis.

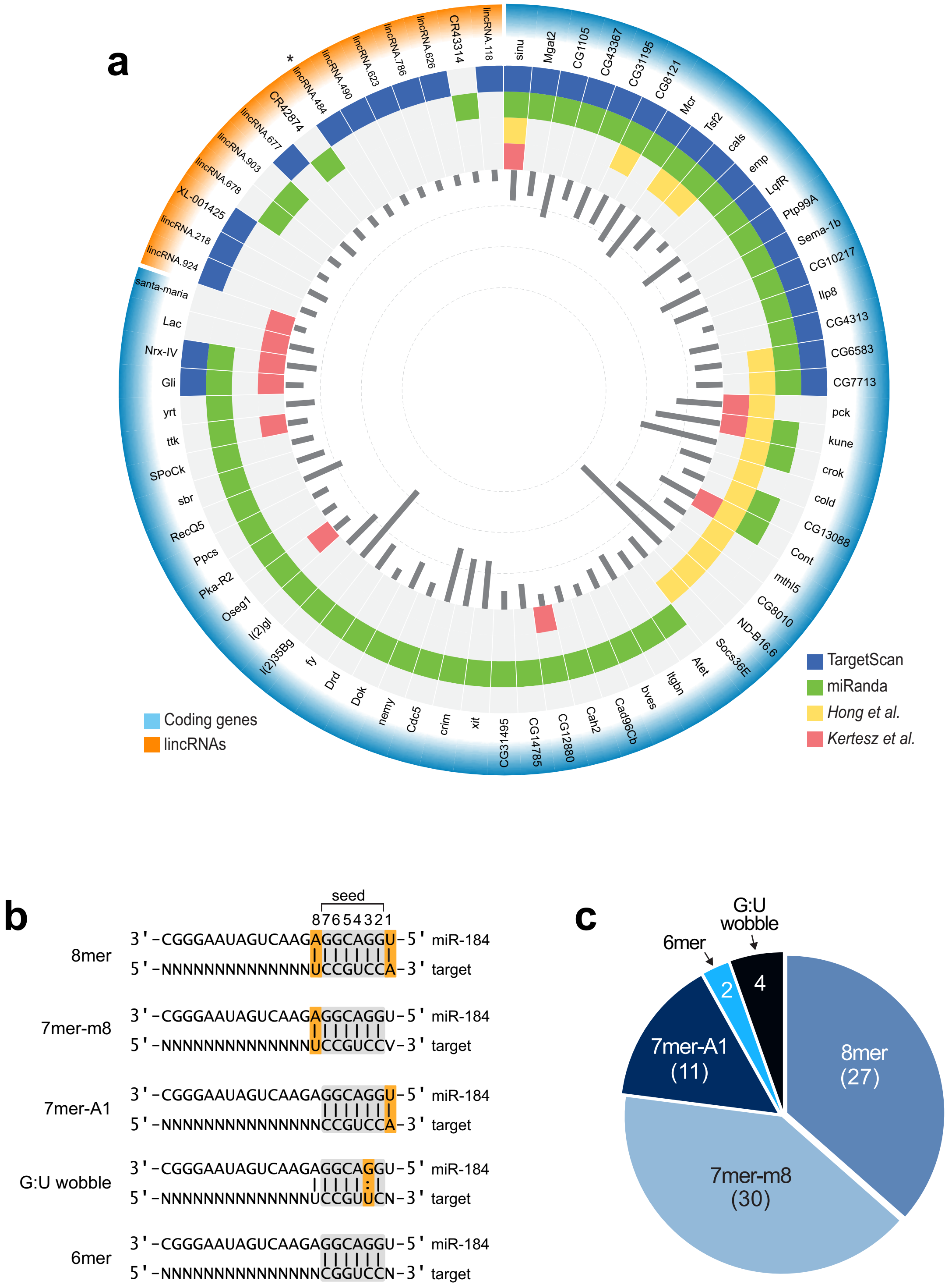

Supplementary Figure 5. Predicted miR-184 target network. (a) Graphic representation of the putative canonical miR-184 MRE network outlining the identity of coding and non-coding targets, their source of prediction, and relative expression levels (histogram) in S2R+ cells (* = putative lincRNA.484 MRE was excluded from further analysis due to genome mis-annotation). **(b)** Classes of representative canonical seed types found in the predicted miR-184 MRE network. Unique base pairing features defining each class are highlighted in yellow. **(c)** Corresponding incidence of single MREs within the miR-184 network.

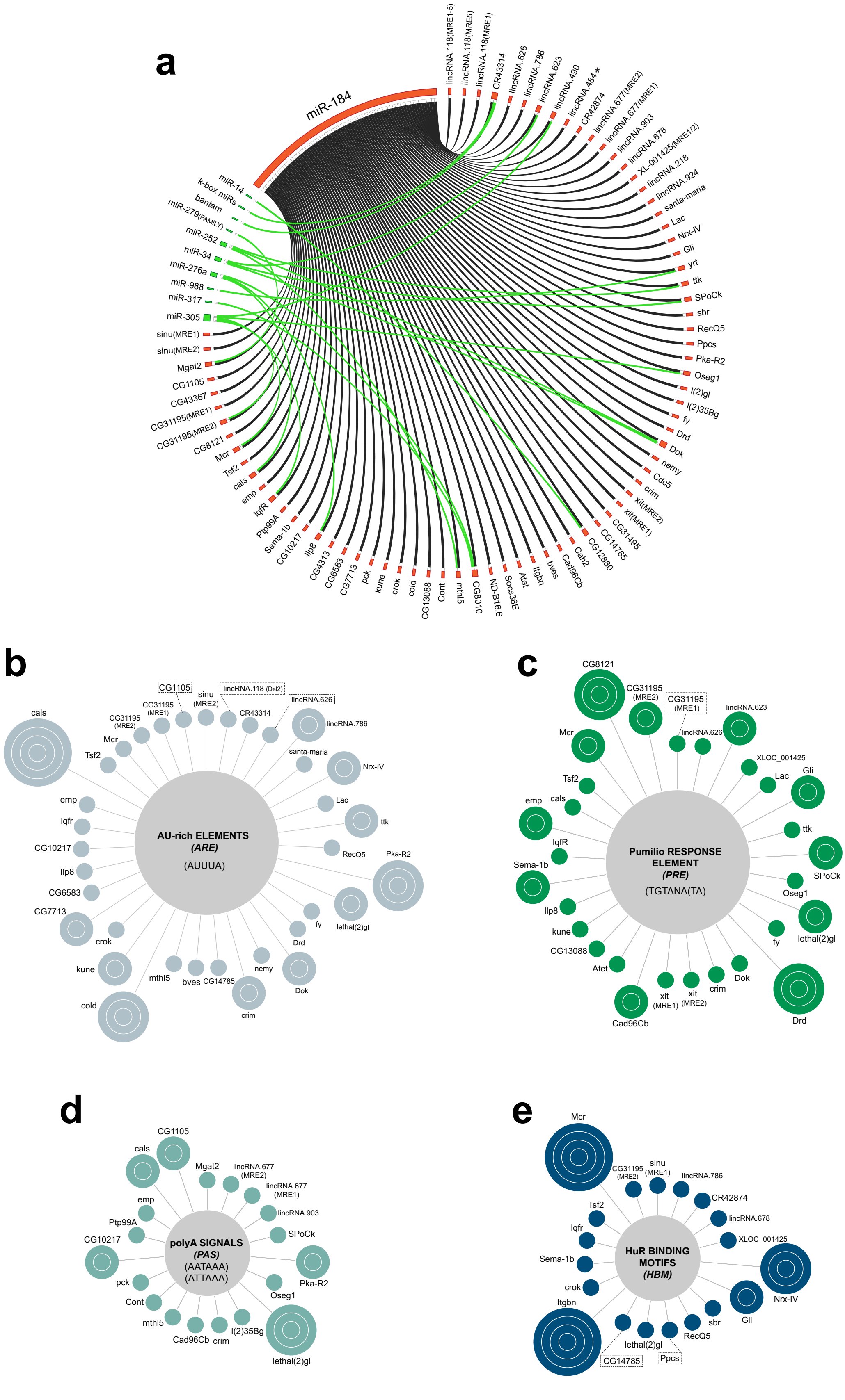

Supplementary Figure 6. Coincidental RNA regulatory elements across the predicted miR-184 MRE network. (a) Prevalence of predicted interactions between most abundant S2 miRNAs (green lines; *TargetScanFly 6.2*) and putative miR-184 targets (black lines). **(b - e)** *In silico* identification of AU-rich elements **(b)**, Pumillio response elements **(c)**, polyA signals **(d)**, and HuR binding motifs **(e)** encoded within a 200 bp window centred on the miR-184 MRE seed sequence. Circles reflect the number of predicted RNA regulatory elements (RRE) in each target.

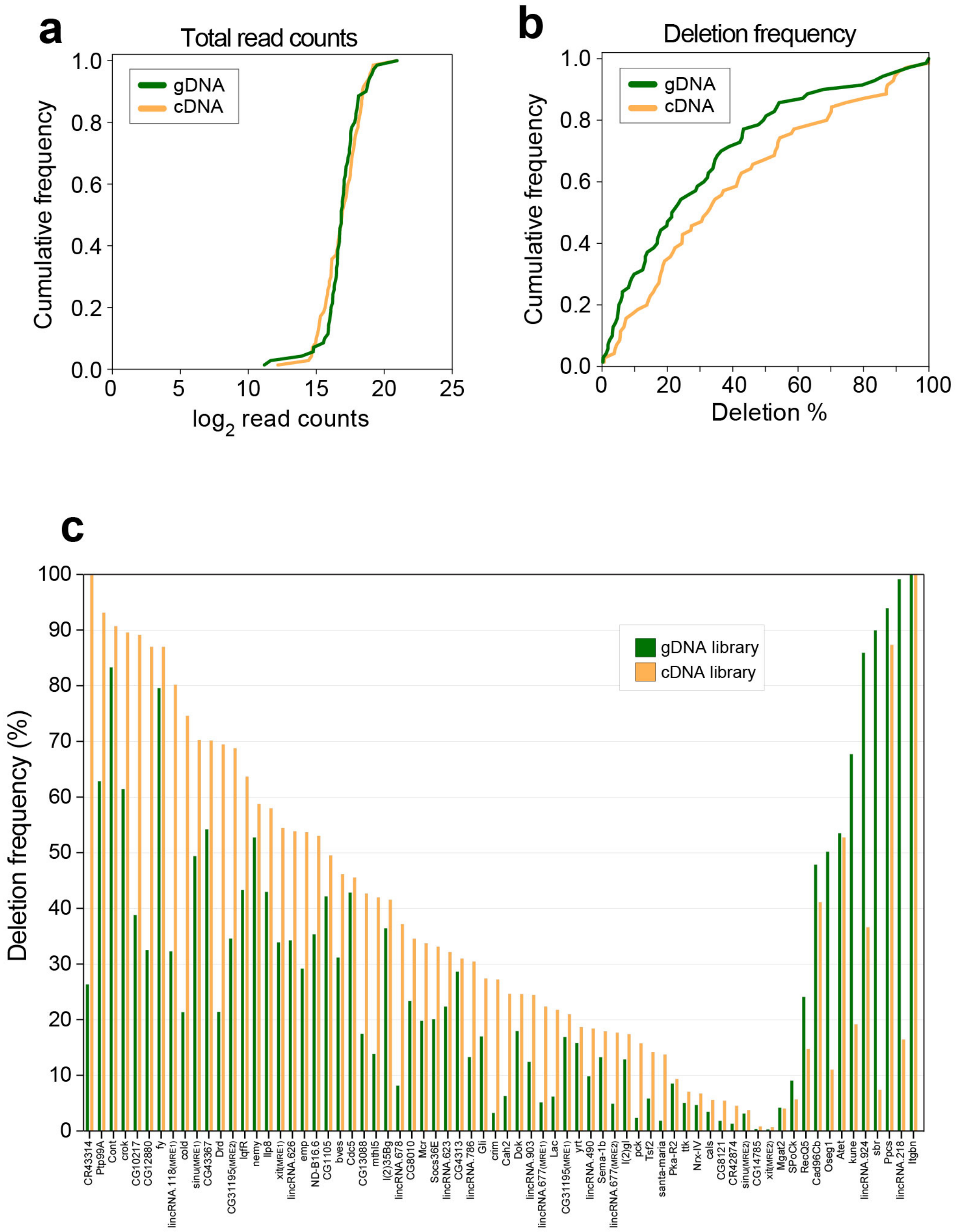

Supplementary Figure 7. Effects of CRISPR-induced genomic deletions on transcript levels. (a) Cumulative read count distribution across all amplicons in pooled gDNA and cDNA NGS libraries. Most gDNA and cDNA amplicons were sequenced at high depth, ranging between 16,384 (~214) to 262,144 (~218) copies per amplicon. **(b)** Comparative analysis of deletion frequencies in cDNA and gDNA libraries at the level of the entire network (Wilcoxon rank sum test, P = 0.037). **(c)** Comparative analysis of deletion frequencies in cDNA and gDNA libraries for every individual target underlying the predicted miR-184 MRE network.

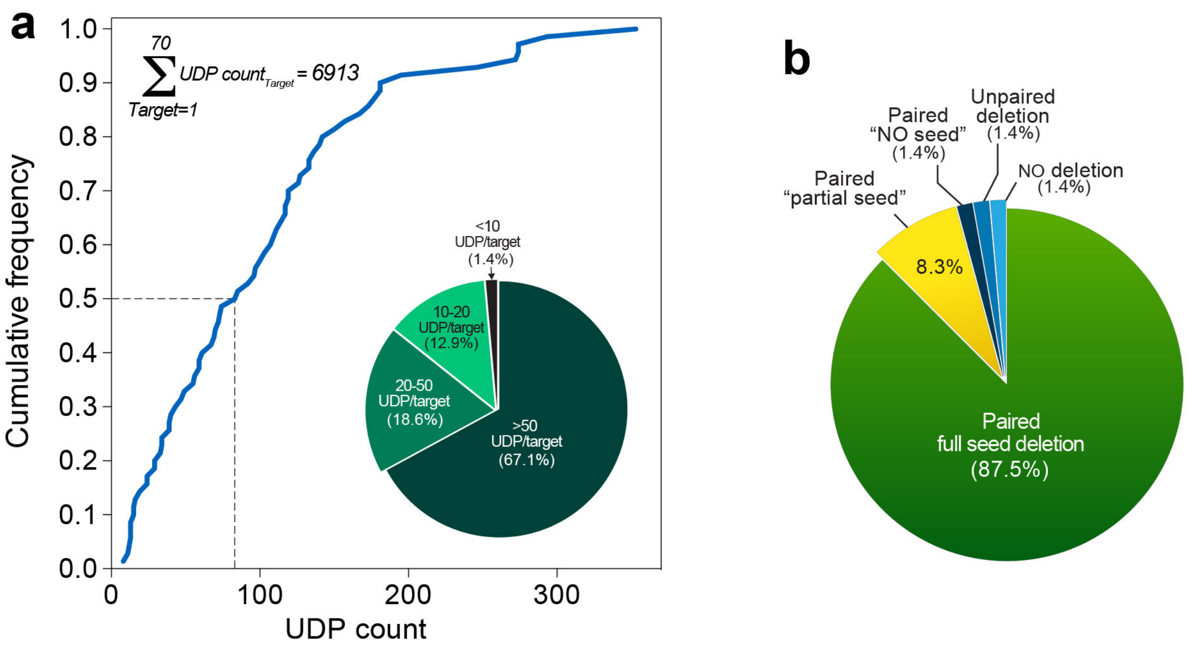

Supplementary Figure 8. UDP repertoire complexity. (a) Cumulative frequency of UDP counts across the miR-184 target network. A partition of the data into UDP count intervals is shown in the inset pie chart. **(b)** Classification of deletion patterns relative to seed coverage. Paired full seed deletions = amplicons carrying the same full seed deletion type in both gDNA and cDNA libraries; Paired "partial seed" = amplicons carrying the same partial seed deletion type in both gDNA and cDNA libraries; Paired "NO seed" = amplicons carrying the same deletion type outside the seed in both gDNA and cDNA libraries; Unparied deletion = deletion types found only in gDNA or cDNA amplicons; NO deletion = ineffective sgRNA.

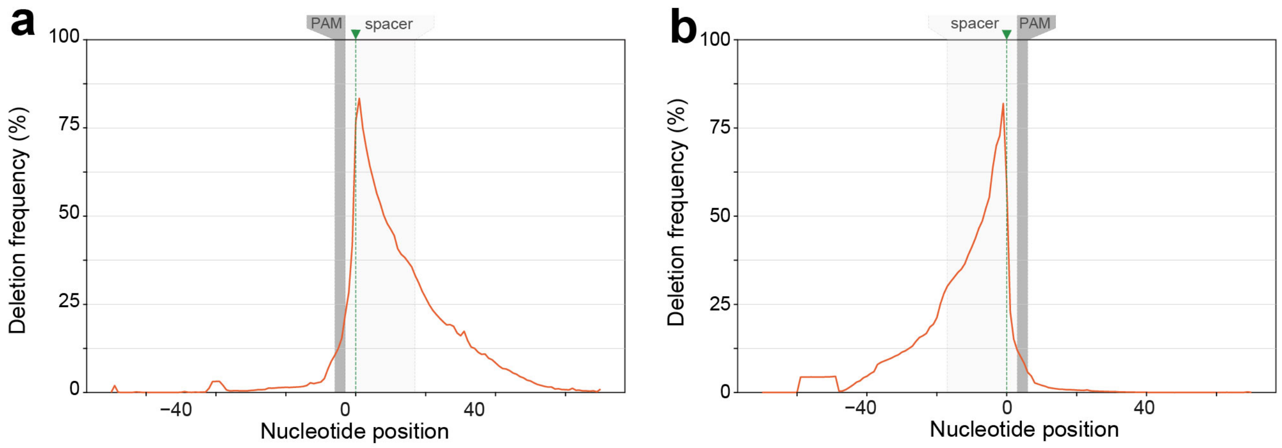

Supplementary Figure 9. Summary of gDNA deletion frequencies across the sgRNA library used for GenERA-based MRE network analysis. (a, b) All deletion coordinates were centered by the predicted Cas9 cut site (green line). A biased asymmetrical deletion pattern (away from the PAM) was consistently observed independent of the protospacer being oriented on the sense **(a)** or anti-sense **(b)** strand relative to the direction of transcription. A total of 55 **(a)** and 22 **(b)** genomic loci were considered accounting for 5357 and 1538 unique deletion patterns respectively.

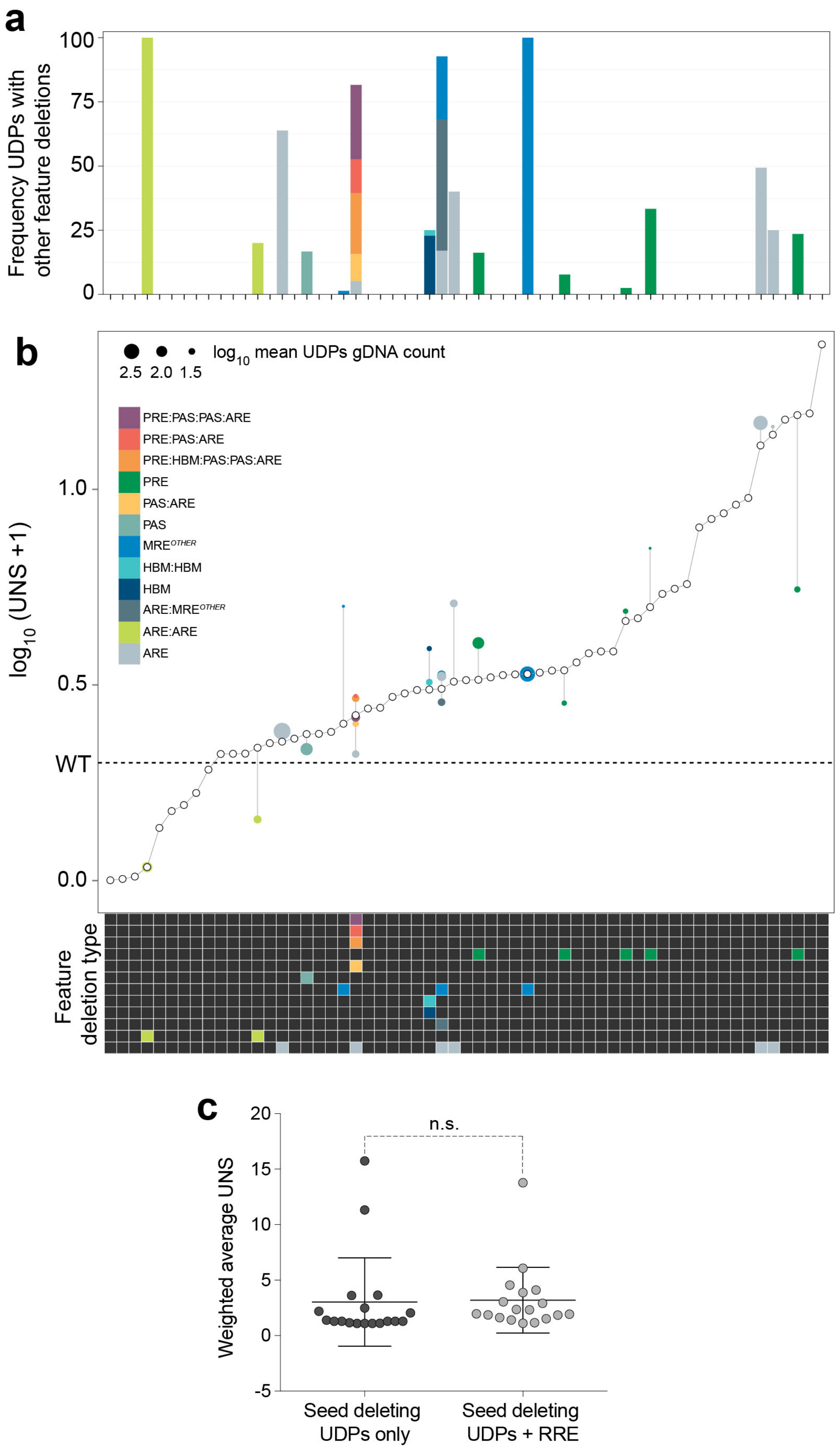

Supplementary Figure 10. Evaluation of coincidental RNA regulatory elements (RRE) deletions on MRE-score calculation. (a) Identity and frequency of all coincidental RRE deletion occurrence in UDPs considered for MRE-score calculations. **(b)** Comparative analysis of weighted average UNS values for seed deleting UDPs sharing RRE alteration events (colored dots) relative to corresponding MRE-score values (white dots). **(c)** Analysis of UNS values from seed deleting UDPs with or without coincidental RREs (paired *t*-test, P = 0.82). ARE = AU-rich elements; PAS = polyA signals; PRE = Pumilio response elements; HBM = HuR binding motifs.

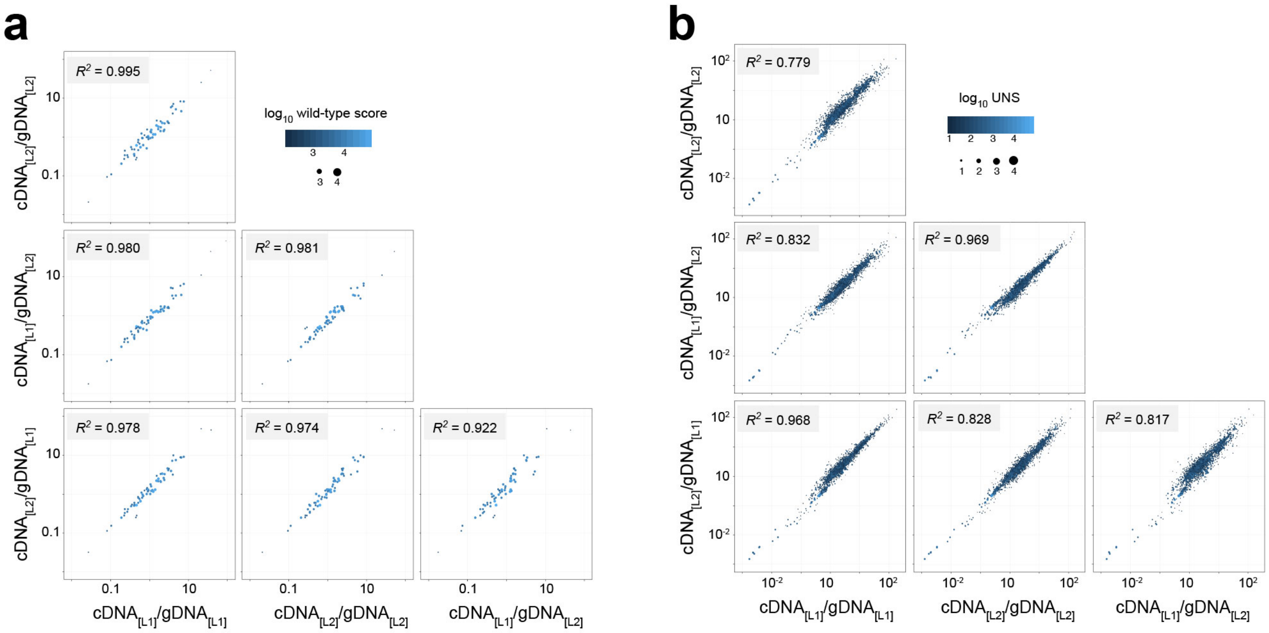

Supplementary Figure 11. Reliability of MRE-score analysis. (a-b) *Pearson's* correlation analysis of wild type scores **(a)** and UNS **(b)** between all gDNA and cDNA library replicate permutations.

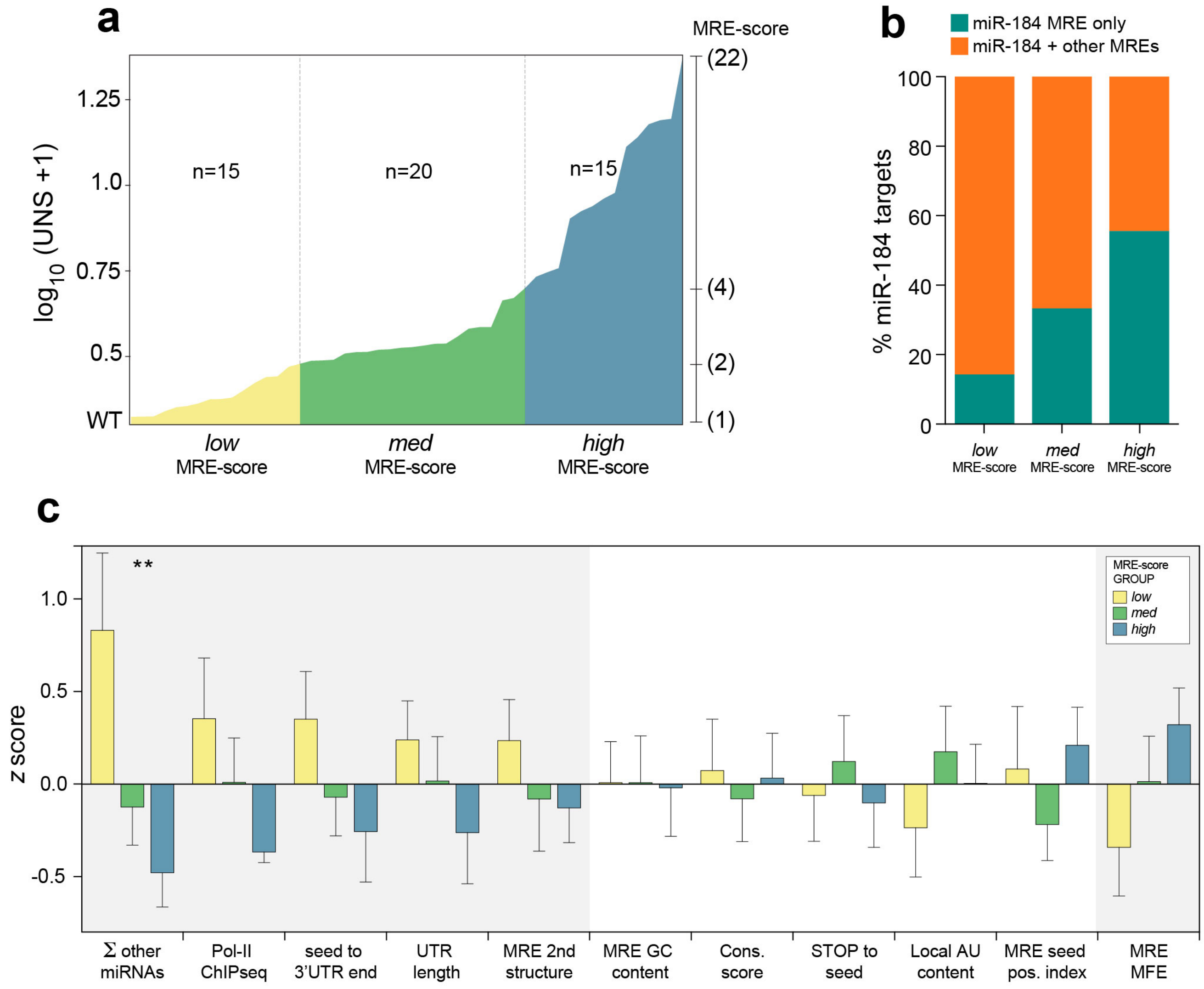

Supplementary Figure 12. Correlation of MRE-scores with contextual features. (a) Partition of miR-184 targets into MRE-score groups: *low* (1 < MRE-score < 2), *medium* (2 - 4), and *high* (4 < MRE-score). **(b)** Percentage of miR-184 MREs co-targeted by top 10 endogenous miRNAs across three MRE-score groups. **(c)** Comparison of average z-score values for 11 contextual features across MRE-score groups. Features with positive or negative trend across the three MRE-score groups are highlighted by gray shaded areas (n = 15 [*low*], 20 [*medium*], 15 [*high*], Error bar = SEM, ** *P* = 0.0065, one way ANOVA). Cons. score = conservation score; MRE MFE = MRE minimum free energy.

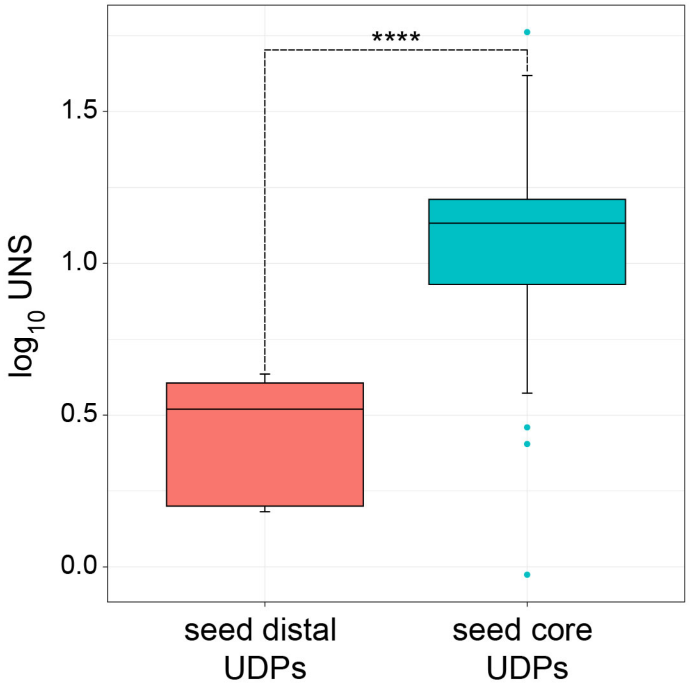

Supplementary Figure 13. Role of *crok* MRE distal seed nucleotides in miRNA -mediated repression. UNS of all seed deleting *crok* UDPs were partitioned into those deleting core seed nucleotides (nt. 2-7, teal) and those affecting only extended seed distal positions (nt. 1 or 8, red orange). Core seed deletions (teal) displayed significantly higher UNS values than those only affecting distal seed positions (red orange) (Bonferroni rectified ****P < 0.0001).

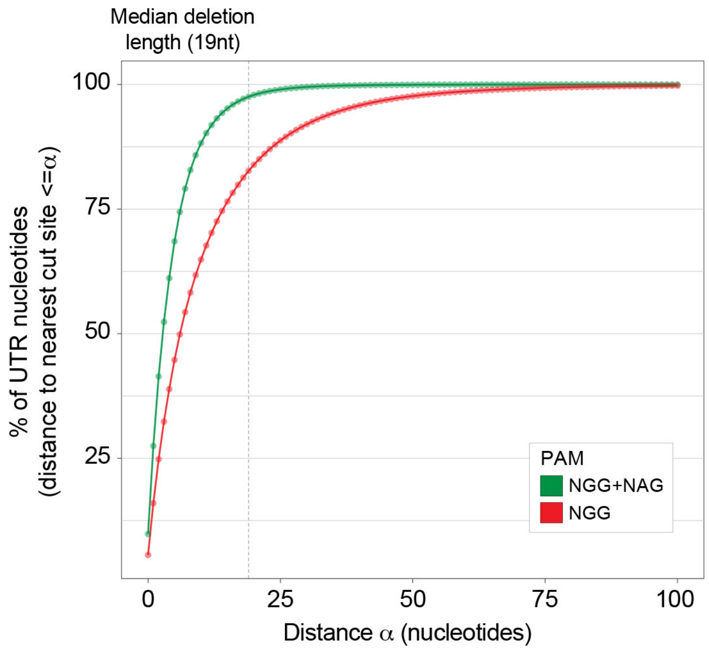

Supplementary Figure 14. Analysis of Cas9 cut sites frequency across *Drosophila* 3'-UTRs. Graph displays for a given distance α (x-axis, in nucleotide) the fraction of nucleotides in the *Drosophila* 3'UTR repertoire at a distance equal or shorter than α from the corresponding nearest CRISPR-Cas9 cut site (y-axis). CRISPR-Cas9 cut sites considered to derive this graph were either NGG PAMs only (red) or NGG + NAG PAMs together (green). Dashed line = median genomic deletion size in the GenERA screen (19nt).

Supplementary Note 1: CG9257 3'UTR saturation editing

*GCCTTTATACCACTGCTGT***TCTGCCTCTGCAAGCTGTGA**T**CCCCA**T**CCG**TTGCTCAC
TCAACG**CTT**TCG**CCT**GCGT**TGG**TTT**CCT**GCTTTGTGATTTTGTGATTTTGATAAAAG
A**CTT**AATTATATA**CTT**ATCGTTTAGTTCGTACGTTACGCACT**CCTTAGAG**TT**CGG**TT
CCCTTATT**CCCAGG**ATGCAAAGCTCTCTAACG**CCC**TGTACTTT**CCA**TATTTGATAAG
CAATT**CTT**TTGTGTT**CTT**CTCGTTGA**CCACTGCCG**T**CCCAGG**CTCGTGTA**CTT**AATC
GTT**GAG**TCA**CTT**GATTTCAATT**CCCGG**ATCGTTTGA**CCG**CTTGACT**AGG**ACAATGTG
CGCAAAAGGT**TAG**ATCGGTTTTT**GAG**A**CAG**TTTTTG**CTTGGG**AA**CTG**T**TGG**TTTTGA
ATGACATTGAATAAA***ATTAACCTTGTTTGTTATTG***aacaaaaagcttttgattattt
tcatgatgg*CCTGGATTGTGCGACGACT*

NN: ORF exon
NN: 3'UTR
NN: PAM sequences
NN: polyA signal
NN/*NN*: Fwd / Rev gDNA NGS library primers
NN/***NN***: Fwd / Rev cDNA NGS library primers

To maximize the recovery of NHEJ-induced deletion events, the gDNA 5' and 3' PCR primers for targeted NGS library generation were designed outside but immediately flanking the UTR sequence. For cDNA amplification, the position of the 5' primer overlapped that in gDNA while the 3' primer was placed immediately adjacent to the predicted polyA signal at the end of the transcript (Fig. 2b, Supplementary Data 2).

Supplementary Note 2: CG9257 multiplex MRE editing

GGGCCTTTATACCACTGCTG**TTCTGCCTCTGCAAGCTGTGA**TCCCCATCCGTTGCTC
ACTCAACGCTTTCGCCTGCGTTGGTTT**CCT**GCTTTGTGATTTTGTGATTTTGATAAA
AGA**CTT**AATTATATA**CTT**ATCGTTTAGTTCGTACGTTACGCACTCCTTAGAGTTCGG
TTCCCTTATTCCCAGGATGCAAAGCTCTCTAACGCCCTGTACTTTCCATATTTGATA
AGCAATT**CTT**TTGTGTTCTTCTCGTTGA**CCA**CTGCCGT**CCCAGG**CTCGTGTACTTAA
TCGTTGAGTCACTTGATTTCAATTCCCGGATCGTTTGACCGCTTGACTAGGACAATG
TGCGCAAAAGGTTAGATCGGTTTTTGAGACAGTTTTTGCTTGGGAACTGTTGGTTTT
GAATGACATTGAATAAAATTAACCTTGTTTGTTATTG

NN: ORF exon
NN: 3'UTR
NN: PAM sequences
NN: polyA signal
NN: predicted MRE seed seqeunces
NN: Fwd gDNA/cDNA NGS library primers
NN: Rev gDNA/cDNA NGS library primers

Supplementary Note 3: DNA sequences of all genes comprising the miR-184 target network.

NN: ORF exon
NN: 3'UTR
NN: miR-184 MRE
NN: Cas9 PAM
NN: sgRNA
T: G:U wobble

miR-184 MREs in coding RNAs

1. sinu

TATTACCAGTGCGGCCACACTGCAGTCCACACCCAAACAGCAAACCAAACAAACATTTCCCCAGGAAACCAGCGAAATTC
CAATAGAATTTCCCACTCAAAGG**ATGCAAAAACCATCCCACTTCGCACGCGAGGATTCCCAGGAGCACCTGCCCACCAGC**
CACACGCAAAACAGTCACACAGTGAACATGAAGAGACGCACACTATCCGGAAGTTGTGGCGTTGGCGTCTTCGTGTTCGC
CTTTGCCTTCATCGTGATTGCGTTTGCAACGCCCAGTTGGTTGGTCAGTGATTACCGCATCACGGGCGCCAAGCTGGATC
GCCTGGGATTGTGGGTGCACTGCTTCCGGTCGCTGCCGGACGTGAACGACGATAGCCAGCGGAGATTCTTCGTGGGCTGC
CGTTGGGTGTATGACCCATTTACCACGGGTTACGATGAGATCCGTGGATTCCTGCTGCCCGCCTTCATGATAGCCACTCA
GTTCTTCTACACTTTGGCCTTCATCGGGATGTTGGTATCTGCCATCGGAGTACTGGTCTTCATCCTGTGTGCTGGTCCGG
ATCAAAAGCACTTCATTACGCTCATTAAATCGTTGGGATATGTGCTTCTCGGTGCTGGAGTAAGTGCAGCCATTGCAGTG
ATTGTATTCGCTGGATTTGGCAACCGGAATGGCTGGATGCCGGAGCATGCGAACAATTGGTTTGGCTGGTCCTTTATCCT
GGCGTGTGTGGGTACTGTGTTAACCCTGGTGGCGTCTACTTTGTTCCTCAGTGAAGCCCACGTGCAGCACAAGAAGAGAA
TTCAGTTCAAGGAATCTCAGACGCGATTTGAGCTGGTGCGCGGATAGTCGATGCCGTGGCAGCTGGCACACTTGCACCAA
GGAAAACACAAGCCAACCAAGAGGAAAACAAAACAAAAACAT***TCCGTCCCCTCAAAGAATTCTCAC***CCTAAACATTACAG
TCGAACTTCTCTAAGGCGAAATATTCTAACTAACAGTTTCTGGTAGTGCACGTTTTTAAAGTTGGTTTATGTTCGTTTTA
TATTTTATTCAATGGTTTCCCTGTGCATAGGTTAAAGTATTCATGTCGGTAACATTCGTTTTATATTACCTTAAATCGTT
TAAATAACTCCCCAACTGGCGGAGATTTCGAAAAATTTTAACGAACCAAAGTTATTTTGGATAAAAATAATTTTCGCCAT
ATATTCATCGCTTCTCTCTAACATACCTAAAGAAATTAATTTTAGTTCAGAGAAGTTCGACTGTACTCGTAGTTTTTAAT
TTAAAAAGTTTATGCAATTGACGGCCGCACCGATTCACAAGCTTTCAGTTTTTGGTACTTATAATCATCCATCCAATCGA
CCAGATCAGTTCCAAGAGAAGACTGAATGTTTTTCCGAATGAAAACCAGAACCAAGCACAGCTAACATCAAACGAAGACG
CTCAACTAGATGACTCATGTTCATGTAGAGTATTCTACAGTATTTGCATATCAATTTTCGCTCAATTAACGCCCAGACAC
AAAGAGCATACCCCTCCGCAATTGTACGTTTATTTTTTTCTTTAGCAAAAGTCGCAGGCTAGTCGGAACTAGCATCGAAA
TATTACACTCGTGCCTTAATTAGATAGAACTATTATGGTGTAGTTCGTAGTGCCTGAAAACAAATTATTGTTATAAACAT
ACCTGAACGACTGACCTGCGACTAACCATAGAGATCATTGAATTGCATAAACTTCAGCTAAGCTAGTGCCTTAATTAAGT
GACCCAGAGAAAGGTAAGCGAAACTCCGCCCATTAGTTTTAAGAACAAAACATTCCCTTTTGAACTCGAATATTCCGCAC
AAGTTACCTTCTTTTAAAGGTAACAATTAGGT***CTGTATTAATCA***TCCGTCC***TTTG***CACGTATTTGTAACATTTTCTAAAC
CGAGAATTTAGCAATGTATTAAAGCAATACATACGTCTTTTACCGTCCCATATACAATAATATGTGTAACATTTTTATGT
ACAAACGACTTTTAGCTAAAGCGAAGAATAATAAAGTTTTTATTGTATACAACAGCTGAATGCAT

2. Mgat2

CAGATACTCAGTTACTCAGCTACTCAGATACTCGTTCGCAGCGGACGGCCGAGCTGAGATACTCGATCGCGAGCTTGTTG
TTGCCCCAGCCAGGCGAAAGTGAAAACGGAACGCCCAGATGAACGCCGAATAAATCAAGCGAAGTAGCAACAAATGCGGG
AAATGTGAAAATGCGCGCGGCAGTGGGTTGAAGCGAGACGGGTAGAGCTAGAGAGATGCCCGTTTGCCCGCTGTGCGTGT
GTGTGTGTGCAGCGGCGGCCGTAGCAGCTGCTGATGACGAGATGAAGGCGCTCACCCAGTGAAGAGCCAGCAGTTACTCG
AATCCGAGGAAGCGAGGAGCGACGAG**ATGTCGAAAATGAGGGGTAATTGTAATTGAATCGAGTTTCCGGCTTAGAAAAGC**
CAGCCTTGCTAAATCCATTAAATGTTTGCCATTGAATAGGTCGCATTCTGATCCCACTGCCCAATACCGGAGCGATGGGT
CGCAAGCGCAACAACTTCTACATGCGGAGTCTGTTCCTGCTGGCCCTGGGCATTTTCGGTCTGCTCCAGTACAACAACTT
CAATTATCGTAAGTAATAGGCGCCAGCAGCTCGCATCACCAGGTAAATCTTTACTTCTGTTGCAGTGGACTCGCGGACA
ATGTGCTGGGCGACGCGGTGACCAACGACTCGGACGACGCCATCCTGGCCATGGTGCCCGCCACCCTGCACAAGTATCTG
ACGCCCCACTCGCGCAATCACAGCGCCTCGGGAGCAGGAGCCCTCAACGGCGCGGGTAAGCCTTCAGCGAGTTAGCCAAG
ATAATGAGATAGTTTAACTGGATTCGAATCACCCTCTAGCCCTGCTCCTGAATGCCAGTAGTCCGGGCGCAGCTACTGCC
AGCACCATCAGCTTCGACGTGTACCATCCTCCGAATATCACCGAGATCAAGCGCCAGATCGTCAGGTACAACGACATGCA
GATGGTGCTCAACGAGGACGTCTTTGGACCGCTGCAGAACGACTCTGTGATAATCGTGGTCCAGGTGCACACGAGGATCA
CCTACCTGCGCCACCTGATCGTCAGCCTGGCGCAGGCCCGGGACATTTCGAAGGTGCTGCTGGTGTTCTCGCACGACTAC
TACGACGACGACATCAACGACCTGGTGCAGCAGATCGACTTCTGCAAGGTGATGCAGATCTTCTACCCGTACTCCATTCA
GACGCATCCTAACGAGTATCCCGGCGTGGATCCCAACGACTGTCCGCGAAACATCAAAAAGGAGCAGTGCGTATTGGCCA
TCGCTATATGTATGGCTATTTCTATAAGTATCCCTCGTTCCAGAGCTCTGATCACCAACTGCAACAATGCGATGTATCCC
GATCTCTATGGCCACTATCGGGAGGCGAAGTTCACGCAGACGAAGCACCATTGGATCTGGAAGGCCAACCGGGTGTTCAA
CGAACTGGAGGTGACCCGCTATCACACAGGACTGGTGCTGTTCCTCGAGGAGGACCACTACGTGGCCGAGGACTTCCTGT
ACCTGCTGGCTATGATGCAGCAACGCACCAAAGACTTGTGCCCCCAGTGCAACGTGCTGTCCCTGGGCACCTATCTCAAG
ACCTTCAACTACTACACGTACCACAGCAAGGTGGAGGTCATGCCCTGGGTCAGCAGCAAGCACAACATGGGCTTCGCCTT
CAATCGCACCACCTGGTCGAACATCCGGAAATGCGCCCGCCACTTCTGCACCTACGATGACTACAACTGGGACTGGTCCT
TGCAGCACGTTTCGCAGCAGTGTCTGCGGCGAAAGCTGCACGCCATGATTGTCAAGGGGCCGCGAGTCTTCCACATCGGA

GAATGCGGCGTTCATCACAAGAACAAGAACTGCGAGTCCAACCAGGTGATCTCGAAGGTGCAGCACGTGCTGCGCATAGC
CCGCAACTCGCACCAGCTCTTCCCGCGCTCCCTCACCCTCACGGTGCCCAGTCTGATGAAGAAGTCGAAGCTGCGCAAAG
GCAACGGCGGCTGGGGCGACATGCGGGATCACGAGCTGTGCCTCAACATGACGCTGGCCACGAGGTAAGGCCGGGGAGCC
CACGGAATGGAAACCAATCAGTCAGCAGCAAGGAAGCCTAGCTAATAGCGTTATCTAAGTAGGATTATTAAGCAGTAGTC
TTAGTCACTGTAAACGAACATTCAGCAAGTACTCTGCATTAGCTAACTAACCGAAAATAATCAAAGGCCAGAAGGCAGAA
GCCGGAAGCCAGAAGGCATGAGCCGGAAACCGGAAACCGGAAGCCAGAAGCCCAAAGACCCTCGCCTCCGGCGCTGCACA
ACTCATACTATAAGTAACCCTCTAGTCACACATTCAATTGGAATCGCCGTGTTGTTGCAACCTCCAATCTTGTGCCGTTC
GCCCTTCAGTTTGTCGATCATCACTGGAAGTGT***TTAGGCTCCGTCCAGATCCAAAG***ATCGACAGAGTTCCAAGCGACGGG
GCGGCAATCCTTCAAACTGAAACCAGAAACTAAACCTACGAATACTAGCGATAGACCATACACATACATACATATGTACT
AACTCGTATCCCCAGATTGTGCCGAAGCCGTTTCGCAAGCGGCCAATTAATTTGAGTCTAGATCTAGAGGCAGCAAATCG
CATAAACTAGTCATAGCAATGTATTACAGCACATGCGCTATAATACATCATAGATCAAACTTTTGATATATCTACGAGCC
TACGTTTTAAGCCTGTACATAGAGGAATTGCGTTGTATACATCACATAAGGTCTTTTTATGATATTAACGCGGAACTAAT
GTAATTTGTTATTTTTTAATCGTTAGTCCAAAGCGCTTTTATGTATTAGGAATTTTGATTAATCGAACGAATAATCCTAG
TGAATAAACGCCAAGTAAATGCTCTACCAGTATAGACTTTAAAAACC

3. CG1105

ATCTCTAATCTCTGAATTCGGCGACCGCAGGGCGTTTCAAAACAGACCGAAAAGTCGTTAACTTATTGGTGCCTTGAGTC
ACAGAATCTCTAAC**ATGGGCCTTAAAGGTTGTGAATTGCAATTGGACAACCCGTGGAACACCTACTACGCCGGTCAGACG**
GTCAACGGCCAGGTGAAGTTTACGTTCGACTCGCCCAAAAAAGTCCGAGGTATTATCATTCGGTTCCTGGGAGAAGCCAA
CACCGAGTGGTCAGAGGAGAAGAGCGTAACCACCAGCGAGGGCAAGACCGAGAACGAGGTCACCCAACTGAAGGGGCATG
AGGAGTACTTTAAGATCCAGTACTATCTGCTGGGCGGCAAAAACAGCTCTGAGACGGAACTACCGCCTGGCACTCACACT
TATCCATTCACCTGTGCTCTGCCGCCTAATCTACCCTCATCCTTTGAGGGCGAATTTGGCCATGTGCGCTATACCATCAA
GGTGACGCTTGATCGTCCGTGGAAATTCGATCAGGACATGAAGATGGCGTTCACAGTAATAGCGCCCGTTGATCTAAATC
TCAATCCTCGAGTCAAGGAGCCATTCAAGCTCGAGCTAGAAAAGTCATTCTGCTGTTTTTGCTGTCGCTCAGGACCGCTG
GCTGTCATCACGAACATCCCGCAAACAGGATTCGTTTCGGGGCAGGTGCTGCCCATCACCTGCGAGGTGGACAACACCAG
CAACGTAAATCTTACGGCAGTGAAGTTTGAACTGCGTAAGCTGGTCACCTTCCACACAAACCAGCCACGGAGCGAGAAGC
GCGAGTCCAAGGTGATCATAGCCAACTTAAGTGTTGGCCCAGTCAATGGCGGCGAGTCTCGCACGTTTACGCAACAAATG
GAGATTCCGGCGCTGCCACCGACCAACCTCCTCAACTGCGGGATTATCGCTTTGGACTACGACCTGCATGTGGAGTGCGA
GGTGAGCGGTCCGCACCGCAATCTCACTGGCAAGGTGCCAATCACCTTGGGCACTATTCCATTGGCTGGCGTACGGCCAC
CTACTCAATTTACGGATGCTCCGTCGGCGGTTCAGTCCGAGGATCCATCGCTGGCGCCCACACAGCCAGTGAGCCCGGCG
AGCCCTCCTGGCGGCGATGGCGTGGGTGGTGCTCTAGGCTGGAACGTGGCTGACAGTACTGGTGGTGGTTCACTGTATCC
TAATATACCGCCACCGCAGTTTGTGGAGACACAGTACCGGGCACCAACGATCGCCGGTAGGGACGACTCCGAGCATACGC
AGATGATCGGCGACGGAGCCTTCGCACCTCGGTATCCGACTTTCCAGTTTAATAATGCCACAGCACCGCCAGCTAGCCAG
TGATTGAATCTCTAAGCTAGACCCACAATGATATTCTTAAACTTTGATGTCGAACTATTTTCTTCTGGCTGTCTCTGTTT
CTGGAGGGTTAGCTTTCGCAATTTTTATCGGCTTTGCATTTATGAAATATTAGCTT***TCCGTCCAAATAGAAAGCATAAAC***
AGGTGCAGTCAAAATAAAAAAGGTCTACCTATACAAAGTTTTTGAAATGCTTAACTTCTGTTTATAATTAAATAGCTTTC
GGACAGTAAATACAAAGAATTGGGTCTTTATTATTTTGACTGCATCTGTGTTTAATTTAATAATCGTATAAATACGCACA
ATTGTCAATTCCGATTTAAACTGCTCAAATTTAATTTTTTCTGTTTTTTTTTAAGCCAGCAATAGTGCCTAAAACTGATG
TACGACTATAATCGTAATAAAACACAAAAGAT

4. CG43367

ATGCGTCTGAAATTGGAGCCTCAGTTATATCCAAATAAACACGAGAATGCCGCCAATCTGCGCGACAATGCGACCAGTGC
CTACGACACCAAGGAAATCTCTGAATTTGACTCCACCATCAAGAATGCCGTGGTGCGCGATTTCCTGGCGGATGACGAAA
GTTCCCAGCTGGAGGAGGAACTACGTCAACTGATCGACACGCAAGCGCAACAGGATGTGGCGCTGGAGAAGCTAGTCATG
TCACAGGACTGTGAGCTGATCACGCTGATGACCAAGGTGAAGGGACGCATTGAGGTTAACCAGAGCGTGTTCACCTTTGT
GGACTTGAGCCCGCCAACAGAGGATGGTTCCAAATACGATTTCAGGTTCTCCACCAACAAGATACGTGAGGTTCATCTGC
GAAAGTACAATCTGCGCAGGAGTGCTTTGGAAATCTTCCTGGTGGATCAGACTAGCTACTTCCTCAACTTTACGACGAAG
ACTCGAAATAAAGTTTTCACAAAAATCGTGGGTCTACCGCTTCCAAACATACTCTACGGCTCGGGAAGATCGCCGGCTGA
GCTGCTTAGGGCCTCGGGACTCACACAGAGATGGATCAATCGTGAGATTTCCAACTTTGAATACTTGATGTACCTAAACA
CAATCGCAGGTCGATCCTACAATGATCTAAGCCAATACCCCGTCTTCCCCTGGATCCTTGCTGATTACACCAGTGACGTG
CTGGATCTTACCGATCCAAAATCGTTCCGTGACCTATCCAAACCCATTGGATGCATAAATCCTAAAAACGAGGCCGAAGT
GCGCGGCAAATATGATTCATTCGAGGACCCCTCTGGTGCCATACCAAAGTTTCACTACGGAACCCACTACTCCAACTCGG
CCGGCGTGCTCCACTATTTGCTTCGCGTCGAACCGTTCACCTCGCTGCACATTGATCTGCAGAGCGGTCGTTTCGATGTG
GCCGATCGACAATTCCACTCGATACCTCAGACGTGGAAGTTGCTAATGGACAATCCCAATGACGTGAAGGAGCTGATCCC
CGAGTTCTTTTACTTCCCAGAATTCCTTAAGAACATGAATAAATTTGATTTGGGCCACTTACAAATAACCAAAGAAAAGG
TGGACGATGTCATATTGCCTGCTTGGGCTACCACGCCGGAGGAGTTCATAGCCATACACCGACGGGCTTTGGAATCGGAG
TATGTCAGTCAGCATCTGCACCACTGGATTGATCTGATCTTTGGGTATAAGCAAAAGGGACCCAAGGCAGCTGAGGCTCT
AAACGTTTTCTATTATTGCTCCTATGAAGGCGCTGTGGATTTGGACAAGATCACCAACCCTGTAGAACGGGAGGCGGTTG
AAGGAATGATTAATAACTTTGGCCAAGTGCCTTCACAGCTGCTGCGGGAGCCACATCCACGACGCCTTACTCAGGATGAG
ACGGCTCTAAAGCTGGTACGTGCTGAGCTCCGAAGGCCGGATCTTACTCAGTTCCTGGACAAGGTGGTGCAGTACTACTG
CGAGCTGTCCACGCCCAAGGATCCCATTGTGTACCTGAGTCCACCGCGAAGTCCTCCGCGATCGTTCCTGCAGCTGAGTC
CCGATGTTCTAGTCAGCATATCGAAGTCCACCATATTGGGCTGCAATTCGTGGCTATCGTTTGATAAGGATCAGGGATTC
CTGCTGGAAATCGATGCCACAACGGCTAATCTAAAGAACCGCAAGCGGATCTTTGGACCCTTCCACTCGTCACAGCAACC
GCATTCACAGCTCTTTGCGGTGAGCACGGATGGTAAGCTGCTCTATGCCGGTGGAATCTGGGATAACTCCTTAAGGGTGT

ACAATCTGAACAAGGGCAAGGCGGTGGCTTCGGTGACCCGCCACCTGGACATTATCACCTGCATAGCACTGGACAATTGC
GGCTCGTACCTGGTCACCGGCTCGCGTGATTGCACCTGTATTGTGTGGAGCATCCAAACAAATCAGCAGGGCGGTGGATC
CACTAACAACATTCCAGTTCATGCGCTGACGGGTCAGTCGCACCTACAGGCCATCACCCAGCTGAATACTCAGAATAGCT
ATTCACCCAAGCCCTTGACGGTGCTCTATGGTCACGACGATGCCATTTCCAGTGTAGCCATCTACACGGAACTGGATCTG
GTGGTCTCTGGATCCCTGGATGGAACTGTCAACGTGTACACACTTCAGGAGGGTCAATTTGTTAGAACACTGAAGCCCAT
TGGATGCACTGAGTCGTGTGTACAAATCTCTTTTGTAACCCTTTCCTATCATGGTCACATTGCATTCAGTGCTCTGGATG
ACACCTCGCACTCGGTGCACGTGTACAGCATCAACGGCTGCAGTCTGGGCTCCAAATACGTTTCTGGCAGGGTGACGGGA
TTGGCTAGTGCCTCCGACTACCTGGTGGTGGCCGACGATGCCGGCGATATCACCATGAGCAGACTACACGGCTTGAAGCC
CGTTTTCGACATTCCCCTGCACGTGCCCATCCAGACGGTGGTGGTCACTCCGGGCAACACCCACATTCTGGCGCCGCTTC
GAGATGGACGCTTGGCGGTGATCGCCGTGCAACTGCCCTCCGGCGGCAATAAGAAGCATTCCGTGCTAAATGTTTAGGTT
CAGCAGGAGCTGGAATGAACACTGGCTGACCTACTGAATTCCCGAATTTTACCCTCTGCGTAGCATAAGTTGTAGTCGTT
CTAGTTTCCTTCGTTCTGCTTTTAGCGCACCCTAGTTTTATTTATTGTGTCTTGTGTCATTTGTCATGATTTATTTCCTA
TCTGCTTCCCATGCTACGTGACGTGCCAAAAGCAAAGCGCCTTTGACCAACTCAAAAAATTCCCAGAAAGTTGACAAATG
TATTTAGAGGTATTCAGCAAGTCGATCGATTGCATTCGCGGAATTTCCATGCCAACTAAGCATTATTTCCCCATCACATG
TGCGTGTACACCTGTCTGGGTCGGCGATCCTCCATCCTGGGCGGCAGAAATGATGGATGGCACACTCCCCTTCTTCGCTT
GATGAAAATCTGAATGAATAATCAATCATTCCATTTGTGGCGGTGCGGAGAGATAGCTCGGCAAATGGGACGCAGGAGAC
GGGAATTGGAGACGGAAAACTGGTTGTCGACGCACTTGGCAACTACAACTACTTCTCTCTGACTCACGAGCGACTTTCAA
ATTGGCCGAAATACAAAGAAAAAGAATTAAGACCGAAAAAAAAAGCGCCCGGCAACAGCGGCAAGGCGCTTAAAAAACGG
CAACCGTTCGCAGAGATCGACGGCATCCAGCTGCTCGGTTGATCAAGAGAAACACTTTCGTTGCGCCCCTTTTCGCCTGC
AGATACAACCAAAAATAGAGCCCAGAGATCTTTGTCCCATTAAGTACGCACACTCGGATCGCATGGTATTGCTACCCAAT
CATTAACAGAGCATTTCCAATTGAAGTATTATCA***CCACAGCACGATCACAGTCCGTCC***ACACAGATCCGAGATCCGCATA
GGCAGACAATACCCAATACTTGGTGCGCCAGAGCAAGAGATTCTATCTATTTCCATACCACCCAGTACGAGTACCAGCAC
CAGCACCATTCCGATCACCAAACTTTCTGCCTTTGATCTTACGCGCCAGCGATTGTGAAAAAAAAACCCCTCACAGACTC
TGAAATCTGAATTTCCAAAGACATGCGACAATATCAAAGGTGACTTGAGAACATACATATATATTTACGAATCGTAATC
GCATATATAGTATAGAACTTTCGCACAATCGGGTCGTGAGATTAGCCCCCGCTTTGGAGCCAACCTATCAATATCTATCT
ACACCAGCAATATATAGTATATCATCTGTGTACGTAAAGGTCCTCCGGATCGAGATCGTTATTTGTTGTTAAGATCGTAT
TGCAAAATATGGCAAAACTGTTTTACCCGTTCAATGTATGTACGTAACTGTACGAGTTTATGTAGATAATGTGACTATTC
GTAGTTTGTAGATCCGTCAATAAAGTGATGAAAAGTGTATGTACTCGTA

5. CG31195

AAACAATATATCGATACTATCGGACAGGTGGCCCATCTCTGTTTGAGGGCGTAGTTCCAACAAGTGCTGAGCATCACAAT
TTTCTATTACTAAGCCCAGCTTTGCGTTGGCGCGCCCCAGAATCTCATTTTATATTTAGTTTCTGCCAGTTTAGTTAATT
AGTTAGTTGATAGTGTTGTTTGTTTCTTCTGCAACAATTGTGTGCGATAGGAGTCGGGCAAA**ATGTTCCCGTCGTCGATT**
TTGGGGCGCAGCTATTTGCTTTTTATGCTGGTGCTCGCCGTGGGCGTGTTCGCCCAACACGAGTGGCAGGCCCGGGATGC
CTTTGATGAGATAAAGAGGCAGTTCGACAAGGTGAACGCGGATAACTGCCCCATCCAACACCATTCGGACCTTTTCATGC
CCATGGACGCGGTGTCCCACAAGCCGGACATCAAGGAGATCAACGTGAATCCGGTGTTCCCCAACCGAACTGCCCTGCTG
CATCTGCAGAATATGGCCCTTAGCAGAAGCTTCTTCTGGAGCTACATCCTCCAGTCGAGGTTTATTCGACCCGCCATCAA
CGACACCTACGATCCCGGCATGATGTACTACTTTCTGTCCACCGTAGCCGATGTATCCGCCAACCCACATATCAACGCCT
CGGCCGTGTACTTCTCCCCCAACAGCTCGTATTCGTCGTCGTATCGCGGCTTCTTCAATAAGACGTTCCCCAGATTCGGG
CCAAGAACCTTCAGGCTGGACGACTTCAACGATCCCATTCATCTGCAGAAGATATCGACGTGGAATACTTTCGATGTTCA
GGATCTGGGCGCCCATCACCCGGACTCCATATCCAAGGACTACACCCACGACCTGTATAAAATAAACGAGTGGTACCGCG
CCTGGCTACCAGACAACGTCGAGGGACGGCACGATACGAAGATCACCTACCAGGTGGAAATCCGCTATGCGAACAACACA
AACGAGACGTATACCTTCCACGGACCGCCTGGCTCTGAAGAAAACCCTGGTCCGATTAAATTTACAAGGCCGTACTTCGA
TTGTGGCAGGTCCAACAAGTGGCTGGTGGCCGCAGTAGTGCCAATTGCGGATATCTACCCCCGACACACGCAGTTCCGTC
ACATTGAGTATCCCAAATACACGGCCGTTTCGGTTCTTGAGATGGACTTCGAGCGTATCGACATAAACCAGTGTCCATTG
GGTGAAGGCAACAAAGGACCTAATCACTTTGCGGATACGGCGCGGTGTAAAAAAGAAACGACAGAGTGTGAACCATTACA
AGGCTGGGGCTTTAGGCGCGGTGGCTACCAGTGCCGTTGTAAGCCAGGTTTTCGGCTGCCCAACGTAGTGCGGCGACCTT
ATCTGGGCGAGATTGTGGAGCGCGCATCGGCAGAACAGTACTACAACGAGTACGACTGCCTTAAGATTGGCTGGATCCAA
AAGCTTCCCATTCAGTGGGATAAGGCCTCCTACCACATTCGCCAAAAGTATCTGGACCGGCATCCGGAATATCGCAACTA
CACCACCGGCTCGCGATCACTTCATGCTGAGCACTTAAATATTGATCAGGCGTTGAAGTATATTCATGGAGTCAACTATC
GCACTTGCAAAAACTTCCATCCGCAGGATCTGATTCTTCGCGGTGATGTGAGCTTCGGCGCCAAGGAGCAGTTCGAGAAC
GAAGCCAAGATGGCCGTGAGACTGGCCAACTTTATTAGCGCCTTTCTGCAGGTATCGGATCCCAACGAAGTGTACTCGGG
CAAGCGTGTGGCCGACAAGCCGCTGACCGAGGATCAAATGATCGGCGAGACCCTTGCCATTGTCCTGGGCGACAGCAAGG
TTTGGTCGGCCACAATGCTCTGGGAGCGCAACAAGTTTACCAATCGCACATATTTCGCACCCTATGCCTACAAAACTGAG
CTCAACACAAGAAAGTTCAAGGTGGAGGACCTGGCGCGGCTCAACAAGACGCACGAACTCTACACGGAAAAGAAGTACTT
CAAGTTCCTGAAGCAGCGCTGGAACACCAACTTCGACGACCTGGAGACCTTCTACATGAAGATCAAGATCCGCCACAATG
AAACAGGTGAATACCAGCAGAAGTACGAGCACTACCCAAATTCGTACAGAGCGGCCAACATCAAGCACGGCTACTGGACT
CAACCACAATTCGACTGCGATGGATATGTGAAGAAGTGGCTGGTGACCTATGCGGTGCCCTTCTTCGGCTGGGACAGCCT
GAAAGTCAAGCTGGAATTCAAGGGTGTGGTAGCTGTCTCCATGGACATGCTGCAGCTGGACATCAACCAGTGCCCGGACT
GGTACTACGAACCGAACGCCTTTAAGAACACACACAAGTGTGACGAGCAATCGTCCTACTGCGTTCCCATTATGGGTCGT
GGCTATGAAACCGGAGGCTACAAGTGCGAGTGCCTGCAGGGATACGAGTATCCTTTCGAGGATCTGATTACCTACTACGA
TGGACAGCTCGTCGAGGCCGAGTACCAAAATATTGTGGCTGATGTCGAGACCCGCTACGATATGTTCAAGTGCCGACTGG
CCGGAGCTTCGGGTCTGCAATCCGCTTTGGGACTTGTGGTCGCTCTGATCGGGCTCACGCTCACCCTGCTGTATAGATTT
AGTTAAGCACCGCACGCCAAATAACTAACGCAAACT***TCCGTCCAAACAAAACTCACATCA***AATCTCTCCACAAAAGATAT

AACTACAAAGCAACACAGACACAAAATTGACACCCAAAAACAAAACGAGCTGCCAGCCAGCCCAGCTCTCCATCGTTGCT
CCCTTTGAACATTGTCATCATTTATCTGCAAGGCGATTAAGTTGTAGACATAATCTATTACAATGAATCTGGTTATTTGT
CATTTCTATCAAAAGCGGCAGAAGACCAAGCAGCAGCAGCAGCAGCCGAAGCAAAAGCAGCAGCATCGTCAGAAAAGCGC
AAACAGCCGCGCCTACTGCGCTCAGATTACGGAAATGCCGTGTAGCTCGTATTTTTCTTCTTCGGAAGATCCTTCCTCTC
TAATGTCCGTGAAGTCGCACAGCTGTGTGTGCGTATGTGCGGCGATCGCAAACCTGTTAACATCGGAACCCAGTTAAGCC
CAGGCGTACCTACACCCATCGGGGAGGTCGTCCAGGTCCTAGCTGAGGGTTGTTTGTGCCCGCACACACACCTTTTTTCC
GACCAACGGATGCCACAAAAAATCCAACGCCTGCCGTCGTATTTTTCTTATATTAAATCGTCGATGGTCTGTTGGCTGCG
ATTTTTTTTGCTGAATTAAATATGTATGTCGATGCTGCTGCCGCTGCGGCGTTTTTTCCACTTTTTGTTTTTTGCGCCTT
GATTATCATATGCGCTTGAATTTTTATTATCTGTACCCTGAGAACTTTTTCTATGCGCTGCAGGAACCAGTTTAGCAGCC
ACTTGGTCTTCTACCAGGGCAAAAAGAAATCTCCAGCTTTTTTCATTAAAACTTTATAGCCATAGGTAGCTGGACAGGGG
CAGAAAAAATCTTTTTCACACATTTTTCATAGTCATTTCAGGGCAGCAGCGGTGGGCAGTAACTGTCTCAGCATTCGGGC
AACTCCTCCATTTCCATTTGGAAAAATGCCCGCGTCTGCTAAAACACGTTCGAGTGGCTGGCCGTGTCCGCTTTTGGTTT
TGTTTCTGGAAGTCTTGCCCCCCTGAACATTCCGCAATTTATTTGTAGATCAGTACAAAGCAATGAGAAGAAATCGAATG
TGTAAATTGTGCCTTAAACTGAATATTTGTAACACAACAGACTATATCAAAAACA***TCCGTCCAATGAGTAGTCTACTTT***G
TATTATGTATCTACATATCTGAATTAGTGATAAGTGCCTTGAGACAAAATCGAATAACTTTTACAACCTGCTTGAGTAGT
CTAATGTCTAGAATTTAAGTGTCTTATAATTTTAAAGCAATCCATCCATATATACAATGTTCAAATATATGATTTTGTAG
T

6. CG8121

CCTGGAAATATCTAAGCCCTGATTCGTATGTTTTGTTTTTGATGCGTTGCGTGGTGATTAATATTTCGTTTTTTTGGGCT
GATTAAAGGATTGATAAAGGACTTGCCTTCTTATACACGTGACGAGACTTAAAAGGCTTC**ATGATGAATTACGGTAGGAA**
AACGCCCTCCACATATCGGTCCAATCCGTCGGTTTATTCCCATGCCACGGGAAGATCCTCGACGAATTTACACTCCAAGA
TGTCCCGATCCACGCGATCCGTCAGGATTCCTTGGTACCAGCGACCGTTGCTTAAAAACAATCAGTACATCGATATACAG
AAGGGTGCCATGCTGGTCGGATTGTTTGCCATTTTTCTGTCCCTCTTCACCATCGCCACGAGCATCTTTGACATCTACTG
CTATGCTATGGCTGCGCCAGGATCCACACATTATGGCTACTACATCATATCCTATGAGTTCGTCTATGTGGGCAACAAGC
ATGTTCGCAATATGCTCATTGTCTTTGCCCTGTTCTCGCTTATCATGGCGCTGATAAACTTTGTGACCAGTGTCCTTCTC
TGCGTGGCTCTGCGCAAGGAATACGAGAGGAAGGTTATGCCATGGCTGTGGTCCTTTGCCATATTTACTGTTTGGCGGGC
TTTGGCACTGATCTTCTTTGCCATTGTCAATGATCTGTACTTTGCCTACAATGTGATTATGGTGCTCCTTTGGACCATTT
TTTGTGTACTGTCTATATATGGATGGGCCGTGGTGTACTCTCTGTTTTTGGAACTGGTGGATCTGACCAAGCTGGAGGAT
CTAGCCCATTTACGCATGGGCACAATGGCTTCCCTGCACGCATCGACAGCAAACTCACTTGCAGGATCACGGCCAACCAC
TCCTCACAGCACCGTGTCCACCATGCCCGTGGGCTAATAAAAGTATTTGCCATCCATCTATGGGGCTCACAACGAAATTG
CACAATCTAGACCGTCCGAATGCAGCTCAAGATAACCCTGATGCTTCGCACTCAAACATTGCTTAAACTATAGTGTACAT
TCCATCCACCAGTTTAGTGCCTTAGAGATGCGCCTCTCGAATACCACAAATTTTAACCGCGAATCAGCGTAATTAGTTGT
AAGT***CTATTCCGTCCAAACGCATATGT***TTTTTGTATACTGTTGAAGAATCGCTGTCTTTATGTATGAATGTCAAAATATC
GTGTTTTTTATATTCATATATAGATGTACAACCCGAATTGTAGCTATGTATCTAAAATATTAAGCGACAAGCAATATAAA
AAATACGAAACCGAAAATATGA

7. Mcr

AACGGTCACACTGCATCAGTCATCAAAGCGGGTGTGGATCGCTGTTGGTTTTGTGTTGTTTCATTTTTTCGAATCTTTGA
GTTCCCGAGTTAGTTTTATTTACCTTTCTGTTGTTTAATAAAATGTGCATGGCCTGCTGAGCAGCCAGATTAGATCGAAT
TTCAGTACCGAGCA**ATGATGTGGCACTTGCTGCGCGCCCTGCTCGTGGTAGCCGCCGTCTTGGATGCACTGCAACCGGCG**
GTCGGACAAAACGACAACTACTACAACCCCAACCAGAACCAGCAGAATCCCCAGCAGCCGCTACTGCCCAACCAGCAGTG
GGGTAACAATCCACAGACGAATCAGTATAGCAACAACAACCAAAACTTTGGCCAAACGAATCCCAGTGATCGCCCGCCGT
ACAGGACCGATTCGGGGAGCTACAACGATATTGCCGGGCAAGATGACTATAATAAACGAGTGGGAGGAGGCTATCAGGAC
AACGAGGAGCCGAGTCTGACTCGTGGAAAGTCGTCCTATAACATCAAGGCCACCTTCCTGGAATCGCTGCATTCCAGGGA
GCCCACTTACTTCATTGTGGCATCTCGAATGGTCAGACCTGGTCTAATCTATCAGGTGTCCGTATCCATTCTTCAAGCTC
AGTACCCGATAACCGTTCATGCCAGCATCGCGTGCGATGGTGTCCAAATCAGCGGAGACTCTAAGGATGTCAAGGAGGGC
ATACCGGAGACGTTGCTCATGAGGATCCCACCAACTAGTGTGACTGGAAGCTATAAGCTTCGAGTGGAAGGTTTCTACCA
GAACGTTTTCGGTGGCCTCGCCTTCCTGAACGAAACAAGACTAGACTTCTCACAGCGCTCCATGACGATATTTGTGCAGA
CCGATAAGCCTCTTTATATGCAAGGGGAAACTGTGCGATTTAGGACCATTCCGATCACCACCGAACTGAAGGGATTTGAC
AATCCCGTCGATGTGTACATGCTGGATCCAAACAGGCATATTCTTAAACGTTGGCTATCGCGTCAATCAAACTTGGGTTC
CGTTTCGCTGGAGTACAAACTTTCGGATCAGCCCACTTTTGGAGAATGGACTATCCGTGTAATAGCTCAGGGACAGCAAG
AGGAGAGTCACTTCACCGTGGAGGAATACTATCAGACCCGATTCGAAGTGAATGTTACCATGCCTGCGTACTTCTTCACA
ACGGATCCGTTTATCTACGGAAGGGTGATGGCTAACTTTACCAGTGGCCTACCAGTTAGAGGCAATCTCACAATCAAGGC
CACGATCCGCCCAATTGGATACTTCAGCAACCAAGTTCTAAATGAGAAGTATCGCCTGGGTCGTTCACCTTTGGAGCAAA
CTAACCTATACAATGAGCGATGGCGCTACAATAATCCCAACCAGAATCCGCAAGTGCAGTATAATGTGCCTGGACAACTG
CCACAGGATGGAGCAGATCTTTCACAGGATATTTTGTACAGGAATCAATATGTGGTCGAGCGACACTATCAGTTCGACGA
GGAGTGGCCATTCTGGGTTAGGAAACCCGAGTATCAGGACAGCAGCTACGAGGCGTGGAGTGGAACTTATCGAAAGACCC
TGCCCTACTTGCGCTACTTCAATGGTACCTTTGACTTCAAGTGGCCATTGAGGGAGCTAGAGCTTCTGGTGCCCAATCTG
GCCAACTCTGAGGTGCTGATCACGGCCACTGTTGGTGAAAAGTTCTACGATGAGATCATCAGTGGTTACTCCGTGGCCAG
GGTGTATAATTCCAGTCTGAGGGTGGTATTCCTTGGCGATTCGCCGCAGGTCTTCAAGCCCGCCATGCCATTTACCACAT
ATCTGGCTGTTGAGTACCATGATGGTTCGCCAATTGATCCAAACCTATTACGCCAGGGTTTAATGGAAGTCTCTGGTTTC
GTGGAGAGCCGCAATGGTGGCAGGAGGGACTGGCCCGCCCAGCGATTGCCTATGTCGCAGCAAAGCGATGGCATTTGGGA
GGTGAAGATTGACATCAGGAATGATCTGAACCTGGACGATCGCCCACAAGCAAGAGACTTCTTGAACGGCGTTCAGAACA

TGCGCTTGCAGGCGAACTTTGTGGATCCGCGTGGTGAACGCATTCAAACGGAGCTCCTTTTGGTGTCTCACTATTCGCCC
AGAAATCAGCACATTAAGGTCACCACCAGCACAGAGAAACCAGTGGTTGGGGAGTATATCATCTTCCACATCCGCACGAA
TTTCTACCTCGAAGAGTTCAACTATCTCATTATGTCAAAGGGTGTTATCCTGGTGAATGACCGCGAAACCATTACAGAGG
GCATTAAAACCATTGCTGTTGTTCTGAGTTCCGAAATGGCACCTGTGGCTACGATTGTAGTGTGGAAGATTAACCAGCAG
GGACAGGTTGTGGCCGATTCCCTTACGTTCCCAGTTAATGGCATCTCCCGGAATAACTTTACCGTATATATCAACAATCG
CAAAGCGAGAACCGGAGAAAAAGTGGAGGTGGCTATCTTTGGAGAACCGGGCTCGTATGTTGGTCTCTCTGGCATCGACA
GTGCATTCTACACAATGCAAGCCGGCAACGAGCTCACTTACGCCAAGATCATCACTAAGATGTCCAACTTCGACGAGCAG
ACCAATGGAACGTATAAGCACATTTGGTACTCTCACGAAGGCAATCCCGATGAGCTGGTGTACTTCCCCGCCTCGTCCTT
TGGCGTCGATGCAAATCGCACTTTTGAGTACAGTGGACTGATCGTATTTACGGATGGTTATGTACCCAGAAGGCAGGACA
CCTGCAATCGTACTTTGGGTTTCGGAGAGTGTTTGAGTGGACGTTGTTATCGCCTGGAGAAGCAGTGCGATGGTTTGTTT
GATTGTGATGATGGAACGGATGAGATCAATTGCCATGCGCGTAATGACACAGAGCTGTTGAACTACAGAAAGTACCGATT
TAATCGCGTGCTTCGCCATTACGAAAATGTGTGGCTATGGAAGGATGTGAACATTGGACCACATGGTCGATACATTTTTA
ATGTGGAGGTGCCTGATCGCCCCGCCTATTGGATGGTCAGTGCGTTTAGTGTGAGTCCATCGAAAGGCTTTGGAATGATG
AACAAGGCCTTGGAGTACGTTGGTGTCCAACCATTCTTCATAAACGTGGAAATGCCGGAAGCTTGCCGACAAGGCGAGCA
GGTGGGCATCCGTGTCACTGTTTTCAACTATATGATAACTCCCATCGAGGCGATTGTCGTGCTGCACGACAGTCCGGACT
ACAAGTTTGTGCACGTGGAGGAGGATGGCATCGTCAGATCGTATAACCCCAGAACTAGCTTTGGTGAACATCAATTTTTC
ATCTACTTGGAGGCTCAAGGCACGACGGTGGTTTACGTGCCCGTTGTACCTCAGCGTCTAGGCAATGTGGATGTGACCCT
ACACGTGGCCACTTTACTTGGAACAGATACCATAACGCGAACACTGCATGTAGAATCGGATGGTCTGCCACAGTATCGTC
ATCAATCCGTGCTACTGGATCTCTCTAACCGCGCCTATGTCCTAGAATACATGCATGTTAATGTAACTCAAACGCCAGAA
ATTCCATACCAAGTGGATCGTTACTTTGTGTACGGCTCAAATAAGGCTAGGATATCCGTAGTGGGTGATGTGGTTGGTCC
CATTTTCCCCACCATGCCCGTAAATGCCAGCTCCCTGCTTTCCTTGCCCATGGAGTCGGGCGAACAGAATGCTTTCAGCT
TCGCCGCCAATCTCTACACGATTATGTACATGCGATTAATTAATCAACGCAACAAAACGCTGGAGAAGAATGCCTTTTAC
CACATGAACATTGGCTATCAGCGGCAGTTGAGTTTTATGCGGCCCGATGGCAGCTTCTCACTGTTCCGTTCCGATTGGAA
CAACTCCGATTCGTCCGTCTGGTTGACTAGCTACTGCTTAAGGGTGTTCCAAGAGGCCAGCTTCTACGAATGGGAGAACT
TCATCTGGATCGATGCCACAATTATTGAAAAGAACATGAGATGGCTTCTGCAACACCAGACGCCACAGGGATCCTTCTTC
GAGGTCACGTGGCTGCCGGATAGGAAGATGAATCGCACCAACTTTGACAAGAACATCACGCTAACGTCCCATGTACTCAT
AACCTTGGCCACCGTAAAGGATATCTCTGGCACTCTGGGCTCTCGAGTTGCTTTGGCCACGCAGCGTGCCTTGGCGTATA
TAGAACGAAATATGGACTTCCTTAGACACCAGGCGCAACCTTTTGATGTGGCCATTACAGCGTACGCCCTGCAGCTTTGT
AACTCTCCGATTGCCGAGGAAGTATTCGCCATCCTGCGTCGCCAGGCCAGGACTATAGGCGATTTTATGTACTGGGGTAA
CCAGGAAATACCTCAACCTCCACGCAAGCTGGAGAATCAAAAGTGGTTCTCGCTGCCCCGTCTGCCATACGAATATGATT
CGCTGAATATAGAGACAACCGCCTACGCGCTATTGGTTTATGTGGCGCGCCGTGAGTTCTTCGTGGATCCCATTGTGCGA
TGGTTGAACTCGCAGCGCTTAAACGACGGCGGATGGGCATCCACCCAGGATACCAGTGCTGCCCTCAAGGCTCTTGTGGA
GTACACAGTCCGATCTAGGCTGCGCGAGGTTTCCTCGTTGACCGTGGAGATCGAGGCCTCTTCGCAGGGCGGCAAAACAC
AAACGCTTTACATTGATGACACGAATCTGGCGAAATTGCAGAGTATCGAGATTCCAGATGCCTGGGGCACCATCAAGGTT
CAGGCCAAGGGTGCTGGCTATGCCATCCTGCAGATGCACGTGCAGTACAATGTGGACATTGAGAAGTTCCAGACCAAGCC
GCCAGTGCCAGCCTTCGGACTTCATACCAAGGCAATCTTCCACGGCAGGAATCAGTCGCATATCTCCTATGTGGCTTGTC
AAAATTGGATAAACCAAAACGAGTCGGAGCGCTCAGGCATGGCGGTCTTGGATGTGGCCATACCGACTGGCTATTGGATA
CAGCAGCAAAAACTGGATACCTATGTGCTCAGCAACCGTGTGAGAAATCTGAGAAGAGCTCGTTATCTAGAACGCAAGAT
TGTCTTTTACTTCGATTATCTTGATCATGAGGATATCTGTGTAAACTTCACCATTGAGCGTTGGTATCCAGTGGCCAATA
TGTCTCGCTATCTGCCGGTGCGCATTTACGACTACTATGCACCGGAACGATTCAACGAATCCATATTCGATGCCCTGCCC
ACATATCTGCTGAACATTTGTGAGGTGTGCGGCAGCTCTCAGTGTCCGTACTGCTCCATCTACAACATGGGCTGGCGCGC
CTCCATGTCCATGTCCCTGCTCTTCTTCAGCGTATTCATATACCTACTGCGAAGTCGCACGCACCTGGTCCTAAACATGA
TGCAACTGCTCACATAGAAAGAAAACGAAACGCTATTCACAAAGTCACCCTCGAAACAACTTGCTAAGATTCGTAATGTT
TTTGTTCATTTATTGAATGTGTTTACTTTGTAGTTTTTTGTTTTGAGTTGTAGTTTTTTTGTTTGCCAACGAAAT***TGCAT***
TCCGTCCAAAAATGCTGGTCAACATTTTGGTTTTATTTTTAGTTCTTTTCAGTTTTACTCCACTGACCCGCTGTATAATT
GAATGTACAAAACAGCAGTTAAACGTAAATTAGATTTTATTTTTATTGCTGAACAAAGAATCTTCTGCCACACGTAGCAG
ACCACATGTAGCCACCTAATGTATCTTGTAAGTCGTAACGAACCGTACTATGCCTTATCATAGAGCGGGCTCCAAGTGCG
GAATGAAAAAATCAAACAATGTGTCTTTCACAAAATGCCTTTAATAGATTTTAGAAATCATTAGCGAATGTATCTTGACA
AAAGTTTGCAAGCATATCGAATGGAAATTTATCTAATTTGTATTATCTAACTCTTGAATACATTTTTAAACGAAATGAAG
TGAAAATCGTTGAAGAAACCGTCCAATTAATTTATGCAAATAATGAATCTGCATAGTTTGTAAGTTTTATACGTATATAC
GCCTTCATATGAACCACAATTAGTTTATAAATTCCCAACACAAATCTCACATAGCCATGTACATGAAGCTACTAAGCGAG
AAACAAGGAAATTTTTGTACACAACTTAAGAATGTCGCTCCTTTCCGTACCATAAGAATCGAATCGAATCTCTATGAAGT
GTCTTGTGTTTTCTTTTGTAATAAACGAAACCTGAAAATAAATAAAAGTAATGTTT

8. Tsf2

GTTACACTGGCTTCCTTGAGCTGGGCTTGGGTGTGGTGTTTTGTTTGAGTAATTGTTTTTGCAGATAGATCTTTTTAGTT
AATACACTTATTAATATTCTTATATCCCGAAT**ATGGCTAGCAGCCTCGTTTTTGTGGCTCTAGTCGGCGCCTTATGTTTT**
ACGCTCGCTAACGCACAACATCACTACGATGAGCACAAGACCACACGGATGGTGTGGTGCACCAAGAGCCAGGCGGAGCA
GTACAAATGCCAGAACCTTACGGTGGCCATCGAGCGGGATCGGGCACTATTCGACGAGGTGTTCCTCAATCTCACCTGCT
TCATGGCCTACAGTGCGGATGAGTGCATCCATCACATTGATCGCGAGAAGGCACACATCACCACGCTGGATGCGGGCGAT
GTCTTCACTGCTGGACGGTACAACTCCCTCATACCCATTATGCAGGAGAAACTGGAGGGCGGCTTTGCGGACTATCAATC
CGTAGCCGTGATCAAGAAGGGATCTCTACCAGACTTGAACAATCTGCGCGACATGCGCAACAAGAGGGTGTGCTTCCCCT
GGGTGGGCAGCTTGGCCGGTTGGATTGTGCCCATTCACACGCTCCAAAGGGAGGGCGGCATGGAAGTGGTGGACTGCAAC
AACCAGGTGAAGACGGCCGCCAGCTATTTCAATAATTCGTGTGCTGTGTACTCGCTTTCCGACAAACACAATCCAATTGG

CGACAACTCCGATAAACTTTGTACGCTTTGCACTGGAAAGATACCCGGAGGACGCTGCTCATCTGCAGATCCCTACTTCG
GCTACGAAGGCGCCTTCAAATGTCTGCTTGAAAAAGGAGATGTTGCCTTCTTGCGCCATTCGACTGTGAATGAAATGCTG
CAAACCACCGAATTCAAGAACATAGCACCCGACACTTTTGAGCTGCTGTGTCGTGATGGTCGTCGTGCCTCCATCAACGA
TTATCGCCAGTGTAATTGGGGCCAAGTGCCCGCCGATGCCATTGTCACATCTTCGGCTCGCAGCTTCAGCGATCGCAAGC
AGTATCAGCAGTTTTTGAAGCGCATTGCCGAGCTGTACTCCGATGGCACACGTGATGATCAGAGCAGGCAGGGAGGACAA
AGCTTCAACAGCCGCAACAACATCAACGATCAGAATGCCTATGGCCAGTTCGATAATAATGATCCGTACAGAACGCAGAA
TCAATACGATCAGTATCGCAGCGAACGATTGGACAGCAGTTTTGCAGAGGAAAGAAACCAGCAGGACGGCACGAACACTT
CCATACTCTATGAGAAGTTCCGCATCTTCGAGTCGAAGCGCTATGGCAAGCCAAATTTGCTGTTCCAGGATTCTAGTCGC
GCTCTAACCGTGATTCCCGAAGATGATCAGTCATTTACCAAGTACCTAGGCCCTGCCATTAACTTTATCTACGGCATCCG
TGAGTGTCCAGTTCCAGCGATGACGCTTTGCGTGACTTCGGAGAATGAGCTTGATAAATGCATCAAGATGAGGACTGCCT
TGAAAGCTCACCTCCTGAAACCAGAACTCATCTGCAAGAAAATGCACTCCCACATCAACTGCATGCAGTTCATCGAAGCG
GGCAAAGCAGACATCTCCGTTTTCGATGCCGGCGATGTCTACACTGGTGGCCTCAACTACGACCTGGTGCCCTTCATGTC
GGAGGTGTACAATCTGGGCGAGCCCGAGTATTATGTGGTTGCCGTGGCCAAGGAGGATGATCCCGATACGGAGCTAACTT
ACCTGAAGGGTAAGAATACCTGCCATACGGGCATTAACACCGCCGCTGGCTGGACATATCCGATGGCGCTTTTCATCTCG
AACGGCTGGATTCGTCCCTACGGATGCGATTCGGTTCGAGCTGCCGCGGAGTACTTCACCAAGTCGTGCGTGCCGGGTGC
CATTAGCAATGAGTACAACACCGGTGTGCCCTACGACAGCATGTGCGATCTGTGTCATGGAACTAGCTATCGCTATTGTC
GTCGCGATGCCTCCGAGGAATACTACGGGCACACGGGTGCATTCCGTTGCCTCGTCGAGGGCGGCGGTCACGTGGCCTTC
ATGAAGCACACCACGGTGATGGAAAGCACGGGCGGCAAACGCAAGGAATGGTGGGCCAGGAATGCCCTAAACGATGACTT
TGAGCTGCTCTGCACGGATGGTACTCGTGCCGAGATCCAGGACTACAAGCGCTGCAATCTGGGCAAGGTTAAGGCCAATG
CTGTGGTCACCCGCGGCGGAGTCAACTACAACGAGACCCAGATGAATGCCTACATCAATCTGCTGACCTATGCCCAACAA
CTGTACGGTCGCAAGGAAGTGGACGCCTTCAGTTTCAGCATGTTCTCCTCGCCGATCGGGCACTATGATCTGATCTTCCA
GGACGCCACGCGGCAGCTGCAAGTAATTCCGCCCAATAAGCGCAGGTATGATGCCTATTTGGGCAGCGATTTTATGCGAG
CACGCCGCATCACCGACTGCTATGCTGGTGCCTCCCAGTTGGCATTATCCGTGGGCCTGCTCCTCGTGGGTTCGCTGGTT
GCAATGCTCTAAGCCACTAGACTCACTTAGATTTCAGAATTCTACAAGCATCTCTAGCACAAAGAGAAAACATATCAACA
TTTTTACATTCCAAATATGATTAAGTTAAATGTGAAAT***CCCTTCCGTCCGAAAATGAAACT***CTAACTAGCCGAAAAATAT
CTGTTGAAGGCTATAGTTTATAATTTATAAAGTCCTGTGCCTTATTATAGTATTTGTTTGTGTACATACCAACTAAGACC
GTCCAAAAAATGTACCTAGGAGATGTAAGCCGAATCTTTTACAACCGCTTATGTAAGTATACAAATAAAATAACTAGATA
AAGG

9. cals

ATTCATATATACTAATGTAATACATATATGTAATCCAACAATCAGTTTCACTGTGGTACATTTTATAATGTTTAATCATA
TATAGTCACGGGAACTAGAGTTTATACACGGTCCTCGAATAAAATGTGTTTTGGAGCTCAAAATAAATATATTTGGGCTT
GATTATTAAAGAACTTCGAATAGAGCC**ATGACGTTTCACAAAACCTTTGGTTACGGATGCATTGTATTAATATGTTTTGA**
ACTCCTGTTTGCTGGAGTTGAAACGTCCAGCGAAAATGATGATGAATATTTAACACAGAAAGAAATCATATTAGAAAAGT
CATATCACGGTTTAATTCGAGAAAATGAAACACTTGTAGAAATAACTCCCCTTATTAAAGTGAACGAAGAGAAAATTTGT
AACTTCCATATTCTCAAAAAACCGTATCACGAAATTCCATTTAAGATTGAACTGGTAAATAACCTAGGTATTTTAAAAGC
ACGTCGGACACTAAATTGCGAGAATCGTAAAAGTTATCACTTTGAAATCTGTGCAATCTATTGTGATGGAACACCTTCAA
ATACAGCCAATGTTCATATTACTGTTATCGACGTAAATGAGTATGCTCCAACATTCTTAGAACCGTCTTACGTAATTGAA
GTAGACGAGGGAAGACTTTATAACGAGATCCTAAGAGTCGAAGCTTCCGATAAAGACTGCACTCCGCTCTTTGGTGATGT
TTGCAAATATGAGATTCTTAACAATGATGAACCATTTTCAATAGATAACGAAGGTTCAATTAAAAACACTGAACCACTTT
CTCACAAAGCTTCTCATAACCACATTCTGTCCGTGGTTGCCTATGATTGTGCAATGAAAGAATCTGCACCCATTATGGTC
AGCATTAAAGTTCGACGCGTTTGTGAAACAAAGTTTGTAGGCATGCCAGAACGCATTGACTACACGTCTGGAAGCACCGA
AAGCCTTCAATTGTTTCCCAATGCTCGCCTAGACTTATGTGACATTTCTTGCAAAAATGAAGAAGACCTTAGGATTCATT
CTTCCATAGCCCTTAAGGTAAGCACACATATTTTTGTAATAATAATATTAATTCCAAATCACGATTTGTTCTACAATTTA
TGGGACCATTAAAATCAAAAACACCTCTTACCGAGTTGGGTTGGGTACCGGTGCAGATAGCATCTTCAACACACGATATA
ATAGATATCAACTTTTCATAAACCGTGTGGTTAACAATAATTTAAATATTTTTTGTGGGGGTGATACCCCCTAACAGCC
GCATCAGGACAAAATTGTATAATGTGTACTTTAAGCTTTTCATATTTTTCCTCCTGAACCATATCATTTATTCAGGCTTG
GTATGCAATCCCTTTAGTATTTTCATAAATTTGGGATTAACAGTTTTCTGCGGCTTGTATGAGTTCGAGTTGGCATTCCA
TTTCCATCGAAATTGGAAAGACATGTAATATCATATAATATAATATCTTATAATATTATATAATATAATTATATAATAGA
TCCAATCACTTTTCAAAAGTGTGAACTTGTTAGTTTTGGGCAGCTTGTGGCAACCATCTAAAACAGAAGAGTTACTATAA
ACTGCATGCTCAGTCTCAACTCTCTGACTTTGTAGTTCCCGAGGTCAGCGTTCATAGACAGACGGACGTGACCAGATCGT
CTCGGGCCCAGATGAAGAATATATATGTATACATTCTGACTACTTTTCAACTTTTTACTCTTGGAGAAAATGTTTTATAA
GGTCTTTTTAATGTTTCAAATAAGTATCTAATCCGAAATATATATTTAAGAGGATAAATGTTACATATACCCTGTTTTTT
AAAAAATGATCTGGCAAGATACCAACTTTGCGGTTATGTTACTTCACGTGTTTGCTTTAGGAACCATTTTCGTAAACCTG
TGCATCATAATGAACCAAATATGAGTATAAGTTGTTAATTTCATTTATATTTTCAGACAAAGCACATCTCATTTGGGTGC
GATCGGGATATATCTAATTGCACTTCAGGCCAAAAAGTGAAAGACCTTTTGCCACATGGGGCAGAATGGACTAAAGAGTT
AAGTTATGACGAAGGTCTAGAACCCATATTTCACTTTGATGGGTCTACTGGAGTAGTAGTTCCTGCAACTGTTATTGATC
ATTACGATTTTTCATCGCAACCTTTTAGCATTCTTACCTTATTTCGACACAACAGTCAGGTTGAAATTAATAAACATGTA
AAGGAGCATATTGTCTGCAGCGCGGACGACCACAAAATGAACCGTCATCATATGGCACTATTCGTTCGCAATTGTCGCCT
TATTTTTTTATTAAGAAAAAATTTTAACGAAGGTGATTTAAATATCTTTAGTCCTGCTGAATGGCGTTGGAAAATACCGG
AGGTCTGTGACAACGAATGGCATCACTATGTTCTCAATGTTGAAGATTCGTCCAAAGTAGATTTATTTATTGACGGCGTA
CGTTTCGAAAATTCTATTGAAAATCGTCATAGCAACCCAGAAGTAATTGACGATTGGCCCTTACACGCAGCCCACGGAGT
AAATACTTCCTTAGCTATTGGTGCCTGTTACCAAAGCTTGGAAAATAGACTTAAACATGGCTTTAATGGAGATATTTCAG
AAGTGAAGGTCTCCCTCAACAGTGTTTTGACGGCAGAGGACATTAAATGTGGAACAACTTGTGCCGAGCATTTATTAGCT

CCAAAGCCGCTGCAAAGTAACAATGAAAAATCGTATTCTGATAACAGCCAGATTAAAGAAAACATTGAAATGAACGAAAT
TTATATTTCGGCGAAAAACAAACATGATATTGAACAATTTATGCGAAAAGTTCAGTATATTAATACTAAACAAAAGCCCA
CGGTAGGACGTCGAAACATAGAAGTGCTTACAACACTGAATTGTAAAAATGAAAGTTCCCTTCGCTTACCGCCAATTGAG
ACTTATATCATGGTTAACGAACCGATAGCTCCACTCGGGATCGATATTGATGTCGTCTCAGCGTCGGTAAATAAAAAATT
ATACCGTTATTAAGAGATATCACTAAAAAATGTATATCAATCTTACAGCTGGAAACCTCCGATCTAACTCCGCCATCGTA
TTCCCCCAAAATAGCAATATCTGGTACGTAAACTACACTATGATAACATTGCTTAGATGGTAGTTTTCAGTCATATGAAA
CAATTCTAATTTAAATTTGTGACGGCCGTGTAACGTACCGTGTTTTATTAATTTTTAAAGCCATAGGCACTGTTGCCGTT
AGGGCCGAAATCGCAGTCGGCTACTAATAAAAATTTAATTTCCTCCATATTACTTTTTTGAGAGATAAATCGTGCGCAAG
AGAGTTGGTACACGCATCGTGCACCCAATGCAGTCGGCTCTACTTGCGTTACCGCCTATTTTGTGATGTGTTTCAACAGC
AATGAATTGTTGAACTGGAAGTATAAAACTTAGGCTATAGATGTAATTAATGGTTAATTTTTATTTCATATTATATTGTA
AATTATTGCATTACATGAATGAGTTTTACATTTATATATGTTTATACGACTCTGATAGTTGTTGGAATCACGAATTGCAA
AATAAAAAAGCAATTTGAAAATAATACAAATGGTCTAAAAACTGTTGTAATTGGTTGGCGTGGTCAAAGTTTACTAACGC
GTCAAAACAACTAATACAAATTATCATTATATATACTAATACAAATACACAATTTATTAATTCATTTTGTAATGTTATTT
CCTGTTGTATGATTTTTAGGAACTTCAAACAAGCTTGTATCATATCAAGAAATTAAATTGGGCGTACACATTTTGGAAAA
AACTTGCATTGACTCGGTATCTAAAAACAATGGAAAATTAGAAGAGAAAAATCATATCGACTCATGCAGCGTCGTTGTAT
TTCCCTCTTTGAATCCCGATCATGAAGACATTAAAATAGATGGTGACGAGTCATTGTCATCTAGCATGGACATTAAGACT
AATATTAATAAGGATGGAGTCGAAATGATTGGAAAGGATACCATTAGCAATTATATAAATGTATTACGATCTCTTGTATA
CAGTAACAAAAAACCTGCATATTATCTTAACCGTGTATTTAAGCTTTCTTGTGCCCAACAAAGTTCCCAGTACAAAAGCG
GTGAATATACACTAACACTTACTGTGTTGCATCCTAAACAGACATTGTTTAAGTCTACAAATGTACTCCCTTCATCGTTA
TCTAAAGTAAACTTTATTGGCAACACCGATAATGAAACATCATTTCACCGGAATAGTGGTTCGGTAAATGGCAATGATAA
TAATCAGCCAACAGGTGGGTTTCCAATACTTATATATAAAAATATGTATAATATATTTAATCCTAATCTTTTTAGAGTCT
AAGGTATATTCATATTCCCTTCTGCATACCAATAATGTGCAAGAACCAAAATCTCACATTCATTCTTTCATACACAAAGG
TAAATGGCACTGTAAAATAAGTTTATAAAAATATATAGAAAATTAAATACTTTTTAGCTGAAGGGTCACATGTTACAATG
CTTATAATTCTTGTAAGTGTTTTTTTGGCGGTCTTACTTTGTGGAGTCTCGATTGCACGTTTAAAAAACAACCAAAAATA
TATTGAACACCATCAACCATGTCCAAAAGTAAGTATATTTACAACCTGAAATTTGAACTCATTTTAGTATACCCTTGCCA
ATGATATATTTTCGTTCAGAAGTTTGAAATACAAGACACTCTTGGGTCCATATAGTGTATAATGTGCTTGATCAACAGTT
ATAGCCGATATAGCTATGTCTGTCGTTCTGTCCGTCCTTTCAAATATGTGCATCGTATTATATTTACATTTTTTCATCT
GGAATTTTCCTACTATTGAATTTATATTTTTTAGATTTCTGATGATGGTTTAATTTGGGATGACTCAGCTTTGACTATAA
CTATAAATCCCATGCAAGCAGATGTTACATCGGACGCGAGTTCTGAATCAGAAAATTCTGAATCTGAAGATGAAGAAGGT
ATAGAATATATTTATTAAATATTATTTAAATTCTTAAGAGATATCTTTGGCATTAGTAGCTTTAAAGGACGGATTCACTC
ACATCAATCAACTCGAATGGGATAACTCTAATATTTTCCAACAGTAAATAACATAATGAATATTTTTCTCAACACTGGTA
CTAAAATATGTAAATGTGCTTAAAAATATAAACCACTAAATCAACAAATATGCAGAAGTTATAAATGAGTTCCAAAGAGT
CTGCATATATGGCTGCAGGCATAATTGTAAAGACGTGCATTTATAGTTTTTTTGCAACAAAAAACATTTTTCTTGCAAT
TGTTTAATTGTAAAAACTAAGTCAGTGAAAGTGCATAACACATCATATGAATGTACGTAAAATAATAGCGCAAACAAAAA
TATAGCTAAGGTATAGTACAATTATTATGTAACCAGCGTCGCTAGATCCATAAATAATAAAAGCTATAGGCATACAAATA
TACACATAAGATCTATTTAATTTATAGTTTGTCAGCCCTCACCTTAAGATGTAAAATGCACATGTCTACCAAATTTCAAC
TGAATTCTGTCGCAATGCATAGGAGGAAG***TTTCTCCGTCCCAATAATTTAAG***CCTAATAATAAAACATAAAAAAAAATTTG
AAATGGATCGTAATACCCAAACAATGTTGACTTATTTAATAAAGCTAAAGTTGTCATTGATTTATTATTTTTTCAAGTAT
ACAACTTAGATTAAGGAACATATCGAAGCTATAAAAAATTTATTTTCAACCGACCATAAACTTGTGAAATTGTACTTTTA
AAATAGAATTTTTAATTGGTTTATTTAATGGTACTATTTATTGAAACATAATTAGTTTCCATTTTGAATGGCGAC

10. emp

AAACAATTCACAAACACAATCGAATATCAGAACACCAGTACACTGTAGTGAAGCCAAACAGTTGGGAGAGTGCTCAAAAC
CCTTTGGAACTCTTGAAATCTTAATGGAACTCTGATCGTTGGCCATGCAGTTGGACGACATTCTGCATACAACAAATGCG
CCACTTTGTACGACAGTTTACATAAACAGAAAGGTCGCATGCAACCCGGTTAGCCTTATTGGCATGCAAAGTCACCGCGG
AGTCAAGCTTCGGATACGGGTCTCCATTAAGTTAACAACTGCAAGGCGGATTGCTCCTCACTCTCCACCACACCCAACCC
CAAAACCGACTTGGTCAAC**ATGAACGGGCCGAAACACAAGTTTTGCACAAAACTCTCCAGCACCTATCTGCGAAAATGGT**
GGATTACGATTGTCGTCGCAGCGGCGCTGATAATTGGCGGCATTGTGGTGGCCTGTGAATTTACCGTGCTGATTGATGCG
GTCGTGGACCGAATGGTGGCCCTGCGTCCCGGCGCCAAGACCTTCGGATGGTGGGCCAAGCCCCCAGTGGAGCCCAGAAT
TAGTCTGTACATCTACAACGTAACCAATGCGGACGACTTCCTCAGCAACGGCTCCAAGGCCATTGTCGATGAGGTGGGAC
CCTACGTTTACAGCGAGACCTGGGAGAAGGTGAACATCGTAGAGAATGACAATGGCACGCTGAGCTACAATCTTCGCAAG
ATCTACTCGTTCCGCGAAGATCTGTCGGTGGGGCCCGAAGACGACGTGGTTATTGTACCCAACATTCCCATGTTGAGCGC
CACCTCGCAGAGTAAGCACGCGGCCCGATTCTTGCGTCTAGCCATGGCCAGTATCATGGACATACTGAAGATCAAGCCAT
TCGTTCAGGTGTCCGTGGGTCAGTTGCTTTGGGGCTACGAGGATCCTCTGCTCAAGCTAGCGAAGGATGTCGTTCCCAAG
GAGCAAAAGCTGCCCTACGAGGAGTTTGGCCTGCTGTATGGCAAGAACGGCACCTCCTCCGACCGAGTAACTGTGAACAC
AGGTGTCGATGACATTAGGAGATACGGCATCATTGACAACTTCAACGGTCGCACCCATTTACCCCATTGGACCACCGACG
CATGCAATACTTTGGCCGGAACCGATGGCTCCATCTTCCCCCCACACATTGACCACGATCGCATCCTGCATGTTTACGAC
AAAGATCTTTGCCGGCTCCTGCCCTTGGTGTTTGAGAAAGAAGTAATGACTTCAAACGAGGTGCCTGGATACCGGTTCAC
CCCACCAGAGTGGGTTTTCGCCGATGTGGACAGCCACCCGGACAACATGTGCTTCTGTCCCGCGGGCAAGCCGTCATGCT
CCCCCAACGGACTTTTCAACGTGTCGTTGTGTCAATACGACTCTCCCATCATGCTAAGCTTCCCTCATTTCTACCTGGCC
GATGAAAGCCTGAGGACGCAAGTAGAAGGTATTTCGCCACCTATGAAAGAGAAGCATCAGTTTTTCTTCGACGTTCAGCC
TAAAATGGGAACTACCTTGAGAGTACGCGCCCGTATCCAGATCAATTTGGCCGTTAGCCAAGTGTTTGACATCAAGCAGG
TAGCCAACTTCCCGGACATCATCTTTCCCATCCTTTGGTTCGAGGAGGGCATCGACAACCTTCCCGACGAGGTCACCGAC
CTGATGCGCTTTGCGGAGCAAGTGCCACCTAAGATCCGCGTGGCGCTAATCGTCGGTCTATGCGCGCTTGGTGTGATCCT

ACTACTGCTTTCGACCTTCTGCCTGATCCGCAACTCGCACCGGCAAAGCACCCTGCACTTGGAGGGGTCCAACTATCTGG
CCACCGCCCAGGTGGACATGAACAAGAAGCAGAACAAGGATAACCAACCCGCCAGATACTAGGCGCTCTAGTCGGTGTCT
GGTCGCAGTACGAGTGTCTTCGGGTGACTTCGTAATAGTATAAGCCGTAGCTAGTAACTGGGACCTGCCCACTTCCACTT
CCACCCCCAATCCCTATAACGATACCCAATCCGACCTCCCTATGTTAATCCCATTCCCCGCGGAGGAATGTGCTTCTATG
TAGTTTAAAAGAAATGGAGCTGGGAAGACTTGTATGTATTTGTATGTGTATCTGTATCTATTGTATTGTATGTAGGTTAT
CTGTGTAAGTAAGCGTTATAAAAATTTTGAAGTGTAGTCTAACGAAACAGTCGTGATCGCTTAAAGGCTCAAAGCTTCGA
CTTAGACAATATTTGTTATACCAAAACAGCAATCGATCCTGTTCTTGCTTTCTTAAACCCTCTTGAAATGAAATTGTATG
TACAATCAAGTCACAAAACTCGAAACGTTTCTCAAATAAAACAAAAT***TCCGTCCTCGAATACAAATGTGCT***TTATAAGAC
CAAATGCTGGGCATGTTCCGCATATGAAATTTATATTCTATAAATACATAACTTTATCGATCTAATGTACAATTTATATT
CATTAATGTGTACAATAAATCACGATATTATAATTAGAGATTGGCC

11. IqfR (Epsin)

TTGGAAACACTGTTTGCAACACTGGACACGACAGCTCACCAAATATTAGCTGCTGTGATTTTGTTTTACAATTTTCGTTT
AAATTTTTACATTTTTGTTTGCGGAAAGCGCGGGTGCGAGACAGGTGCGCCGTTTCCATGCAAAAGTGCAAATCGTCAGC
TGCAAAACAGTCAGAAAACGGCACGCCAAGCGAAAACGAGTTCGACGTCTTGTTGTAGTTGGATTTTCCAGCCACTTTTC
GACGAGATTGTGTGAAAAATTCCGTAGCATTCAATAGTCTCAAA**ATGGTGGATAAATTCATCAGCATGTGGAAAGTGCGC**
GAATTGGCGGACAAGGTCACCAATGTCGTGATGAATTACACGGAAACGGAGGGCAAGGTGCGGGAGGCCACCAACGATGA
TCCTTGGGGGCCTACAGGACCCCTCATGCAGGAATTGGCCTATTCCACCTTCTCATACGAAACATTCCCGGAGGTGATGT
CCATGCTGTGGAAGCGCATGCTGCAGGACAATAAAACCAACTGGCGACGCACGTACAAGAGCCTCCTTCTGCTAAACTAT
TTGGTGCGAAACGGCTCTGAACGGGTGGTAACCTCCTCTCGGGAGCACATCTACGATCTGCGCTCGCTGGAGAACTATAC
ATTCACCGACGAGGGCGGCAAGGATCAGGGTATTAATGTTAGGCATAAGGTACGAGAGCTTATAGACTTTATTCAGGATG
ATGATCGTTTGCGCGAGGAGCGCAAAAAGGCGAAGAAGAACAAGGACAAGTACATCGGCATGAGCAGCGACGCCATGGGC
ATGCGAAGCGGTGGCTACAGCGGCTATAGCGGTGGATCTGGAGGAGGCGGCGGTGGCAGCGGTGGCTACAATGATGGCGA
CTATCGCTCTAGTCGTGGAGACAATTGGTACTCCGACAAAAGCGCCGACAAGGATCGGTATGAGGATGATGATACTCACT
ACGATGGAGAGCGAGAGGGATCCGATAGCGACTCACCCAGTCCAAGACGTAACTATCGATACAATGACCGTGCGAGTCCT
GCCGAAGTAGCCAGCGAGGCCAAACCTTCCAGCCTCAACATGAACATTCGTTCGAAGACCGTCAGTTCCCCTGTCTCCAA
GCAGCCCACTTCAACGGCTTCTGCCAAGCCAGCGCTGTCCCAGAAGAAGATCGATCTGGGTGCGGCAGCAAACTTTGGAA
AGCCAGCTCCTGGCGGTGCTGCTGGCATTCACTCACCAACTCACCGTGACACTCCCACCAGCGTGGACTTGATGGGCGGC
GCTTCGCCATCGCCGTCTACTTCCAAGGCAAACAATAATACGCAAAGCAATAACAACGATCTGCTGGACGATCTGTTCAA
GACCTGCTCGCCACCGCCGGGGCAGGAGAAGACGCTGAACAGTGCTGCCGTGATTGTGGATGACGATGATGACTTCAATC
CGCGTGCCAGCGATGCTAGCCAGCAGGAATTCGGCGACTTCGCCTCTGCTTTCGGGCAGCCCTCGGCGGGATCGACGATC
AGCGAGCCACCATCAACGGGCCTGGTTCCGGCTGCGAACGATGAGTTCGCCGACTTTGCGGCGTTCCAAGGCTCGACAAC
GTCGACATCTGCGCTGGACGGTAATTTGCTGAAGACTGCCACGCCGGCGAACGATTCGTTTGACCTGTTTAATTCAGCTC
CCACCTCGACGGCAGCAGCCACAACGGCTACAGATCTCCTGGCAGGCCTGGGCGATCTGTCCATTCACCAAAGCATGCCC
ATGGACAATATGATGCCTCCCATTCCCGCCGTCACGGGCAATAATCTGCTCCAGCCCATGTCGGTGACCAATAATAACAA
TAATACCAACGGAGGCGCAGTCCCCGCCGCTGCCAGTGTCCAGTCTACCGCTGTGGGCGCCACCTGGTCGGGCGACCTGA
AGGGCGGCAAGATGAACATTGACCTGGACAATCTGCTGATGAGCAAGTCGGGCAAGCCCAGTGCCCCGGCCCCTTCGATG
AATGCCCTGAAGACCAACAGTCCGGCAAAGGCGCCACTGAATGTGCAGACGGGTGGCGGATTCCCTGGACTGTCGCCAAT
GACCAGTCCGAACATTTTTGGGGCTCCGGCACCGCAGCAAAGCATTCCACAAAACCAATCAGCATTTGCCAACTTTGGAG
CTTTCCAGCAGCAGCAGCAGAATCACAGCAATAATAACAATAATAGCTCGTCGGCATTCGACTTGTTTCAATGATATTTT
TTAGCCTAATTTAACCCAACCAACCTACTACCCAAAATGTCCACTCTCACATTCACATTTCCACGATCGCTAAACTTGTC
AGTTCTATATATTATTTACGTTTTCCTTTCCCCTTGCTGTACAAAGTTTGCGACACGTGCAATTTGTTTCTAAATTCGTT
GTTAAACAATTTACCCCCGATGCAATTTTCCGGATCAAACCCATTAATT***CCACATCCGTCCTGATCGTCCAC****TCGGAA*TT
TGAAAGTCGTCATCAAATGCAATACACAGAGTAGTTGAATAATGGTTGTACTAAACGGATTCGTTAGCACAACGCGCGGA
CGGACGGCTGAGCGATCCAACATTGAAATTGTTAAACAAAATACTACACGTAATAAAATCAAACAATTTTAGCAATAGTT
AACGTTACGATGTGTCTGTATATAAATTGAATGTAAAGCAAATATGAAATATAAAACAATTGAAACC

12. Ptp99A

AGTTCTCCAAAGCACGCGCGTGTATAAGCACGTATGTAGATTCATAGATACATATATCGGTAGCACCAAAAAAAAAAAAA
AAATAAAGAATCGAGCAAACCAACCGCACAATAATAAACCTTATCAATAAACAACGCTGCGAAAGAGAAGCGACAAGTGA
CGAAAGACATAGCCAGTAGTGTGCTCGTTTCGTCACAACAAGAGCAGCCTCATTGAGGCAAATTCGTGTGACGGCGTGGT
GTTCGTAGTTATTGCGTACTATTTTTGCAGTGAACTGGCGACACTGCAGTTGCCAATATGACGAGTGACGTAATTTGTGC
AAGTATGTAGGCGCAGATCGCGGGTACACGCAGCGAAAAAGGGAGCCACACTCTACACACAT**ATGTCGTCAGGTGGCGGG**
GAAAAGCGCTGCAGCAGCCCCATGCTGCATGCCACATCGGAAATCGGTGGTGCAACTACTGCAACGCCGCGCCTGCTGCG
CCGCAAGTCGCAGCGCGGAAATTGGTTTACAAAGTGCTTCCATGCGGCCTACTACTATCTGGACGACCCGCCACACCATC
CAAATGCCCCGCAGGTGGACTGGGAAGTGCCCGTTAAGATCGGCGACGAGATCCGAGCGGCTGTGCCCGTCAACGAGTTC
GCCAAGCACGTGGCTTCCCTGCACGCGGACGGGGACATTGGATTCAGCCGGGAGTACGAGGCCATCCAGAACGAGTGCAT
CAGCGATGATCTGCCGTGTGAACACTCGCAGCATCCGGAGAACAAACGCAAGAATCGCTATCTCAACATCACGGCCTATG
ACCACAGTCGGGTGCATCTGCATCCCACACCGGGACAGAAGAAGAATCTGGACTACATCAATGCCAACTTCATCGATGGC
TACCAGAAGGGACATGCCTTCATCGGCACCCAGGGTCCATTGCCCGACACCTTCGATTGCTTCTGGCGCATGATCTGGGA
ACAGAGGGTGGCCATCATCGTGATGATCACCAATCTTGTGGAGCGCGGGCGGCGCAAGTGCGACATGTACTGGCCGAAGG
ATGGGGTGGAGACCTATGGCGTAATCCAGGTCAAGCTCATCGAGGAGGAGGTCATGTCCACGTACACTGTGCGCACCCTG
CAGATCAAGCACTTGAAGCTCAAGAAGAAGAAGCAGTGCAACACCGAAAAGCTGGTCTATCAATACCACTACACCAACTG
GCCCGACCACGGAACACCCGATCATCCCCTACCCGTGCTTAACTTTGTCAAGAAATCCTCGGCCGCCAATCCCGCAGAGG

CTGGTCCCATAGTCGTGCACTGCAGCGCTGGCGTCGGTCGCACTGGCACATACATCGTCCTGGACGCCATGCTCAAGCAG
ATCCAGCAGAAGAACATCGTCAACGTCTTTGGCTTTCTGCGTCACATTCGTGCTCAGAGGAACTTCCTGGTGCAGACCGA
GGAGCAGTACATATTCCTGCACGACGCTCTGGTGGAGGCCATCGCCTCGGGGGAGACCAATCTGATGGCCGAGCAGGTGG
AGGAGCTGAAGAACTGCACTCCCTACCTGGAGCAGCAGTACAAGAACATCATCCAGTTCCAACCGAAAGACATACACATT
GCATCCGCCATGAAGCAGGTGAACTCGATCAAGAACCGCGGCGCCATCTTCCCCATCGAGGGCAGTCGGGTGCACTTGAC
GCCCAAGCCGGGCGAGGATGGCAGCGATTATATCAACGCCTCGTGGCTGCACGGCTTCCGGCGACTGCGGGACTTCATAG
TCACCCAACATCCCATGGCGCACACGATAAAGGACTTCTGGCAGATGGTCTGGGACCACAATGCGCAGACCGTCGTGCTG
CTCTCATCACTGGATGATATAAACTTTGCACAGTTTTGGCCAGATGAGGCCACGCCCATCGAGAGCGATCACTATCGCGT
CAAGTTTCTGAACAAGACCAACAAGAGCGACTATGTGAGCCGCGACTTTGTCATCCAGTCGATACAGGACGACTACGAGC
TGACGGTCAAGATGCTGCACTGTCCCAGTTGGCCAGAGATGTCCAATCCCAACAGCATCTACGACTTCATCGTCGATGTG
CACGAGCGCTGCAATGACTATCGCAATGGACCCATCGTCATTGTGGATAGATATGGTGGCGCCCAGGCGTGCACCTTCTG
TGCCATCTCCTCGCTGGCCATCGAGATGGAGTACTGCAGCACGGCGAATGTGTACCAGTACGCGAAGCTGTACCACAACA
AGCGACCCGGGGTGTGGACATCCAGCGAGGACATCCGCGTCATCTACAACATACTTTCATTTTTGCCTGGCAATTTGAAC
CTGCTGAAGCGCACGGCGCTTCGGACTGAATTCGAGGATGTGACAACGGCCACGCCGGATCTCTATAGCAAAATATGCAG
CAATGGTAATGTTCCACAGCATGTCATACTGCAGCAGCAGCAGCTGCACATGCTGCAGTTGCAGCAGCAACACCTAGAAA
CACAGCAGCAGCAGCAGCAGCAACAGCAGCAGCAGCAGCAGCAGCAACAACAGACAGCACTCAATGAGACAGTCAGCACA
CCAAGCACCGATACTAATCCAAGCCTCTTGCCCATTCTGTCATTGCTACCGCCCACAGTCGCTCCTCTATCCTCCAGTTC
ATCCACCACACCACCACCACCAAGTACTCCAACACCACAGCCCCCACAAACCATCCAACTGTCATCGCATTCGCCATCCG
ACCTGTCCCACCAGATCTCATCCACAGTCGCCAATGCAGGATCACCAGCAACTGCAAGTGCAGGCGCAACACCAACGACT
CCAATGACACCGACAGTCCCACCCACAATACCTACAATACCATCACTCGCATCCCAGAACAGTCTAACCCTTACTAATGC
CAATTTCCATACTGTTACTAATAACGCTGCTGATCTGATGGAACACCAACAACAACAAATGCTGGCCCTGATGCAACAAC
AAACGCAACTGCAACAACAATACAACACGCACCCGCAACAACATCACAACAACGTCGGTGACCTGCTGATGAACAATGCG
GACAACTCACCAACCGCTTCACCAACGATCACCAACAACAATCATATAACCAACAACAACGTCACAAGCGCAGCAGCGAC
AGATGCTCAAAACCTAGATATTGTAGGCTAAACTAAAGATAAATTTACAGAGAGGGAATTTAAATCATTTTATAAATGTT
TAAATATAAATACTTAAATTTAAACCTTACACCTACGTACATAGCGATGTATTTATTAAAGTCTAAGTCGTTTAAGTGAG
AACAGAGCGTCTAGCGCTGTGTCCATAAATTATTCAACAATTCGTTTGCCCTGTAAGCTCATATTTTACTTTAACTTACA
CACCCACACTCAGTTGTTTCCCCCAAAACTATCTAAATTATTTTGTCTTATGTAGTAGTTTTTATATCGTAAACATAGGT
TTTTATACACCATACTTTACCCACATTTAGTCACGAGGGAAAAACAAAACAAGAACGAAAGTCAAAAGCCGCAGTGAAGA
ACAAGAATAATCGCAAATCTAAAGTGGAAACGTAGTGGAAATGCACAAATCGAACAAGTAGAGTTAATAACATATTTGTC
TAATCATTTAAATACGCTGATTACACATAACTAACGTTAATGCAGGCTAATGCTGCACACACTTGTGCCATGATTTTTTT
AAGCTCTCCCAAGCAAATTCCTATTAAAGTAATGAATATTAGTTGTTGAATTGAATTAGTTGATTGAGCAATCAATTTGC
ATTTTAATTCGAATCGATTCAG***TCCGTCC******TAGTATCTGTTATTCTT***TTCATTTTGCCTTCCTAACAATTGGTTCCACTTT
AAGACAGTTTAATGCAAAATGACAAACTTAAGCGAACCTAGCTACAAATTAATTGCGAATTTAGTGGGCACTAAGCCGAA
TTACCGAGCACTCTACACCACCCACACCACACACATCACTGTAAATCCCAATCAAAGCTAAGTAAATCACACGATTTCCG
CACAAAGCACAAGAGCATCACGAAAATTTGTAAATACAGCTTAGATCAGATAAAAGATACTGTATAAATGTCAGAAGTCA
GAGAACCCTTCACACTCTGCAAAGCTAATAATATTGTAAAAATAAAAACTAATTTAAGCGAACGCGACAACTATAAGTGT
AAATCGGCAAAGTAGTCGCAAGCAAAGTATTATTAAACTATATGATACGTGTGAAAAGTGAGATAAAAGAAAATCCCAAT
GAAAACCCAAGCTTAAGTTTTGTTTTCCTCTATCGATGCGTAAATCTAGCCTGTACAGTTCTCTTAAAGTTAGACCCGAA
TGAATTATGATTAAACCTAGCACTCTGAATGGCCATAGCCAAATGGTTTGGAGCTCTCCAAATCGGGCAGTTAGAAAGTA
TATACCCCCCACAAGTAATTTAGTGAATATTTTTACATTTTATATGTAAAGTATTGGTGGAAATGAAGAACATACGAAAC
GATGAACAAACATGTTTTAAAGACTGGTTTTTTGTACACTTTAACTTGAATAAGCATGCATACTGATACTGATTATGTAA
ATGATTATTATGAACGAATGTCAAAGGTCCTTCAGTTGCCCCTCCCCGCCCCCGCAAGACCTGTCCCGAATGTAGTTTCC
ACTCAATCGCATGTAGTCAGACTACTTGCGGATGCAGACCGAGGATTTGGGAGCACACTTCAATATCCGATCGTATCAAT
CCAATCATTTGTCAGCTGATACCAAATAGGCGGCTCTACATGTTAAACAACCAACTAATGACCAATTTTGTGAGATTATG
TTTCTTAGGCACTAGACCCGACTTAGGCTAAGACTACTTAAACGCTAGAAGGGCTTGTAAATTTCTCATACCGACACCGG
CGGTGGTAACAAATGCTTCATCTGTAATATAACGACAAGAATCATATTATTTCGATACAATACTAAGGCAGGCAATACTA
TGCATTTTAACTGTTAAACACACATAAATAGTAGTGCGTATGATGCAACTATAGGTGGATATTTCCATGCATTATGTACA
TAATGTTGTAAGTGAAAAGAAAGAAAGAAAGAAAAGCTTAATCAACTGCATTGCGAATGAAGCAACGAACACCTTTTGTG
TGATCACACAGAGTTCTACCGAAATCTCCCAATTAAATGTGCACACTCTTTTCGAGCAAATGGAATCCTTTCCGCGACTA
AACTACCTTCACACATTAACTAAAGTAATTGAGAACGATAGAAACAAGGCCACAGATACAACAGATGATTATCTCTAATT
GACGGTGTTATGCAATTTATAAATGTGATGAATGTCAAAGTTAAGCGTAAACAATTTAAAAGCAAGCGAAGAAAATACAA
AAACACAAAAAAAAATACAAATAAAAATAAATATAAATATAAATATAAATATAAACAAACGATTCATGAGAAAGAGCTGA
ATGTAGCGAGAACTTAAGTATCTAAGCATGCCCACGATTTTAGAGCTAAGCAAATAGATGAAGATACAAATACAAATACA
AATACCCAGAGGCAGACCATAGGTTTTAAAGAACGAACGAAGCATTTGCCCCACATCCTGACAGGTTACCGCAGCTTGTA
GAGGCCATTGCAGCCGGAGGAGTCGCCACAGAGATCGGACAAAATCTCGCGATAAACAGAATTAATGATATGAGTATATG
TATGTATATGTATGTACACCTAAGCTAATCGTAAGCCAATGAAAGTTGAAATGGACTAAAGATACATGGAACAGACAACA
TTGAAAGCGTCTAAATAAAGAACATCAAATTGC

13. Sema1b

TCTAGCCCACGTTCGGTTCGCGGTCCGCTGGTCAAACTCATGATAGTAAAAAAAAAAACAAAAACGAAAACAGCCGAAAA
CACAAGACACAAAACACAAGTAAGCCGGAAAGAAACTGGCGAGAAGAAAGTGTGCAGAATTCACAAGTAGCCGTTATATA
AGTGGATTTAATTATCTGATTCGGTTTTGCGTTTTCCCTTTTGCCACCAACAGCTAATTGCATTAAAATAAAACCAACAA
GTTGTTCTGCTGGGCGTAAATCATTGTGTCGTTGTCGCTGACACTCGAATACTTATAGCCAGATGCTGAAGCAGCAGCAG
CAGCAGCAGCAGCAGCGACAATAACACCAGTAATCAGCACAACTATCGACGACAATGGCAGACATCGGACATCAGAATGC

GAGGCGTCAGTGATTAAAAGCAGTCGGAGAGGCCAGCCAGCCATCCAACCAGCCAGCCAGGCAGCCCCATTGGCACCACG
AGCAAAAAGCAGCTGAATCATCAGAACTCGGAGGAGTAGTGGAGTACTAGTTTCGCGCATCTCCTACTACAAGCCAGCAG
A**ATGAGCTCCAAGAGCAGACCTTCATCGGCGGCGATGTTAGTTAATGCGATACCCATGATACTGCTGATCACCTTATCCG
GCCTGACGATCGTGGCAGGTTGGATGCCCGACGTGAAGCCTGATTTGCAAACAAAACAAGACAAAGTCCTAGCCCATTTT
ATAGGCAACTCGACGGACTATTTCAAGATTTTGGACCACAACGATGAGTTTGTTCTAGTTGGTGCCAAGGACGTCATCTA
CAATGTGAGCCTAAATGGCCTGAAGGAGATTGCGCGTCTGGAGTGGCACAGCACGGATGCGGACCGGGAGCTGTGTGCCC
TAAAGGGGAAGCACGAGTGGGATTGCCATAACTACCTTCGCGTCTATGCACTGCGTCCCAATGGCGAGGTGCTGCTCTGC
GGCACCAATTCGTATAAGCCGCGCTGTCGTCACTACACGCCGGTGGAAGTGTCATCCGAGGAGGCCGGCTCTGCTGGTCA
CGCCCATGCCATGCGCTACGAAGTCAGCCGAGATGTGGAGGCCCAGGGCCTGTGCCCCTACAGCCCCGCCCACAATAGCA
CGTACGCCTTCGCCGACGGGCATTTGTACAGCGCCACCGTGGCGGACTTTTCGGGTGGAGATCCTCTCATCTACAGGGAG
AACCTGCGTACCGAACAATATGATCTCAAGCAACTCAACCAGCCGGACTTTGTTGGCGCCATCGAGCGCAACGGTTACGT
GCTGTTCTTCTTCCGCGAACTCTCAATGGAAGTCATGAACTTTGGCAAGGCCGTTTATTCGCGCGTCGCCAGGGTTTGCA
AGAACGATAGAGGTGGTCCCTACAGTCACGGCAAGAGTTGGACTTCCTTCCTTAAGGCGCGACTCAACTGCTCGGTGCCC
GGCGAGTTTCCGTTCTATTTTGACGAAATCCAGGCCATTAGTCCGATTGTGGAGAGCGGATCCAAGTCACTGATTTATGC
TGTCTTCACAACCTCTGTGAATGCCATTCCTGGCTCGGCCGTATGCGCATTTAACGTAGACGATATACTGGCCGCCTTTG
ATGGAGAGTTCAAGTCGCAGAAAGATTCACAGTCCCATTGGCTGCCAGTGGAGCGTGAGCAAGTGCCGAAACCACGTCCA
GGACAGTGCGTTGAGGACTCTAGAACTCTGACCAGCATCGCGGTTAATTTCATTAAGAACCATCCCTTGATGGAGGAGGC
TGTGCCAGCAGTGCACGGTCGTCCGCTGCTGACCAAGGTGAATCTGCACCACCGACTTACTGCGATCGCAGTGCATCCGC
AGGTAAAGTCCCTAAGTGGAGCTTACTACGATGTTATATACAGTGGCACCGATGACGGCAAGGTGACCAAGTTCATCAAT
ATACTGAGCACTCACCCGAACTCAACTGTTGATCGCCTGAAGACAGTGGTTATATCAGAGATGCAGGTCCTTCCACTTGG
AACGCCAATTAGGGAATTGGTGATCTCCACTTCGAAAAACTCGTTGGTTGTGGTTAGCGATGGCAGCTTGGTTAGCGTGC
CCCTGCACCACTGCTCTCACATTGTCGACTGTCTGGGCTGCTTAAGTCTCCAGGATCCCATTTGCGCCTGGGACTTACAG
ACACACGAATGCAAGAACCTGGCTACGAGTCAGCACAAGTTCGGAACAAAGACTTATTTGCAAAGCTTGAATAGCACCAA
GAAAGCCGCTGCCTTGCTATGTCCTCATATTCCCCGAGATGCACCCGGTGCGGAAACAGTAAGCTTTGTTACCATGGCTC
CACCGCCAACAGAGGAGCAGAAGCTTTTGTACTCCAATGTGGGCTCGTCGCAGGGCAATCAGCCAAGCTTGGAACAACAG
CCAACTGGTGGCGATGACTTTGGTTTGAACAAGATCACCCTTACGTCCATGGATCCCGACGATATTATGCCGAATTTGAA
CGATCCCCAAAAGTCTACGGCCAGTGTAGATGAAATCAAATATGCTTCAACGCCCAGCTTCATGCTACTTACAACTATCG
TTTTCTTTACCTTCCTTGTGGGTGGAATCGCAGGAATATGCTGTGTGAAGGCTAAGAGCCGCCTGATGGAGGCCCAAATG
GATGAAAGTCACCGCAATCATTTCGGCAAGCCAATGCAATTTAAACATCAAAATACTGGCAAAGACATCAACCTGTTAAT
GGGCTCCAATGCCTATACGACCACACAAAAAACACCTAATCTGGATTTAGAAAAGGATCGCAGTCATGAGTGCAAAAACT
CCACGGAACATTTGGAAAAAGAGCTGCCCTGCAAAACGTCAACGCTGACAAAAGTGAAACGCACTTACATCTAG**TGGCCT
TGTGAGGGCAGCCAACTGCAAAATTAGTCGAATTTCTATACTCTAAAACATATTTTCGTTGTTTATGTTGCTGTTAGTTA
TAGTAACCCCCCACCCAATTTGCGAATAATGTAAACGACAGTCTCGGATGCGTTTGAAACGCATCCGAAAGATAGGAAGC
AGAGCTGTTGCGTCTTTTTTACTTAGCTTGTATTGTAATTTAATTTGATGTAAAATTTGCGTAAGCTTAGTTTCTTTGTC
AAAATTTAGTTAAATTTATTGACAACGTAGTGTGCGGGCTGCCGCAGCCTGGAGCTAACCCGAAGCATTCCGCATTCCCC
TGACGTCGACCTTTGCTGTCCAAAATAGGAGTGCCGGCTCTTGGCTACGCAGCCCGATTGTGTCGCCCCTTATGTGTACT
CTTAATGTATAATCTTTAAGTGCAAATATTAGAGCTGTACAAATTTAATCACATATCATGCATACCGAAATCGAACCCGA
AGCGGACCAGCCCCAATTATTTCTCGAAATTCAATCGAATATTCAACAAGTATTTTGTTAGACTGAATGATTAATTATTC
TATATGAGTTTGTTTTGTGGAGAACTAACAGAATGCGACAGAGACTTTAAAGCTCTATGCTTTGTGGCTAACAAAAGCGT
AAAATTGTATCTAACATAAGCTCTATAAGGAATTGAACATAGAATGTAATTAAAAGTACGAACTAGGCTAAGAAACGAAG
AAAACTTCTATGAGGCTGTTTGATAAGAACTGACACCGCAGCCCCCACATCATCAGATGGAAAAACGCACAAGAAAGCGA
AAATAAATGAACACTGCAACAACAGCAAGCCTTATAAATAGTATAAAATCGTGTCGAATCTAGGCCTTAACGCTAAACTT
GTAAGCTTTGGATGGAAATAATGAGCTTGGTTGGCAATCAACGTAAATCAGCATTTTAATAATTTTATGTTTAATTTCTA
AG***CTGTTGTCCGTCCCACTAAAGAA***GTTGCATAAATACAATGTACAATGGCAATTTTATTTTTAAAACGTGTAAAATGCA
ATTTTCATCGTAATTAATATATTTTTATACGTATATGTACGCCTAATGTCTAAATCTTAACATAAGAAAGTCTTAAAAAT
GGAAAATCCCGATAAAAGCTATATATAAAATATTTTTAAATCTTACAAAAAAAAACGTAATGTAATGATTTCTCATATTG
TGATAGATTTTATTGAAATTTTAGAAAGATGAAATGATATGCCTTTTGGTTGAAAAACAATGATATATATGACGAACGAG
TCTGATTGCATGCATGATAATATTGCGTAGGAATGGGTCTTCCACTCGGAGGATCAAGAACCAGAGAAAACCCTTCATGG
TGTTTAGTTTTTATTACTGTGTATGTCCTTGTAATGTGTATATTGTTGTATACATCCGATAAGCAATTCGTAAATCTAAT
AAAGTCGTCATTTTTTTCATAACTC

14. CG10217

CCAGTGTTAGTAAGGTCGCCTTTGCCCATCATTGTTGTTAGTTCTGAGGACACCAAACACGCA**ATGCATTATTACCTGGC
TGCTATCTGCCTGGTGGGCTTAATGGGCTCAGCCCTCGGTCAGTACAACCTGGTGCAGAAGGACAACTGCATCATTCCAA
AGATCCTGCAGGGATCGTGGTTCTCGTGGGAAACGGGCCTGCCCACGCTGACGGTGATCGACGCCACCTCGATGTCCAGC
CGAGGCTACTGCATCAACTACATGCGCCACAAGGGCGACGAGTACTCATTTGTCTTCAAGGAGCGATCCAAGGACTGCTA
TCACTGTGTCAGGACCATCATCCGCACCCTTAATGTGTTCGAAAAGTTTGAGGGGCCCTGCGTGGGCATACCCGCTGGAC
AGGAACCCACCGTGGACTATGTGTGTCGTGGTGTTAAGGATGACCAGCAGTTGATAACGCTCTTTAACGAGAACTTTGTG
CCCATCAACTGCCGATCGTCGCTGGAGGGAGTGTGGCATTTTACGTACCAGAACCGATTCCGATTCACGGGCGTATGCGA
TAAGCCCGACGCTCGCATTCAGTCCTGTCAAACGGCAGGAACGCAGTTCCTCATCCAGAACCAGAAGTTCAACGTCACCT
ATCAGCAGTGCGAGGGTATGGAGGGCACATTCACCGGCACCGTGGAGTTCAGCTGCCTGGGCGATTGGTTTGTGGGCAAG
AACCACTACTTTGCCGTGGCCAACACAAAGGAGTCGCGTAAGGATGAGAAGTACCGCTGCTTCCTTAAGAATCGTGACGA
TGATCTTTACGTGGGCGTTTCCATTACGGCCGAATGTAACACACTGAAGACGCCAGAAACGTCACCGGAGCGTCTAAAGC
TGACGCCAGTGAAGGCTGAGTTTGTGGAACCCGGTTGCACGCTACCACAAAACTTTAGCGGAGAATGGGTGAACACTGCC**

AACATTGATGCCGATGTCTCGATCAGTGAGACCCACATAAACGAGACGTACTATCCGGATAAAGCTCGATACCGAAAGAC
AATCTATGTTTGCCGAGAGCGACGGGGTAATCGTGTGATGATGGCTCGCCTCACTGTCGACGGTTGCCAGAAGGATTACG
TGTGCTTTGACTTTATGCCCCGCCACCACAACATCATTCGCTACCGCAAGGGTTTGGCTGTGATCAAGGACGATTTTAGT
ACAGTCTGCTCTTGGGTTCAGTTCCCCAATTCGGAGGCATGGAAATATGATCTGTTTCTTGCTCGAAATCCTGTGCCTGT
TCGTTGCCCCGTTGCGGGAAAATTCAACTTTACGCAGCGAGGAGAGCATCCCTTCAGAACGAGAATTCTTGGTGGTGTCA
CTCTGAGTCCGCGTCCCGATATCCATTGCAAGCAAAACATCTCGGATTTATCGGTTTGCGATACAGATCAAAAGGAATTG
GCGGTGGATGAAAACTATTGCCTGTCGGTGGATCACTTGGGTCGTCCCGTTGATATCTACAGTGATCCCGATTACCGCAT
GAAGTGCATTGGCTTCTGGAAGGAGAACCTCAAGTCCTACCTGATTACGTACGACGATTTGGATCCGCTGTCCAAGTATC
GCTGCTGGGTCTATCAGCGTGCCGACCTCAACCGTGTCCTCATGTCGCAGGCTGTGGGCGCATTCTGCAAACTGGAGCAA
GATGTGACCTCGTGGAACCACTCGGAGGGCGCTGCCGTAGCCATCGATGCCGTGGAGTACGAAAGGGAGCGTGACGACTG
TCCCATGCACTTCGATGACGGATTGAATCCTTGGAAGCCGTCGGATGCCTCCAACATCATATTTGATTGGGACTTTTACC
GAGCAGGAGCAGCGACACTAAAATTTCAACTGACTGGCCTCGTTATCGTGGTCTTAGTCCAGGTTAGGAGTTTATTAGCT
TAATAATATCAAAAGTATTTTTTAACTATTCCATCCAACGAAATTTACAAAAATTTTACAACCAACATAAAGTCACTTTT
AGGAGCAATTGCGTGCCTTAGAATGCGAATTTTAGAAAGTTTTAAGCAAATTTTAAACTTAGTTTGTAAGATCATCACAA
TTTTTACGTGTAACAG***CCATTCCGTCCCAAACGAATCTC***AAATGTATTGTGCAAATAATTAAAGCAGTTAAATAAATGCC
TTGAACT

15. Ilp8

CCGCTCGTGATTATCAGGTAACGTCGATCGGACGGACGGGTTAACCATTCAGCAAGTTAGCTCTGCAGCAAGTTTGCCCG
CCAAAAGAAAACACGTATACGTAGTGTTGCAGCCAGAAAGGTGATCGACGTGCCTACTGATCTATTGATCTATTGAGAAT
TGTGATCCTAGAACGCCACTAAA**ATGAGTTCAAAGTTGCATATGTGCCGCTGGATGCTGCTGGTCATCGGAGTCTGTTGC**
CTGATGGGCAGCTCGTCGGGCAGCTTCTGCTCCCTGGAGCGGATGAAGAAGTTCGCGATGGAGGCGTGCGAGCACCTCTT
TCAGGCGGACGAGGGTGCCCGCCGCGACAGAAGGTCCATCGAGTTCGCGCACCACCATCTGAATCGACTTGGATCCGGCA
AAACGCACAACAAGCATCACTACATCAGCCGAAGCAGCTATCCAATGGGTGGCTACCTGAAGGTGACCCGGGAGCACTTC
AATCGCCTCAGCGAACTGGACATCTTTCCGCGCTACAAGCCCATCAAGCCGCACCACGAAAAGAAGCATCGCTTCAAGAG
GGATCACTCGAGCAGGTCCTACAACAACATTCCCTACTGCTGTCTAAACCAGTGCGAGGAGGAGTTCTTCTGCTAAGAGA
GCTCCAGTGTCATGCTCCATGCCCCACGCTCCAAGTCGGTG*CCATCTGGTCAGAATCTTCTGA****ATCCGTCCATAGTTATA***
GTTAGGCTATTCATGTACATTCTACAAGTCGCGCTCACTCTTATAGAGTACCTTATTTGTGCCCACTCCCAGGGACACAG
ATCCATTTACAGTATTTATACACTTAGATCGATTTTGAATAAACAATCGAATACGGCAACTGATCGG

16. CG4313

GCGACTGCCTTCGGGCCCACTTTTTGGGTGGGAACC**ATGTCGGTGTATGGATGCGGAGAATTCAGTTTTAGTACAAATTC**
GGCGCAGGAACGAGCAACGCGTCTTTTGCCGAACACAAAGTGCCGCGCGATGTTCCCACCAAAATCCGGACCAACTCCGC
ATCCGATCTCGATAGCGCTGCCTCTTTCGGATCCCTATGCTACTGATCACATGGCGGATCAGGACGCGGTGTTGCCGGTA
CCCCTTTCCGACAGCCTGGACCTGGACGTGGACGTGGACCTCAATCTGAATATGAATCTTAATCTCAATGTAGACGACAG
GCGCCAGGTGCTCTTCGAGGGCTATTCGGACGAGCTGCTGACCATCGCCTGGGTGGCCTGCATAGTGTTCATCATTGTGG
GCGTTCCCGGAAATTTACTGACCATAGTGGCCCTGTCGCGAGGTCGTCAAACCCGCAACTCCACGGCCATATTCATAATC
AATTTGTCCTGCTCGGATCTACTCTTTGGCTGCTTTAATCTGCCATTGGCCGCGTCCACTTTCAAGGAGCGGGCGTGGAC
CCATAGTGATCTGCTGTGCAGGTTGTTTCCGATGCTGCGCTACGGCTTGCTGGCGGTGTCCCTGCTCTCCGTGTCGCTGA
TCACCATCAACCGGTACATCATTATCGCCCATCCCAGGCAGTATCCAAGAATTTACCAGCGACGATATTTGGCTTTGATG
GTAGCCGGCACATGGATCACCACGTTTTCCATAATGATACCCACCTGGCGGGGCGTATGGGGTATCTTTGGCTTGGACGT
CAGCATCGGCTCGTGTTCCATCATGCATGATCGGTATGGTAGATCCCCCAAGGAGTTCCTCTTCATCGCCGCCTTCATGG
TGCCGTGCATCTGCATCGTGATCTGTTATGCCCGAATCTTCCTGCTGGTGCGAAAGGCGGCCATTCGCGCGGGAACCGCT
GGCAAGACCAATGTCAGCGATGTGACTCCCAGCTCCGCGCCGCAGCATCAGATTCAAGCCATGGCCACACCAAAGAAGCC
GGAGAAGGTGACCACGTCCAGCGGCGAGGCGAACGAACCGATCGCAGGACGACCCTTTGTGGTGGAGGAAAACTTAGCGT
ATATCGATGACAATGCTTCGACGGACAGTCTGCCGATCTCGTACAGCATACGGAGGAGGGATCAGGACCAGCAGCCACCG
CCTGTTGATGCCAATGTGGTGCTCAAGGAGCGGGAGAAGGAAAGGGATCGGGATCAGGAAAAGGTCAGCCTGGGACGCAG
TCAAACGCAACTAGAGATGGGCAAAACCCATGGAAAGAACCCTATCACTACATCCCTGCGCACCACCTCCTTCACACGGT
TTAGCCCGCGGAAATCCCACTACGCCTCCATGGGCAATACCTCAAATGCATCCTCTATTTACCCGGGAAGGATGAGTGCC
AAGGACCGGAGATTGCTTAAGATGATACTGGTAATCTTCGTGTGGTTTGTAATTTGTTATTTGCCCATCACGGTGGCCAA
AATATGGAAAAGCGCCACCGAGGTGCACTGGTTCAACATTGCTGGCTATCTACTTATCTACTTGACCACCTGCATCAACC
CGCTGATCTACGTGTTGATGAGCTCCGAATACCGAAGAGCTTACTGGAACTTGCTGCGCTGCCATGGCTCTCCAGATACC
CAGAAGCAGCGGAATCAGGCCAATGCAAAGCGAAAGCACCTGGAGTCCAATCGGCAGGTGAAGGCCACGACGTGACTCCG
ACAGATGCCTTAACACC***TTCATCGTCCTCCGTCCTAGCGG***GATATGATATATGTGTGCCATATGGTAGTAATACGATGGG
AGAAGTACCCAAATGTCATGTTGAATGATCTGCGGTAAGAAAAAATTCCTTGATCCCCTGGTGATTATAGTGCCTCCAAA
AAAGATTTCTAGTCACATGATCCGGCCGGTCGTCCCTTCCACGCACACACGAAAACACAGATCACCCATATATATCTATA
TCTATATCTATATAATGTAGGAACTCCAGAAGTATATTGCATTTTATACCAAATGACTTTAATATCTGGTTTACATTTGA
ATTCCATGTTTAAGAACAATCGATGTTTGTTTAAATTTTGTTTTTTTTTTCTTTGCTTGTCGCTAATTATTGTACCAATA
CATAATAGATATATCCTTATATGGATACGCCCTTTCTATGCCCCCAATAAATGCTTCGAATAAT

17. CG6583

TTTTATAGCGATCTACCATATGGTCACACAGTAACTCGCGTTATTTATTTTCTTGTTTTAATTCTATTTTATTTAAGTTA
AACAAAGCGCACAAAACAGTTTTAAGTAAAAA**ATGAATGTCCTGCTCCGTTGTACGTTCCTGCTGCTCACCCTGGCCATA**
TGTGGCAGCTGGGCCATTCGATGCTACCAGTGCTCCTCCGACCAGGATCGCAAGGGACACGACAGCTGTGGCGCCTACAA

GAGATTCAACCGCACCGAGCACATTTCCATTGAGTGCAACAGCGACGAGAGCCACATGCCAGGATCCTTCTGCATGAAGG
TGGTGCAGCAGGGACCGAGAGGATTCATCTGGGACGGTCGTTGGCGCCAGGTGATCCGACGATGTGCATCCGTTTCCGAT
ACCGGAGTGGTTGGCGTCTGTAACTGGGGAGTCTACGAAAACGGAGTGTACTGGGAGGAGTGCTACTGCTCCAGCGACAG
CTGCAATGGTTCCAGCTTAGTTCAAATGCACGGATCCCTGCAGCTTCTGTTGGGACTCCTGTTGATTGGCGTTGCCTCCA
GAATATTTAGAAGTTAATAGGAAAACCAGGCTTATCCTCACCACAAACGAAACACGCAATAAGTGCCTTTTCGCCACTCC
ACATGATATCTAAGCTAGGATTGA***CTTTTGATATCAAAAACACAT***_TCCGTCC_ACCTTTTTCCTGCCTATAATTAAGTCGA
AAAGTTGAAAACATTTTAATCGTATAGGTACATTAGTTATCTACAATATCCAAACAAGAGGCAGGTAAATTTACAGACCA
AAACTAGCTAAAATATTTAAATGAAAAGTAACGTTAAACAATTGTAAATTTCCTATGTCGCTTGTACTTTTTATATAGTT
AACTTAATGAACATGTTTTCAAAATGATTTTACTACATTTGATTTTTAAAAAATGACCATTCCGAAAATGCTTACGTTTG
ACATATTTATTACTAGTATTTACCTGCCATATCCGAATAACCACTACCATATCGAAAAACCAGTAAATTGTACACACAGA
ATATGAAAATATATACTATATTAAGAGTATT

18. CG7713
ACGCACGCATGGCAACCCTATTGCTCAAGCCGTTGTTGTTTTTCTAATTAAGCGATTATTATTTAAAAAAAGCAAC**ATGG**
TAGTGCTGAGCTCCTGCTGGTCGCCCATAATTTGGTCGGATAATGTCCGGACGGGAAGCTATGCCGTGGCCGGCTATACG
GCTGCGCTATCCGCCGTAATGATCACGCTGATTTCGTATATGCTAGCAGGCGGGGAGTCTGCGCAGCTCTATTCCCCGCT
CTTTGAGACGGATATACGTTCGTCGATGCCCGTGGCAGGTGGCTTCTTTATCATCTACTTCCTGCTAATCATCCTGTCTT
CCTATTTGGTCTACTACGGTATTAAGATCAGCACTCGAGGATGGCTGCTACCCTGGCTGGGTCTAATTGGACTAGCCATT
CTCTTCCAGTTTAGCTGGAGCCTTTGGCTTATTGGAGGCTACTATATTTATATCTATTGCTGGCTGGTGGTCTTCTCGCA
GTACCAGATATTCCTGGAGATCCAGAATCCGAACATTGAGCTGCTCATGCCATGATGGAAGAACCTCCATACAGAAGCTC
GAATAATCTATTAGAATACTTTTTGA***CTTGACACGCAACAAGAAC***_TCCGTCC_ATGCGCATTTTTTACAATAAGAATTCCA
TACAATTCCACAGATGCCTACCAAATAGTTTTATATAAATTTATATATTTTACACACATTTATACACGATCAATTATACT
TATGTGTAATTCGAATGCCTTAGTCTTCTAAACGACAATTAAACAAATAGATTC

19. pck
CGAAGATCAACAAGCCTAAAGTAATAAATTTAATAAAAAGCCCCACCTAGTTGTTGCTGCATTTTTTGGGTGTGTGCGTT
GTATTTGTTTACTGACAATCCCCAGAAAAGAAACTCCTCTGCTCCAACTGCGATCCCTCGAGACCCAGGACAGCAGC**ATG**
CGCGAACTTAACAAGCAGCAGTCGCAGGACACCACGGACTCGGGCGTCGAAAAGGGCGACTATCCGCGGGCCACCAATGG
CGTGGTCTTCGGCGCCATAGTTACATTCGCCAGCTTTTTCGTGCTGATGATGTCCTTTTGCTCGCCGTACTGGATCGAGT
CCTACGAGGAGACGCGGGCCAGCTTCAAGAACATGGGCCTGTGGCAGTACTGCTTTAAGGACTTCGTGTACCCCAAGTAC
GCGTTCTTGAAGCAGTTCACCGGTTGCCACAACATCTTCTCGCACGTGAGTACAGCAAACTGGCCGGTTCTTATCCTTGT
CCTGATCTTCTCCCACTCGTACAGGAGTATTACGTTATCCGTGAGTATCTGCTGCCCGGCTGGCTCATGGCGGTGCAGGG
CTTCGTGACCATGTCCTTCATCATCGTGTTCCTTGTGCTGGCCCTGCTCTCGCTTACCATCATCCGACTGCCTCTTAAGG
CCGTTCTGCAGTACGAGTGGCTGCTCGTCCGCCTCTCCTACATGGGCACCGCCATCTCGTCTCTGTTCATGTTTTTGGCC
GTATGCATCTTCGGGGGCTGCGCCTACCGCCGCGACTGGATGATGTACCCCAAGTTCAACGTGCTGGGCTGGTCCTACGC
CCTGGCCGTTGTCACCTTCATGCTCCTTGGTCTAGCCGCTCTGATCCTGCAGCGCGAGGCCCGGCAAGCCTACGACGCCC
GCGGCGAGCAGAAGAACCTGGTAATGCAAATGGAGATGCAGGAGCCGGGTTACCAGCCGCCGCGCCACCACCACAGCCAG
TCGCGGAGCCTGCAGGGCTACATATAACAGCTGTCCGCCCGACTTCCATACATTCCGTTCCGATTGGATCTTAGCTTAAG
TTTTCAGACGCAATCTCGTGCCTCGAATC***TGCGCAATCGACG***_CCGTCC_**A**_**TAG**_TGCCTTTAACAATCGACCATATGTATCT
ATATACACGCCGACTCAGCCGAGATCAGCTCGTATCCGTTTGTAATTGTATTAAATAGCACGTACAGCTAAACGTAAGAT
CAGTGCGACCAAAAAGATCCACTAACTTTTTACACATCTACCATTATACTTTTTATATAGGCTCAAAAATGTTTACAGCT
TAAATTTTTAGACCATATTCCACCTTTTATAAGCAAACTGGTGTAGAGACATATCAACATTGAATTAAATGTATTATTTG

20. kune
CACACTTTAGGGCGTGTTATTTTCCAAGCCGACTTGTTAACGAAATTTTTATAGAAATAACAA**ATGGGTGCGTCCAACCG**
CATTTTACAAGTTGCCCTTTGCGCGTCCGTGTTTTCCCTAATATGTTTTGTTATCGCGTTTTCCACGCCTTATTGGCTGG
TTACAGACGGCAGATTACAAAACCCGCGATTCACTAACCTAGGCCTTTGGGAAGTTTGTTTCAACAACTTCCAGGACATA
CACCGATTTTTTGACAATTCCTTTAACGGATGCCTATGGGTTTTCGAAGAGGAATATTACATCATACACGACTTCCTCCT
GCCTGGCTTCTACATATCCGTTCAACTTTTTGCTACGCTATGCTTTGTAATGTGCCTTGTTGTTATACCACTCACTGTAG
CATTTCTTCGCACTTCTCGCGATGATGACAGGTACATGGTGCTCCTGCTGGCGATAGGATCATGCCAGGTTGTGGGCTCT
GTTTTCGGGTTTATTGCTGTGGTCATTTTTGGCGCCAAAGGAGATTCTCGGGATTGGATGCCAGGATGGCAGAACAATGA
TATGGGCTGGTCATTTGCTCTGGGCGTTGTTGGCGCTGTGCTGCTACTGCCGTCTGGCGTACTCTATCTAGTAGAAGCGC
GTCGGGAAAGGTACAAGCGCTTAAACGAAATCAGCAATCGGGAGATCAGTGAATACGGAGATGAGTATTACCAGAATCAG
GGACCCTCCGCAGTGCTTCCGACCGCTCCATCGCCATATCAATCCTATTTTGCATCTGAGCCAAGCCGACCAAGAAGACC
CCAACAAAGTAGTGCATCGAATTCCGCTGTACAAGGAGGAATTCAAACAGATATATAAATCAAAAATGAATTCGAAGGAA
TTA_TCCGTCC_***ATTTACACAATATTTAA***TAATAATTAACAAATATACAAATATATACGATGTAAGGTAGAATAGTCATTCC
ACTAAATCCACTACAAATGTTTTTAAAATGTATCGAATTCGAAGGTAATAAATATTTGTCAAATAA

21. crok
TCGAACGCTGCTCACACGGTCACACTGCTATTTACACGCCCAAAAAAATTGAATCCAATTGAAACGCGAAGAAAAACATA
AATATAACG**ATGAAAACACTGGAGAAATATATCCTATTCGCCATCGTGCTCTGCTGCCTGCTGCAATTGGGCCAGGCGAT**
CAAATGTTGGGACTGTCGCAGCGATAACGATCCCAAATGCGGGGATCCTTTTGACAACAGCACGCTGGCCATCACCGATT
GCCAACAGGCGCCGGAGCTGGAGCATCTGAAGGGCGTGCGACCGACAATGTGTCGCAAGATCCGGCAGAAGGTGCACGGT
GAATGGCGCTACTTTCGCAGCTGCGCCTACATGGGCGAGCCGGGCATCGAGGGCGACGAACGCTTCTGCCTGATGCGCAC

TGGCAGCTACAACATCTTCATGGAGTTCTGCACGTGCAACAGCAAGGACGGCTGCAACTCGGCGGGCATCCATAGGCTAG
GACTGATGGGCGTCCTAACTGGCACACTGCTCTCGGTGATTGTGGCTCATCTTCTGCGGCAATAGGTGGATCCACCTGCT
TCAGCTGTAGCCAATGCCACGCCCCATTTGCCACAAGGCTGATCAGATAGTATAAACTGTATTAGCATGCTTGCTTGTGT
GTTCAACGTAGATATAAAAACTGAAGAATTCTATAATTGTAACCGAGATATTTAGCAAATATTTTTAAAATTTGTAAATT
ACCTTAATTAGCAGTAACTTACCTGACGCGCGAACAGTCTTGTGGTGAATGAGCGATGTGTAATCAAGTGCCTTTTCTGT
AACGGACTCGTATTGTAACTTAAG***CCTGGAATACTATTTCCGTCC***AAAAAATATCTTACGCCTATGACATTCCATCTCGT
AAACTGAATCTTGTAATACTTTCTATTTTCTACATTTATTTGTAATGTTTTTGCGAAAGATCGCCTAGTTTTAGTCGAC
TTACAAGTTTTTATTGTAATGCGATGTCAAATAGAACATTTTTGAATAATTAAAAAAAAAAACGGTTATACAC

22. cold
TTGGTGTTGCAGAACGCGCGTTCTTCTAATTTGTTGCTAATGTTTTTTTGGGAGTGGTTTTGTTTGTTAATTTGACTTGT
TTATTTACTTTGAGCTTATCGGCTCCGATTGCGTTAATTACTCCAGATATACG**ATGAAATCGTGGGAGATAGCTGTGGTG**
CTAGTCGCCGCTGTTTACTTGTGTTCGCAAGTAAATTTCGTGGCCGGTCTGGAGTGCTATGTCTGCTCCAACCAGACGGG
CAACACGGAGAAGTGCCTGAACACCATCAAGACCTGCGAGCCCTTTGAAAACGTCTGCGGAACGGAGATCCGCTGGGGAT
CCCAGCCGTACTTCTCGGAGGGTGCTCTTAAGCAATACTACGTGTCCAAGAGGTGCATGACCAAGGAGCAGTGCCAGTCC
AAGAGGAAGCGCTATATGCAGCTGTACTGCACCCACATCTGGTACGAGGATTGGGCGTGCAACGAGTGCTGCAAGGGAGA
TCGGTGCAACTACTTTGTCATTAGTGGCGCTCCTTCAAGACAAGGCTATGGTGTCTGTTTAACCCTGCTCACCGCACTAT
TAGGACTTGGATCTTGGCTCATTCCACGCTCTTAGCCTCAAGAGCCTTTGCTATACCAAGCACATTTCGCTTTTTGTATT
ATTTAAACGAAGCCGTCCATAAGCTAAGTAGATTCCACACTCCATTTAAAACTACACCGTCAAAGTGCCTTGACTCAGTT
GGGAAGTAATGCCATTTACTATCCTGATGCCTTGCGAAGCGAAGTTTCTTTTCTAATACTTTACATTTTATTTGTAACAA
AATTGTTTCTCTGGATTCAAGTTTTTAGATTTTTACGTTTATGGGACAGTTTTTATACTCGTGCATCAATGACAACAA
GAGCTATTTTGATTATTGCTTTATTTCGAATTTTGCTGATGAATACGAATTGCATCCACGACATATCCTAGACAAATTAT
CTAAGAGAAAGCTCAAACATGAACATTAAGCTACATATAAGAATCAAAAGAATGCCTTAAAAATAACAATCTGTTTCATA
TCGAATCTCTCCCACAATAAAGCAACTTTGACGTCTTTTTGGCGCTTTAATAT

23. CG13088
ACAGTATTTGTTTTTGGTAATCTCGTTTTGAACAAAAATTTCGATGTGAG**ATGGAGCAAATAAATGAAGATGTTTGGATA**
GATATACTAAAGTACCTACCAATGAGGGATCAGTTGTCGCTGGTCGAAGTCAACGAGAATATATCGGCATACGTGAAGTA
TCATTGGAGTCATCTCAAAACAGTGACTCTGACCCGCGAGGATCTGGATTTTCTGGATCGGAATAACAAGCTGATGCACG
AATGTCTGGGTGGCTGGTCGGCAACGGTGGAGCGTTTGAATTTGCAAAGTGCCAGCAGTGATCTGCTGAGAAAGTGGACG
GAATACGACTTTCCCCACCTCCGATCGCTCGACTGCCACATGGACTACAATCTCGAGGAGGCGGACGAGGAAACCCTTCT
GCTAACCGAGCTGTTCCCGCTCTTGACACGGCTGTCACTCAATAGCAGCACAACAGGTCGTTACTTGTGGCACTGGAAGC
AACTAAGGGAGTTGAATCTCACCTGGTGTGAGTACCTGGACCCAGACCACTTCGAGGAGATCTTTGGCAACCTACAGCTG
ACCAAGCTGACTATGCTCTATTACGGATATAACGTGAACCTAGGCGAAAAAGTTGTGGACATAACGCGATGTACCACGCT
TGAAGAGCTGCACATCGATGACCACCATTTGCTGGGTGATTTCCTGCCCCGACTGATGAATCTGCCGCACTTCCGCCGCT
TGGCCTTTTACACGCGAGACTACTATGAGTATTTGCTAGGTTCGGTGGCCAGGCACAAGCCGCTGAAGGTTCAATCGCTT
CTCTTCAACGACTCTTTCTGGAGCAGTGAACGGGTGGCGGGAAGTATACTGCACATGACCAATCTTCGTCGGCTGGTCCT
GCAGGAGGATGACATCGATGCGCAGCAGCTGCACACCATCTGCCACAAATTGCCAAACTTGGAGGAGCTACACCTTTTGG
CGATGCGAGAAGTTCCGTCTCCCAGCCACCTGTGGAACTCTATAGGTCACTGCCATCTTCTTAGGATTCTTAACCTATCG
TCTACCAAGCTGGTGGACCTCCGAAGCTCCTGCCTGACAAAAGTTCTGCTAAGTCGCAGGATTCCACTGACCCTGCATCT
CCACAACACTGGATTGGATCCTAGTAAAGTTCTACGGGATTTTGATAGTGCCTCCTTGAAAATCTCCTTTGAGCCGATCC
ATTTGAATATTTGGAGTCCGCGATTTGTGGAAATCGAATTCAGTCCAGACTCGTAGTAGTCATTTGAAAAGACTTAAATG
A***CATTTCTACCTCAATCCGTCCGG***TATACGAATATATATGTAGATGGAGATCCAAATGATATATCCTGAGTAAAATGTTG
TACCCTTTGCAAGGGTATAGAGTCAAATAGCTTAACAAAACCACAAAATCTTTTATCTGTCTACCTACCAAAATCGTATT
AAATTCAAGAAAAT

24.Cont
GCCCACAGTCGTGGCTTGCGCATATCTAATTGCTGGTCGACTCAGCTTCTGGACTGTTTTGTTTTTATTTAATTAAACTA
TAATTTAAAAAGCTGTAAATTGCCTCTGATACTTATTTTGTCCGGATCCGCACAACTCGTGGTTTTTTTTTTTGTCAGCA
GGAACACCTCGCAGTGGTAGCGTTTTCAACCGTTCGTGTGCTGCGTCCTGGAGTCGTAATTAAGAAACTATCGAATTCCA
GAGCAAAATGTGCCTTTAGTCTGCTGCGAGACATCTAAACCAGAACCCACGCTATTAAAGCTAAGGACCAACT**ATGCTAG**
CAAAAATCGGCCTTCTGGCGAGTATTCTTGTGCTCAACTTAGTTGGCCAGATCACGCCCCAATTTTCCGAAAACCTGCCG
GATCCGGATCCGCAGTCGGGACAGCAGCCTCAGAACTATCAGCCTAGCTACAACAAGGATTACTCTCCGCGATACAACCC
CCTGTATACTGGACAGCAAAGCGCTGACCCCAACCAGTTCGACAATACGCTGGTCGACGGACAGAGCCCTAATACCTACA
AGGGGTACTATGATGGCAGGGCAGGAGGTGGGGGCCTTGGCGGCAATGTAGTCGGGCCTGGAAACAACCTCGGCGGACTC
GGCCCACAGTACGACCCCTTTAATCGGAACAGCATAGGTTCAGCAGGTGTCAGCTACCGGGACGCCTATACGGATGAAGA
TAACTTTTGCCCGGAGCACTGGGTCTCGTTCAGACAGACGTGCTATCGCTTCATCCGCTCGCCCAAGCGCAACTGGGCAG
AGGCTAAAAAGATCTGCAAGGCACACAATGCCGACCTGATAAACGTGGATAATGTAGAGAAGCACTCGTTTATACTCAAG
AATCTCATCCTGCAGAATCAACGGCAGAACCGATTCTTCATCTCCGCCCGGCAAACGGGCCCGTTGAATTGGGTCAACGA
TGACAACACCCAGTTAGTCCAGATCGAAGACTCCTTTTCCATGGACGAGCAAGTGCCCTTGGAAAACGAAGATCTGCACG
ACAATAGATTCCTTGTGCAAAACGACCTCAACAACCAAAATATCAATAATCCAAATCAGTTTTACAACTCTCTTCCGGGG
ACTGTTAATCAGCGCAATCAAAATAACCTTCGCGGCTTTATAGGTCCCAACCAGCCGTATGGAGACAACCGATATGTGCG
CGATCGCGTTGTGTACGCCTTCTCAAAGAAAAGGGATCGGTGGATGTTCATGCCAGCCTATGAAATTGAACTAAATCTCT

TTATATGCGAGTCAAAAGTACTATACAGCTCAGACAATGTCAACATAAAGTTAGACGACAAGCGTCCCTACCATTATGGA
CTCGATATAAACGACATGGAACGGATTCCACGGGGTCCTTATTTCGTAAAGCAGCCAAACGACACGACTTTCGATGTGAA
CAAGAATCGCCTGATTAATGATGTTACTCTTAGCTGTTTGGCCAACGGGTATCCAACACCTTCATACACATGGTACCGAG
AGGTATACGTAGACGACAGACTCGAGTACCAAAAGATTGATCCCCTGGCGCAGGATCGATACACCATATCAGGAGGCAAC
CTGATTATCTACGAGCCGAAGCAAGCGCTGGACCAGGGCGCGTATCATTGTGTGGCAGAAAACAAATTTGGGCGAATTCG
ATCCGAAAGCGCGCATTTAAACTTCGGTTTCATTATGGAATTCAATCTAAAACGGTCTGCGGAGACAAGCGAGATGAATT
GGGGCAAATCCATATTCTGTGACCCCCCGCAGCACTATCCCGACGTACGCTATTACTGGGCTCGAGACTATTTTCCTAAC
TTCGTGGAGGAAGATCAACGCGTATTCGTCTCCCGCGATGGTGCCCTATACTTCTCCTTCATAGAAACTGTGGACAGAGC
CAACTACTCGTGCACCGTACAGACCCTTGTGTCGGACACCGGTCGCAACGGACCCTTCTTTCCGTTGCGCGTTACCCCCA
ACAGCAACTATCAAGCACTGATATTTGCCAACACCTTCCCTAAAGTCTTTCCGGAGGCGCCAGTGGCAGGTGACGAGATT
CGGCTGGAGTGCATGGCCTTCGGTTACCCCATTCCGTCATACAATTGGACCCGCCAAGGCCTACCTCTCCAACGCAACGC
CTACACGATCAACTATGGGCGTGTCTTAATAATCCAAAATGCGACAACGAACGACAACGGCGAGTACTCCTGCACGATTA
CCAATCCCCGGAAGACACTTATGAAGAGCATATACATCAATATTCAAATGCGCCCACAATTTACCATACCTCTTAAAGAC
ATGATAAAAGACTACAACAGTGACGTGACTTTCATTTGCGAGGCATTTGCAATTCCGGACGCGAATTACACGTGGTACAA
AAATGCCGAACGACTCGACCCAGCGAATATTAATCGCGACCGATACATTATCCAAGACAATGTGTTAACCATTAAATTTC
TAGAAAAGGATAAAGACGACGCAATGTACCAATGTGGTGCCCAAAACCAACTGAAGACCTCCTTCTCTTCGGCGCAGCTC
CGAGTGCTGTCTATGAAACCGTCCTTTAAAAAGCATCCCCTTGAATCTGAAGTCTATGCTGTTTACAACGGCAATACGAC
AATTGTGTGCGACCCGGAAGCCGCTCCACGCCCTAAGTTTCAATGGAAGAAGGATGGACAGGTGATTGGCTCGGGAGGCC
ACCGGCGCATTCTGCCCAGTGGTACCCTGACAATATCACCCACGTCGCGGGACGATGAGGGCATTTACACATGTATTGCA
TCAAACCAAGCCGGCACCGACGAATCGCACGCGCGTGTTATTGTATTGCAGGAAATTCGTTTCATTGAAACGCCTCCACA
ACGAATCGTTTCTAAGGAACACGACTTAATCTTCTTGCATTGCGAGGCCGCCTTTGATGAACTCCTCGACATTGCTTATG
TTTGGAAACACAACGGCGAAGTCCTAAAAAACAATCACGACGGCACCGGACGCATTATTGTAGATTGGAACAGATTGACA
GTGCATAACACGAGCATGCGCGATGCCGGGGACTACGAATGCGTTGTCAAGTCGGCGGTAAACGAAATTAGCAGCAAGAC
CAGTGTTTCTATTGAGGGAGCTCCTGGCGCTCCTGGTGGTGTTCAAGTCATACAGATAAGTAAAACGAAGGCCATCATTG
AGTGGGTGGACGGATCGCACAACGGTCGTGCGATCCGCTACTACAACATCCTAGGCCGTACAAACTGGAATCGCACCTGG
GTTAATGTGTCGACCCATGTGCAGGCGCGAGAAGTAGATCGATACACCTCTCGCCAGCAAGCCGAGGTTGTCAATCTGAC
GCCGTGGTCGGCTTACGAATTTAGTGTTACTGCTGTAAACGATCTGGGAATCGGAACGCCCTCGGCCCCCTCGCCAATTT
ACAGCACCTATGAGGACAAACCATACATAGCCCCGAGGAACGTGGGTGGTGGAGGTGGAAAGATTGGCGACCTAACTATC
ACCTGGGATCCGCTTCTACCGCAAGAGCAGCATAGCCACGGCATACACTATAAAGTGTTTTGGAAGCTCAAGGGAGCCAT
TGAATGGGCATCTGACGAAATAAAAAAACAGGATCACATGGGCGTGGCTGTGGTTAATATTCCTCTTAATAACTACTATA
CGGAGTACGAAGTCAAAGTCCAAGCCATTAACAGTGTTGGCAAGGGACCGGAGAGTGAAATAGCTGTCATACACTCCGCT
GAGGACATGCCACAGGTGGCTCCTCAAAAGCCCATTGCGCTCGCCTACAACTCCACATGCTTTAATGTCACTTGGCAACC
TATAGACATGTCCCGTGAAAACATTCGTGGCAAACTCATTGGCCACAGGTTGAAGTATTGGAAAACGACGCACCAGGAAG
AAGACTCGGTATACTACCTGTCACGTACTACAAGGAATTGGGCGCTTATCGTAGGTCTACAACCAGATACTTACTACTTT
GTTAAAGTCATGGCTTACAATGCTGCAGGTGAAGGACCTGAAAGCGAACGCTTTGAAGAACGAACATATAGAAAGGCCCC
GCAAAAGCCGCCATCTTCGGTTCACGTGTATGGAATAAACCCGTCTACCGTGCGGGTTGTTTGGAGATATGTTTCACCTT
CGCAAGACGAGGAACCAGTAGAGGGTTATAAGGTTCGCATTTGGGAATCGGACCAGAACATGATTACGGCCAACAATACC
ATAGTGCCAATCGGACAGAAACTGGAGTCGTACATCAACAATCTGACGCCAGGGAAGAGTTACAACATGCGAGTCCTCGC
ATACAGCAATGGAGGAGACGGTCGCATGTCCAGTCCAACTTTACACTTCCAGATGGGCAAGACCACGCGCAACGGCGCAA
ATACCCGACATGGCCACAATATCAATACGGCTCTTATCTTAAGCACATTGCTACTCATTAGCACCTTTCTTTACACAAGC
CAATGAATGCAGCACAAATTAAGATCCATAACTTTAGGCAGTAAGAACATACATATACTAAGAAGTGACCTTATATTTGA
GTAAGTGAAGTTATAAAAAGAACTACATTCCATCCGTGCTCTCTTGCAAGCCCCTTAATTGATACAAATGCAAT***TCCGTC***
CATTTTATAGACTTTTATCAAAAAATAATTTCTAAAATTTTATCAACAAAATAAATATGAATACCATTATATGGTCCAGT
ATGTTGCTGAACTAAAAATAAC

25. mthl5

ATTCGCTTTTTGTCGTCGCTTCGAATCGGTTGATGTTGCTTCTTCAATTTTTATTTCGTTTTGAATACTGTTTAAAGAGG
AAGTAAAGCGCGCATTGTTTATTAACCGAATACGAGTCTGAAACCCAGACGAAAGTGCCTTTGTGGGTGTGTTGATACAA
AAAAAAGTGTCGCCTTACTTGAGTTCTGGCATGCAAGTCATGATCTTGGCTCACAGCCGGCACCTTGATGACGCATTTCT
TAGCAAGCGCAGATCCACATAAACACCAACGACAACTGCCTTTTACCTGTGTATTGTATTTGTATCTGTGTCTGTGTTAC
TAAGCAAAAG**ATGCTCGTAAAAACGCTTGGCGCGCATTTTGCAGCCGGGCAAAATGCAAAAAAATGCAGCTGCTGCGCTC**
TGCTGATTTCCCTGCTCTGCGTATTACTTCTTAGTCTAAACCCTTTACCAGTAACCAGTCATGTCACTAGCGCCGGATCA
TCAACAGCCCTATCTTCCGATCCCAACCTAGTGCTGGTCAACAAATGTTGCGAGAAGTTCGAGATACACGTGGACCATGA
GTGTCAGCAGGTCAATGAAACGGACTACTTCCAGCCGATGTTCACCAGCTATGGAGGAGAGCAAAACAGGCCCGTTAAGT
TCAAATTCGTCATCGGGATTCCAAATTGTGGATCCATGCAGATGTGGCCAATATACCACTATGCTGGCAGCTCTGATAAG
CTAGTGTTACTGGATGATGGGAGGCTAAGGCATTACACCAATGCCGAAAACGAAGCAGAGGAGCGTCATGGAATACAATC
GGATTATGAGGAGGATATTGCCGGCAGTCTGGAACCGCTATACCATGACTACGATAAGGGATTGTATTGCATTGACAAGG
CCACTTCCAGTACTGGAGAGGAAAATGTGCTATTCGCCAACATTTGCTTGGCCCGCAAGGAGATCAAGTGGAGTGACTCA
AACTTTCTGCTCCGCAAAATCTTAAACCCAATTTTCCATGGCATCTCGCTTGTTATCCTGCTGGTCATCGCCATCATCTA
TTTTATACTTCCTACTCTCAGAGATCTGGTTGGTAACATTGTGACAACGATAGCCATGTGCCTTATGGTAAGCCAGGCAG
CAGACCTTGTCAGAATTTTCACCGAGCTGACCAGCCATGTGAGCTTCATTGTGGCGGACATTATCCTGTGCTTCAGCCTC
TTGGCAGCTTTCTTCTGGCTGAACAGTTTCGGTTTTTACATTTGGAAGACCTTTCGTTCGAGGAACGTCTTCCTGCGAGT
CACGGACGGCAGGAAGTACTGCTATTATTCTGCGTATGCTTGGGGATGCACAGCAACGATGGCTGCACTGGCTGTCTTCG
CACATTTCTTCCTCGACGCCGAATCCTACAAACAGGAGCACATGGTGGGCGAGCAAGAGACGATCGGCTGGCTAGGCATA

TGCATATTCTTTGCTCCAATTGCTTGTACCATTTTGGTAAACATATTCTTCTATGTGACCACCAGGAAGTTGATTAACCG
CAGGACGGTATACGGCCGCATTGCGCACAAATTGAAAGCCAATTTTATCATGTTCTCGCTGATGCTGTTAGTAATGTCTA
TAGCCTGGCTATTCCTCATAATGTCCTGGTTGCAGATGGAAGGCCTGCTTTACGCCCACATCGTAGTTAACGCCCTGCAG
ACGCCGCTGTTGCTATACATATGCGTGCTGCGCCAGCGACATGTGACATTTCTGCTGAAAAAGACCTGCTGCTACAATGA
GCCACCGTCGGCAAACGACTGGGGCGATGAGCTGCATTATATGAACGGCAACGATTACTGAGGAG***CCACCGTCCACACTA***
ATATAAGTGTTTTCGTAGTTTAGCAATGTCCAACATTGAGATTTGTACTTAATTTTAACTAATTTATGCTGTACGAGATA
GTTATTAAATGAGAAGTCTGACACCT

26. CG8010

GCCAGTGCCAGTGCAGCCAGGGCTGCATCGCCATCAAGAACATCTGTGGCAGAACAAATAAAAAAAAGGAAAATACTCGC
TGTGTATTTTCGGAATTTAATTTGGCAACTTCCCCCCTCCGAATTCAGCGACTTGGCCTGCTCGCACCGCCTGGACACCT
GCTACTGCAGTGCTCGCAAC**ATGGACCACCACACGTACGCCAAGAGCGGATCCAAGAAGTGGTCCGCGGAAGAGAAGCGC**
AACCTGGTGGTGCAGCGGATCGCGGCGGACGAGCTCTTCTCCCGCTACTCACCGAAGCACGCGGAGCCATGGAAAAAATT
CAAGGAAATGGCCAAGATCGGCGATTTCAGCGAGATTGCGCTACGCAAGCAGTGGTTCTCCATGGTGCAGCGGTACCGCA
TCCACAAGGCCAACACACTGGGCGTCCCACTCAACAAGCAGAGCATCGAGGAGCTGAACAAGGAATGGGAGTTTTTCGGC
CTCATCCACGCCTACATGAACCAGAAGACCGCCGACCTGCACTCGTACGCGCTCAAGGAGCCGAATGTGGAGCAGCCGAA
CAATAACCTGGCCATCGCCAGTGTCTACTCCTGCGACGAGGCGCAACTCTCGCCACTCATGCTGGGCGTGCTCAATGACC
ACAACTTTGGCGAACGCTCGCCGCCCAACGGATGCGACACCGATGACATGGACGAGTCCAACACGTCGCCCTGCCACGGG
AACAGTGGCCACAGCAACAACGATGAGCTGGACAGGCTGCTGGCCGAGGCGACGGCGCGCAAGGATGCGGCAGTTGGCGA
CGGCGAGGATGTGTGCATGCTGAGCAACACCAACTGCCACGAAATGGAATGCGGCCCGGTGATGATTGGCGGCGAGGTGT
CGCTATCCAACAGCAGATACAACGACTTCGAGGAGGCGACAGTGCCCACGGCAACGGCTGTGAAGAGCGAGAAGCCGGAG
AAGCCTATCCCGAAGGCGCGTAAATTGTTAAGGAAACACCATCGCAAAGGTCTCAGCGAAAAGGAGAAGTATTACAGGCA
CCGCCGTCGCTACGAGCAGCGAATGGAGAAGCGTCTGATGGGACTGTGCACCGTGGTGGGACAACTGCTGAAGCAGTTTG
CTCCCGACATGGATGTCCAGCCACTGTTGGCCCTGGGCAGCGATGTGACGCTCAGCTCGCCAGCGCCAAGCAGCAGTGAC
GAGGAGAGGAGCGAGGTTCTGGAGGAGACGGAGGAGGATCAAGAGCTCCAGCAGGAGCAGCAGCAGATGCAGCTGGAGCT
GGAGCAACAGAAGCTGGGAGTCGAGGCCGATGCCATGACACTCAGAGTCTGAGCGAACGCATCCGAGTGCTTCTCTGGTC
GGTCGCCTGCTTGTTTCAGGGTTCAAGTGCAAGTTCCTAGCTGTGTTGCTTCACAATCAACAAACAAAATATCGTGTGGG
CTCTCAAATGACTCCCACCACTGCGCTTCGTCGTC***ATTACGCGGACAACCGTCCACAG***TGATTTTCCGCTAAAGAATAGG
GCGTGCACAGCGCCGCATAGATAGACACGTACGTTCGTTCAGTGATAACGAAAGCATAATACGTTAATCGAAAGAACGAA
TGTAAACGCATTAGATAAACCAGAAAGCAAACC

27. ND-B16.6

ACTACTTGGTATTTTACCCTGCCCCAAGTCAGGCCACACTGCCTTGGTGTTATGCAAAAAACGTCATTTTTTTGACAATT
CAACAGATTTTTTCCCTTTATTCGAGCGAGGAGTCACC**ATGGCGACGGCAGTGCCGCATTGTCCCCCGAAACAGGACCTT**
CCACCGCCGGGCGGCTACAAGAAGATACCCTTTGCCCGCGTGCCACCGAAGAGCTACTTCACAGGCTTCACCACCATCGG
CACCTATGTGGTTGTGACAGCCGTTGGCCTGGGCATCTACTACTTGACCGCCAAGAAGGTGAAGCGCGACGAGATCGAGA
TGCGTTCCGCCCAGAATGTCATCTTTCCCATTTTGGTCGCCGAGCGGGATCGCGAATTCCTGCGCCAGTTGCGACGCAAT
CGGGACGAGGAGGCCGAGCTGATGAAGAACGTGCCCGGCTGGGAGGTGGGCACCTGGTATGGTGAGCCCGTTTTCAAGAC
CCTGCCCGAGGATACCCTGGTCACGCCCATTTTCAAGGAGTTCTACGCCCACTCCGACTGGAAGTCGTACGCCAAGCGTG
CCCACTTGAAGCTCTGGTCCTAAATCGGCCTAGTTAATGGATTAGTTAATGCCTAAGCACGCACCTTTTTGTTTGTAGAT
CATCCGCCTAAGCGCCAAAAAAAGAAAGTATCGACCAATTATAATGTCAGATCAGATTGGCTGAAATACCGATTCTCTTT
GTGTGTGTTTAATCCAAATCCTGTGCTGGACCACCCGAGGTAGCCAGAGATTTTTACCAAA***CCGGGGGCGTTGCCGTCCA***
GCTGCAATATAGCCCACGGGCAGTGCTCAAAAATGGCCAAATTAGATGGATTTGGTGGCACCAACAGCGGGGAATCCCAG
TTGGCTGTGTCACCAAATTAAAATCTAATTGAAAATCTGCTGCTTAGTAAAAATAATAAATTCACAACATAAAACA

28. Socs36E

AGTCACACTGGAGAAACGGAACGAAGCGGACGCAGTGCGAACGATACAGATACTTTTCGGGGAAGCGATACTTCTTCGGA
TTCGTGCGTGGTTTCGTTGCGTCGAGTGAAATTGCACAAATTAGTGGAAGTGCACACTGTCGAATGGAAATAGTGCAGTG
CCATCGCAGATCGAAAAGTGCAATCAATTAGTGTTTGCTAGTGTGAGTGTGTATGTGCCAGCCAAACGGTTGAGATAACG
TGAAAACGGGGAAAATATCTTTCAAAGCAAAACTAAAATATGCTTAAACTACGGTTTAGCCAAATTGCCAACTAGCCAAA
AGTAACGAGCACACCGAAACCAAAAAAAAAAGCCAGCAAACCAAAATAGAGAAAACAATCGGAGGTCCACGCCAACTAGG
ACTCTGCCCCACCTCGAGCTTCCA**ATGGGTCATCACCTTAGCAAGTTCTCAGCACGCGCCAATAGCCAGGAAAAGAGAAA**
TCCTACCGCCAGCTCGAGCCCCAGGGAAGCAGCAGCACTAGTAGAGACGATCAGCCTGGACGAGGAGGAGCTGCAGGTGG
AAGAGATCCTCACAGAGGCCACTGTCTCAGCCGCCACATCGCCGGCGGAAGTGCGTCAGATCATCATCAAAATCCAGCTG
GCCAAAATGGAGAACGGCAATCCGGTGGCCGCTGGTCAGGTGGAAAGTTTCGCCGAGGTGGTGGAACAGGAGGAGGAGCA
GCAGCGGCAATGCGCAGATGGTGCCACCGACGACGACGCCGCCGCCCTGTCTGCGGATTCGCTGAATGCAAATGGGCAGA
TTGTGAATGTGACGCTGGAGGGCGGCTACGGCGAGGTGGGCGTGGTGACAAATCGAACTGCTGGCAGTTCGCCGGAAGCG
GCCAAAACCGAACGACAGCCCGTGAATAACAACGGTTTAGCGGGAAGCGTCGGCGCTGCGATAAGTACGTTACAACTGCA
CGACAACCATGGCGGCAACGCCAACTGCCCCTTGGAAGCCGACGACGATGACGACCTGGATGAGTCGCTGTTGCGTCGGC
GTCGCAGCAACAGCAACAGCCGCAGCAACATCCACCACCACCACCACCACCAGCAGCAGCAGCAGCAGCAGCACGAG
GCCGAAGTCGTCGAGTATTGCGAAGTGCTCGAGTCGCTGCCAGTCAGCAATATGTTGTCGGCGGGAACGCAACACAGCAG
CAAGCCAGGTGGGCAAAAGTCGGAGTGTGAGAGCGAGCGAACTCTCTTCTCAATTTGGAAAGTGCACAGCGAAAAATCGG
CACGATTTTTTTTTTTAACGAATCAAAACATATAATAAGTGAATAATATTTAAATCCAATTTGTCAGCAATTAAGATTCA
GTTGTGTGATTTCAAAAATTTAACCAAGTGATGAAATCCAATCAAGTAGTGACATCATGAACTTTTTAAATTTCCAGAAG

CAATAATAGCAACAGTTGCTGCCGCAGTCGCAGCAGTAAAGCACTTTCAATGGGAGCAGCAACATCCTCCGCCTCCGGAT
CCGCATCCACATCCGTGTCCACATCCACATCCTCGAAGCGACGATGCTGCAAGTGCAAGTGCCGCGACGCCATCCGAGGA
TTAAGGGATATGCTGCCGAGCAGCAGCAGTTCCGTGAGCAATAAAAAGGATGCCGCTGCGGTGGTGGCGATGGTTCCGAG
GAAATCCCGGACAGCTGACCGCCGCTCCACACCGCCCACGCTGGGCACATCGAGTGGTGGCTCACAGCGCCGGATGCAGA
GCCAAAGGATACGAAATTCCGTCGCCGTTCCGGACGCCAGCCATCACCATCATCAGCTCATCATTCGGATCACATCGCCG
GGCTTCGTGGTCGAGTCGCCCAATGGCGGCCGCGTAACTGTGGTCACCGAGCCCGCCCCCCAGTCGCCCCAGACTCCGCC
CCGGCCACGTCCCCTGGCCACGGTCGCCCCCGCCGGAGGGGCTGGCCATGCCGGAATCGGAGCCGCCCGCAACATGACCG
TGCACTCGCAAATCGACTTCATGCACTGCCTGGTTCCCGATCTCGAGAAGATCACGAACAGCAGCTTCTACTGGGGCAAG
ATGGACCGCTACGAGGCGGAGCACCTGCTCGAGGGCAAGCCGGAGGGCACGTTCCTGCTGCGCGACTCCGCCCAGGAGGA
GTTCCTCTTCTCGGTCACATTCCGGAAGTACGGCAGGTCACTGCACGCTCGCATCGAGCAGAGCGGTCACAAGTTCAGCT
TCGACTGCCACGATCCGTGTGTGTTCACGGCGCCCACGGTGACAGGACTTTTGGAGCACTACAAGGACCCCGCCTGCGTG
ATGTTCTTCGAGCCCTGCCTGACGATACCCCTGCACAGAAGGCAGACCTTCTCCCTGCAGCAGCTCTCCCGGGCGACCAT
TGTGTCCAACACCAGCTACGACGGGATCAACCAGATGGAGCTGCCCGGTCGGCTGAAGAGCTACCTTAAGGAGTATCACT
ACAAGCAGAAGCTGCGCGTCAAGCCCATCGACCAGAACACCCCGATGCCGTACTACGGCAATGTATAGCGGGAGGAGCAG
CAGGTGGGCTTCACCTACTACGAGGACGTGGAGAGCGGCAGCGAGGTGGACGAGGACGACGACGAGGGGGTCAACATACG
GGTCTCCTACGTTCAGCACGCCGAGGTGGTGGCCCAGCTGCAGCAGGTCACCACGGCGTCGGGC***CCATCGGCTCCGTCCT***
CATCCAGATCGTCCAATCTTCAGGAGCACCTCAGCAGGCTGCCCAACCGCATGGCAGCCGCCTTTGGTCGGATGTCGGCC
AATCTGAATTGCTACCAGAAGACTGTCGCCACGCCGGAGCGTCACTCCAACTCCTCCTGAGGATCAGGACGTGGACGGAG
GGGCAAACAGAACCCAGAAACCAAAACTCAAACTGAGACAATCCCAGTGCAGCGTGTGCCGCCACCATAAGTTAAAAGAT
CAAACGCGAAGCCACATAACAAAATCGCTAGCTCGCTACTACAAGACTGTCATGGATCAAAAACCAAATGTTCACCATAT
ATATCTCACCTATTGGAATGCAGCTCGGACGGATTTTTGAGTTGTGGCACTCTTGAAAGCGAGCTGGTAGAATGTAGAAT
CGAAATCGCAGTTAGCTAATTGTACTATAAACTAAGTATTATTATCTAAAGCAACCCTCTCTCCTACACACATAAGTATT
GCAAAAAGAAGGGCCAATCGAGCTTTTCGTAATCGCTGGTATTCTTGTAATATTTCATTGTATTTATTTGAAGTATGGAA
TCTAAATGATGTGTTACTGTTACGTGTAAATACGAGATACGAATGCAGCTTAGTTAGTGTAAAACTCTCTCATTAGCCAA
ATTTCCAAATGTAATTTTCGCCTATTTATGAATATACTTTATTTATTTATTCGAAGTGATTTCGTTCGCAAGCGAGTGCT
TTGTTTAAAAAGAAAAAACCCGTCAGTTGTACACGTGTAGAGCATTTGTAAATACTACCGATTTTCGCATTAAGCTGTAG
CTATATTAATATAATGAATTATAAATCATGTATTCCTACCTAGGTGGTTGCTGTTATTCGTAAACATTCAATAAGCAGTT
GTTTCCAATACCTATTTAATGTATTTTACATATCTCACATGTTGATGAGCATGCGTATCAATATTCAAAAACAAAATCGA
AAAAAGAGTCAAACAAATAAAGTTGATCAAGATATTAG

29. Atet

TCAGTTCGCATTCAGTTCTTCGTCGTTCGCGCTGCCTGAATGTTGTTGCCTTCTGCGGTCGAGCGGGATTTTCGTTTTCG
GCCAGGAATCGAGTCCAATCGCAACGGATCGCATCGCGGTGAATAAAAACACGTTTCGCCTAGGCAAAAGATACACTTTT
GCGTATTCCTGGAGTGCCTCGCTTACCATCTGGTAATGGTATTTCGATTCTGTTCCTCTCACTCCCGTCGAATCGTCGAA
TTGTGGCGCATTTAAATAGCTGGCGTTGTTTGTTTGTTTGTTTGCTGGCTGCCGCGACCATAACACAGAATACAACAAAG
AAGCCAAAGGCACGAGCACGAGTATCTCGCTGTCTCAGTATCTTCTGAAAGTATCTAATGTGCGCGGGAGCGGTACAGTG
AATACGGTCTCTTCTGGCAGCGGCCGAAAAAAAAAAAATATATAATGCTAATAAACCAAAAACACGTTTTATGCCATTTGT
GTTTTGCTCTTCTCGGAAATAGGCCCCATCTTAATAGAAATCCAAAGAGCTGAAAGCTGAGTGGTACAATCAACATATAA
CGTTTACTTCCAATGCAGATGCCAATCGCCAGCGTATTCAATTAAATAACAGCATTCAAGATCCGCAAATACGTATCACA
AAAAAATATAAAGCATTAATCAATTGAGCCAAATGCTGATGCCATATGCCAAAAGCGATTGCAACAAAAACTAAACGCAT
TCTCAACTAGAACTACACAGAAACTAGCGTC**ATGAATGAGCTGCAGGCCTCCAGTGTCCCGCTGGTGGACAAGTGCTCCT**
CCCACGCGGTCATCCTGCCGGCGGCCAAGAGTCCCAACAGAGTCTCCGATTCGGCAGCCACTACGGCACCTAATGCCGTT
CTCTACACAAGCGGGAACATGTCCCTATCAACGCTCTCCACGGGCAGCGGCAATCCAAATCCAAATCCGAATGCGAGTAC
CAATTCCAGACAGGAGCCAGTGCCACTGCTCAGCCAACTGAAGCTGACCAACGATCTGAAGCAGAACAGTCACGGGTCCC
AGAACAACCTGTGCAATGGCGGCTTGGGCAGTAACAACCATGCCTTGGCCCCCAAGGTGGCCAACAACAGCGGTGGTAGT
CCGAATGGCCAGAAGAAGGGTACCATCGCCTTGTCCCATCTGCCCCAACGGCCGCCGGTGGACATCGAGTTCTGTGATAT
ATCCTACTCAGTTACCGATAGTCATCGGCGCGGATTCAAGACCATCCTGAAGAGCGTCTCGGGCAAGTTCCGGAATGGAG
AGATCACAGCTATCATGGGACCCTCAGGAGCCGGAAAGAGTACGCTGATGAACATCCTGGCGGGCTACAAAACTGCCCAA
CTCAGCGGCTCCGTACTAATCAACAGCAAGGAGCGAAATCTGCGGCGCTTCCGCAAGCTCTCCTGCTACATCATGCAGGA
CGATGTTCTGATCGCCAATCTGACTGTTCGCGAGGCCATGATGGTGGCCGCCAACCTCAAGCTGGGCAAGAATATGATCA
GCTACGCCAAGGTGGTGGTGGTCGAGGAGATCCTCGAGACAATTGGCCTCAAGGAGTCGGTGAACACGCTGACCTGCAAT
CTGTCGGGTGGCCAAAGGAAGCGACTGTCCATTGCCCTCGAATTGGTCAACAATCCACCAGTGATGTTCTTCGACGAACC
CACCTCCGGACTGGACAGCTCCACCTGCTTCCAGCTGATATCGCTGCTAAGATCGTTGGCCAGAGGAGGCCGCACCATAG
TGTGTACAATCCATCAGCCGTCGGCGCGTCTCTTCGAGAAGTTCGATCATCTGTATCTGCTGGCCCAGGGCCAGTGCGTT
TACGAGGGCAGAGTGAAGGGATTGGTGCCGTACCTATCATCACTGGGCTACGAATGCCCCTCATATCACAATCCTGCTGA
CTATGTCCTGGAGGTGGCGTCCGGGGAGTACGGCGATGCTGTACCCAAGCTGGTGGATGCGGTGAAGAGTGGGGCTTGCA
AGAAGTACGCGCACAAGGATTATGTACTGACGTTGGCCCAAAAGGGCTGCAACAATGACATTATCAAGGGCAGTGGCAGC
GGAGCGGAGAATGCAATGGCCATTTTGACCCTGGAGGATGAGAAACCCCCGCTGGAGGACAGACAGCTGGAGCCCTCTAT
TCCCGTCGATGATCCTGCGGAGCTAAAACCACCGAAGCTGGAGACGCAGCAGTCCCAGAACTCCGATTGCAGCGTGGTCA
ATATGCCGACTAATGCCGTGGACGACAGTTGCAGCTTTAGCTCCTCGAAGGGCACACAAAATGCTGTAGGTGGTTCGGGA
TCGGGAGGACCCAGTGCCGTTGTTGGCTGCATGACCTCGCTGCTGGATTCGCACGAGAGCGTGGTCACATTGCCCAACAA
GACGGGATTCCCCACCAGCGGCTGGACACAATTCTGGATCCTGCTCAAACGCTCATTCCGCACCATCCTGCGCGATAAAA
TGCTGACCCACATGCGACTCTTCTCGCACGTGATCGTTGGAGCCATCATTGGGATGATCTACTACGATGTGGGCAACGAG
GCCAGCAAGATCATGAGCAATGCGGGATGCATCTTCTTCGTTTCCCTGTTCACCACCTTCACGGCCATGATGCCCACCAT

TCTGACCTTTCCCACCGAAATGTCAGTGTTCGTGAGGGAGCATCTCAACTACTGGTACTCCTTGAAGGCCTTCTACTTTG
CCAAGACCATAGCGGACATGCCATTTCAGATTGTCTTCTCCAGCGTCTATGTCCTGGTGGTGTACTACCTAACCTCACAG
CCAATGGAATTGGAGCGGGTCTCGATGTTCGTGCTGATCTGTGTGCTAAACTCACTGGTGGCACAATCGCTGGGACTGCT
GATCGGGGCGGGGATGAACATCGAGACGGGCGTATTCCTCGGCCCCGTGACGACCATACCCACGATATTGTTCTCCGGAT
TCTTTGTGAACTTTGACACCATTCCGGGCTACCTGCAGTGGGTGACCTACGTCAGCTATGTGCGCTATGGCTTCGAAGGT
GCCATGGTAGCCATCTATGGAATGGATAGGGCCAAGATGCAGTGCAATCAAATGTACTGCCACTATCGAGTGCCCAAGAA
GTTTCTGGAAGAGATGTCCATGGATAACGCTCTGTTTTGGGTGGACGCCGTCGCCCTGATCGGAATCTTCTTTGCCCTGC
GCATCATCGCCTACTTTGTGCTGCGATGGAAACTGCACATGATTCGCTAAGCGGACAGCTTGTGGGATTTGTTCATAGTC
TAGGAATATCATTATTACCATACGTTAGAGATCACCTGTACATAGAAAGGGGTTTGGGGCCTAGTGCGGGGGAGGTTGGG
ATCCCAGGAAAGCTGGATTGTAGCTAGTTGTAGCTTTATCGCATTCTAAAACTTCTCCCGTCGCGCTTTGTACGTTTATA
CAGACAGACAGACATACACACACGCATCGCATCGCATCGCATAGGTATCTGGAAGACGATTCTAAATATATACCCATGGA
TATATATACACCCGTACAGTAGCTGTTCTGCATACCACAAAAAAAAATGGAATTTACATAGAACTATATATAGAGTTAAT
ACTAGGAGTACCCACGTAGAGCCCCACTTCAACACACACTTCAATTGAGGTACATATAATTCTTGTGACCATGCAGAAAA
GAGTCTGAACTTAAGTAGGTTGAAAACATAACAAATTGTAGACAAGATCCTTTAGAACGGAAACCGAATCGGTGCAGAGT
TTAGGACACGACTTTCTC***CCAACGTTCTTCACTCCGTCCCT***AAGTTGTACTCTCAAAAACACACTATAATCTTCTTTTGG
CAATCGATTTCGAGCCCAGAAAGGCAGCAGCGTGCAATCAAACGGAAGTTAGGGCGAGCGTGTAAAGCGGAAACGGAAAC
AGAAATCATTCCCTTGTGATAGCAAGCGGTGCAGTCCGGGCTGGAGAATCCACATACCGGACGGGAAAGGTAGAACCAAC
AAATCGGAGAAGGGAACCTGGCCAACTGGTGATATAAAGCAATTACTATTTTTACGATGGGATGCAATCGGATTTGTGTT
AGCTGCGCGTATTTGTGATTATTACATTTTAATTTAGTACATGTTTAGTTTAATTTTATACGTCTACATTTGTCATGCCA
AAATAAATGCAATTTTCATTTAAACAGCACGCTCTCCTTCCTTCGGCAGCCACACCCAAAGTAAATTATTTAATGATAGC
CTTGGATGTAAAACACATTGGATTGATACACAAACCAAGTTTAACTACAGAAATCGGTGGCAACATATTCAGGCTGCTTT
CAAAAGGACAGTACAGCATTGTAATTATTTATTAAGCAATCTGTTGTGAAAACCCCCCGGGAAATATTCGTTTAAGTATG
GTGTGTTGTATATTTCGGTAGTTTGTAGCCCATGTTACACTGTACATTAACGTTAAAGAATGGCCATTACTGTACACAAA
ACCCATATTAATTACTGTCAAAATCTGGTAGAGTATTTTTAATAAAGAACCCAAAACATCATCGAT

30. Itgbn

ATATTAGTTACGCTGGACTTCTAAGGCCGAACGGTCGCTAGCTCCCTTAGCAAAAAAAATATATGTGATCGTTGGTAGTG
AAAATTGCACGCATTTTGAAA**ATGACGTCACTGGGCGGAAGGGCGTTCTTGTGGATATATTTGGTTTTTCTAATAGCTGA**
GATCTCTCATTCGGATGCAGATTCAATTGATGATCAGTGCAGGCACGCAGACAGCTGCGAAAGATGTTTATCCGCTCATC
TGGAATGCGCTTGGTGCACGGACAAGGAATACCAGGTGGGATATCGTTGTCTCTCTCGCCGGCAACTACTTAACTACAAC
TGCAGTGAAACGGATATATACGAGAACCAGCCGGTCCTAGATGTCCTTCAGGATAAGCCCCTAAAGGACTACGAAACCAG
TGATCAGGCTGTCCAAGTCACACCCCAGCGGGCATACCTTAAGTTGGTCAAAGGAAACACGCAAAGAATGAAGCTGAGCT
ATAGAACTGCACGCAACAATCCACTCGATTTGTACGTATTGATGGATCTAACCTGGACAATGAGGGATGACAAGAAAACC
CTGGAGGAGTTGGGAGCCCAACTCAGTCAGACTCTTAAAAATCTAACAGGAAACTACAGATTGGGATTTGGTTCTTTCGC
GGACAAGCCAACTCTACCCATGATTCTGCCACAGCATAGGGAAAACCCCTGTGCTGCGGAGCGGGCCACGTGTGAACCAA
CCTATGGATATAGACATCAACTTTCGCTAACCGATGATATACCAGCGTTCACCTCAGCCGTGGCGAACAGCAAAATCACT
GGAAACTTGGACAATCTCGAAGGTGGCTTGGATGCTCTAATGCAGGTTATAGTGTGCACCAAGGAAATCGGCTGGAAGGA
ACAAGCTCGAAAAGTGGTGATCTTAGTGACCGATGGCTTTATGCACTTAGCGGGGGACGGTCTTCTAGCAGGTATTATCC
AGCGCAATGATAAGCAGTGTCACCTGAATAAAGCAGGTGAGTACACGGGTTCATTGAACTACGACTACCCCTCGCTGGAA
GAAATCTATCGGGAGTTGCTTCGTCGCAAGATTAATGTAATCTTTGCTGTCACCGAGGAGGTTGTAAGTAGCTATTGGGA
ACTCAGTGCCCTAATGAAGGAAATTAGTTACGTGGACATCCTAAGTGCTGATTCCTCTAATATATTGGAACTAATCAAAA
AGAGCTATGAGAGTTTGATTAAACGCACTCAGTTTGCCGATAACAGCCCGGATTTTATTGACATGGCTTACTATACGGAT
TGCGGTGGTCAGTTTCCCAGCTTACAAAAGCGGAATTACTGCAATAACGTCACTCTGGGCAAGCAGATTGATTTCTACGT
GGACGTCACTCTCAAGAAATATCCCGATAATCAGGTTTATACACACAAAATCCGGGTGGAGGAAACCTCCTTGAGCGAGT
TCATGGATTTGGATGTTGAGCTTCAGCGGCCGTGTCCCTGTCAGGAAACACCAGATCCGGAAAACGAGGAAGGACGTTTC
CTGTGCGATTATAAGGGCTATTTGTACTGCGGAATGTGCGAATGCGATGAGGGGTGGACTGGGACCTATTGCAACTGTCC
CACAGATGCCACAAATGTCACCAGCAATGAAGCTCTGCTCCAGAAATGCCGTCAACCGTTTTCGGATAAATCCACCTCCG
AATTGGTTTGCTCCAATCACGGGGACTGTGACTGCGGAACTTGCTTGTGTGATCCTGGATATACGGGTCCTTTTTGCGAG
TGTCGCGAATGCCTGGACTGCGATGAAAAGCTGGCTGATTGTTTCTGCGGCCAATGCGTTTGCAAATACGGATGGTCGGG
TAGCAAATGCAACTGCGATGGGGATACCGATGCCTGTGTGGGACCCACGGGCGAAATATGCTCGGAAAGAGGCACCTGCC
AGTGCGAGGAGTGCCAGTGTGAGGAGCCGTATTTGGGTAAATTCTGTGAAATCGATCCAGAGAAGGACAACAAACTTTGT
TTGTTCTACGAGCCCTGTGTCACATGTCTGATCGAGCAAAAACAGGGAATGGGCGTCTGCGAAAACTTGACTGAAATATG
CAGCAGTTTGGATAGACAAGAAACCTATCCATACAACTTTGTCCACGAGCTGGATCCTGAGCAGGATCAATGCTTGGTAC
GACTGGTGAACAAACATGGCATCCAATGCGACAGCTTCTTCGTCTATCAGGTCATCGACCACTCAAACTTCTTGACCATC
CAGGCAGTTGATTGCGAGCCCCCGGATTACGTGGCTCTGGTGGGATATATATCGGCATTTACGCTGCTCATTGGACTTTT
GATCATTTTCATAATTCTGTGGTACATTCGAGCTAAGGATGCCAGGGAATATGCCAAGTTTGAGGAAGATCAGAAGAATA
GTGTCCGACAGGAGAATCCAATTTACAGGGATCCAGTGGGTAGATATGAAGTGCCCAAGGCGCTAAGTGTTAAGTATGAT
GAGAATCCTTTCGCAAGTTAACATGAAAAATGTTTTTTTTTTAAGATATTAGATTGTACTTTGACTTAAAATTTTCTTCA
AAATTTGTGTATACCCGTTACTCGTAGAGTGAAATTATACACTAGATTCGCGTTGAAAAGTATGTAACCGGTAGAAGTAA
GCGTTTCCGTTCATATTTTAGTTTTAGTTTATTTTCTTGATCACAATACATAGCCGAATTGATCTGGCCTTGTCTGT***CCG***
TCCGTCCGTTTGTTTTTTTTTTTTTCTAATTTTGTTCCCATTTTCTGTCGGTATTCCAAAAAATCTCTCCTTCGCATTTT
TACTTGATGAGTAATGGATAGCTGATATTCGGTTAACTCGACTATAGCATTCTCTCTTGTTTTTAAGCTATTGTAACTCA
AGAATTACGAGTTCACGAAGACATTAATACAAATATAAATTTTAGGTGTTTATTAAAAAGTATTAGCCCCGTTCAGAAAT
AACATTCCAATTCCAAGACTGGTCACAACAATAGCACTATTAAGATCAATAATTTTATATAATATATTTAGAACATAAAT

TTGTTTTTTATACATAGAAATTATATGAATTAGTTCAAAAACTCGTGTATATCTACGCTAAATGAGCTAAAATTTATTAC
AGCTAAAAAACGAGTAAACGTATAACAAAGTTAGCTTTCAGTTTTCGTATTGGACAGCTCCTTTCAGCGATTGTATTCAT
ATAAGTACAAAAATAATGGCTTAAGACATCGTTTACATTAATCCGCTTGGGGAGTATAGATGACTACTGGCAAATCGGAA
ATGCAAAACATTACCTTAACTTAAGAGGCATTACAAAAATACAATTCGATTAATTAAGGC

31. bves

ATCGGTAATAGGGGTGACAACTCTGACCGCTGGCGGGTTTTATTGTTTCCCTTTTAACCACTTCTTTTGGCTGCTATTTG
CGGTTTATTCGGCAGTTAATTCTCGATTTATGATTAGTTACTATTAGTGAGTCATCAAAAGCACGGCTGGTATTCGAATC
GGCAATTCATAACCGGCTGTTCCTGGGCTTATATCCCAGCTGTTTTCGACTCTCCGTGGGAGCGAGCACGAGTAGTGAGC
AGAGAGCGAAAAACACATTTCAACCACCACACAGTGGGGTACGATTATTATTTTTTAGAAAGTTACCAAAAGTTGTCTAA
ATACGTTTAGACCAAAGATGTCAACTACGCCAAAGCAACCCAGCTGCCTAAGCGATGCAAAATGTGACAGGCGGCAGCAG
CAGTAGCCTCATCAAAGGTTAGCTCACCAATAGCCCCAGCTAAATATTAAACAAATACGGAAATAAGAGTCTAAGGCCAT
TAACTAAACGGCTGACTAAGCTGTAAAATGCAAGTAATTCAAATTTCAACTAGCTAGGATATGAACCGCAAGCAAAAAA
TACATTCAAGACACCAACCGCAAAGCACAAACATTTTGAAAAGGGCAGCAGCAATAACAAAACAAACCACCACCAACAAA
GGTTGAGGAAAACCGCGCGTAAGCAACAACAAACGAATTAGGCGAAGGAGAGAAAGGTGGAAGATAGAAACAAAAGGCAG
ATTTTTGGCATTCGCCTAGCGTGGACAACAACCAACCAGTACATCAACCAATCAATTTGGTAATTAGGCAAAATACGTGC
TGCAAAAAATAGCTCAACGATCAGGCTGAGAGGAGAGTGACAGACTGAAACGAAGGCAGTTCGCCTGGATCAACGGA**ATG**
CCCAGCACGGCGGGCAGTGCAGCTGGCGTTGGTATGGGCGCCCTGATCAACAGTGCCGGCAGCAGCGCCAGTAGTGTCAT
GGGCATTGGCATGGGCAGAGCAGCTGCTACGGGAGCAGGAGCACCAGGCAGTTCGGGATCTGGAGCAGATGCCTCCGCCG
CCGGCACTTTAATCGCCCAGAGCACGGCGGGAACGAGCGCCGCCAGCAGTGGAACGATCACCTGGGACAACAATGGAACC
CTGCGATCGATCAATCCCGGCGATTGGTCCATCGAACAGTGCCTCGGGCCACATCACCTGTACTTTCAGCTCGGCTGGGC
CTTTCTCTTCCTAGCCTTTTTGGCTCCACACGGTCCCTACGGCGCCCTGTGGATGCGTGCCATGCTGCTCATTGGCTGCC
TGATGATGGGCATGCATGGCTACTTGGTGGCCTTTGCACCGGACGTCATCCTGTGGTCGGGCATGGGACTTTTTGTCAAC
TTCATCTATCTCGTCGTGGTGCTATGCCGGCTGAGGCCTGTGCGATTCGAGCAGGAAATCGAAGCGGTCTACCTGGCACT
TTTCCAGCCGCTGCACGTGACGCGCCACCAGTTCAAGAAGGTGCTCAACTGCATGAAGGTGATACGTGCCCTGAAGTACC
AGGAGGTCTACGCCCAGGAGAAGGTCACCAAGGTCGACAGCCTGTCGCTGGTGCTGAGCGGCAAACTGGTGGTGTCGCAG
CATCAGCGCGCCCTGCACATTGTGTTTCCCCATCAGTTCCTAGACTCGCCAGAATGGTTTGGCGTCTCGACCGATGACTA
CTTTCAGGTCTCCATCATGGCCATGGAGGAGTCGCGGGTGCTGATCTGGCATCGCGACAAGCTCAAATTGTCAATTATGG
CCGAGCCCTTCTTGCAGACCGTCTTCGATCACATTCTGGGCCGGGATGTGGTCAAGAAGCTGATGCAGGTCACCCAGGTG
AGCGAGTCGATAGCCAGCAATGGCTTCCTGCCCTCTGGTGGATATGCTGAGGATGCGGAGGACAAGCCCATGTTGATACT
AAAGAAGAGTGTGGATGTGGGACACGGACTGACGGCCCTGATCAACCGGCAGCTACAGGCGCATTAAACCATCTAGATTT
CTGTCAAATTGTTTAACCATTTTTCAAATGGCTTAACAGCCGTAAATTAATATTACTTCAGAATTGAACAAATTTCATAT
GTAGCGTTCTCTAACATGAACTTACTTAAAACAAATGATCTTTAAGAACAAACATACCAATTTTGTATAACCTTTTTAAC
TGATTATTGATGCACAGAACATATGTACGTTGGTATTCTTTAAAGTCATAATCATCAATTTACTGACTAAACCGCTTAAT
TTCCTTGTTCCTTAAATATTAAATATAAAATACAGAAATGGGCTATTAAAATAGTGCTGTTACGATTTTCTGTATCTGTA
CTCTCAGTATTATTTTAGTGACCGCTGCTGGGGTTAGCTCTTCCCCACGCTTTCATTAACATTCTTGATGTTTGGGGCTC
TTGATTTCTCCACTTCGACCTTGTGTGTCTCCCTCCCTCTTGTCGCTATCACACTCTGTCCCACTATCTATCTCTGTTTG
CACGCTTTCTGCATGGCGAAACAACATAACTTTACTTAAATGAAGCCCGTACGAGCATGTTCCTTTACTCGGTCGCACGT
ACAAACAACAACAACAACAACAACAACTACTGCTACTGCAACAACAACTAGAACAACAACGCGTACAAGAAGCAACAACC
AGCGCCAACAACATCGAGCAAAGTGCAATCTGAATTTATGCAACCGCCCGCTGCTGCTCAACTTGCCACGAACAGAAATC
AGAGCA***CCCATCCGTCCCTTCACCTTTGA***CCTGCACAAGAACTACCAGAAAAACAACCCCACCCACACATGCAAACAGCC
ATCAGCCATCAGTCATCAGTCAGTCAGTCATCAATCATGCGCAGCCACACCAACAGCAACATCGATCACGATCATCAGAA
AAGAGCGAAAAGAGCTGTCGAAGATATCTGGAAGCTATCCATCTAAATCCGCATCCTAAGTTCCATATATAATAATCTCA
TCTTTAGCATAGCAACTAGCACTAGCTAAACAAGCATCTAACACATCTAATCGTTCTAATAGCAAACTTTGTATTGCCCC
CACAACTGCAAACAATTAGCCAGAGACTTGAGTTTTTGTGTAAAGTAATTGACTTTGGGAAACATAAACTTTTCGTCTCA
GCGGAAAAGTGCTAAAACACCGTGTTACCATTTAGAAGAAACTTGGAGAAGTTACTAAAGAAGTTTACAGGCGTGAAAAA
AAAAAATGATTCGCCATTTCCCCTGATAACCCTTTTTAGGAGGTTTATTCAAAAAACATAAGTAGTCTTTTATCAAACGT
ACACTGACTTAAATCTAGGAAACTGGGAGAACTGCCTAAAATGATTTGTAGTTTCACCTGACAACTCTATTTAGCAGTGT
AGAGAAAAACGTAACATCGTAGACCGTTATTAAAAAAAAAAAACAAGAAAAAAAACTATTCAATTAAACCCGTAGTAATA
TACTTGTATTCCATGAAGATAAAGAAACCAATTATTTCACAAAGAAAACACAAAAACAAGTAGGAAACTTACTTACATTG
CAACACTGTTGAATTCAATATGCTCCCTGAATCCTTCGCAAAAACTATCGTTAACTGCAGTCAAACCAGTTGTAAAAATT
GTAATTTATTTTGACTTATCATGGTTGTATGTATGTTGAGTTGTAGTCAGACAAGACACTTGATTGCACATGAATTAGTT
TCAGTTTGAGTGGGGTTTTCAATTGAACACGATCAGAAAGCCAACCAAGGTTTAAATTTGAAATTAATTGATCGTGGTTT
TGAGTTTGAATTGGAATTAGTAATATTTGATATATCACGCAGTATACAAACATTTTATATTAGTGGAAGTTTTTGAGTCG
ATCTTCTACAATATAAACGATTATTACACTCGTGTAATAAAGGAAACCTTTGTAGACGCAGACTATTTTCCAAGAACCTA
TTATAAGTATAATGACTAGCATTGAAACTGGTTTGATTACAGCTCTTCAGCTTCAATTACCGATTCTAGATCTCAAGATC
GATCAGTCTAGTGACAGGTGATAGGTAATTAATGAACTTTTCCGCTGGGCCAGGAGTTTTCAACGCTTTATTTGATTAAA
TTCTTATTTCTTACCGCTTGGCATTTACTGGAAATTTACTGTGCTGGCACATCAATCAAGTCTCTAAATCTTCCATTAAC
ACATTATTATTTTATTTGTATTTCTATATGCATACGTATTTTGCGTTGATTCGTTGATTGATTTGCATACAAATTGAGCT
TGAAGAAAGCGTGAAAACTAATTAGAATTAATATCGAATAATATTTGATAATGTTAAGCAATACATAGCCTAAGTTGTTG
TACATACGCTATATATATATATATTTGCTGTACATAATCCCGAGTGCCCTCAAATCCGAGAACCACACCCCCGTCCCCTAAT
CTCCACTAATACTCCCCCAAGAAAGTGTAATAAACGTAGTGGAATCAATGTGAATGTAAACAAGCCAAAAACCGAAAGCT
AATAAAACAAAAAAAAAACACAC

32. Cad96Cb

CGAGGAGCTCGTTCAGCGCTGCGGAGTACTAACTGAAAGCGTTTCGGCCAACATTGTTCTTTGTCCGTTGCAATCGCGGT
CAAAACAATGAAATAACTAACATTTAGTGCACCTGGAAGATAAAGCACGAAAGGATTAAGGCTAAACAAAGGAAAAGTTG
CACATAAATTCAATTCGTTTATGGCACATCACAGATAGACGCAAAGTTATTGCTTAAATAAAAAACACGTACCGGTTAAA
CAAGGGCATTACAAATGGAGTATGGAAAGTGCTAAAGAACATCAAGTGAAGTGACTGAGCTAAATCTTATTTTCATGATT
TGTGTGGAAGTGTTATGTGGCTCTGAACTGAGGAAGCTGAGGAACTATCAACTAACCTAATTGCTAACATAATATCGTTG
TACAAA**ATGCGGATAAATCACCAATGGATATACTATGCGATCTTGTCTCTCTGCTGGTTTAATAAAGCTACTCTGGGCGC**
CATTCTGGACTCACGTTGTTACCTGGAGGGCGGAGGTTCCGCGGAAAGTTTTCTTGCCACCGAGGATTTGGCGGTGGGCT
CCATAATCGGCAAGCTGCGGATCAATGGGGATGCCAATGCCGAAACAGGCGACATCAATCTTTCATTGAGGGAAAAGAAT
GCACCAGTTGAGATTCATCCCGGCACCAAGGAGCTAGCCCTATCGGTGGAACTGGACAAGGAGGGTGTGAGAGGACCATC
GTCCATTTACGTGAATGTGATCTGCATACGACGGCATTCAACGGATCCGAGCTTCGTCATTCCCGTTAACGTGCGAGTAA
CTGATGTGAATGACAATGCCCCCCAGTGGATCGGAACGCCCTACACTCTAACTCTTTCGGAGGTGACCGTTCCGGGCACT
AGAATCCTGCAGGGCGCCCGTGCCGAGGATGCCGACCAACCGGGTCCCTTCTCTACGGTGGAGTACCAGGTGCTGCCAGG
ACCGTACTCGGAGTACGTGCAGTTTCTCAATCCTCTGGAAGGCACGCTGGTGCTAAAGAAAGCCCTCGACTACGAACAGC
TTCAGAATTTCACGGTGAAGCTGAGGGCGCAGGATCAGGGAACACCGCCACGTCACTCGGACACGTTGCTGCGGGTGGTC
ATCACGGATGCGGATGATCAGAACCCAAAGTTCCAGCGAGAGTCCTACAGCGCTGAGATACCAGCGGATGGTAGGCCAGG
AGAGCTGCGCATGCGGCCGGAGCCCCTGAAGGCGGTGGATCAGGACGAGGGTATTTGTGCGCCCATTCAGTACACGATAG
TCCAGTCGCAGGATGCCAAGTACTTCCGCATCCATCCGCACAATGGAGCTATTAGCCTGCTGACACCCATCGGATATGCG
GATGTGGCCAATGGGGCAACGTTGGTGGTGAAGGCCACGCAGATCGACAATCCAGATCGTTATGCCTTGACCACTGTCCA
GTTGACGCGACCTGGAAGTCACAGTGATTTGAGCACTCTGGCCTTCGTCCAGAAGCGATTCGTGATGCGCATTCGCGAGG
ATACGGCAGTTGGGAATCGGATTCTAGCACTGCCCACCAATAAGCCCGGCAAACATCTCAAGTACGTCATTCCCGATCCC
GTAAATTCGCAGTTCTTTAGCGTAGGATCGCTGGGAGAACTTGTGCTAGCCAAGCCACTCGACTACGAGAAGATGACCAA
GCACGAGTTCCAGGTCCTAGCCACCGATGGCATGACCAACAGCACCGCGGAACTTACACTGGAGGTGATTGACGTAAATG
ACTGGGAACCTCGGTTCCGGGAAACGCACTACGAGTTCATGGTGCCCAAAAGCTCCCTGCAATCTCGAGCGGATTCCTTT
GAGGGCGTGCTCATCGGCAAGGTTGAGGCTGCTGATGGGGATCGTAATGACAAACTGGAACTCTCGCTGCGTGGTCAGCA
CGCTGGACTGTTTGAAATTGATTCCACTGGAAATATCTACATGCGTCCGGAGCAGCTACAGAGTCTCAACGAGTCCACGG
TCCATCTGATAGCCATCGCCACGGACACGGGCGTTCCACCCAGGAGCACTTCCGTTCCGGTCAGTGTCACCATGGAGGGT
CTGACGCTGGCCCGGTCCGGATGGAACAGCAACATGTTGGGCATGTTCGGCATGATAATGGGCCTCTTTCTGATGATCAT
AGTGGCGTTAAGCTGCTACATAGTCCGATCGAAGAAGCAAGGCAAGAGTGCATCCAGCGGATTGGGACTCGGTCGGAATC
GGGTGCACAGCCAGGCGCACAGCAGCGTGTCGTCTGCCAATCTCGTTACCCACGAAAAGCTAGCGGGGAATGGCAACGGC
AACGGAGCCAGCGTAACGAGCGGCGGAGTCTCCGTGCTGCACATGAAGCACGGCGGTAACATTACCATGGCCAATCCGAT
GAACAATGGCTTGCATCACGTGGCCAGTGGAGCCAGTAGTAGCATGGCCCTCGGTAGTTCCGCCGCCTTGCTGGAGCGGG
AACGAGAGAGGGAGCGGGACCGGGAGCGCCAGCGGGAGAGCTATGCGGCTACAGTGCGCAGCATAGTTTCGCGCGCCTCC
GCCAATGGCCAGCTCTTCGAGGAGGATGAGATCGAGCACGACTCACTGCAGACGGAGAACAACAATCGGAAAGTGGCAGC
CACGGGAGTGGCAAGCGGTGGGGTTACCACCATCAGTACCACACCCGCCAACGCGGGCTCCAATCTTCTATCCGCCACCG
ATGCCATGGGTTCCTCTGAGAACAACTTGACGGTCTACTTCTAGAGCGCAGGAGGGGAGGCAGCTACTCCCGGAGAGCTT
GTAAATATCGCTGCTAAGTCAGTTTAGGATTAGGTTTAGGCCCCGCACACGTGTATGTACATTGAGTAGACCAAATATAG
CATTGTAAACAGACCCAGCACCAGATCTAGGCTAAGTCCAAGCGATTTCCATTGTAAAGTTTTGCTTC***CCAGATTGTAAG***
AGGATCCGTCCAATAAAGTTTTCAACTATTC

33. Cah2

AATTAGTGACTTGTACAAAAAAAAAAGGAATACAAAAGAGTCTATTATAAACACGGGACATACATAAGTCTTCTTTGGTC
TTGGCCCAAGATTTTGGCTACGAGGGCAGACATGGACCCGAGCACTGGAGCGAGGATTACGCCCGCTGCAGTGGAAAGCA
CCAGAGTCCCATAAACATCGACCAGGTCAGCGCCGTGGAGAAGAAGTTTCCCAAGCTGGAGTTTTTCAATTTCAAAGTGG
TGCCCGACAATCTTCAA**ATGACGAACAACGGTCACACGGTGCTGGTGAAGATGAGCTACAACGAGGACGAGATCCCAAGT**
GTGCGTGGTGGTCCTTTGGCGGAGAAAACTCCTCTGGGCTATCAGTTCGAACAGTTCCACTTTCACTGGGGCGAGAACGA
TACGATTGGCAGTGAGGATTTGATCAACAATCGTGCCTATCCCGCCGAGTTGCACGTGGTATTGCGGAATCTAGAGTATC
CGGACTTTGCCAGCGCCCTGGACAAGGATCACGGCATCGCCGTGATGGCCTTCTTCTTTCAGGTGGGCGACAAATCAACT
GGTGGCTATGAGGGCTTCACCAACTTGCTCTCCCAGATTGACCGCAAAGGCAAGAGCGTTAATATGACCAATCCACTTCC
ACTGGGCGAGTACATCTCGAAGTCCGTGGAGAGTTACTTTAGCTACACTGGTTCTCTGACCACACCGCCTTGCAGTGAGG
AGGTCACCTGGATTGATTTCACAACGCCCATTGACATCACGGAGAAGCAGTTGAATGCCTTCCGTCTGCTGACAGCCAAC
GATGATCATCTGAAGAACAATTTCCGACCCATCCAGCCGCTGAACGATCGCACCTTGTACAAGAACTACATAGAAATTCC
GATTCACAACATGGGCTCGATTCCACTCGTCGATGCGGAAAATGCGGCTGGCAAGTGGAGGGCACAAGCCGCAGCAGTGC
TGTTGCCCCTCGTTGTCCTCGCTGCATTGTCAAGGACCTCCATTTTCCGGGGTTTTTAATTAGAGTTCTGCGGTGGTAAA
TCGAAGGTTAAATCGTTAAATGCCTAGCTGGCGCTTAGGCGTCCAATTAAGCGCTGTAATTTACATTTTCCGCTCTTGTT
CGCCGTCGCTGTTTGTTGTTCAAACACGAGTCCCGGTAATTTCCTTCGGCAAGGACTCAACTGCCGTCAGGACATCCATT
CATATCCACACACACACACATATATATATATATATATAAATATATATATATGGGCAAACAAACAGGCGGACGGTGGGCGAAA
TTATTAAACCCCAAATGGGCGGAGCTTCAGCTGGCACGAAACCCTTACTCATCAATTATGCAGCACCTCGCACAGCGACT
GTCAACCGAATCGTACCCCGTATCCGTACCCGTTCCCGTACCCGTACCCGTTCCCGTACCCGTAACCGTATCCGTATCCG
TATCCCC***ATATTCCCTTCCGTCCGAGGAGG***CCCGCCAGTGCGAGGGGAACCCCCTGGATGTCCTCGCTATTGCACATTGT
GATTTTAAATGCTTGGCACAAGCAGAGCTTTTTATTCGTGTACCTTAGATTTAAGTTTTGTAAACATAAAACCGTAATAA
ATTTTATTGTTTAACATCT

34. CG12880

CATTCAGTCTTTGGCGAGATTTTATCGTGTGCGGACGTATCTGCGGTCGAATTGCGGAACTTGAAAATCCAAATCCGAGT
GGCAAAGATACCGAATTTCGAACTGAAAACCGTGCTCGTAGTTGACGCGAATTTTTAGATGCACTTTCTCAGCATTTGGC
GCCACAAGCAAAAGACTTAGAAACTCTAATTCGATTGCGTTTACTCGAAATCGTTGCTTGCCAC**ATGCTAAAAGTGTCAG**
TCGGCAAGCACAAGTTCATCAACATGCGTCCAAGCTGGATGGGAACGGCGGCCTGGCTGCTGGGGCTCTACTGCCTCCTG
TGCATCGCCGCGATGGTCGAAGGTGCCAGCAATTCGGTAGCTCTGGCTGCGCCCACTGCATCCGCTGCCAAGGATGTCGC
CCATATGCACAGGTCGAAGAGGCTGGACAGGCGATCAGGAGCGGGATCGAGTCCTCTGACGGCTATCAAGCAGCCCAAGC
AACTGGCCAGAAGCGCCCATGGACTCAACAAGAAGCTGCTCAGCCAGATGGGCTTCATGAAGGTGAAGGCGCCCAACAGC
CACAACAGGAAGCGACTGGCGGTTGAGTCACGACGCCACTCCTCGCGCGATGATTCACACATGTTCATCATCAAATTGCC
GCCAAATATGCACTACTATGCTGGACCCGGTGCGAAGAATTCCATCGCAGAGCAGCAGGACAAGTCTGGCCACAAAGGTA
ACGCACCCAGCACGATGGCATCCAGCACACCAGTCGCAGCAGCCGCATCAGCAGCAGTTGCAACATCACCAGTTGCCGAA
GCGTCGGCGTTGAAAGCTAACGGGAAAAAGGTCTCGTTTCCCTTCAGCTCGAATGGCCGGCCGGGCCGCATCTACCACTG
GAACCTGCCCGTCCTGAAGGAGGCGCTGGCCAAGAAGCCCCACTTCGCCCACGTCTCGGCGGCGGATCGCAATAGACTGC
TGGACATCCAGGGTGTGCCCACGTGGCGCCAGCCCTGGGAGAACGAGACCGCCGAGAAGGGCTTCAGTGCCGGCGGCGGG
AAGGCCAAGTATCGCAAATCCCTGAAGCCCAAGTCACCCACATATTATGCACCCGCCCAGGCGGTCAGCAAGCAGAGCTT
CCACAAGTACTTCGCGGGCAATGGCAAGCCCAAGGGATTCTATGTCATCAAGGAGCACCAGAAGAAGCCGCAGTTCTACC
AGAACATCATCTCGTAATCCAACGGCATACCCTCGGTATACCCAGGGAATACCCTTGAAAGACTGCGGTGCCGGGCGTTC
TATTTTCAATATTCTAACGAAGGGGAGCTTCTC***CCCTCTTCCGTCCAGTTTCCACT***TGGAATACCCAATCCTGGCGAATC
CCGATCACATCTATAGCATCCTGGCCAAATGTTTAACAATTGCGATGGGTGCGTTCTATTTTTATATAACGGCAATGAAT
ACAATAACAGGTGGGCTGGTCCAGGTGGGGTGCATCTCATATCCATCCATGTGGTCAGCCATATCAGCACAAAGAACAAA
GTGTCAAAGTGACATGACTTCATTCGAGATTTCAATGTAAAAACAAAGAGTTTAAAGTTCAGAAAGGGGTTTTGATTTGA
GCACAAACTGAGTTGAAACTAATCGGATTATATATTTAATTGAAATTTAATATCTAAAGTTTAATCAGCTTAGCAGCAGA
GTGTGAAATATTCGAGTGGCACTTCTTCATACACATATCGTTTTGATCGGAAATCCCAAAATCCTTTTCCTATGAAGGAC
ACCCCATCCGAAGGACCAGTCCAAACTGCATTTAGCTAAGCAACTGCGGCAAGCAATAAATTATGGCATAATCAAGCTAG
GTTTAAGTGCATGAACGACTTTGTTTTGATAAATAAAAATATTGACTGATTTTTAAGTGCAATTCCCTTAAAGA

35. CG14785

TAGCATACAACAGTCTCCTAACAGCTTCTCCGAAACCCACTCCCAATCTAAATTTTAGTTTTTATTCGCGTTCAATCTGT
GCAAGTTGCAACCCCTCCCTATCCAACC**ATGAAGCGGCTGTCCCGACGTCCGGTCTCCGATGAGCAGAAGGCCCAGATGG**
GCAACCAGATTCATGAGCAGGGCGCAATAGAGGTATTCATGTCGGTGGCCACCGTCTCGCCGCTACTCCGAACGCGGGTC
CTTCCGCAGCTAAAGGATCAACAGGATCAGCTGAAGGACAAAAAGCAGGACCGCAGGCCGAAACCGGAGCCAGAGAAACT
CGAGAGCGGCGCTCAGGGCGACGCCGGTTCCGCTTCCTCGACGTCGGCCCACGCTGTAAAGCTCTTGCCAATCGAGGGAG
ATCCAGCCAAGCTGAAGGCGCTGACCAGCCGCCAGCAAATTGCCAGCCTGCTCAATCACCTCGGCGACGGCGCAGAGGGC
GATGGCGAGGCCAAGCAGATCGTGATGGAGCTGCTCGGCAGCGGGGTGCAGCCGCACTCGGCCATCGAGCGCGTTTCCGA
ACAGCTGTTGCACGTGCTGCGGGAGCAGACTCTGATGGAGGCCGGCATATTCGTCGGCAATGAGCGGTATCGCTTCTTTA
AGGGCGTGCTGCAGAACTACGCCAAGATATACGCGGCGCGCAAATTTATAGATAAGCTGCCGAAGCTAGATCAGCTTGGC
CCGGATGCGGGTGCGGAGCGCGAGCTGTTCGCCCGCGTCGTTGCGGAGGAGGTGCTGCAGATCCCGCAGCTGCTGCGCCA
GCTGAGGTCGTCCCTGTCGAAGATGGAGTTCTTCAACGAGGACACCGCCTTCAAGGCCTACCTGGAAGTCCGTTATCACC
CGCAGGCCCGTATCCTGTGCGAACTAATGCTCACCCGCATCTCTCGCGTCTTCCTCTGCATTGTCGGCTCACCATTCTTC
CTGTACTTCACCGAGCACGAGCTCACGCACATCTTGCGAAACTGTTACCTCAGCGTCAACTCGGAGATCGAGATATTCCT
TTCCGTGGTGCTCTGGCTGGAGCACAACTGGCTTGAGCGCGGCGACTGCGCCGAGCGCATCTTAGCCGAGGTGCATTTCG
CCTTGATGCCGACTTGGTACTTGTGCACCCTGGACAGGTCCAACCGGTGCAACCACTTTGCCCGAGTAATCCACAGTCCC
GGCGTGCAACGCTTGATTGCCGCAGGCCTAGAAGACGCCATCACCCTCAAGTCTAAGCCCCGCTTCAGTGGCGCCGCTTT
ACACCGACAACGAAAGCATCAGGAGGAGAGGCTCTTGGTCCGCGACTGGATAGTGGATACTGAGTGCAGCCACCACCATA
AGAGCCACTGCGAGCAGTTCGTTTACCCCACCTACGACGCGTTCAAAGAATACCTGGCCAGGATTATCTGCTGCGCGCCC
AACTGCTGGCGCACGTTCCGTCCTGCCCAAGAGGTGTACCGCAGGGACTTCCGCTGCTGCTCGCTGCCACACTTCAACTA
GACTCAGCCCGCGTCCCAGTATTCTGTACCTTCCTGCTACTTCTGGGCAGTCGTGATT***CTGTCAGTTCCGTCCATTGTCC***
TTTGCCGTGTCCATGTTTTTTTTAATGCCATCCACACCACATCCATAATCCTTTACAACACCCTGACCAATTCACCGACC
TGATTTAGAAAATAAAAGCATTGTTGGCCATCC

36. CG31495

GCAGTTATCGCAATCGATTTTAGGTGGATAACATAATATACAAAACCATTTTGTAAAGAACAGCTGATACAATTTGACCA
TTTCATATCCAATATAATTTCTCCATAAAAGAAGCCAACCCGGGCGCCAAG**ATGGTCAACATCACTTTACCCAACACTGA**
GGAGCGTGTGAACCTCTTAAAATCGCGCATCAACAGGCTGGGTGACATTACGGTGGCCGGCGATGTTTGCGCACGCGAAA
TTGCAATGAAAACCAAAAATTTCACGGAGGACGAGCTGTGCCAACTGGTCCGAGAAGCCCTCCATGAAGCCATTGTCCGC
ACTCACGTCACCAGATGCCCGAAGGGCCTTCAAGTCACTCAGGTCGATCTCCTGGCCGCGGTAAAGATCATTCAGCCACG
ATTTGGCCGGCAGGAGGAGACTCTCCAGCTGCTCATGCCATATGAATATCTAGATCGTACCGCTTTCGATCCGAATGATC
TCCTGTCCACCGGACGCCCAAGATGGTCTTGCCATCTGGTCGAAGGTAAGCCCAAGTCAGGCCTAACTACGGTGGCCGCT
CAAATGGCTTTGAAAACAGACTGCCCTTTTATAAAATACATCTCGTCGGCCGAATTGCTGGGACTTAGCGATTCCGAAAA
ATGCCAGAGAATACGGGAAGTCTTGGAGGACGCCTATGTTTCCAGGCGCAGCTGCGTCATCATTGACGACTTTGAGCGTG
TCATTGGTTACGGCGCCCTGGGTAAGCGGTACTCCAAGGAGTTCCTGCAGAAGCTGACGGTTCTTCTGAAGAAACAACCG
CCCAACAGCCACGAGCTCATCATCATTTGCACAAGCAATCGTCTCGATGTCCTGGAGGAACTGGGCTTGCTATCCGTTTT
CACTTCAGTTCACAATGTGCCAAATGTTTCCACGCCTAAAGAACTTATGGTCATAGTGGAAGCTTCAAAACGATTCGAGC
CCGAGGAGCTACGCCAAATCGAGATCGCCATGGATGGCCGCAACGTCTCCATTGGCATCAAACGGTTGCTTGACTTGATT
GCGTGGGTGAATTCCCTAAATCCCGACCGTCGAGCTGCCAAATTGCTGAAGAAATTGGGCGAAGCAATGGGATGGGTAGG

GAACCATTCTAGTCCACTTGTACCTCAATACATTTAAGACGTTAGGTTAGGAAATGCAATATTGTATCTACAATTCCAGG
AAAATAATTCCAAGTGATTCCAAATAATTCAGTAATGCTTTATATTTAAAAGAACCACGTAAGCATAAAAAAACCATCTA
AAAAAATAATATTGCATTATTTTTGGATGAACTTGCTTCCATTGCATTGCAATGTTCTTAAGACTAAAATGAACAGCCC
GATAATGTGATTGCAAAGATACTAGATTATTATATTATATATATATTATATATACACCAGGCCAGACCTCTCAGTGGCCT
TCGCCTGCTGTA***CGGAGAGCATTCCGTCCTCCAGG***AGTTCGGTGACCGGTGGGCCCAACATCTCCTGGGCTGCTCCAAAG
GCGGGCTTGATGTCGTTGTCCAATATATATATATATACATAATTATATATATAATATATATTATATATTATATATATATA
TTATATTGTTCTAGTGTCTTTGGTGATTGGCATAACAAGTTTTATTACCATATTATATATATATAAGTAAAAATAAAGAT
ATTTGAATTGCAA

37. xit

ATCGGACAGCACTTACCGCTGATAAGGAATGCATCGAATCTAAACTACAATCCCAATCCCTGAGAGAAACGAAG**ATGAAG**
GACCTCTTCTGGCACCTGGTGGGCATAGCCACCGGCCTAAAGATCCTGCTGATTCCAGCCTACCATTCCACGGATTTCGA
AGTCCATCGCAACTGGCTGGCCATCACCCACAGTTTGCCGCTGAATCAGTGGTATGTGGACGCCACCAGCGAGTGGACAC
TGGACTATCCGCCGTTCTTCGCCTACTTCGAGTGGCTGCTCTCGCAGGTGGCCAAGTACGTGGATCCACGAATGCTGGTG
GTGGATAACCTGAATTACGAGTCCAAGGCCACCGTTTACTTTCAGCGGCTCTCGGTGATTGTTACGGATCTAGTGTATGT
GCTCGGCGTGCGCAGCTGCCTGGGCTCACTGGGATTGGGCAGGGATACGCAGCAGTTCTTTGCCGCCTCCATGTTGCTGC
TCCTCAACGTGGGATTGATCTTCGTGGACCACATTCACTTTCAGTACAATGGCTTGCTGTTCGGCATACTGCTCCTGAGC
ATCGGTTCCCTCATCCGGCAGCGGTTCCTCTGGAGCGCCTTTGCCTTCGCCGTGCTGCTCAACTTTAAGCACATCTTTCT
GTACATGGCGCCCGCTTTTGGGGTCTACCTGCTGCGTTTCTATTGCCTGGAGCAAGCGAGTGTGGTGTCTGCGGTGGGTG
CCGTTGTCAAGCTGCTGGTCGTTGGACTTACTCCGTTCGCCGTGTCTTTCGGACCCTTCTGGCAGCAATTGCCGCAAGTC
CTCTCCCGTCTGTTTCCTTTCAAGCGTGGCCTAACGCACGCCTACTGGGCACCCAACTTCTGGGCGCTGTACAATGCCGC
CGACAAGTTGGCCGCCGGAGTTTTGAAGGTACAGGATGGCGGTGCCTCGACGACGTCAGGATTGGTCCAGGAGGTCAGGC
ACTCGGTGCTGCCAGCCATCACACCGCCTGTGACTTTTGCCCTGACCGCTCTCTTCATGCTGCCCATTTTGGTGAAGCTC
TTTCGCAGTGCCAAGAAACAAAGTCCGCTGGTCTTCCTGCGAGCGGTGGTTTTGTGCGGCTGTTCCTCCTTCGTCTTCGG
CTGGCATGTGCACGAGAAGGCCATCTTAATGGTGCTCCTTCCGCTCTGTCTGCTCACGCTGGTAAACCGGGAGGATGCCA
GATACGCCTATGTCCTTGGCATTGCCGGCTATTTCTCTCTCTTTCCGCTGCTCTTCGATGCGGATCTGTACATTCCCCGG
TATTCCCTGTACATGTCGTACGTGGCCATGATGTACGGCCAGCTGTATCGCATCTTCCCCGGCTTCCGAGGCTTCCACAC
GCTGGAGTGGCTATACATGCTGGGCTTCATGGCTATTCCGCTGTACGAGCATCTGCTCTCTTTCCTGCTGCACCTGGACC
AGCGCCTGCCCTTCTTGCCCCTACTGCTGACCTCCGTGTACTCGGCGTTGGGAGTGCTCTACTTCTTTGGGGCCTACTAT
CTGTACGCTCTGGGCATTAGCTGGGGCAAGGTGCCCATCGCATCGTCCACATCCGCGGCCGCTGTTAAGAGAAAACGAAA
AACCAAATAAACGGAGCGCTATAAAGGGCTTCAGCAGAAATCCGCAATTGGTCACGTCTTGCATTCAGAGTGGTAGTGCT
GGTGGTGCAGCCGAGCAGTCTTCAAGGCGCCTGGCGCAACAGCCAGTTCGGCGCCGCTGGACGGGATTCTCTGGTCCGAT
CGGCACAATGGACAACGAACAATGGCTGTTAAGCAGAATTCGGTGGGCAGGTAGCGGTCACCGGCGGCGTCTAAGGTGCC
ACGTACCTGTTGGCACGACCAGATTATGGCCTTTTGGGCCATAAAACAGGTACGAGGCGGAATAGTC***GGCCGGCCGTCCG***
TCCGAGTGGGTGACATTCCTGGAAAAACCGTGGAAAACACGCTGGAGCTGAATGAATAAGTGTATATCAATTGCCAGCCT
GGGCAGGGAATACT***TCAGTTGACGAGCTTCCGTCCAG***ACGAACAGATCGACATGCAGATGCACATGGAACTCTCCTATGG
CCGCGGCTACGATCCAATCGGGAGGCAACCACCGTCTCTTCCTCCTCCGTCGCAACAGCACTGGTCCAGCAATGGGTTCT
CCGACGACTCCGCTTCCTGCTTCCGTCGGGGATCCTATATTGAACTGGCCAGCGGAGCCATGCGTCGCGTGGAGGACATA
CGCACCGAGGACTTCATCCAGTCGGCGCTGCGCAGTCAACTATTTGAGCTGCGAGAGGCGACAGTAGTGAGGATAGACTG
GAGTGGATGCCCCAGCCTGGTGACACTCACCTTCAGCTACGACACACACCACGCCAAGATGGACTTGCAGGTGCAGCCGG
GTCATCCAATGTTTGTTTACGGACAGGGCTGGGCCTCCTGCGATCCACAGTTGTCGCTGCAGCTGTACGAGCTCAAATGC
CAGCAGCTGCAGGTGGGCGACATATGTTTGTCGCTGGTGCCAAACGAGCAGCCCGCCGCACCATGTCCGCCTCAGCCGGC
GCCGCCACCGCCGTGTCCGCCTCCTTCTCTGCCCCCGTCCATGGAAATGCCAGTGCCGATGGGTTCTCCGCCTACCGGAA
GCTATGGATTTCTGCCGTTTAAGCATCCGTACGAGATGTACGCCCAAATGGCCAGTTTTGTGGCCGTGTACACCCAGCAC
ATGATGGGAAAGCTAAACTATTAAGCCACAAATTTGGTGATTTAACTGGCGTTTCAGGAATATATTCAACGCTATTCCTT
GAGTACGCCACGCATTTACAAATTTAATCTAGGTACAAAAATCACAATGTATTTTGCTTAATTTTCTGAAATAAAGTTTA
AAGTAGCTGTAA

38. crim

CTCATTTGCGGTCACACTGCTCTCTAGGGCGTATCTTTTATTATTTACGTTGTGTATTTGTGTTATCTGTTTTATTTTGA
AAAGAAACCCCGCTTTATTAAACAAAAC**ATGCACTACCATACGAATCTCATTGCAGCCCTGCTGCTGGCAGCTCTAATTC**
ACGAGGGCTCTGCAATTTGGTGCTACCGCTGCACTTCGGCCACGCCCGGCTGTGCGGAGAAGTTTAACTGGCGGGGAATA
GGATTCCTGGGCGAGCACTGCCCGGAACCGGACGACATTTGCGTCAAGGTGACGGAGCGACGCGGCGCCAGGGAAACAAT
CACACGTGACTGCCTAAGCGCGCTGAGCTTCCGCAAAGACATTCCCGCCGACAAATACGAAGGATGTCGTCCTGCCGCCC
ACGATGAAAAGCTAGCGAACTACGTAAACCACACGATCAAGGAGCACGACGTGCGCCGTGACTACTACACGGACACCACG
TTCTGTTTCTGCTTCCTCGACCATCGATGCAATGGGGCCAGCGGCCTACAAACGTCCGCCGTAATCGGTCTTCTCACTCT
AATTCCTGCCCTGCTTCTACGCTAGAATCTCCTCTTGAGTGCAGTATTTAAGTGCCTTACTAACTCGCTCTGCAA***CTACG***
AATTCCGTCCAACATTCCACACTTTTTGTACAACCTTTTGACATGACTACAGCTTTTAGAGTCTTGTGTTTACTCAACTA
ATTAAATACATTTACCATATTTATAAACAGGAGTGAAATCGTTGCTTTAAAGGACCTCTACATTCTTGGGCGCCCGGTA
ATCATAGGCAGCCACCTCGGGTCGCGTCACCAGATGCTCCGAATAGACTTCC

39. Cdc5

TATCGCTGTTATCGAAGACTAACCATCGCTAGCGGCTACTGCAAAAAAAAGAGCGGAGTTTCGTGGACTCGAGTGAAAAT
TGTGAAAATCCATTGTATACGCGACAGAAAGCAACATAAGGCCCCAGA**ATGCCGCGAATAATGATCAAGGGCGGCGTGTG**

GCGCAACACGGAGGATGAAATCCTCAAAGCTGCGGTTATGAAATATGGCAAGAACCAGTGGAGTCGAATTGCCTCCCTCC
TGCACAGAAAGTCCGCCAAGCAGTGCAAGGCCAGGTGGTACGAGTGGCTGGATCCCAGCATCAAGAAGACCGAGTGGTCC
CGTGAGGAGGACGAGAAACTGCTCCACTTGGCCAAGCTGATGCCGACGCAGTGGCGCACCATAGCCCCGATCATCGGACG
GACGGCAGCCCAGTGCCTGGAGCGCTATGAGTACTTGCTAGACCAAGCCCAACGCAAAGAGGATGGCGAGGACACGATGG
ATGATCCTCGCAAGTTGAAGCCCGGAGAGATTGACCCCAATCCAGAGACCAAGCCGGCGCGGCCCGATCCCAAGGACATG
GACGAGGACGAGCTGGAAATGCTGTCCGAGGCACGCGCCCGCTTGGCCAACACGCAAGGCAAGAAAGCCAAGCGCAAGGC
TCGCGAAAAGCAACTGGAGGAAGCCCGCCGCCTGGCCACTCTCCAAAAGCGGCGCGAACTTCGTGCCGCCGGAATTGGCA
GCGGAAATCGCAAGCGGATCAAGGGAATAGACTACAACGCCGAGATTCCCTTCGAAAAGCGACCTGCCCACGGATTCTAC
GACACTTCCGAGGAACACTTGCAGAAAAATGAACCCGACTTCAACAAGATGCGGCAGCAGGACCTAGATGGAGAACTCCG
CTCAGAGAAGGAGGAACGCGAACGCAAGCGGGACAAGCAGAAGCTGAAGCAGCGCAAAGAGAATGAAGTGCCCACCGCAA
TGCTGCAAAACATGGAGCCCGAGCGGAAGCGCTCCAAACTAGTCCTGCCTACGCCGCAGATTTCCGACATGGAACTACAG
CAGGTAGTCAAGCTGGGACGCGCCAGTGAGATGGCAAAGGAAATTGCCGGTGAGAGCGGCATAGAGACGACGGATGCTTT
GCTAGCCGACTACTCCATTACACCCCAGGTAGCAGCCACTCCACGTACTCCGGCTCCGTACACCGATCGCATCATGCAGG
AGGCCCAAAACATGATGGCCCTCACCCACACAGAGACGCCACTCAAAGGTGGCCTGAACACACCGCTGCACGAGTCAGAC
TTCTCCGGCGTGCTGCCCAAGGCGGCCTCCATCGCCACGCCTAACACAGTGATTGCCACGCCGTTTAGAACCCAACGCGA
GGGCGGAGCAGCCACTCCCGGCGGATTCCAAACGCCCTCCTCCGGAGCCCTAGTTCCCGTAAAGGGGGCAGGAGGGGCTA
CGGGTGTCGTCAATACACCTGCCTACGTGAGGGACAAGCTAAGCATCAATCCTGAGGAGAGTATGGGCGTCACCGAGACC
CCAGCACATTACAAAAACTACCAAAAGCAACTAAAGTCTACACTGCGTGACGGTCTCTCCACGCTGCCCGCTCCACGGAA
TGACTATGAGATCGTGGTGCCTGAGCAGGAGGAAAGCGAACGCATTGAGACCAACTCAGAACCCGCCGTCGAGGATCAGG
CGGATGTCGACGCCAGGTTATTGGCTGAACAAGAGGCGCGCCGAAAGCGAGAGCTAGAGAAGCGCTCCCAGGTTATTCAG
CGTAGTCTCCCGCGACCAACTGAAGTGAACACCAAGATTCTGCGACCGCAGTCCGAGAAACAGAACCTTACGGAACAGCA
GCAGGCCGAAGAGCTGATTAAACACGAAATGATCACCATGCAGCTGTACGACTCGGTCAAGGATCCCGTGCCTGGTCAAT
CGCAGCACAAGTTGGAACAGCTACAAAGCTACTTTAAGGCCAATCCCTACGAGGACATCTCGCAGCAAGAGTTGGCGAAG
GCCAAGCAAATGCTCACCGAGGAAATGGAGGTGGTGAAGGAGCGCATGGCGCATGGCGAACTGCCCCTAGACGTTTATGC
CCAGGTGTGGCAGGAGTGCCTCGGCCAGGTTCTTTACCTACCCTCCCAACATCGCTACACACGCGCCAATTTGGCCAGCA
AGAAGGATCGATTAGAGTCTGCCGAGAAACGGCTGGAGACAAATCGCCGCCACATGGCCAAGGAAGCCAAGCGCTGCGGC
AAGATCGAGAAGAAGCTGAAGATTCTCACCGGCGGCTACCAGGCGCGCGCCCAGGTGCTGATTAAGCAGCTTCAGGACAC
GTACGGCCAGATTGAGCAGAACTCTGTCTCACTCTCGACATTCCGCTTTTTGGGCGAACAGGAGGCCATCGCTGTGCCGC
GACGCCTGGAATCGCTGCAAGAGGACGTGCGTCGCCAGATGGACCGCGAGAAGGAGCTGCAGCAAAAGTACGCTAGCTTA
GTGGAGGAGCGCGATTCGCTTTACTCTCAAATCGAACATATAACTGGCGTCAGGCCCACGGCCCAGCAACTCCTGCCCGA
CCAGGAAGCGTAAACCTTCTTTTGGAACCCAAGTCCGTTCTTATTTAATTACAGTAATTACATGCCAGCAAACAGCTATC
ACCACCTAATATCCCGATCCATATTCCTCGCTAGCGGCCGTACTTCTGGTAGACCCACTCCTTGTTGTCCAGCGGACAGA
CCAGGCGCGTTTTCAACCAGCGCGCGATGCAGTGGTAATGGAATGCGTGGTTGCACTCGCCCCAAGCCACAGTGCACTCG
TCTTGGTTTGCATTCGGGTCCGCCTGGCACTCGATGCACAGGTTCATGATGTGGTTACGGCAGATGGCACAGTTGTCCAC
TGCTACGTCCCATCCCCACATGGCGTGCGCAACCCATTTCTTCACCA***CAAAGCGCTCCGTCCTGGCCTGG***ACAGCTCCTC
CACTACAGGATGGCTCTTCGTCATTGAAGTCCATGTCGTGGAAGTCCTCCGTCTCCTCAACCTCTATCTCCTCGGCCATG
GTGTGAAATTACGAATTTTTTTCTTTTTCAAAATACAACACTTATTTTCGTATTCGAATTTGATTTGAACTATTTTAAA
TTCCATTTGTTTTTGGCCTGACAGTTAATAAAAAAAAGTTGACCACTACCGATTTATCTACGAAATAACAACGGCGAATA
ATTTTAAAAATAAATGCAG

40. nemy

AGTTGTCTCTAGCTGATCGCCGCGATCTGATCGTCGTTACACTTCATTTGATAGTAAAATCAGTGAATTTAATTTAGGAT
TTTCGTGCGGTTGTTTGTAACCGATATAGAAGCACAATTACGCGGAAAACGACCTTTAGTTGGGCCATCCCAGATATATT
GTATATACACCATATACACCTATATAGAGCCGATCCGTGACGCACCGCCCAGCGATTCTACGTTTAGTGGGTGGCGGTGA
CAGCATAAGCAAGAAAAACCCAATAAATTTCGGCCCAAATAGCAACACTTGTCTAA**ATGCGAGATCGTCACTTGGCCAAA**
ATGCCGCGTGGAGGAACGACAACATCGCGCTATGAGGAGGATCCAGATAATGCCTGGAATTGCTGCTCCTGGTGCGAATA
CTTGCTGATCGTCATCCTGTCCACGATCCTGCTGGTGGGCGTGCTGGTGCTAACCCTCTTCTGGGTGATGTTCTACCGCG
ATGGCTTCGCCTGGTCGGAGAGTCCCAAGCAGCAGTTCAATCTGCATCCCATCCTCATGATTGCTGGCTTCGTCACGCTC
TCCGGATTTTCCATCTTGATCTATCGTCTGTGCCGCTGCGTGAAGCAAATCTATGTGAAGCTGATCCACATGTTCTTCCA
CGCCGTGGCCATTCCGTGCATCGCCCTGGGTTTCATATCCGTCTTCGCTTCCCACGACGCGTTGCATAAGGTCAACTTCT
ATAGCCTCCACTCGTGGCTGGGCTTCGTGACAATGGGCATGTTCGTGCTCCAGTTTGTGATCGGCTTCTTCAGCTTTCTG
GTAATGCTGTGCTGTGAGAACAAGACCTACAGCTGCCGCTCGGCCATGGTGCCCATCCACGCCAGCTTGGGACTGGCCAA
CTTCTGGCTGGCCATTGCCACATCCGTCACGGGACTGATCGAGAAGGAGCGCGAAACCGTCAACGAGGCGGGCGTGAGCA
GTGAGAACAAGCTGGTGGAGCACTACATCACCAGTGCCATCGGCGTGACCCTGATCTTCATTGGCATCATCGTCACCTTC
GCGGTGCGGCGTTCCAATGCGCCCGCCTCGGCGAAGGTCTACGTCACGGA*GCGCATTTAGACGGTCCGTCCGG*CTCGTCA
GAGTCTCCGCCGCCGGAATTGGTCCACGGCCAGGAGGGTAAGCGAGTCGCAGCAGGTGTACCCGAGGATTCCGGAACTCG
GTGACTTGGACTGGACTTGGGGAAACGGACCGGCGGTGTCCTTTCGCCAGGCTTAGCGCACAGAGACCACGCTTCTGCAA
CTCTATCCGAACCCTTTGTAACCTTTTATATGCCAAATTAAATCCACATCATACACTTAGACTACTCCATATGGCTATAA
TCAAAACCTTTGCCCACTTTCAATCTCTCAGTAGGTGTGTAGGATTAGGAATCTCAGCAGATGAGCAGAAGACCAAGCAA
GCAAATCTAATCAAAATTTTGACGTGTTCAGCCGAACTTACTGATGCATCCAGGCACTTGCAAGCTAGATTAGCCAGTTT
TTTGGTACTATAGTAGAATTTTGATATACTCGTAAGCCCATGTAACTAGTGTATGTATGATCGTACCACGTAACGCATTG
AATGTACGCACTCGGACTCAAAACTGTCGCATTTGATGTTTTTTTGTAATCGTGCAAGTATTTGTATTTTCATAATGCGC
TAAGCAACCTAAGCTGTAAACCAATTTTACAAACAAATAAATACTTTTGCCCTGCCTGAGGGCTCTCATAATAATCGCAA
TTGTTTATGCACTTTTTAAATATTTAAAAAACGGTGAGAACTCGGAAAATAAACAAATCATATTCCCTTGAAAGGGAAAT

TAAACAG

41. Dok

TGCAACCGTGCAGCGCATAAATAAATAAAGTCGACTTGCCTCTGTGCTCGCTTTTTGGTGATTTTTTTTTGTGCTGCGGC
TGCGCTTTTCGAAATTCGAATCTCCGTATTCGCTGTGCGATATGATGTGATGTTTACTATGAAGTGATCAACTAAATGCG
ACCGCGAAGCTATCGTAAAGCACAGAAAAACGGATGAATTGAAGGAAAACTCCAGAAATCGCATAGCCCAACTCCAGCAA
CAACAACAACAACAACAATTCGGAATCGGAGGCGCATTGTGTGAGCTAGATCGGCGAAGGAGAGGGAAAGAGAGCAGGAG
ACGAAGGAGAGTGGGAAGAAGGAGAGGAGAGGTGGAGGAGTGGTGCACAGA**ATGGATGTTGAAATACCTGTTTTGGCTGG**
ATATCTTAATGTGCCGACCCAAACTGGATTTTCGCTAAATCGCATCTCGAAAAAGAAAGCATGCAGTCGCTACTGTTTGC
TATTCAAGGCCAGTCGGCATGGGATTGCCCGCCTGGAGATGTGCGAAAGCAAGGAGGACCGGAACCCCAAAATCTTCACC
CTCGAAAACTGTGTGAAGATCACTCAGGAGCCGCCTCCCGAACGCCTCATTCACATTGTCAAGCGGCAGGCTACCCTAAC
ACTGAGCACAAGTAGCGAGGAGGAGCTCAAGGATTGGATCACCGCCCTGCAAACGGTGGCCTTCTGTGACACCTCACCAT
CGGGGGGAATCGGGGCCATCGAGGAGGACAACGACCTCTACTGCAGCTCCTTTGATGGCTTGTTCATTATCACGTTGATT
CCATCCGAGGCCTCCATTCGTTGCTGCATTGAACCTAAGACATATATGCTCCAAATGACGCCCACTGAACTTCAGTTGAA
ATCGGAGGATTTGGGCGCCACAATCGCCATGTGGCCATATCGTTTCATTAGGAAGTACGGCTATCGCGATGGCAAGTTCA
CCTTTGAGGCGGGCAGAAAATGCACCACCGGCGAAGGCGTTTTCACCCTGGATCACACCAATCCACAGGAGGTATTCCGC
TGCATGTCCGCCAAGATGAAGTCGATGAAGAAACTGATTAGCGGCGATAGTCTGAGCACCTTGGAGTGCGGTGAGAATCA
GTTTAGTGCGGCAGCTGGCATGGAGCCCGGTTCGCGAAGCCCACTGCCACCATCACCGTCGAGTAATCCCCACGGTGGCG
AGTTCGAGATCAACTCAACGCAAAGCTGCATATCCCTGAGGGGCTTCATTAGCTCTAACGATTCTCTTAATAACTTCTCG
GCGGGTGGAGGTGGAGCAAGTGGCGCCACAGCAACGGGTGGAGGCGTTTTAGGAAGTACCGGAGGAATCACGCTATCCGT
GCCCGCAAATAGCAATAGCAACAGCAGCAGCAGCAAACACATTCCCAACAAACCGCCACGCAAAAGCCCGACAACCCATT
GTGATAAATACAGGAATATTGTGAAATATGAACCTGTGGCCATAACTACGATATCTTCGCCATCCGCCAATTCCGATGTG
AACAGTTCAGTGATACTGAAACCCCTGGAAGGCGAATCGATATCCCTGCCGGAAACAGCAGCCGATTTACCGCCCGACTT
GCCGCTGCGAAATGAGAGCGCCTCGAAAGCACCGCCTCCGGAGCGGGATTACGAGTGCATCGAAAATATCACAGAGGCGT
GGCGCACAAGGGGCGTGGGCGATGTTAGGCACAGTGAACGTATGCCCATACTCTGCGATACCTCAGAATTTGTACGCCAG
CGATCAGTATCGAAATCCACCTGTGGCAGCCAAAATACACCCAAGATCATAGACATCGATATCGGTGAGGGTTGCACTAG
CGCCACATCCATTAGTGAATCGAATTACGATCGTTTAGACTTTCTATCGCCGAACAATAAGACATCGAGTGGCTATAAGA
CCATTGTGAATGTCACACCCGGTAATCGCATACGATGCAGTCCACCGCCACCCAATGAATACGAATTGATCGCTAGTCCC
GATACGGAAAGCTTCCGTAAAGCCGATGACAGTCATCGCGGTTACGGGGTGCTGCGAAAGGCTAGCACCACTACGAACAA
TAATAATCTAGGAATCTCCAATAGCAATATCAGCGGCGGTAGTAGCACCATCACAACTGCAACGGCCAACAAAGGCGGCA
ATCTCGAGGCGGCAGCCATCGATCATCGCAGCTATAATGGCCTCAACTACACAATGGTCAGCAAACCGAAGCGAGTGTAA
AATATTTAAGGATTTCAGATCCGATAGCAAGGATCCAGAGATCCTGGCATCGAACTGGGTGCAGTGGGGTTTTGGGGTGG
ATCGGGAGGAGATAGTCCTAGTTTTTATTTGTCCCAGAACTGTTGCTGTTTTCAATTTAATTCAACACAACTTGTTGACA
TGTTAATTTGGATTTTCCCAGTAGTCAAATTCATGACTATTTCGTTATTGGACGACGCCAGATGAATAAGCTTGTGTGCT
TTTCGCGATACATTTTAAGTGTTGCGATAACAAATGTAAATGTATGTTCAAAAAAATTTAAAAAACAAAATTGAAAACAG
TAACGTGCCAGTAACTTTTTACATAATTTCATACATTTTGAGTATAAGTGAAAAAGAAGAGACTGCCGAGTGCCTTACAG
TTCTACAAAAAGAAAGAAATTCCTGGCTGGAAATCGAGGGAAGGAAGTGCGTCGAATCTCTAAATCCCAGAATTTGTCCT
TCCCTTTGCCAAGCCAAAAACAAATACCATTCGAATCGTTCAATTTTTTTTTTTTTTTGTAATTGTTTTTCGTTAATTATG
TTAAATTCGTAATCTGTGATTGTATTGTTAATTGTGAAACTGTTACATTTTTAGCAAGCACTGCCCGCCACTTGATGATC
GCACTCGAAAAATTCCCCCGGCTCTTACTTAAAATGTAGTGTTAAATTAAAGCAAGAGAAAAATGAAACTCAATCGAATC
ATGGACGTAACGAAAATTTACAAAATGTGTATGCGATTTATGCTAAATATATGTGTTTTTGTATATTTAAAGGAACTAGA
TTTTAAATACACTGCAATTGTACTTGTTT***CCGTTTA****TCCGTCC****GTCTTCTCA***AGGTTTTGATGCCATATAAATCGGTTAT
TCGAATTGTTATTCCTATACGTTTTTGTATGATAGATCATATAGCTGCCATAGGAACAAACGGTGTAATGCCATTATCTC
AAAAATCACCCAAAATAGTTGACTCTTATTTTTTTTTTTTTTGCTCAATCTTAGACTGTAAATATACAATAGGTTTCTTA
AGTGTTGTTTTCGATTGCACCTCCTCTCAATAACCCAAATAAGAAACCCGCCCCATCGTTACCCCATGCACGTTCGAGTT
CTCAAGCCAGGATTGTTGTAAACGACAGTTTTGAGTATTATGCAAAGGTCACAAAAAAAAAAAAAAAAAATTTATATAAA
TTAAAACGAACAAACGAAGGCGAACACAAATAAGAAAAATACC

42. Drd

AGTTGACGTTGAATGTTTCGTCTTGGCGGTCGCTTAATTTAATTCGCTGAACGCTGACACAAACGATCCGAAAAATCATC
GATCATCGCCGCAGTTGCAAGACAAATTTACTATTTCAATTGTTGTCATTTTTTTTTTCTGGTGTTCATTTCTTTTGCAT
CTTTTGCTCTTGTGTTTGGAGTATTTGTGTGATTATGTGCCAGAGAACTGCAAGCTTATAAGTCGCAAAAAAGAAAAGTA
CTTAAAAGGGGTTTGGTTAATCCAGGATCCTTGACAGG**ATGTCGCGTATGTCGCATATGCTAGCACTACTACTGGTACTA**
ATTGCCAGTAGTCAGGCGCTCGAGGGCGTGGTCCAGCCCACTTGCAATCTAAAGGACAATTGCAATTTGGGCGATAATCT
TCTGGCCGGAACAAATTCCGCATCCGATGCTGGCGATGTGTCTGCCGATAAGCTGGAGAGCAAAATTGCATCGATTTTCG
AGGATAAGGTCAACTATAGTCTAAAGTTATTTGCGGACGATGCGACGACAACAATCCTGGAATATCAAACGGCCAATTTG
AGTCAGTTGCATGCGGTAACGCCTGGAAATTACACGATACACCAGATGGAACAGGCGGAGGATGATGACGACCAAGTGGA
GGAGGATGAAAGGGCCTATTTGTACAACATTGGCAAGCGTTTTCTGGCCATTACGCAACCCTTCGACGCGGCCAGAAATC
CGGATGCCTCGACTTTGTGTCGCCGGCAGATGCATCAGTTTCTGAACGCCTTGGACAACTTCGATCTGTGGGCTCTGAAG
ATGCACGACTCCAGTGGCAAATTGAACTCGGGCATTCTGAATGGCAACATCAATCAACCCGGCGATTTTGACCAGTGCCT
GGGCATTCAGCAACGCATGAATCAGGATCAGGATGCGGGCCAGGATCAGGATGGTGACTCCATCATTCGGGGCCAGTATT
GTTTGGCCTATGCCCAGCCCGTTTTGCCACACAATTCGAAGCGATTGAAGAGTTTCTTCAAGCTGATCCAATCACATGGA
CCTTTTAAGAGCGAGTTCAACGACCCCGGCCACCGAGTGCCCCGCTACTCCTTGATCAACTGGGGCCTCTGTGTGCCCTC
CGGCTGCTCCGCCCGCGATGTGGAGTACAGTGTGGCCGAGTATCTGGGCAACCAGACCGCTTCCACGGGCATCACCTTCA

ATGTGCGCGTGGAGCCCCAGATGTGCCAGGTGCGCGATCAGCGGCCCTGGGATCGGAACACCACGTGGGCGGTGCGCTTC
TTTCTGCTCGTGCTCTCCGTGGCCGTACTGTCCACCATCTACGATCGGTCCACCAAGTCGCAGCCCAAGCAGAATCCCTG
GTTCACGGCCTTCTCGCTGGACAAGAACCTGCGCTGGCTCTTCAGCACGAGCAGTGCTCCCGGAGACATTGAAGCGGTGC
ATGGCATCCGCTTCCTGAACGCCATCATGCTGATCTTTTCGCACAAGTCCATGGCCATGTTCTTCAATCCGTACAACAAT
CGCACCGCCATGTCAGAGAGTCTGGGTCAGCCGTGGACGGTCATTGGACGAGCTGCCTCCCTGTACACGGATCCCTTCCT
GCTCTTCAGTGGAATGCTGACATCCTACTCCCTCTTTGGTCGCCTCATGAAGCAGCAACCCATCAGGCTGAAGAACGAGT
ACATTAGTCGGCTGATGAGGATTGTTCCGCCCCTGGCCGCCTTGATACTCTTCTGCACGTATGTGCTGCCTCTGTGGGGC
AGTGGACCCCAGTGGAATCTGGTGGTGGGCCACCATGCGGACATCTGCAAGAAGAACTGGTGGCGCAACCTGCTCTTCAT
TCACAACTACTTCGGATTCAGCGAAATGTGCCTGACGCACACGCACCATCTGGGCATCGATACGGAACTCTTTGCGGTGG
CTCCACTCCTCATCCTGGCACTGTGGCGGTGGCCAAGACGTGGCCTCTTCGCCCTGCTGCTGCTCTGCACGGTGGGTACG
GCGGCTAGGTACTACACCACGATTGTCAACCAGCTATCCAACTATATCTACTTTGGTACCAACATTCAGCGCCTGTTCCG
CACCGCCGACTACATGTACTCCTTCCCGCCCCACCGCTCCACGGTCTACATCATGGGCATCCTGCTGGGCTATGTGTTGC
GCAAGTACCAGAATGCCCGGCTGAGCAGCCTGCAGCTGCGACTTGGTTGGCTGGTGGCCACCGTCTGTGTGCTCGCCTCT
CTGCTGGGACCAGCGCCCATGGGTGACATCAACTACGTGTACAATTCCACCCATGCAGCTATATACGCCGCCTTTGCGCC
GATCGCCTGGTGTTTGTTTTTCTCCTGGATCGTCTTTGTCTCCCATAATGGATACACAAgATAAACTGACGAAGCTGTTT
GCCTGGCGCGGTTTCCAGGTGTCCACGAAGCTATCGTATGCCATTTACCTGACGCAGTTCCCCGTGTTCTTCTTTAATGT
CGGCCGAAGGCGTCACATTCACCACTACTACAACTTTGTTTCGATAATTCTCGATACCAACGAATTCATATCGATCTTCC
TGGCCTCCGTGGCCCTGACGGTGCTCTTCGATGCACCGTTCCAGAATCTAAAGAAGCTGCTGATCAAGCGACCCACGGCG
GCAAAGGTGGTAAAGGATAGCAAAGCCAAGGCGGCAGAGTCCCAGGATGCGACGACAACGACACTAGCCTCACAGCCCAG
CAGCACTGCCCTCCTACAATCGCATCATCCGCACTCGGATTAGTCTGGATGCACACACTCTTTCTTAGCTATGTATAACT
TGTAAATGTCTTAGCATTTAGTGGCAATATGTTTAGTGATCCCCAGCGAAATCCCCCCCCCCCCCCCAAAAAAAAAAGA
ATCGCAT***CCAATCCGTCCATGTGCTCATCA***CATCCCACAGCCTTTTCCCCGAATTGAATCCAAGACAGCCCCTAGTTTTA
GTGGAACGCTTTATACATTGTAAATTGTACTATTAGTCAAAACGAGCGACAATTAAGAACCGAACCGAACCGATTCGTAG
TCATAAACCAATTAATCAATCACTGCTTGATGTACGATATTATCTTGCTTGCTAAACCGGGCCTCGAGAGGTAGTCTCTG
CTGGGGAACCCTATGTCATATCATATTCACATGATATTGGGGCCCTGGACGCAGACTCTCGGTGCCCATGTTTATTAGAA
TACTTACGTTACCTTAAGCGAGGTCGAATGTCAGTTTTTAAATGGTAATTTAATTAATAAAATATATAAATATGTTACG

43. fy

TCGATTTAATCGATGTAAGCATCATACGTAGCAGGTTCGGCGAAGAAGAAGAGCTGCG**ATGTCCATCTATTTGTTATGTT**
TGACAACAAACGGCGGATTGCCAGTGTTTACACGAAAGAAGGGAGAATGCGAAAATCTGCCCTTCTCGACGGTGGCCTCC
TTAAACGGCTTTCACATGTTCTTTAAGTCGCTGGGAATCCAACTGGAGGCCACGACGACCAGCAAACAGCAGCCTATCCA
GAATCAGGCTCCCGCGGATGCCGAGGAGTGGACTTACATATGGCGCGACCGGGAATCCATCACCCTGATCGTGTGCGGCT
GCGGTGTTGGGTCGGAGCAACTGCTTCAGACGCTGGCTGATCTTGTCTTTGGAGCGATAGGCATGTTCATTACCCGGGCC
GCAGAAATGGCACATGCAACGCTCCTCGATAGACTGAAGAAGGACGCCAAGAAGTATGTCCCGATTGTTGACGCTATTCT
GGAAGCCGCCTGTGCGGGTACACAACTCCTGGGCTACACGGACTGTCTGCTGGCGGCGGAAAACGCCCAGCTGCTGCAGT
GTCTAAATGAATTTAGTGGCCACTGTGGCTCGCTCTTTTGCTGCCTGGTTGTGGGACACCGTATCGCTGTGGCCACTGAG
GGCTGGTGGGATCTGGACACTCGGGACCGCGAGCTGCTGCTGTTTCTGTTGAATTCGTCGAGCACAATGCAACACGATGT
GCCGGTCTATTTGCCCGTGAAAAGTCCCCATATAGCCTACCGCTTTGTCAGTATTCCTGTGGCGTCGAACAGTGCTCTGT
GTGTCCTGTGTGGCGCTGAGAATGCCTCGTTCCGAGAGTTGCATGCACATGCCATGAGTGCCTACCGGAATGAAGGCAAC
CTTTTGGCAGCCGCCGAGCGATGCGTGCCGCGCAGTCTGCCCGAGCATCTGGAGATCGATGGAAATGTGCTGGCCATCAT
TCTAATTAACCGACACACGCGAAAAAGTCTCTTTACCAGAAATCTAAACCAGTCGGCGAGTGTCAAGCGCATCATTGTGG
GCGATCTACAGCGCTTGGACATTCTAAAGAAGTTTTTCGATCAAACTATGGATGCAGTCCACCAATTTGAAACCCAGTCG
TCCAGCAATAAAACTACGTTAGTGGACCAATATAGCTGCTCGGACTACCACAAGTGCTATGCCCATATGGATGAGCTGGG
CAATGTCATATTCTTGTTGTTTGTGGCAGCGGTGCCCTCGCACGCAATGAGATTCCTTGCGCAACGCATCCATGCTAATA
TACTCCAGGAGAAGTCAGTATGTTGGTGATACGATATTTCTTTCTAAGCCTAAAGACTGATTTTTGTTACTACTTTTCCA
TGAAATAATGTTTTTTTTTAAATGACATTATATTTAACGAACAATAGGGTACAACTCCTTTTCTAAAGTTAAGCGTAATT
TTTGACAAATCTTATATGTATATGCTACATACTAATATGGCTTTAATGA***CTTACAATTCCGTCCTTACAAAT***ACTATTTA
TATATTTTAAATATACAATATGTCCTT

44. l(2)35Bg

ATTGCCATATCTAACTTGTGAAATCAAAAATCGGTTCCGTTCGGTGCTTGAACTTTTGAGATATTCTAGAAACACATCCA
CA**ATGGAGAACTTTAAGGGTCTACAAAAATCCCTATACATATGGACGGACAGCGCCGACTTGGACAAGCGCGTGGAGCAA**
CTAAAGGCGGCCACTGGCGGCGATGTGGCTTTGGAAAATGTGCACCGCCTTTCGTTTTCTTCCTATGCCAACTCCAGTTT
CGACCTGATTGTGATCGAGTGCGCCCAGCTGACCGACAGTTACGTAAAACTGCTGCACATGCTGAAACCGAGTGGCAAAC
TGCATTTGGTGTCCTACATCGGTCCGGCGGCTAGTTTGCTGCAGGAGATCAAGCTATCCGGATTCATAAACTGTCGCGAG
GACTCTCCAGATGCTTTGACCGCCGAGAAGCCTGGCTACGAGACAGGATCCTCAGCCAGGTTGTCATTCGCAAAGAAGAA
CGCCAGTGCAGTCAATGTGTGGAAGATAAGCGGTGACGATGAGGAACTGATCGATGAGGAGGAGCTGCTGGACGAGGAAG
ACAAACAGAAGCCGGATCCGGCTGGTCTGCGTGTCTGTAGCACCACGGGCAAGCGCAAGGCGTGCAAAAACTGCTCCTGC
GGGCTGGCCGAGGAATTGGAGACTGAAAAACAGAGCCAGAAGGCCACCGAGAATGCCAAGTCAAGCTGTGGAAATTGTTA
TCTCGGCGACGCCTTCCGCTGCTCCACTTGCCCCTATTTGGGCATGCCCGCCTTTAAGCCCGGTGAGAAGGTGCAACTGG
CCGACAACCTCCTGAAATCCGACATTTAACTTAGGCTCACCATGGTATACCCAATCAATCACATTTCAAATAGTTTATTA
AGCTGACTTACTAACAAACAATAATGTCATAATGTAAATACAAAAATGCACCGCGTCATAAACACAGCATGTAGATTTAA
ATATCCAAGTGATCACATTTCGCATGGTGTACCCTTGATGAGATCGCGGTTCTGGCCCATCAAAATACGATAGTTGACGG
CCGAGGATCGAGTTCCTTGTACAAATATGTCGGCATCTTGTCGGACAGCACCCAAATAGTGCCTTCATTGTCGATTTTC

ATGTCATTTGGAAAGACCAGAGTGTGCGAATCGGAATCGATTAGCCCCTGGGTGTCCGGAGTGTATGGACGCTTGATGTT
CCAACAAGCAATGGCGTCTTTGTTCACCTGCGTATAGAAGATGACGCCGGTCTCGGGGTCAAAGACCTCGGCGGTGGACT
GGCCATTCATGCCACGGTCACCAACGTATTTAAAATCGTAATACGAATCGCCAGCGGTCACGTGTGATTCGTTTTGCAGC
ACGCGGTTCGAAACCTTGAACTCCTTGGTACTGGCCAGGGCATGGAAATAAATGTCCTTGGAGTGATCTGGATTCATTGG
GCCCACGGCCAGCC***CAAAAACGCCATCCGTCCACTGG***AAGTTCACGCCGCCCACATTGAAATCACCATGCAGAGGATCGA
AGTGGAAGAAGTTGTGCTTGACGCGGTAGGATTTGTCGTTCCTCAGGGAGTACACAATCACACCGTAGGCACCCAAATCC
GGAATGTAAGCAAAGGCATCTTGGCACTCCGAGCGGTCCGCATCCACAACCTAAATATTGATTAATTCGATCAGTTTGGA
ATTTAAATAATACGCCTATGCATACTCACAATATTCGCAAAGAACGAGTCCTCTTTGGTCTGATCAGCGGGAATGGTGAA
ACGGCGCAGTAAAGTATCTGTTTTCAAGTCGAAAACCAGGATTGAATTGGGCGTAATCTGCTTGGGACTGCCCAGGATGT
CGGCCAAGCCAGTATCGAGCACCCACAGGCGATCGCATACGTCGACTTGAATTCGGAAGGTGGAAATGACAGTGGAGTTG
TCCTTCAGCTGGATGCCGGCATCTTGAGCCTTGTCCGCGTCCAGACGTCCACCGGATGGTGTCTTTTGGTCCTGTGGCTG
CACATCGATGGGCAGCTTGTTGGCCTCCCAACTGGGATACGGATGCAGCTTTGGCGACTTCTCCGTCGAATTAATGTCGA
TGTAGTTGAGTGTGGCAGCAACACCAGCCTTCCATCTAAAGGGAAAACTTGTTTAGTTACTAATTACATTTAAACCTGAC
ACTGGCAAGTAACAAATTAATAACATTAAAAAGTACCATTCACCTGCGCAATTATTATGACTGATTGGATATAGTCTAAT
ATAAGCTATAGTTTTAATTTATATCTTAGCACTATCAGTTAAAACTTTGACGGCAACTATAGCTTAAATAATTATGCTGT
ATTATCAACAAAATATACTTTTTTTACGTATCTT

45. lethal(2)gl

GCACACTAGACCCACCGATCGCGACTTTGTGTGTGTTAAAATGACGCGCATATCGTTTTGGGCTAATAATATTGTTCACG
TTTTATAAAACAGATACACGGTTAAGCTCTGACCGGTCGTATTATTATCTGTGTGTTCTTGGTTGACACGTTTATTTCAT
TTATTCTCATTTAAAGCAACGATGTGAAGAACAGTGCGGGCGTTTCTCAACATAAGAATACATACAAACTTATGCGTGAT
GGTGAGCAAATAAAATAGTTCAAAAAATTACTCGGCCACCGCCTTAACCGTACAAACGGAAATACATAAAGTTTAGAATA
AATTCGGCCACAATTAATATACTTATTTATTCATCAAAACCAAACCACCACAAGAGCATAACCAATCATGTTAAAGTTTA
TCAGAGGAAAAGGGCAGCAGCCCAGTGCTGACAGACAACGCTACATCGTAAGTTTAGAATAAATACGGCCACAATTAGTA
TACATATTTATTCATCGAAACCAACCCACCACAAGAGCATAACCAATT**ATGTTAAAGTTTATCAGAGGAAAAGGGCAGCA**
GCCCAGTGCTGACAGACACCGCCTACAGAAGGACCTTTTTGCTTATCGTAAGACGGCACAGCATGGCTTTCCTCATAAGC
CTTCGGCTCTTGCGTATGATCCAGTTTTGAAACTTATGGCAATAGGGACGCAAACAGGGGCTTTAAAAGTTTTCGGTCAA
CCCGGAGTTGAATTGTACGGTCAGCATACTTTGTTAAACAATTCAGCATCGGAGCTTAATGTACAATTACTTGAATGGGT
GTATGGAACTGGTCGCATACTTTCGTTGACGGCAGCGAATCAATTAATTCTATGGGAGCCAGTTGGAGCAACGTTGCTGC
CAATCAAAACACTACCGTTTGACGGCAAACTTAAAAAAGTTTCATCGCTGTGCTGTTCTCTCAGTAAGGATCTGCTATGG
ATTGGAACAGAAGGTGGAAACATCTATCAACTGGATTTACATACATTTACCATTAAGGAGCCTGTAATTTACCATGACGT
TGTGCTAGAGCAGGTGCCACCAGCCTACAAGCTAAATCCTGGTGCAATTGAGTCAATCCGCCAACTTCCAAACTCCCCTA
GCAAACTTCTAGTTGCATACAATCGCGGCCTTTGTGTTTTGTGGGATTTTGAAAGCGCATCTGTCCAGCGAGCATACATA
GCCCCTGGACATGGACAGAGCGTTGGTCTTACAGTGAACTTCGAAGGATCTGAATTTACCTGGTACCACGCTGATGGTTC
ATACGCCACTTGGAGCATAGATAACCCAGAACCGCCGTCGAATGTTAATTATGTGCCTTATGGACCTGATCCATGCAAAA
GCATAAATCGACTGTACAAAGGCAAGCGAAGATCCAACGATGTAATTGTTTTTTCCGGCGGCATGCCACGGTCAGCATAT
GGTGATCACAATTGTGTGTCCGTTCACGCCAGCGATGGACACAAAGTGTGTCTTGACTTTACGTCTAAAGTGATTGACTT
TTTTGTGACCTTTGAAAATAATAGAGATGTCGCTGAAGTTCTTGTTGTACTACTTGAAGAGGAACTCTGCGCTTACGATC
TTACTGACCCTAATATTTGTGCTATCAAAGCGCCATATCTTCACTCTGTCCATGCATCAGCTGTCACTTGCAATTACCTT
GCTTCTGAAGTCGTACAGTCGGTATATGAAAGTATTTTAAGAGCTGGAGATGAACAAGACATTGACTATAGCAATATTAG
CTGGCCTATCACTGGCGGTACTCTCCCGGATAACTTAGAAGAATCTGTAGAAGAGGACGCGACTAAGCTTTATGAGATTT
TGTTAACTGGTCACGAAGATGGTTCTGTTAAATTTTGGGACTGCACTGGAGTGTTGCTTAAACCAATTTATAATTTTAAA
ACTAGCAGCATTTTTGGAAGTGAGTCAGACTTCCGAGATGACGCAGCTGCAGATATGAGTGCGGAACAAGTCGATGAAGG
AGAACCGCCATTTCGGAAATCAGGACTTTTTGATCCTTATTCAGATGACCCTCGTTTAGCAGTGAAGAAAATAGCATTCT
GCCCAAAAACCGGACAACTTATTGTTGGTGGCACAGCGGGCCAAATAGTTATAGCCGACTTCATAGACTTACCCGAAAAA
GTGTCTTTAAAATACATTTCAATGAATTTGGTCAGCGATCGTGATGGATTTGTGTGGAAGGGTCACGATCAGTTAAACGT
GCGATCGAACTTATTAGACGGAGAAGCAATTCCTACGACGGAACGTGGTGTAAATATATCGGGAGTACTGCAAGTTTTGC
CGCCAGCCAGCATAACATGCATGGCACTCGAAGCAAGCTGGGGCCTAGTATCTGGTGGGACTGCGCACGGCTTAGTTCTC
TTTGACTTCAAAAACTTTGTTCCAGTATTTCATCGCTGCACTTTAAACCCAAATGATCTTACTGGAGCAGGAGAGCAGCT
GTCTCGTCGAAAGTCTTTTAAGAAATCATTGAGGGAGTCATTTAGAAAGCTTCGCAAGGGTCGATCGACCAGGACCAACC
AGAGCAATCAAGTACCAACAACGCTGGAAGCAAGACCCGTCGAGAGGCAAATAGAGGCTCGTTGTGCAGATGACGGGCTA
GGATCCATGGTGCGATGTTTACTATTTGCCAAAACTTATGTTACTAATGTCAACATAACGTCGCCAACTTTGTGGTCAGC
AACAAATGCCAGTACAGTCTCGGTTTTCCTTCTGCATTTGCCACCAGCGCAGACCGCGGCAACTGCCGTCCCGTCGGCAA
GTGGCAATGCACCACCACACATGCCCCGCCGAATTTCTGCGCAGCTTGCTAAAGAAATACAATTAAAACATCGTGCTCCT
GTGGTGGGTATTTCTATTTTTGATCAGGCGGGTAGCCCTGTCGATCAGCTGAACGCCGGTGAAAACGGGAGTCCACCGCA
TCGTGTACTTATTGCTTCCGAGGAACAGTTCAAGGTGTTTTCACTTCCGCAACTAAAGCCGATTAACAAATATAAGCTTA
CCGCTAACGAAGGTGCTCGGATTCGCCGCATCCATTTTGGTTCGTTTAGTTGTCGCATATCCCCGGAAACACTGCAGAGT
ATGCACGGTTGTAGCCCAACTAAGTCCACGCGTTCACATGGCGATGGAGAGGCGGATCCTAATATCAGTGGAAGCTTGGC
TGTAAGTCGTGGAGATGTATATAACGAAACAGCATTGATATGTTTAACGAATATGGGCGATATCATGGTTTTATCAGTAC
CTGAATTAAAAAGACAGCTGAATGCCGCAGCAGTGCGACGGGAAGACATTAATGGAGTTTCGTCACTTTGCTTTACAAAC
TCTGGAGAAGCACTGTATATGATGTCTTCTTCTGAACTGCAGCGTATTGCTTTAGCCACGTCCAGAGTCGTGCAACCCAC
TGGCGTTGTTCCAGTAGAACCATTAGAAAATGAAGAGTCTGTGTTGGAAGAAAATGATGCAGAGAATAATAAGGAAACCT
ACGCATGTGATGAAGTTGTGAATACATATGAAATTAAAAATCCATCAGGCATTTCAATATGCACAAGGCCTGCAGAGGAA
AACGTTGGAAGAAATAGTGTTCAGCAAGTTAATGGAGTCAACATTTCAAATTCACCTAATCAAGCTAACGAGACTATCAG

CAGCTCTATTGGCGATATTACCGTTGACTCGGTGCGCGACCATTTAAATATGACGACCACCACTTTGTGTTCTATTAATA
CAGAGGAAACCATTGAAATACGACGGAAACGAGTACTAGTTCTGTTGTAATTAAATCTATAATTACAAACATTTCTCATG
AAAAAACGAACGGAGACAACAAAATAGGAACGCCAAAAACAGCGCCTGAAGAAAGCCAATTTTAACATTGACAGAAGCCG
TAACCTACTAATTATTTTATACCTTTACAGAAATACGACGCAAACGAGTACTAGTCCTGATGTAATTTAATCTATAATTA
CAAACATGTCTCACTAAAAATCGAACGGGGACAGCAAAATAGGAACGCAAAAAAATTAAATTAAATTAAACGCGATTTAC
ATACAAACAGAAATGACAGAATGATAATATAAAATATTCATTTTTTATTTGGCTTAAGCGATGTTGTTGTTCCAAAACCA
TATAATTATTTATGATTTTATGTAATGTTTTCATGTATTTTCGCGTAGTGACTTTATACCCTTTACCCGTATGATGAAAC
GAAACATGATATTTGTAGAAAGGTACAAAGGATAAAAGTTGACATTTAAGGCCGATTGTAAAATATTAAAACGCAACCTG
GATAACATGCCGATTAATTACTTCTGTTCGTCCGTATAAACACAAATA***CCGATAAATTGGTCCGTCCCCAT***TTAATAAAT
TGTATATTAAAATGGTCTTTATTTTGGATAAATAATTCAAATTATATAGGAACATTTGGGTTTTAAGGGATAGAAACAGC
GCTGGTGTCTTTCATTTCCGTTCGTATGACCGTTCAAAGATTATAAAAAAATTGTCGTGCCCACATTTTTTAAATTTCTT
TTATTTTCGGTTAAAATATTGTGATACAAAAAAATGCCCTTACAAAATTAGGACAACAACAATTTGCTTTATTATGATGA
ACAAATTAGGAGTACAAAAAATAGCCTGATGCAAGGATGGGGAGAAAAAAGACCTTGAAGTTCAAAAGAATTTGTTGTAT
CAAATCCTCAATAAGAAAACTACTTAAAATTAAAAGCGTGTTTCAAATTAATTGTAGAATATTTCGTAAAATGAAATTGC
AAACTTTTTTAGCACGAAATTACATGTATTTAATTCTTTGGCTAATACGTCAGTTAAAAAAGGCTACATTTTGATGATAC
TCACAAATGTAATTTTTGCATTCTACACAGGTGCAATTAAAAAATTATGGTTTTTATGAGTTTTAGAGGGAAGAAAATAT
GTCAATATGAGAAGTAAGTTAATGAGCCTTCAAGTACTTTAGTACATAATGTAATTTAATTTTAGAAATCCTACGGCTTT
ATATTCAATTGTAAAGTATTTTAAAATTCCTGACGATTTGTAGTAATTGTTTTGTTCTTAGCTTTTTTGCATTCTCCTCC
GTTACTCCGTTACTCGTATAGTAAAAGGTATAGTATATACAATAGTATTGTAGATTCGTTGAGAAGTATGTTACAGGCAG
AAGGAAGCTTTTCCGACCATATAAAGTATATAATTGAGTATTTAGCTTAGGATTATTAATAAAACATGTATTTTATTTAA
C

46. Oseg1

TTGTTGTGGTTGGTTGCCATGGAATCTGTGTGTAAACAACGCACCTGCATTTAGGAATTTCTACCAACTATGCAGGATTA
GCCAATTCGACTTCTGCAACA**ATGAGGGGTGTTCTCAAGTGGCTGGAACGCATAGAGTTTCCCAATAGCAAAGACAATGA**
GATCAGTGTCCACAATGTGTGCTACGCACCCGATGGCAGCCAATTGGTGGTGGCTGCGGGGGATCGGTTGCTCATCTACG
ATCCAAACGATGGCTCCTTGCTGAACACCTTGAAGGCGCACAAGGATACGGTCAATTGTGTGGCCTACTCGCGGGATGGC
AAGCGGTTCGCCAGCGGAGCCTCCGATAAAATGGTCATCGTGTGGTCACCTCAGCTTGAGGGACTGCTCAAGTATTCGCA
CGGTGACTCCATACAGTGCATGAGCTTCAATCCTGTTTCGCACCACCTGGCCTCGTGCTCCTTGTCGGATTTTGCCTTTT
GGTCGGCTGATCAGAAGGCGGTGCAAAAGTACAAGATATCCGCGCGTGTCAATGGCTGCTCGTGGACCAATGATGGACAG
TACCTGATCCTGGGCCTGGCCAATGGCACCATATCCATTAGGAACAAGTTGGGCGAGGAGAAGGGGAGAATCGATCGGCC
TGGTGGGCCCAACAGCAACGTCTTCAGTGTCCAATGTTGTCCGGCCAGCGGTCCTGGCAGTGTGGACACCATCGGTGTGG
TGGACTGGAGCCAAACGCTATCCTTTCACACTCTGAGCGGCCAGATGATTGGCAAGGAGCGAACCCTGGGCTTTGATCCC
TTGTGCCTGCAGTACTTTCCCAATGGGGAGTTCTGCCTGGTGGGCGGCTGCACTGGAGGTCTACACTTCTTCACCAGGGA
GGGCATTCGGCTGGGTACGTTGGGCGATACCTTTGACACCTGGATCTGGACAGTTGCCCTACATCCCAATGGTCAATCCT
ACACCATTGGGTGTCAAGATGGAACCCTGGCCTGCTTCAACATTGCGTCTAGTACCGTGCATGCCCTCTACCGCGAGCGC
TATGCCTTCCGGGAAAACATGTGCGACGTGATCATTCAGCACCTGATCTCTGGCCAAAAGGTGCGCATCAAGTGTCGAGA
TTTAGTTCAGAAAATAGCCATTTATCGGAATCGACTGGCGGTTCAGTTGCCGGAACGTGTGGTTCTCTATGAGCTGAGCT
CTGGAGAGGATCAACCCATGCACTATAAAGTGCGCGAGAAGATCCAGCAAAAGTTCGACTGCAGCTTGCTGGTTGTTTGT
GGCCGTCACATTGTCCTGTGCCAGGAGAAGCGTCTCCAATGTCTGGATTTCATGGGTGTCCTGCAGCGCGAGTGGATCAT
GGACTCGTTCATAAGATACATCAAAGTCACCGGTGGCCCCGTGGGAAGAGAAGGACTAATGGTGGGCTTGAAGAACGGCC
AAGTGTTCCGAATCTTTCTCAACAACTCACTACCACTGCTCATCACCACTGCGGTGTCAGCGGTTCGCTGCCTGGATATC
AATTCGAAGCGCAGCAAGATCGCAGTTGTGGACGATGTGGGTCGCTTGGTTGTGCGGGACGTAATCAACGATACAATTTT
GTACCAGGATACGGGTGTAAACAGCGTCACTTGGAACACGCACCTGGATTCGATGCTCTGCTATACCCACACAACAGGCG
GATTAAGCGTTCGCGTGGGGAATCTGCCGCCGAGGGCACCACAGAACATGCATGGCGTGGTGGTGGGGCTTTGCGGAGCC
ACTGCCTTCTGCCTGCGCGGAAATATTATGCACAATACGCCTTTGGCTCTCGGTTCCACCATGTGGCAGTTCATCGAAGC
TGGATTGTTTGACGAAGCCTACCAGGTCGCCTGCCTGGGCGTGACCACCAGCGACTGGGAGGGATTAGCTCAATCCGCTC
TGGAGGCACTGCACATAAATATCGCTCGCGATGCCTACGTCAAGGTGAGGAATCTTCCGTGGCTCAAACTCATCGGTGAG
CTACGAGATCAGCAACAGCGATCGGCGGTTCCTAAGGAGGTACTTCTCGCAGAGAATTGCGCCTTTGCCGGCAAGTTCAA
GGAGGCAGCTCGTCTATTTCTCAAATGCGGCCAGTCCTCAAGAGCACTTGAAATGTACACAGATCTACGTATGTTCGATT
TAGCCCAGGAGTATATTAAGGACGGCACCGACCAGGAGAAGAAGGAACTGGTACGCAAGCGAGCGGAATGGGCATATTCG
GTTAAGGAGCCACGAGCTGCTGCGGAACTATTGCTTTCAGCTGGCGAAAATCAGAAGGCCATTGATATTGTCGCAGAGCA
AGGATGGGCCGATGTGCTTTACGATATCGGACGACGACTGAGTCTTACGGAACGCGATTCCCTGGAATCGGTGGCCCAGA
ACCTAAAGACCCTCAAAGCTCTTCCCCTGGCTGCTGAGATTTTCAAGAAGCTGGGCGATGAAGCCCAGGTGGTGCAACTG
CACATCGAGGTACGCGACTGGCCGGAAGCATTTCGTTTGGCGGAATCGCTGCCGGAACTTCTGCCCACGGTGCACTACCA
GCATGGGCAATGGCTGGCCGAAACGGATCAGTTTATAGAGGCGCATCAGGCCTACATAAAGGCAGGACGCACCAAGGAAG
CCAATCGTTTGCTTAAGCAACTGAGCAATACGGCCATCGTGGAGGAGCGCTACTTGGATGCGAGCTACTTTTATTGGCTG
CTGGCCAAGCAACATTTAGATGTATATCACGCCAAGGAAGAACAAAATCCTGTTGATCACCACCTTACGGAGTACAAAAA
TCATTTACGTGTTGCCAAGGTTTATTACGCCTACAGTGTCATATACTCATATATGAAAGAACCATTCACCACACATTCCC
CAGTTAGCCTATTTAACGTAAGCCGCTTTATACTAAACGAAGTGGAGCACAAAGGTGTCCCCAAAGGTGTCAGCATGTTT
TCGGTTCTCTTCACTCTGGCCAAGCAAGCTAAGTTTTTGCAGGCCAACAAGTTATGCCTTCTTATTAACAAGAGACTGCA
GTCGTTGAAGCCACCAGCTGGAGTCCAAGAGCAGATTGATCTAAATTTTCTAAATAGCAAGGCTTGCAAAAGTGGCTTCA
ATGATCCGGAGGAGCTTTTGCCACTATGTTACAAGTGCTCCAACTACAGTCAACATTTGAATAGCAATTGCTGTCCCACT
TGCCGGCAGGATTATATTTTCTCTTTCGTTTCCTTCGAAATTCTACCCCTAGTACAATTTTATCCAGAAGTGGATATCTC

TGATGCCGAGGCGGAGCGCTTGCTGCTGGCTCCTGCAAAGCACGCCGAGGATTCGGATCCATTTAACGAGGATGTGGCCA
GTGCATTGCCTTTGAGCTTGGATCGCAATGGCCTGCGTGCCATAGATCCCAATCATGTGCTGATTCTTAAACGGAGGGGA
AAGAATGTGCGGAATGTGTACTATCGTAATATATTACCCGATCTTCAGGTTACCTATTGTCCTCAATGTCTGCTGCTTTT
CTATGCGGAGGACTTTGAGCTGCAAGTGCTGCAAAAAGGTCACTGTCCCTTTTGTCACACATCCTCCGAAAAGCTGATGG
AAGACTTTTGAAATCGACATAAGAAGTGTGTGCAAATATATAATT***TCCGTCCAAAACTGTACAAATAGG***AACTATACGCA
AATAAATACGGTGTTACGCCCAGGTTTCCTCCTG

47. PkaR2

AACTTTTTGTAATTTTTGCCTGACACGGAATCGGAATACAGAGGCTTCTGCTTGCGATATATTTCACCGTTCGCACTTGT
TTACGTTTGCCATTAAAATTTTTCAAAAAATCTAACGAGTAACAGCGGTTGAATCGCCGGTGGCAAAAATATAGAACCGT
GTGCCGTTGTGCGTTGGTGGATCGCTGGAAAATTAAATACGAATCCAGCAATCAAAATCTATTAACCGAAATACTTTCAA
AACATCAAACAACAGCGAAAGCGGCAATAAACACCAACATCAACGATCCAAAAATAAATGTAGAAAAACAAGGAAAGTCC
ATGAGAGTAACAAATTAAATTTGGTTTAGCCAAAACAACAAAAGCAAGCTAAGGAACAAGGCAAAAAAAGTCAGCGAAG
AAAGAGACGGAGGAGCAGGTGAAACAGGTGGACGCAGGAGACCCACCCACCCGAAAGAAACTATCACCACCAGCAGGTGA
CAGCAAAGCAGCAGAAAGTCAG**ATGTCGAGCGATTCGAGTCGAAGGATCCAGGTGCCCGAGGAGCTGAAGGAGGTGCTGC**
TGCAGTTCTCGATCTCCTTTCTGGTGGAACAACCGCCGGATGTGATCGATTACGCTGTGGAGTACTTCACCAAACTGCAG
TCGGAACGCCCGAGCGTCTCTCACACGGATCAAAGCACCGATGACCAGCTGAGCGTCAACTCACAGGATGCCGATGCGGA
ACCACCAGTGATGGCCAGCTCGCGTCGCAAATCAGTTTTCGCCGAGGCCTACGATCCGGAGGCGGATGACGACGATGATG
GCGCCACCGCCGTGTTCCCCAAGACAGATGAGCAGCGCGCCCGGCTGGTCGAGTCGGTGAAGAACGTCCTCCTCTTCCGA
TCCCTCGAGAAGGAGCAGATGAACCAAGTCCTGGATGCCATGTTCGAGCGAAAGGTTCAGCCGGGTGACTTTATCATCCG
CCAGGGTGACGACGGCGATAACTTTTATGTTATTGAATCTGGCGTCTACAAAGTCTATATTAATGACAAGCACATTAACA
CCTACAATCACACTGGACTCTTCGGTGAATTAGCGCTGCTCTACAATATGCCCAGAGCGGCCACCGTGCAGGCGGAAACG
AGTGGTCTACTTTGGGCCATGGATCGCCAGACCTTCCGCCGCATCCTCTTGAAGTCGGCCTTCAGGAAGCGGAAAATGTA
CGAGGAGCTGTTGAACAGCGTGCCCATGCTGAAGGCCTTGCAGAACTACGAACGCATGAATCTGGCGGATGCCTTGGTTT
CGAAGAGCTACGACAATGGCGAACGCATCATCAAACAGGGTGACGCCGCCGACGGCATGTACTTCATTGAGGAGGGAACG
GTGTCCGTGCGCATGGACCAGGACGACGCCGAGGTGGAGATCTCCCAGCTGGGCAAGGGACAGTACTTCGGCGAGCTGGC
GCTGGTGACACATCGACCCCGGGCAGCATCCGTCTACGCCACGGGCGGCGTCGTCAAGCTAGCATTCCTGGATGTTATTT
GCTTCGAACGCTTAATGGGAAAGTGTAGTGGGGGCATTCAGCGTAGCATTAGTGGATATCGCTATCTGGAACAGGATCTG
CGCGAATATTTCGGCAACTTGCCGCTTAATTAGCTTTGTAGTTGTAGAAGTAGTAGTAGTAATAGTTGGAATCCCATTTT
CATGGCCCTAATTGATTGATCAAAATTGATTTAAGTCTCAAAGATGGATTTAAAGTTTTTCGCAAGTTGCTAAGACATGC
GGTCATGAAAATGGGTCCTTTAATTAAAAAAAAATATACATTAAATTTATATAATTTAACTCAAAAA***TCCGTCCAGTCCT***
AGACACAGAGGCCTTTGAGCGCATCATGGGCTTCCTGACCGATGTACTCAAGCGCAATATCGTGATCTATGAGCAAATGT
TCACCGACATGGCCCGCCGTAATACGAATTTGTGAAGAAATCAGTTTGAAGCGTTCTTATTTGTATAGTTTATTTTATTC
CAAATTTGCAATTGTACCATATACCATCATTCAAATCAAACCAGAGCTATACAAAATTTAAGAAAGTAAATAAAGCCAGT
TTCGTTCCGACACAC

48. Ppcs

AATTCGTCCCTGTCCAACACTGCAATCAGCTGTTACCTGGCCAACGCGTTTTTTTTTTTTGGCCTGCACTTTGAACCCCG
AGGTTTCGTGCGAATAAAGATACGGATTTGAGTGCGCCTGTCCACATTCCCGCTCGTCCCGCTCCGCCATCCATCGCTGC
CTATCGCATTTTCGGTTCAGAATCCCGCGGACCTTGCTGCCGTGCACATCGCATCGCATTTCCACAGCTGCGTTTTTCAG
CATTTTCGTTCGCTCTCTCTCTCTCCGGTTTTTCAGAGCGCTCCGTTCCGCTCTCCCGCTCGCGCGCGCTCCCCTTAACG
GTTCCATTGATAAGCAGCGCCACCCGCAGCCCCTGCCCCGCCCCCCTGCCACGCATATTCCGCCCTTCGCATTTCGGAAA
CTGCAAAAAAAAACCAAACCAAAGCAAAGCAAAAGCAAATCAATCAATTAAGATAGTACGCCGAATAGTGCAGTGCGTGT
GTTAATTAATTTTGGTTGCCATTTCGCACATTCTGTATTTATTAATTGGCAGTGCATAGCTTCCGGCGGAAGAGGGCAAC
TTTCCGTTCATTTTCGAGCTTCCGTTTGTCGGCCAATTAGCAGCAGGTCGACAAAGAAAAAGCAAAAACAAGCGTAAAGG
ATAAGCCAAC**ATGACGCACTGGGAGGACTTCTACAACACGCACCTGCCGCCCGCGGACTTCGAGGACAATCGCTCCCTGC**
TCAAGGAGTTCTGCGAACGGCACAACAAGCTCCAGAATCGCATCGTCCTCGTCACGTCCGGTGGCACCACAGTTCCCCTG
GAGCACAATACGGTGCGGTTTGTGGACAATTTTAGTGCTGGAACGCGTGGCTCCGCCTCGGCGGAATACTTCCTCGACCA
CGACTATGCCGTGATTTTCATGCATCGCCACAAGTCGCTGGAGCCCTTCACGCGCCACTTCACCGGCCAGCAGTTCTTCG
ACATGCTGGACATTGCGGACAACAGCCAGAGTTCGACGATAGCCATCAAACCGGATTCGGTGGACGTGTTTGCTCCGGTG
CTGGCCAAATACAAGATAGCCCGCGAAACGCAGATGATCCTGTACGTGAACTTCACCAGCGTGGTGGACTACATGTGGCT
GCTCCGCGCCGCCTGCGAGTGCCTGGCCGCCTTCGAGGAGCGAGCCGTGCTCTACCTAGCCGCAGCCGTATCCGATTTCT
ACATACCCGAGGACATGATGCCCACCCACAAAATGCAATCAGGAGATGGAGCGCCGACGATTTCGCTGCAGCTGGTGCCA
AAAATGCTGGCCCCCCTCGCCAGTTTGTGGGTCCCGCACGCCTTTGTGGTGTCCTTTAAACTGGAAACGGACGAGAGCCT
ATTGATTGTGAAGGCACGTGACAGTCTCAACAAATACAAGCACAAGCTGGTCATTGCCAATGTGCTGCAGACACGCAAGC
ATCGCGTGGTGTTCGTCACGCCCACAGATTCCTACGAGCTGCATTTGACTCGCGAACAGACGCTGCAAGGACTCGAGATC
GAGGAGCCCATCGTGGCCGATGTGGTGCAAAAGCACGGCGAGTTCATCAGTAACGCTCAACAGCGCCAATGACCGTTGTC
GATGCGCCGGAGGCCACAGACGCAGCATCAGCAGCACCACCACCAACAACTGGCCACCGGTGACGAGGACGAGGATGGCG
GCGATCTTGATCGGGATCCATATTGCGTGACCAGCTCGCATTATTGCCGTGTTCTGAATCCGGGCACGCCAGGCTTTGCC
CGCAAATATTGAGCAGGTTATAGTCCATAGTGTATACTGGCATGTGGATGACAGACATTTACATTCGATTCATCCTTGCC
CTTGGACTTTGAGTTCCCGCCACAATTTAGCTATCTATTTATGGTCGCGCCTCATAATTGAGGTGGCACCGCTCCATTAG
TCCATTATCCTGTTGTTATTATTATTTTGGTATTAGTTAATGTATTGGCCCTATTGTTGTCCTGAGAAATGATGTCACTT
GTATGTATCCGCCATTCCCTTGGCTCCTTTTCTTATCTTCCTTTGTCTGCCGGCACGTTTATGTCATCGAGTATACGCAG
CGTGTTGCGCAGACATCAATAAGCGATTTTGATTTATGTTTGTGACAGAGCTTATGATGCTTATACATTTAATGAAGCAT

CGATCGATCCGGCGAACTGGAATTTAAATCCCACAACACGTGTGTTTTGATATCCTTAAATCGAAATAGCGACAGAGTGC
ATGCAATCGCAATTTGCCGGACGTTGTGTCCCTGGACAACTGGTGCTGTGTGTCAGTGGCAGGCGACGCGACAATTGATT
TGCTGTTTTGCATATATGGGGTGGAAAAGAGTGGCAGCTTTGCCCGTCTAATGTCCTTGTCGGTTTAAATTTATGGCGGC
CCAAGCCTTGGCGCCTGTCTATCCGAAGCAGGTCAAGTGCCACCGTGCCAAAGGTCAGCTTAAAGCACCTCAAATTGAGT
TTGAAAGCGGCGTAGCGAAAGCTAATTAACATCCCACACTCACACAGGCACTGAGTTATTTCTTGGCCAGGCTGAGATAG
ACTGGCGCTGGGCATGGGTTTCCACATCCGTAACTTCAGGGTCCTTTGCGGACGGCAGCCTTTCCACATTTTTATTGCAT
GTTATGTCGTTGCCACACGCCCGTCGGCCATAAGCTCCATGCCGCCCGCCTAAACGAACCTCTGGCGAAGGGTTGGCGGT
GGATTTGTATCCTCCTTTCATGGAGTTGTAA***CCACTTCCGTCCCGCCCGCATTT***GTGGTTTAATTTTTCCAGCCCACAAC
TATGCAGTGCTTTGTGTGTGGCCGGCAGTTTTGTTTTCGTTCGTTCTGTGGGCTGGGCAAAAAAAAAAATTCACATTTCT
TTCTGACGTCGGCTTTTTGTGGGTTTTGCTGGGATGGTTGGCTGGGTCTCTGCGTTGTTGGTGCCTGCATCAAATTAAAT
TTTATTGCGCTCGCACGCACCGCAAACACCGGCCTGGGAACAGATACCGAACATGGGAAACAATCACACATGTGGCTTAG
GATTTAAGCCAGGCTCCGCATTTCCTTCGTTTTCACTTTCCTGTTGCTCGGTTTTTATGGCCTCGCCTACCTTAGTGTGC
ACCCACATATCAAGATACCCCCCCCATGTGTACTTCTCCCCGCGTATCGATTGTGCATTAAAGTATAAACATAAAAATTC
AGTTTGCCAACATGAGTTGCGTACAATAAAATATGTATAACTAGCCAAGTGTGCGAACAAGATGGCAGCTGCGAATGGAT
GGTATTTGCTTTTACTGGCAAATGGTGTTTTTCTTTCTCAATGAGTACTAAGAGAGAATACTTAAGCATGCCAACCATAA
ATAAGCTACTTAAATACCAAATAAGTTTTCGAAACATACATGTAAATTCGAGAAGAACCTCTCCATATCCGTAACTTAGG
ACTGCATTTGATGAAAAGGTCAGTCCCTGATAAATTCTTAGTGAAATTCAAGATTTATTAAACCTAAGTACATGAAAGAA
TTTCAATAAAACAGAAAATCAAAAGTTCAACAACTCTTCAAGTCATCTACATATATGAAATCTATTCTCTATACATACAA
TACATGTACTATATTATCCAAGAGGGGAATAAAACCAAGTGAACCAATTCGTTAAAGTTTCAATATCCAATTTGGACCAA
CCGATAAGAACAAAATTAGAAAAAAATACAAACAAGCTATAATTAATGACGAATATATATATATATATATATGTATATGT
GTATATCTCGATGTATACCTTTAAATATAAAACGAAACAAAGGAATTTCCATTGAATATTTACTCTCTACAAGCATTTTG
CCCTCTAAGCTAGATACATAACTATTTTATTGTTGAAAACGAAAACAACTAAATCCAATTGTACGCAATGCAGTTTTGGT
CACTGCACCTGCCCTCTTTCTTACCGGAATCCATGAGCTGTTTAACTGTAAAACTGTTGTAAAATGTTTACCAAAAAATG
TACCATATGCCGTACTAAGGTCTAGTATCCATATACATATACATCTACAAAGATACATCAATATATATATATATTTGAATGT
GTATTAAACTGGAAATGAAATAGCTTTAAATGTATAGACAAACGAGTGTGTGTT

49. RecQ5

ATCTCTAAACCAGCTGTTTTGGCGGCAACCAGTTTTGGTCTGAAGCAAAACAATGTTTATTTAAATAAAACGCAATTTAA
ATAACAGCCCAAGT**ATGGCGCATGAAAGCGCTGTGCACGAGGCGCTGAAAAAGCATTTTGGCCACTCGAAATTCAAATCG**
GATCTACAGGAAAAAGCCGTGAAGTGCGCTGTTAAAAAAAAGCAGGATGTGTATGTGTCGATGCCCACGGGCTCGGGTAA
ATCCCTGTGCTTCCAATTGCCTGGTTTGATGTCAGAGAATCAAATAACCATTGTTTTTTCTCCGCTTTTGGCTTTGATTA
AAGATCAGATAGATCATTTAACCAAACTGAAAGTGCCGGCGGATTCTTTGAACTCCAAGATGAGCACCAAAGAAAGGGAT
CGTGTTATAATGGACTTAAAGGCCGTGAGAACCAACCTGAAGTTTCTCTACATAACCCCGGAACAGGCCGCCACCAAGTT
TTTTCAAGACCTGCTGCAAACCCTCCATAAACACAATAAGCTTGCCTACTTTGCAGTCGACGAGGCGCATTGTGTTAGCC
AATGGGGACATGACTTTCGACCGGACTATCTAAAGCTGGGGGAGCTGCGTTCCAAATATTCCGACGTCATCTGGTTGGCT
TTGACTGCCACAGCTTCAAGGGAAGTCAAGGAGGATATATATAAGCAGTTGCGCCTCCACCAACCCGTGGCTCAATTTAG
CACTCCTAGCTTCAGGAAAAACCTCTTCTATGACATTGTTTACAAGAATTCTATCGAAGATGACTTTCAGCACCTGGCAG
ATTTCGCCAGACACTGCTTGGGCAATCCGAAGGAGTTTAAGGATACACCCAAGCCACAAAGAGGCTGCGGAATCGTATAT
TGCCGGACTCGAGACCAAGTGGAGCGCATGGCGATAGGGGTAACTAAACAGGGAATCGGAGCAGTAGCCTATCACGCTGG
TCTGAAAACAGGAGAACGTACTGAAGTCCAGGAAGCGTGGATGCGGGGTGATCAGCCCATTATATGTGCAACCAATAGTT
TTGGAATGGGTGTGGACAAACCAAGTGTGCGCTTCGTTATCCACTGGGATGTACCCCAAAATGTGGCTGCTTATTATCAA
GAATCTGGTCGCGCTGGACGCGATGGCTTGCAGTCATATTGTAGATTATACTACGGACGGGAAGACGTCCGGAGTATACG
TTTCCTTCTTCAAAACGATGCCCACAGAGCCAGGGGTCGTGGTGACAAAGAGCTGCTTACGGAGAGAGCTATCAAACAAT
TTGAGAAGATCACGGAATTCTGCGAGCGAACAACCTGCAGGCATAAACTCTTTTCGGATTTCTTTGGAGATCCAACTCCA
GATTGTAGTGGGCAATGCGATGTCTGCAAGCGTCCCAAGAAAGCGGAAAAAGCACTGGAGATTTTTCATCGATTATGTAT
GGATGATGCCTTTAAGTCGCACATTTCGCTACAGGATTGCGCAGATCTGTATGAGGGTGAGTTTTAAAACTTCGTTATCT
TTTTAGCTATTTCTGATCTTAAAGCCATATTTTTATACCCGTTACTCGTAGAGTAAAAGGCTATACTAGATTCGATGAAA
AGTATGTAACAGGTAGAAGGAAGCGTTTATGTATGGATATTCTTTACCAGGATTACTATCCGAGTCGATCTGGACAT***CCG***
TTTGTCCGTCCATATGAACGTCAAGGTCTAAGGACCTAGCGGGATCTCTGGAATGATTTTTTTTATTTAATTATATGTCA
AAAAGTATCTTATAGTCGATTAGGTCGAGTATAGCGCTCTCTCTTGTTTTTATATCCTACTAGGCGGCCGACCAGGTATA
AAGCGTGCTGCCCAGGAGTACGCAGGCGGTGAATCTGGCTCAGATGACGATTCTGGTCAGAGTCACTCATCAATGGCCAA
GAGAGCCAAAAAGGAATCACAGGATTTCATTAAGCAGCAGTTTAATCTTCGAAAGCAAATTAGTGCAGCTAGGCAGTTGG
AACAGGAAACAATTGCTCAAATATCTCGCGTTCGAATGGCAGAAGCCACTGAAAAGAAAATCGCAGGTCTTCAGGCCACT
CATCGGGAAAAATATTTGACAGCTTTAATCGACGCACTCAAAGCCAATGTAGACAAGTGCAAAGATGAACCAGGGCAGCA
GCCAAAAAGTGTATTAAAATACAATGACTATGAGGCAATGTCAGTAAATATGGAATATGACGTGTTTCGCCAGAATAAGG
TGGCCAATATGTACCGCCATGCCTTAGTTAAAGAGATATCCACAATCAAGAAACTCACAGAACAGACTAAATTATTGCCT
CTTTTGGTGGACTATATTCCGAAGCCTGAAACAAGTTCTAAAGGTGCTTGGACAGGTGGAAGCGTGGCGTACTTTGAGCG
GAAACTCAAAGAACTAGAAGAACAAAGGCCACAAAGCCTTAAGGAAGTTCCAAAAGCCCTAAAAGACAGAAAAGGTTTTA
AACAGGAAAATAGCAAGCAGACTTCAATTTCCTCTTTTTTTAAAAAAGAAATTAAAGAAGAACCAATAGACTCGCCAATT
CAGGAAGAACGTGTAAATATTAAAAGTGAACAGATGGATTTAGAAGAAAGCACACCTAAAGATATTGCTTCCAATCATGT
TAAGGAGGAATCCGTGACCCCAGAAAGTTCTTCTTCTGATCGTGAGCTTGAACTCCAGCCCGACAGCATAATTGGAGAAA
GGAAATTTAAATTGATGTCAAATGGTGATGGAAGCTATGACACTCACCGCAGGAAAAGAGTTTCGCATGAAACTCAACCC
AAAGAGAAGGAGTCAATTAAAACAAAGTCCAGTATGCAGCATCTTTTTGGTTCTTTTAAAAGCGAGTCAGAGGAGTCTAA
CCAATCTTTGAATGGTTTTAAAACTGCTCGACAAATGCTCGAAGAAAATAATTCAAAACTCGAGGGCGTACCTGAAAAAT

TACCTATTAAGGATGGTGAAAAGAGAGTTTTAGAAAACAGGGCCGATTCTATTGGATTTACTTCAGCCAGAGAAATGTTA
GAGAGGAGCAAACAAAGGGAAGGGGACGATAAGTCAAGCAACAAACTCGAAAAACTGGGATTTATTTCAGCCCGGGAAAT
GCTGGAGAAAAACAAACTTAACGAAAGATGTTTGGACAAAAAAGACGTTAAGGAAAAGAAATTAGAAAGTAGGGAAACCA
AAAGATCGCTACAAAACAACGAATTACAACATAAGCAAGAGGAAGACCGTAAAGACAAAGCATCCCACAGGAAAACGTCA
AAGGAATCGTTATCGAACTTGAAGAAGGAAGGATCCGACTCCAGAGTTTTGCAAAAGGATACAAAACCAGAAACCAGAAA
CCATCAAAAAACAGATGTCAGCAAAAATGTGGTGCAGTGGCTCAATCCGTATTATAAACGAAAAATAGCCACCAAAGAGC
TTTTCAAAGCTTTGGCAAAATTGCTCACTCAGCGCATTTGTGATGGAAGCTTAGGAGATGGCGATGCTGGAAAGTGCTAT
ATAAAGGAAACTTTTCATGGCCTGCAGATGATAAACAACGATCAGGACATTGAAAAATATTTCATAAAATCCAAGTGACT
AAAAATCTCCTAATGATTTTATCACGACATATTGTTTCGCCTCGTTAAAAGTTGCATCATCTTTTTTTTTCGTTAGTTAC
TATTTTTGTATATAATAAGCGCATTAATTATCAATAATACATTTAAATAAGACGTTTTT

50. sbr

**GATAGACCTGTCAGAAATTAAGAAAACAATATCAACATCAAGCGAATATACTTCGCTCGCTACATATTTCTAGTTAGTTT
TCGTAACTTTTTCGCTTTGGTGGCAATTAAATCAAATTTCCTCGAAGAATAACTTGTAAAAAGGTCGCAAACATCCGTCC
AGTTTCTGCCAACCATCGGCCACTGGCTATCGAAAGAAGATCGCTAGTGAATTAAATTTCACAAAATTGCGTTGTACTTT
GGCCGTGGAAATTTGCCAAAAAACAGCAGCTACTTTGCTGCTATATTAGCAAATAGGTTCAGGAAGTCGAGAAAAAATTTT
TCCAGAAGTTGGCAGCAGTTTGTGTGAATGCAAAAAATACATCGCACATAGTTTTTTGCCCTCAAGACAGTTTTCTCAGT
AATAACAACAGCACCCACACAAACACACAACACCACTCAGCTAAAAACGTGCTGAAATTCGCATATCTTTTTGTTGCATA
CGTTTTTGAGTGAATATTAGTAAGATGCCCAAACGCGGCGGTGGCAGTAGCCAGCGGTACAACAACAACGTTGGAAATGG
CGGCGGACGTTACAACGCTCCCGAGGATTTCGATGATTTTGATGTGGAGGATCGCCAGCGACGCAAGGATCGAAACAAGC
GGCGCGTCAGCTTTAAGCCCTCCCAATGTCTACATAACAAAAAGGACATCAAGCTGCGACCCGAAGATTTGCGTCGATGG
GACGAGGATGATGACATGAGCGACATGACCACGGCCGTTAAGGATAGACCCACCTCCCGACGTCGGGGATCGCCCATTCC
GCGCGGCAAGTTCGGCAAACTGATGCCCAACAGCTTTGGCTGGTACCAAGTCACGTTACAAAACGCCCAGATATACGAAA
AGGAAACACTCTTGAGTGCTCTATTGGCAGCGATGTCGCCACATGTCTTTATTCCTCAATATTGGCGAGTGGAGCGAAAC
TGCGTAATCTTCTTTACGGACGACTACGAGGCAGCCGAACGCATTCAACATCTGGGCAAGAATGGCCATCTTCCAGATGG
CTATCGTCTGATGCCACGAGTACGCAGCGGTATACCACTAGTGGCCATCGACGATGCCTTCAAGGAGAAGATGAAGGTCA
CAATGGCCAAGCGTTACAATATTCAAACCAAGGCGCTGGATCTTTCCCGTTTTCATGCAGATCCGGATCTTAAGCAAGTT
TTCTGCCCACTCTTTCGTCAGAATGTGATGGGCGCTGCCATTGACATTATGTGCGACAATATACCCGATTTGGAGGCACT
TAACCTGAATGACAACTCCATTAGCAGCATGGAGGCGTTTAAGGGTGTGGAGAAACGCTTACCGAACCTCAAGATTCTCT
ATTTGGGGGATAACAAGATACCATCTTTGGCCCACCTTGTAGTGCTTCGCAATCTGTCCATCTTGGAACTCGTTTTAAAG
AACAATCCCTGCCGTTCCCGCTACAAGGATTCCCAGCAGTTTATCAGCGAAGTACGTCGCAAGTTTCCCAAACTGGTTAA
GTTGGACGGAGAGACCCTGGAGCCGCAAATCACATTTGATCTATCCGAGCAGGGACGTCTTCTCGAAACGAAGGCATCCT
ATCTGTGCGACGTCGCTGGTGCCGAGGTGGTGCGCCAGTTCCTGGACCAGTACTTCCGCATATTTGACTCGGGCAATCGG
CAAGCTCTGCTAGATGCCTACCATGAGAAAGCGATGCTCTCCATATCAATGCCTTCGGCCAGTCAGGCGGGCAGATTGAA
CAGTTTCTGGAAGTTCAATCGCAATCTCCGGCGCTTGTTAAACGGCGAAGAGAATCGCACCCGAAACTTGAAGTACGGAC
GCCTGGCATGTGTTTCCACATTGGATGAATGGCCAAAAACGCAGCACGACCGACGCACCTTCACCGTCGACCTGACCATC
TACAATACTTCAATGATGGTTTTCACCGTGACGGGATTATTCAAAGAGCTGAACGACGAGACCAACAATCCCGCCTCCAT
GGAATTATATGACGTTCGCCACTTTGCCCGCACCTACGTGGTGGTGCCACAGAATAATGGCTTTTGTATCCGCAACGAGA
CGATCTTCATCACAAACGCTACGCACGAGCAGGTGCGAGAGTTCAAGCGATCGCAGCACCAGCCTGCTCCCGGAGCTATG
CCCTCCACTTCCAGTGCAGTGACCAGTCCTCAGGCCGGGGCAGCGGCGGGTCTGCAGGGTCGTCTGAATGCGTTGGGCGT
GGCCACTGGACCGGTGGCTATACTATCAGGAGATCCGTTGGCGGCCACCGCACCGGTTAACAGCGGCAGTGCCGCCATAT
CGACAACAGCAGTGGCACCTGGCGCCCAGGATGAGAGCACTAAAATGCAAATGATTGAAGCCATGAGCGCCCAAAGCCAA
ATGAATGTGATCTGGAGTCGGAAATGCCTGGAGGAAACGAATTGGGACTTTAACCATGCCGCCTTTGTGTTCGAGAAACT
ATTCAAGGAAAACAAAATACCGCCTGAGGCTTTTATGAAGTAA**ATCGCATAGGAGTTTCCGTAGGACAGAGCCGCGTGCC
ACATCCACATAATCGAATGCTGTTTTTTTTTTTTTGGTTTTGTAATTAAATTTTAAAATTATTAGAGAAACCTCTATATA
ATAATAATAATTAATATTATTAAGCTGCGAAGTTGTGTGCACATTCGGGCAGTAGCAATTATTATCCCAGCACTGCGGGC
AATGTGCATCAACGATCACAGTTCTTCGATAGATTAGTTTAGCTCTCTTTA***AGTTCCGTCCGCAGATCCGCTGG***TCTACA
TTGAGGCCAGGACGAGTCTGCGAACGGTAGTCCCTTTAGAGTTAAAGTTGTTTTAGATTCCTAAGCCAAACACCTTCAAA
CACACACAACACGACAACACTATTAAACGTAATGCAACAATGTTGTCGAATCGAAAGAAACCCAATTTTTATTTTAATAT
ATACGACGATACCGAAACCAAGGCGATGGTCGAAAAGCATGGATCGCGCCAATAATTCTATATTCCCCGCTTTCCCAGAT
CCTCGACTCGCCTTACATTTTGTATACAAATACCATAGAATAAAAAGAAACATTTTGACCACTGTAAAAATTTTGTAACG
ACTCGGAAACCAAAATTACCTTTATTTCTTAATAGAAAAAAATTATTCGATTTACAATACACGCTTAGCCGTAAATTCAA
TTGAATTTAAGTGTAAGATATATAAAAATTATATACATTAAGACAAAAGTGTAGATTCATAAATAAATTTTTCAGAATAG

51. SPoCk

GCACTTCCATTCGAAAGCCTGACGATGTGCCATCTTCCGGGCCGTTGTTGACGCGGTGAATCGAGGCGGAAAAATCGATT
GTGCTCGTGTGATTATTTTTGTTTTGATTTGCGGCCTCCCGCTCTCGTAATCCTCGCGCGGCCGCGGCCACATGACTCAT
GCCAGGCAGGAACA**ATGAAAAATCACAATAAGTACGTCAAATTGCCCCAAGACCTCGATCTGAGTTTCCACAGTGATTTG
GACCTGCTGCCGGATACGGCAGCCGACATCGACGTAGAACTAGACCCGGCTGCGGACACGGATGTGGCCGCGCTCAGTGT
GAACGTGCCCGCGTCTCTGGGCCTGGACGAGGACGACGACGACGAGGTCATTATCGCCCCCGGCGGCGGAGGCCTTCGAC
TAGCTCCCACGGCGGCGGAGGCAGTGGCGGCACACAGGCAGCATCATCAGCAGCACCAGCAGTGCAAGCAGCTAAAGAAC
CAGCTGGAGCAGAAGCAGATTCTGCCCAAGAGCAAGATCTGCGGACTAGGCAGCGACAGCATCGTCGAGACGGGCCTTAC
CCTCACACCTGAAATGCTGTTGTCCACCTCGGAATCATCGACCCACAGTGCCTCCGAGGTCGCCGGACGACTGCAGGTCG
ACGTGCGTACCGGCCTCAAATGGACAGAGGCCAAGTATCGTGCCAAGATCATCGGGCATAACGAGCTGCTGCTCGTGGCG**

GAGGATCCGACATGGAAAAAGTACATTGAGCAGTTTAGGAACCCGCTAATTCTGCTGCTTCTTGGTTCTGCTCTGGTGTC
CGTAATCATGAAGCAGTTTGACGACGCCGTGAGCATAACCATTGCCATCCTAATTGTGGTCACGGTGGCCTTTATCCAGG
AGTACCGCTCCGAGAAGAGTCTGGAGGAGCTTAAGAAGCTGGTGCCCCCTGAATGCCACTGCCTGCGGGAAGGTCGTCTA
GACACATTCCTGGCACGCGAACTGGTTCCCGGGGACATAGTGCACCTCAACGTGGGCGACCGGGTGCCGGCCGACGTGCG
TCTTTTCGAAGCTGTGGACCTATCTATCGACGAGAGCAGTTTTACCGGCGAAACAGAGCCGGCGCGCAAAATTACTGATG
TTTTGCTTAACAATACGAACGTAAAGGACCACAGCAACATGAAGAACATTGCCTTCATGGGCACTCTGGTGCGATGTGGC
AACGGGAAGGGCATAGTTGTCAGCACTGGGGAGCGCAGTGAGTTCGGTGAGGTCTTCAAAATGATGCAGGCAGAGGAGGC
TCCGAAGACGCCACTGCAGAAGTCGATGGACATTCTAGGCGCTCAGTTGAGCTTCTACTCCTTCCTGATTATCGGTGTCA
TCATGCTGCTGGGCTGGCTACAAGGTAAGCCGCTTTCGGAAATGTTCAATATCAGCGTTTCACTGGCCGTTGCGGCCATC
CCCGAGGGCCTTCCTATCGTGGTAACTGTGACTCTAGCGCTTGGCGTAATGCGGATGGCTAAACGAAATTCAATCGTTAA
AAAGCTGCCTACTGTTGAGACTTTGGGCTGCGTTAACGTTATCTGCTCTGATAAGACCGGAACTTTGACCAAGAACGAGA
TGACGGCCACGATCATCATCACTTCCGACGGATACATGGCGGACGTTACCGGTGCCGGTTACAATGATCAGGGTGAAATC
CACATACGGCATTGCAACAACGTGGAGATGGCTAAGACCAATATTACAAACCTCCTTGAAATCGGGGCGGTCTGCAATAA
CGCTTACATACAAAATGGCACCCTCCTAGGACAACCCACTGAAGGAGCCCTTGTGGCGGTAGCCATGAAGAACGGAATGT
ACGCCACAGCTGAGAACTACGTTCGCATCCAGGAGTATCCCTTCAGCTCGGAGCAGAAGATGATGGCTGTAAAGTGCATC
CACAAGTACAACAACAACAAGGAAGAGATTTTCTTCGCCAAGGGGGCTCTAGAGACCCTGTTACCGCAGTGCACCAAGTA
TCAGTTTGGTACCCAGACGGTACCACTTACCAAGCAGAACGAGGCAGAGTTCCTAGCCGAGGCGTACGAGATCGGGCGCA
AGGGCCTTCGCGTGTTGGCCCTGGCAAAGGGCCGGTCCATGCAAGATCTGATCTACTGCGGCCTAGTCGGCATCACTGAC
CCACCAAGGCCCCTTGTTCGAGAGTCTATTGAAATGTTGATGCAGAGCGGAGTGCGTGTTAAAATGGTGACTGGAGATGC
CCAGGAAACGGCCTTGGCCATTGCGAATCTCATCGGTATCGATACAATCCATCACCAGACGCTTTCCGGTCAGGAAATGG
ATCAAATGAACGAGCACCAACTGGACAAGGTAGCCAACAACGTGAGCGTATTCTACCGCGTATCGCCACGCCACAAACTG
GAGATAGTCAAGTCCTTGCAGCGCAGTGGCAACATAGTCGGCATGACGGGCGACGGGGTGAACGACGGAGTGGCCCTGAA
GAAGGCGGACATCGGCATAGCCATGGGAAAGAACGGGACAGATGTGTGTAAGGAGGCGGCTGATATGATCCTGGTCAATG
ATGATTTCCACACCATAATCGCCGCCATCGAGGAGGGCAAGGGCATATTCTACAACATTCGAAACTTCGTGCGCTTTCAG
CTTAGCACATCAATAGCTGCCCTGGCCCTGATTGCCCTGGCCACTCTGATGGACATTGCTAACCCGCTAAATGCCATGCA
GATTTTGTGGATCAACATCATAATGGACGGTCCGCCCGCACAGTCTCTGGGCGTGGAGCCCGTCGACCACGATGTGCTCA
AACAGAAACCACGGAACGTGAAACAGCCAATGATCACAAAGTCAGTGGTGGTGAACGTTCTGCTTAGTGCCAGTATAATT
GTACTGGGCACGCTGTGGGTGTTCCAGCGCGAAATGGCCGACGGGACGCTGGGAAAGACCAAACGGACACAACGATGAC
CTTTACTTGTTTCGTGTTTTTCGACATGTTTAACGCCCTGTCTTGCCGCTCGCAGACAAAGAGTGTCTTTACCATCGGAC
TCACTACCAATCGAATGTTCTTGCTGGCCGTCGCTTTTTCGATCATTGGTCAAATGCTCGTTGTCTACTTTCCGCCGCTG
CAAATGGTTTTCCAGACGGAAGCTCTTACGCCGTATGACATATTCTTCCTGGTCTCCCTAACCTCATCTGTGCTGGTTGT
TTCAGAGATAAAGAAATGGTTCGAGCGTACCATGGAGCGCAAGATGTACAGCACCCGCTCCGAGCTGGATTTTGTATGAC
AAAACGCAAGCGCTAGAGAAGATTTGCACCCCAAAACCGAGTAAATTAAATAACTAACAAAAAAATCCATTAAAAAAGTA
AAACTATGCCAAAACAAGCGAGGAGCGCGGAGAAGTGCAGTTCGAAAGAAAAGCCGAAAATAAGAGCGACGGACCAAGGA
TGTGTGTGTTTAATTTTACATTTGACTTGACCAATTAAGAATGATTTAATTGTTATTGTGGGTTTTCAACCAGATTGCAC
AGCAGGGATTTTCATAGATTTGAAATCCTACTCGTTCTTCCCCACAGAGAAGCAACCGGATTATAAACAAGTGACCACTT
ACTATAATCCGAGTATGTTCACCGGGAAAGTGCATTACCACTGGGTCACAGTCAACATACATAAAAATAAAACGGATGCG
AGACAAAAAAGGCATGAAATGGACAAAGCTAAGCGAGGCGTAGTAACGTAAAACATGGCTTTTTTTTGGTATTATTGATA
AATAATTTATTCGGTATTTGAAAGCAAGCATTCAATTTGTGTAAAATTGAGGCAAATCAAAACTCTAGCGTGCTATCAAA
ATAAAACAATTTTTGACGAACTCATCCCAAATCAACTTTAAAAGGAGAGTTCAGATCAACAAAAGATTTTTGTATGTAAT
CAAAAATGCTATAATTTCAGCATACTTTGTGGGCGTTGATGTGATGCTTTGATAATTAAGTTCATGATTAATGGGCTTAC
CAAAACGAAGTAATGCACGAAGCAAATGAGAAATACGAAGCAATCAGCACCGCGTTTACTCGAATGTAAAGAAATTTTCG
CTCATATACTTAAATAAAAAATAAATTCATAGGAAAGCATGGTTCTTTGTACTATTCTGTTGTTTGTTGTTTAAATTGAA
CTTTGATGCAATAGATTACTAAACGGCAAAAGAAATTTACAACTAAAGCTTATCCAGATTGTAGTCAAAAGTATACATCT
CAATAAAAAATTAATGAATTTTGAAAAAATAAACAAATTCTATAGATTACCAATACTTGGGTACCCCTTATAAATTAACT
TAAATATCATTGACATAAAATTCATATCATACCAACTCAGAGTTCACTGTTGCACTGGAAGCATATCACAATTTGTCCAT
GTTTTTATACCCGTTATTAAAAAAGTAAAAGGCAATTCTGTATTCGTAGAAATGTAAACCCTATCAAAGAATATATATTG
TTGCTCA***CTCTGTCCGTCCGTATGTCTATC***CGTATAAATGTAGAAATCTTGGCAGCTATAAAAGCTAGAGGTTAGGCATG
TAATTTTCCAGACCACCACAAGCGCGGCCCATAACTGCCACGGCTTCATTTTCTAGAAATGATTTTATATTTTTAAGTTA
TAAGTCTTGTCAATTCCCAATGATATATCCAAGTTTTTTTTTTTAATTTTGATATTTTTTTTTATAAATTTTATCGATAA
TTAAAAACAATTTTGAAATACTTCCCTGGCACTCCCACTAGTCGAGTAACGGGTATTTATAGTCGAGATATTGGACTTTA
GCGTTTTCATTTTTTTATTTGAATGAGCTTGTATTCAACATTTTTCACAAAATTTCAACTTGGTTATAGCAATTTATGCG
CACCCTGCAGATCGCATTTAAAGGACCAATCGTTAAATACTTCATTTGATCACACACATTAAGATCAGCTTACGCTCAAC
AAAAACGCACTCATATCATGGCAAAAAAAAAAAAAATGAAAAAACTTAAAAATACAAAAAAAAAAGAAGAAAAACATGGAA
AAGTAATTGATGAGAGCTCGTTCAACAGATCAAGCAATTGAAGGTTCATAATGATATCAGCGCATAAAAGCTCACTAAAA
GTACATAAGCAGAAAACCAGAGACACACAGGCCCACACACGTTCAGTCACGTCTCATCCACAATGGTAACAATAATTAGA
AAAATAAGGGTATCAATATAGAATAAAAAATATGAATGTCTGGAGGATTAAATAAAATGCACCAAATTAAATAAATATGT
TAGCCAAAGCAGGTATACATTATGGAATTTAAACTGAACTCTACTCAATGTTATGTGTAAGCAAAGTCCGAAATTCGACT
TATAATGAATGATGGTACTAAACGAATTCTTAACCACAGAAATATTATTGATCGTCTATTAATATATATGTAAGTTTAAA
TGTCTAACTAGACCGCCTAGTTTAAGAGCTCCGAAAAATATGTTGCCTAAATTCACCATTTACTAAAATAGTTTAGTTTG
GTGCAAAGAAATGTACAATTGATGACAATGGTACGAGTTCTTAAAATCCATTTTTAAAAACGCTTAACCTATAAAAATTA
ACTATAACTATCTGTGCTATTAAACTTAAGCTATAAAATATAGATAAATACCAAGAGTAATTCAAAATACATTCATAAAT
ACTTCGAGAATAATAAGAACCATGAGAAATGGCATAAAGTAAATTGAACCATCATCGTTTTGTTTTACTAAATCTATCCT
CTACAAGTGTATATCATTTTATAAGTTTAACAATATTGGACAAGATCTTAGGCATAAAATGAATTTTCCAATGGCAACC

ATACATTTAATATTAATCTGCGGAATATCGTG

52. ttk

AGTTCGTGCCGTGTGATCAAAGCTTTCGGTAAGGCTTCGGCTCGGCTTTGTGTATCCACCTTAGCGGCTCTCTGTGGTGC
TCGTTCTGCCTCTCTCTCTCTCTCGGTTTTGGGGCTCAGCTCAGGGACAACAAATGCGGTAAAATGGCTTCTGCGTCGAC
GTCGACATCCCTGCCGTTTCAGTTCTGATTGGAGGTTCGTGCGCGGCGTTTCCTCAGCTGATTTCTTTGCCTTTTGCCTT
TTCGCAACCCGCTTTCGGCTCCGTGTTCGCCTCGGTTGCCAATTACGTGTAACCAACAACAAGAGAAGAAACAGAAACAA
CAACAAGTACTTTCGAGTGTTGCAAGCCTTCTTCCGCCGAAGAAACCGCAAAACGGATTACCAAGAATATTTGTAAAATT
GCACGCCCTGGAATATATGTTTTAGCAGCTGTCAGGCCCCAAGGAGTAAGAATCCGAACCCAGGACAACGAAAGGACCTT
TGATGCCCCACTTGCATGAGGACATAGATCGCGATCGCACACACTCGGACATCGCTTAGTGACGCAACCACAGCCTCCCA
ATGAAGATGGCATCTCAACGCTTCTGCCTGCGGTGGAACAACCACCAGAGCAACCTTCTGTCCGTCTTCGACCAGCTGTT
GCACGCAGAAACCTTCACAGATGTGACGCTGGCCGTCGAGGGGCAACACCTGAAGGCACACAAGATGGTGCTATCCGCCT
GCAGTCCCTACTTTAATACCCTCTTTGTAAGTCATCCGGAAAAGCATCCGATTGTCATACTTAAGGATGTGCCCTACTCG
GACATGAAGTCGTTGCTAGACTTTATGTACAGGGGCGAGGTCTCAGTGGACCAGGAGCGACTCACTGCATTCCTGCGCGT
GGCCGAGAGCCTGCGCATCAAGGGCCTCACCGAGGTCAACGACGACAAGCCCTCGCCGGCAGCAGCAGCTGCAGGAGCGG
GTGCGACGGGCTCTGAGAGCACAGCCACTACACCCCAGCTGCAGCGCATCCAACCGTATTTGGTGCCCCAGCGGAATCGC
TCGCAGGCCGGCGGTCTGCTGGCCAGTGCCGCCAATGCCGGAAATACACCCACCCTGCCGGTGCAGCCATCGCTGCTCAG
CTCGGCCCTGATGCCCAAGCGAAAGAGGGGCAGACCCCGGAAGCTATCTGGCAGCTCGAATGGCACGGGGAACGACTACG
ACGACTTTGATCGCGAGAACATGATGAACGACTCCTCCGACCTGGGCAACGGCAAAATGTGCAACGAGTCCTACTCCGGC
AATGACGATGGCTCCGACGACAATCAGCCGAACGCAGGACACACGGATGATCTAAATGAAAGTCGCGATTCTCTGCCCTC
GAAACGATCAAAGAACTCCAAGGATCACCGCGTGGTGAGCCATCATGAAGACAACAGCACTTCCGTTACTCCTACTAAGG
CCACTCCAGAGCTCTCCCAGCGGCTCTTCGGCTCCTCTTCGACGACAATCTCAGCAACGGCTCCAGGCGGCAGCAGCACT
GGTCCTTCGGAAACGATTTCCCTGCTGGAGATCAGCGATGAAAGGGAGTCCGCCCCGGTTCACCTGCCTACTATTTTGGG
CCTGAAGATACGCGCCATTAACACTACGACGCCTGCGCAGCAAGGATCCCCCCAGACGCCTACGAAATCCAAGCCGAAGA
TACGGCAGGCGACAGGAAGCAATAACAGCAACTCTCTGCTCAAGCAACAGCTGCGAGGTGGAGCCAAGGATCCTGAGGTG
CCGCCTGCCACCAGGATCACGGGGGCAGTGACCCCCAATGCTGCCCTTAATGCCGAGGAACAGTCCAAGGAGATGCCGAA
GAAAAACCAGGACGAGGTCAACGCCTGCATTGGTCTGCACTCGCTGGCAAATGCCGCCGAGCAGCAGGCTGCTCAGGTGG
CCAGCACTGGGAACCTGCACCACCAGCTACTGCTTCACATGGCAGCAAACAACTCCATGCTGAACACCACCGATTACTAC
CAACAGCAGCAGCAGGAGTCTCCCTCGAGTGCTGGCCAGTTTATGGATGACGACCTGGAGCTGCTGTCTCTTAATGATCA
GCAGGATAAAAGCGATGAACCCGATCATGAAATGGTCACGCTGGCTGATGAGAACGCTGGTTTGCCTGGTTACCAGGGCA
ACGAGGCGGAAGCCACACCTGCGCAGGAAGATTCGCCTGCTGCGGAGACAGCAACGGCACCACCACCCGCGCCTAGAAGT
GGAAAGAAGGGAGCTAAGCGTCCGATTCAACGGCGGAGAGTTCGACGCAAGGCCCAGTCGACGCTGGATGATCAAGCGGA
ACACCTGACAGAGATGTCCGTGCGGGGGCTGGATCTCTTCCGGTATGCGAGCGTCGTGGAGGGCGTCTACCGCTGTACCG
AGTGCGCCAAGGAGAACATGCAGAAGACATTCAAGAACAAGTACAGCTTCCAACGTCACGCCTTTCTCTACCACGAGGGC
AAGCATCGCAAGGTGTTCCCATGTCCCGTGTGCTCCAAAGAGTTCTCGCGACCAGATAAAATGAAGAACCACCTCAAGAT
GACACACGAGAACTTTACGCCGCCGAAGGACATTGGCGCCTTCAGCCCCCTGAAATACTTGATCAGTGCAGCGGCTGCAG
GGGATATGCACGCTACGATCTACCAGCAACAGCAAGATCATTACCACCGCCAGTTGGCCGAGCAACTGGAGCAGCAAAAC
GCCTCCTTTGACAGCCGGGACAGTTCGCTTATCCTGCCCGACGTGAAGATGGAGCATGCGGAAGACCAGGATGCGGAACA
GGAGGCGGAACTGAGCGATGGTGGCTATGACGCCTCCAATCCAGCAGCAGCAGCAGCCGCCATGTTGAGCCTGCAACAAG
ACGTAATCATCAAGGATGAGATACAGATCTCTCCGTCGCCCTCGCCCACCCCGCCCGCTTCCTGCGCTGTGGCGGAGGGC
AAGTCCTTGGCTTTGGCCTCTACCGCACAAACCGCAACGTAAAGACTTTGAAGGACCAGAGTTACGGAAGGACTACGAAC
GCACAACAACGTTTACCTTAAGCAGCGAGCTCTGAACTGTAGAATACATCATAGCAACATAAGCAATCGTGGCACTCAAA
CGCAACACACCAGACTCAAAACGATTTTTAGTTAATGTAAACCCGTAAGCAATATTAATTCTAACA***CTTGCTCCGTCCAG***
AACGCCTTTTTTAAGCAACACTAGTATTTAATTTACCTAGGACATAAGTTTTGCCCAATTGAGCAACGCAATTGAGCAGT
TCAGTGAACTTTAATGACTTTCGGCTTTAATTATAAACAATTCGACTGGCCGCTTACTGGATCCGCACACGATCCGTAAG
GCAAGCAACTTTAGAACTTTTCATTTCTCTTTGTGATTTTGTTAAACCTTTTTATATAGACCTTTGTTTTTTATTTTTTG
TTGTACATAGCTATATTTTGTTTAATACTCAAAGTCCGTAGTATAATTTTGTTCATTTCCTTGGCAACAAGTTGGAATCA
AATGTATTGGCTAATTGTGCAATCTTCAAAACGTGTTGGTGGCTTCGGAGGAACGCGACAACGCTGTTCTGCGGCCCCAC
GAAATGTTGTAAACTTAATTAACTACTAAGGCCACTTAACTAGGATCTAGGTGTAGTCATAAGGCATTGGATCAATGTTC
CAGCGTACAACAAAACGGACTTCCAGTGAAGAGACAACTGATATTTATACGGAATCCCAATCTGGTATTATGTATTATGA
TTTTTACAGTTTACTTAACGCAACAAATACGACTTAAGACTTGTGCGCTCAATGACTAGAAATTATAGTTGCGATTTTAG
GATAACTATTCATGGAAAAACCACAAACGAGTTTTTAATAAAGAAAATTTTACTGATT

53. yrt

GTCGTGACCACATCTGGTCACACTGCTCGCATAAGATCAACAACTACTGCGGCCATTCTTACATTCTACTCCTAAAAAAA
TTCCGAAAGTGCACTTAAATAGCCCAAAATAATAACAAAACATAATAGTGACCGGACGATGCAGCACCGCCAACAAAATA
AAGTGTGAAAACAAGTGATTGAATACCCGGAAAGTCGCCTCGCGAAGCGGGAACTAACGTGTTTGGCTGGCCTGTTTTTT
CTCTCTCCTCTTTTTCGCCAACCAAAAACCAGGCTAACAGTGATTTTTTCGCGGGGGAGGGGGTGCGACGAGCCAAAACC
CGTAGTAAAGAAACCGCCCGAAAGGAGCGCCCACTTTGGCCAGCAACTTTAGCTCAGCTCCCCGAACCAAAAGTGAAGAA
AAAAACAAAAAACTAAAAGGAAAACTTAAGTAAAACGGGGGCGTCTATCGCTTGCAAGTGTGTTGGTTAGTCAGTGTACG
TGTGTGTTGGCCAGAACAAGTTGGCTGCGCGTGTGTGCGTGTGAGGGGTTTTTTGATGTCTGCGCTGTCTTTTTTCCCCA
GCCGAGGTGAGCACACAGGTGAATTATCGGTGCATTCGGCTGCCTTGGCAACAATTCCATAAAAACAACACATAGAAAAC
AACAATAACGACCACCCGCAATATATAATACATACATACACACGTTTAAAACCAAACAAAATCCAGAAAA**ATGCTCCGTT**
TTCTAAGCCGTCGCAAGGTGCGCAACAACTATGTGGATAATTCGAGAGAGGGCGGCGGGGCAACTAGTTCCGCGGTGGGC

GTGGCTGGGGGTGGTGGTGCCATCACCTCCGGCGGCAGCTCACAAATAAAGCCACAACGCATCGTGGTCAACAAGAACAA
AATCGACTGCCGCGTCATTCTGCTGGACAACACTGACCTTTCCATCGAATTATCGAAAAAGGCATTGGGCAGCTTTTTGT
ACGAACAGGTATTCTACGCACTGGACATCATCGAGAAGGATTACTTTGGGCTGCAGTTCATGGATGCCAATCACGTCAAG
CACTGGCTAGATCCCACCAAGCCCATCAAGAAGCAGGTCAAAATTGGGCCGCCCTACACGTTCCGGCTGAAGGTGAAGTT
TTACTCTTCGGAACCCAATACACTGCGCGAAGAGCTGACCCGCTACCTGTTCTTCTTGCAACTGAAACAAGATCTTCTCG
AGGGGCGACTCGACTGTCCGGAGGACAAGGCCACCGAACTCTGTGCTTTGGCATTGCAGTCTGAACTGGGCGACTATGAC
AATCAGGAGCATTCCGCCGCCACCGTCTCGGAGTTCCGTTTTGTGCCCGAGCAAACGGAGGACCTGGAGATAGCCATACT
AGACGAGTACAAGACGTGCCGTGGTCTCACACCTGCTCAAGCAGAAACGGCATTCCTGAACAAGGCCAAGTGGCTGGATA
TGTACGGTGTTGACATGCACACGGTGCTCGGAAAGGATGGCTGCGAATACCACCTTGGCCTTACGCCCACCGGAATACTT
GTTTTCGAACGCGATCAGAAGATCGGCCTGTTCTTCTGGCCCAAGATCAGTAAGCTGGATTTCAAGAAAAAGAAATTGAC
TCTTATTGTGATCGAGGATGACGACGAGGGAAGAGAGCAGGAGCACACCTTTGTCTTCCGGCTGTACAACGAGAAGGCGT
GCAAACACCTGTGGAAGTGCGCCGTGGAGCACCACACCTTCTTCCGGCTTAGGGCGCCGGTCAAAGGCCCCTCCGCCCGT
CAAAACTTCTTCCGTATGGGCTCTCGCTTCCGGTATTCCGGTCGCACCGAGTTCCAGACCACGCAGCAGAGTCGAGCCAG
GAGAACGGTCCAATTTGAGAGACGACCATCGCAGAGATTTGCTAGCCGTCAGTCGCACCTGTTGCGCGAACGTCAAAAGG
CTTCCCAGGAGGCGGCGGTGTCGGCTGTGGCCTCGGTTAATGCTCGAGCTGCTGCCGCTGCGGCTGCCGCAGCCGCATCC
CAATCGCCTGCACCGGCTACTCCCTTAGTATCTTCCCAAGTATCGACACCGACTCCTAACAACGATAACAATAACGACGC
CTTCGACTCGCTCATTTCCACGGATCAGTTCATCACTGTTACACCTTCGCCTTCGGTGCTTACTGTCATTCAAAACTCTG
CCATTATTGAACCGGATGCGGCTTGCAGCGGCTCCATCAGCTCCTCATCCCAGGCAGCAGCCAACTTTGCTTCGCTGGGT
CGCAACTCCCTCAAGTCCAACTTCAGCAACGAGGATCCGGCGTACAGCAAGATTGGATCTATTGGCAAGGCGTCAGGATT
GACTGTGAAATTGGATCCAGACTACACACCACCATACTCACCGAACGCCACTAAGAACTTTGATGAAACCAATCCATTTA
GAAATGCAACGAGCTCCCACCACGGCAGCTTTGGCAAGGCTTCCATTAGCTCCGGTCAGCTCAGGGTGACTATCAACAAG
ACCTTCGACATGGACCACAACACGGAGACACTGAAGCGCCTCTCGAACGCCAACGAGAAACCCAATAACCAAGTGAAACT
GGCCAATGTGAACACCACGGCCCTGCCGCCTGGCAACATCAAGTGCAATATTTTAAAGGCTCGCGTTGAGGAGGAGCTGG
GTGCCGGAGGCAACGCCAAGCTTACCAATCAGATGTTCGTACCGGCCGCCCACAGCAGCAACATGCACACGAGCAACACA
AACAGCAGCAACAACCATGCACACAGCGATGGACCGGATTCCCTGAACGCCACCTACATATCAGTGGGAGGCGATAAGCT
GACGCTCAGCATACCGGAACAGAAGCCGTCGACGGGTAGTAGCAGTAGTTCCAGCACTTCGAGCACCGCTACCACCACCA
CCAATGGAAATGGACCCTATAGTAATGCCACCTTTGTGTCTGGATTCAGTGCACCACTCACTCCGCCGTCGTCGCTGCCT
GCGAATCTGAATAATACCGGAAGCGGGTGCAATACAACCCTTACCAGTGTGACCACCATCAGCACGCCCAGCAGCCCCAC
CGCCACCGTTAGCACGACCGCCTCGGAGTCAGTGGCTCCCGCTTTGAGCAACGCCTCGGCCGCCGAAATACTCATTAATG
AAATATTCATTAACAACATCATAAACAATAATGCCAGCGGCAGTATCACTGCCGAAACTGCTGCCAAGCCGGCGGGCAGC
TCGACGCCCAGTGCCGGCACCTTGCTCTTCTCCACGCTCAGTGAACAGGAGCGCTTGGAATCGCAGAAAACCAACCAGCA
GATGAATCAGAGTCTAAATCTGAATCAAAACCACAGTGAAGTCGATGCGCCGCCGAGTGAAAAGAAAACTCCGAGCAATT
ATCCGGACAGCAGTCGCATT***CCGTTTCCGTCCAGCAGCAACAG***CAAAGACATGGTATCGCCCTGGCTGGTTTCCTCGGAG
GTGGTATCGGCACCCAAGGGACGCGAGTCGGCCATCATTCGAAAATCCGTTATTACAACCCAGTTGTAATTATTGTTTAA
CAGTAACCCCTACTCCGCGTATAAGCTAAATGTTCTAATTTATAATCGGTAGCTATGGAAATTTAGGCAGACGTAACGTA
TTCGTATTTTATCGTAGACAAACTTAGACGAGTTTTAAGCATTCCAAGCGAGAGCAACGACAAAAAGACAATGAGAGAGC
AGGTCCAATCAAAAGCATACTGTCCCTAAATGTGCCTTAATGATCTATATTTATATACTTTACATATATCATATACACCG
ATGCGATATCGAGGAACGAAATGTGCTAAACCGTGTACTTAATAAAGTATTCAACCGCTAACTGTAAGTGTATAGACTGG
ATTTTTAACAGAAAATAAAAACGTGATGTTGGGCGTTCTGCTTAATATACTAGGCTTATATTATAGCTAAGAGATGTGAG
GACCGTTTAATGTGCAGTATTCCAATTAACAACTAGCAGACCACTAAGAACAACGAAATTTTAAACTGCAGCCACAAAAT
TGACTGAAAGTATACAAATCGAAATGAGCTGAATTGAATGTAATTGTGCGAGTATTTTTGTAAATGTATTGTCCGCCGGG
GGTCCCAGAACCGAAAAAATGACTTACCACACACCAACGAAATGTTAAGTGCAAGCAATCCACTCAATTATTTTTTGTCT
AAGCATACAGCAACGATGTTGAAGCATTTAAATGTATAAAATATTCAATAAAAAGTGCATTTATGCATCATGTATACAGT
ACAACAT

54. Gli

AGTCTTGTTTTTGGTTAGTGATTTTTTGTTTGTTACCTTTTCGGGGCGTTGGCCAAGAAAGTTATTGTGAGTGTAGAAAC
GCGTAAAAGTTGAAACTAAAATATCATC**ATGATGCACAAATTGAAATATCGCGATAAATTAAAATGGCTTTTAGCCCTTC**
TTGTGCTGATCGGCACTTGTTTTATTCAGACAAGGGGACAAACAAGAGATCCCAGATTTTATTCTCGGCCAGGCGTTGAC
TACCATTGGCCAAATCCAGGCGATCCGGATTACAGAACCTACACGTTCAACGATCGCCGATATGGTCATTATCAGCCAAA
TGGCTATGGAGCCAACTATCCAGGCAGAAATCCACCGGGACAATATCCACAGGGAATGCCGAATGAAGATCGCTTTCGAT
TTGACCCGAACGATCCGAATGCGAGAACCCAGTTTCCGGGAGTGCTGGCCGGATGGCGAGAGGATTTGCAGGGCAAGCAG
CGGCGGGATTCGTTGACCCTGGAGCGGGATGTTTTCGTGACCACCAACTATGGCCAGGTGCAGGGCTTTAAGGTGTACAT
GTACGATAATCCAGATCCGAAGTCCTTCTATCGTCCCTACCACTCGACCGTGGATCGTGTGATGGGCGAGTGCTCGGTCT
TCCTGGGCATTCCCTACGCCCTGCCGCCCACCTTCGAGGGCAGGTTCAAGCCACCACGCGTCCATCGAGGCTGGCAGCTG
CTGCAGGCCGTCGACTTTGGACCCGCCTGTCCACAGCCTGTGCGATATACGGGTGCCACGAAAGGAATCATGGACATGGA
CGAGGATTGCCTCTACTTGAACGTGTATTCGCCGAAGACTGGTGCTGGTGTGGCTCAAAAATACCCGGTTATGGTGTACA
TCCATGGCGGCGAGTTCATTCGTGGAGCCTCCAACCTATTCCAGGGTCATATTCTGGCCTCGTTCTACGACGTGGTCGTG
GTGACCCTGAATTACCGCCTTGGTGCCCTGGGATTCCTATCGACGGGTGATGAGAACTCGCCCGGAAACTACGGAATCCT
CGATCAAGCGATGGCGCTACGTTGGGTCTATGACAATATTGAGTTCTTCAACGGCGATCGGAATTCCATCACTCTATTTG
GTCCGGGAGCAGGAGGCGCCTCCGCTGGACTCCTGATGGTGGCACCACAGACGCGGAACATTGTGCGTCGTGTGATCGCA
CAGTCCGGATCGGCTCTAGCGGATTGGGCGCTCATCCAGGACAAGTATCGCGCCCAGAACACGAGTCGCGTGCTGGGACA
GCTGCTGGGCTGCTCCATTGAATCGTCGTGGAAGTTGGTCAACTGCCTGCGCACCGGACGCAGCTTCTATGAGCTGGGAA

ACGCTGAGTTCTCTCCCCAGGTGGGCAGCTTTCCATGGGGTCCAGTTCTGGACCACAACTTTACGTTGCCCGGCGACGAT
TGGTACGAGGGATGGCGCGAAAAGGATTGGCGTTTCCTCACCCAAACGCCGGAAACCCTCATCCGTGCCGGTAAATTCAA
CCGGAATATTCAGTACATGACGGGCGTGACCACACAGGAAGCGGCCTTTTTTGTGGCCCAAAACGAATCCCTAAGTCCGT
ACTATGAACTAGATGGACGTTTCTTCGATCAGAAAATAAGGGAACACGTTTTCCGCTACAACTATACACTTAATCCGAAC
GGAGTTTACGAGGCCATCAAGTACATATACACCTTCTGGCCGGATCCCAATAATAACACCATAATCCGGGACCAGTACAT
AAACATGCTGAGTGATCTCTACTACCGAGCACCGGTGGATCAAATGGTCAAGCTAATGCTGGAGCAGAAGGTACCCGTTT
ATATGTACGTACTGAACACCACTGTGGAGGCACTGAATCTGCCACAGTGGCGAAAGTATCCACACGACATCGAACGTTAT
TTCCTCACCGGAGCTCCCTTCATGGACACCGAGTTCTTTCCCAAAAAGGAGCATCTGCAGCGCAATATGTGGACGGATAA
CGATCGCAATATGAGTCACTTTTTCATGCAGACCTACACGAATTTTGCTAGATATGGCAATCCGACGCCGCAACAGGTGC
TAGGCATGCATTTCCAGCGCGCATACCAGGGCGAGATTCGGTACTTGAACATCAATACCACGTACAACTCCTCCATTCTA
CTCAACTATCGGCAGACGGAGTGCGCCTTCTGGACGCAATACTTGCCCACAGTTATTGGAGTGCTGGTGCCCACTTATCC
ACCCACCACGGAGTATTGGTGGGAGCCCAAGGAGCCACTACAGATCGCCTTTTGGAGCATGTCGGTGGCCTGTTTCTTCC
TCATAGTCCTGGTGGTCATCTGCTGCATCATGTGGCGCAATGCCAAGCGCCAATCGGATCGCTTCTATGACGAAGATGTC
TTCATTAATGGTGAGGGCTTGGAACCGGAACAGGATACGCGTGGAGTGGACAATGCCCACATGGTGACCAACCATCATGC
CCTGCGCTCCAGGGATAATATCTACGAGTACCGCGACTCTCCATCCACCAAAACCTTGGCCAGCAAAGCGCACACGGACA
CCACCTCGTTGCGCTCACCCAGTTCGCTGGCCATGACCCAAAAGTCCAGCAGCCAGGCGTCCCTCAAGTCAGGGATCTCG
CTCAAGGAAACCAATGGCCATTTGGTGAAGCAATCTGAAAGGGCAGCCACGCCACGATCCCAACAAAATGGGTCCACCGC
AAAGGTGGCGTCTCCTCCTGTGGAGGAGAAGCGTCTACTGCAGCCACTTTCCAGCACGCCCGTGACGCAGTTGCAGGCGG
AGCCGGCCAAAAGAGTTCCCACCGCTGCCAGTGTCTCGGGCAGCAGTCGGAGCACCACTCCGGTGCCCTCTGCCCGCAGC
ACCACCACGCACACCACAACAGCCACCCTGAGTTCCCAGCCAGCGGCTCAGCCGAGGAGAACCCACCTGGTGGAGGGAGT
GCCTCAGACATCCGTATAAGTAACAGCTCTCTAAGCGCAGGATGCGCTTAGGGAGTCAGCCGAATCGTCCAATTACAGAG
CCAATCAACAATCCAGGAGGAAGTATGCCAGTTTCTCCTGGAATTTTTCTTTAAAGTAAGTTAGTTATTTTCGAAAAAAG
TAGTTTATGTTTTGTATTTATGATAGGTTTGTAAACAGATCTTTGAGATCTCAGTTTTGAGTTGTAGTTTAGGCAAACCT
GTATTTCTAATGCCTTACTTTAAGTTAAATTGTTCGTAAGTTTGATAACCCAACGAATACAAATCCATATCAATGACATT
CCATAGCCTTATCATCGGCCTTTACGTGTATATATAGAATTTAAATGTATAATAAGAATTATTTAAATCTAGGAAATTGC
CTGCCTTAACTGAAGGAAATTAGCTATATAATACAAGTATTTTTATAACAAATGCTGAGCTTAAATTTTTAAAACTCACT
TTTAGTGGCAGTAATATCGAATGAAATTGTAAAGAGAGTATATCTTTTAAATCAAATATTAAAATACAAAAATAAATTAA
CCTTATAAAAAATAATGCATTACATTATTTAAGACCTGCTATTATTTGATAACAAACTCATAACATTGTCACGTCCCGTA
TTATAAGAATATCATTAGAATACCATTAGATCGTAGCCACTCCCACACATCATCAAGAGCAATACACCTACAGTGCCTTA
ATAATATTTGGTAATTGTCCTCGATGGACCCACCCACCCCATCCATGATGTACCAACTAAGTGTGCCTTTCATACATACA
TAAATGTACTCCTAGTTATATACTATATATGTTCTATATGTTCTGTGTGTCGCATATAAGCAAAATGA***CCA**TACAAAATC*
AGTGTGAACCGTCC*AATAACTTATGTTTTCAAAAGTGTACATTGTTTTTCCTGTATATATCATCATTTGTTACACGATTG
AAAGCATATTGTTTTTACCACATTATATGTTTATATGTACTTTAGATAAGTTTTATGTATTTGTCCATAAATAAATATTT
TTACATTA

55. Nrx-IV

TCACGGTCACACTGGCAGCGAGACATTCTGTTCGTGTGTCATCCGCGTTGCGATAATTATTTTCTTAATTCAAACGAAAC
TCTTCCGAAGTGGGCGTGATCTGCTGGAGCCGTACACCCAGTGGATAAATTACATAAATTCAAAATCGATTTAAAGCGGA
GCGTAAAGTGCAGTCCAACGTTTTTAACACCGCGGACGCGTTGCCACAAATCATCAGCTTTTCACGGTGGTTTTTCCAGC
GGAAAAGTGCATTTTGGTGTTAAACTTCTTCGGAAATCGAATATAGATGTGTTTATAAATCTACCAAGCGATAGTGTTTA
ATTAACAGCCTCCAAG**ATGAGGCCGCCCAGGAGCAATACGAAAGCAGCATTTTCCTCGCTTCAGTTTGGATTGTTATGCC**
TCCTATTGCTGGTCAATAATGGCATTAAATCGGTGCAAGCAGACGCCTTCACGGACTATTTCTCCGACTACGATTGCAAC
CAGCCGCTGATGGAAAGAGCCGTCCTCACGGCCACATCATCCTTGACGGAAAGAGGACCCGACAAAGCACGACTAAACGG
TAACGCTGCTTGGACACCGGTCGAGAATACCTATAACCACTTTCTAACACTCGACCTGGGAGATCCGCGCATGGTGCGCA
AGATCGCCACCATGGGACGCATGCATACGGATGAGTTCGTGACGGAGTACATTGTCCAATATTCGGATGATGGCGAGTTC
TGGCGCTCCTACGTGAATCCTACGAGCGAGCCGCAGATGTTCAAGGGCAACTCCGATGGCAACTCTATCCATTACAACGT
CTTCGAGGTGCCCATTATTGCCCAATGGGTGAGGATCAATCCCACCAGGTGGCACGATCGTATTTCCATGCGTGTGGAGC
TCTACGGCTGCGATTACATCTCTGAAAACCTATACTTTAATGGCACTGGCTTGGTGCGCTATGATCTGAGGCGGGAACCC
ATTACCTCCACCAAAGAGTCCATCCGCTTCCGCTTCAAGACGGCCTTTGCCAACGGAGTGATGATGTATTCGCGTGGCAC
ACAAGGGGATTACTATGCCCTGCAGCTTAAGGACAACAAAATGGTGCTGAATTTGGATTTGGGATCGCGGGTGATGACCT
CCTTATCGGTGGGCAGCCTGCTGGATGATAATGTGTGGCATGACGTTGTGATCTCAAGGAATCAAAGGGACATAATCTTC
TCAGTGGATCGTGTCATCGTAAGAGGACGCATCCAAGGCGAGTTCACACGACTGAATCTTAATAGGGAGCTGTATCTAGG
TGGTGTGCCCAACGTACAAGAGGGCTTGATTGTCCAACAAAATTTCTCCGGCTGCTTAGAAAACATCTACTTTAATTCGA
CCAACTTTATTCGAGTGATGAAAGACAGCACTGAACTGGGAGAGGGATATCTCTTCACTAGGGTAAACACGATCTACGCG
TGTCCTTCGCCTCCGATTTATCCGGTGACCTTCACTACCCGTTCGTCGTTCGTCCGTTTGAAGGGTTATGAAAATTCGCA
GCGTCTTAACGTCTCCTTCTATTTCCGTACTTACGAGGAAACTGGAGTAATGCTGCATCACGACTTCTATTCGGGTGGTT
ATCTAAAGGTCTTTTTGGAGTTTGGCAAAGTGAAAATTGATCTTAAAGTCAAGGACAAAGCGCGAATCATTCTGGACAAC
TACGATGATCAGTTCAATGATGGCAAATGGCATTCGTTCGTTATATCCATCGAAAAGAATCGCTTGATTTTAAATATTGA
TCAGAGACCGATGACCACAACAAAGAGCATGCAGGTTGCCACTGGAGCACAGTACTATATTGCTGGTGGAAAGGACAAAA
ATGGATTTGTCGGTTGCATGCGCTTAATTTCGGTGGATGGAAATTACAAATTACCGCAGGACTGGGTGAAGGGCGAGGAG
GTTTGCTGTGGCGATGATGTCGTCGTCGATGCCTGCCAGATGATTGATCGCTGCAACCCGAATCCTTGCCAGCATAAGGG
ACTTTGTCACCAAAACTCCAGGGAGTTCTTCTGTGATTGCGGACATACTGGCTACGCCGGAGCTGTTTGCCATACATCAA
ACAATCCATTGTCCTGCTTGGCTCTCAAGAATGTCCAGCATGTGCAGCAGCGTGTGAACTTGAATCTCGATGTGGATGGC
AGTGGTCCCCTAGAACCTTTCCCAGTGACCTGCGAGTTCTACTCGGATGGACGCGTGATTACCACCCTCAGTCACAGTCA

AGAGCACACCACAACAGTAGATGGTTTTCAGGAGCCTGGCTCCTTTGAGCAGTCCATCATGTACGATGCCAACCAACTGC
AAATTGAAGCTCTGTTGAATCGGTCTCATAGCTGCTGGCAGCGCCTGAGCTACTCCTGCCGCTCCTCCCGACTCTTCAAC
TCGCCATCTGAGGCCGGAAATTTCCGTCCCTTCTCTTGGTGGATATCTCGCCACAATCAACCGATGGACTATTGGGCTGG
AGCTCTACCTGGCTCTCGTAAGTGCGAATGTGGAATTCTGGGCAAGTGTCACGATCCCACAAAGTGGTGCAACTGCGATT
CGAACAGTCTGGAATGGATGGAGGATGGTGGTGATATCCGGGAGAAGGAATACCTTCCCGTGCGAGCTGTGAAGTTTGGC
GACACTGGAACTCCGTTGGACGAGAAAATGGGTCGCTATACTCTGGGACCGCTGCGCTGTGAGGGCGATGATCTGTTCAG
TAATGTGGTGACCTTCCGCATTGCCGATGCTTCTATTAACTTACCACCATTCGATATGGGTCACTCTGGAGATATTTATC
TGGAATTCCGTACCACACAAGAGAACTCTGTGATCTTCCATGCTACCGGACCTACAGATTACATTAAGCTAAGTCTCAAT
GGCGGCAACAAATTGCAATTCCAGTATCAGGCGGGAAGTGGTCCACTGGGCGTTAATGTGGGCACAAGCTATCACCTGAA
CGACAACAACTGGCACACGGTTAGTGTCGAGCGCAACAGGAAGGAGGCTCGTCTGGTGGTTGATGGTTCTATCAAGGCTG
AGGTTCGAGAGCCGCCGGGTCCAGTGCGTGCACTTCACTTAACATCGGATCTGGTGATTGGCGCCACTACTGAGTACCGC
GATGGTTACGTAGGATGCATTCGTGCACTGCTGCTGAACGGAAAGATGGTTGACTTGAAGGAATACTCCAAGCGTGGATT
ATACGGCATCAGCACCGGTTGCGTGGGTCGCTGTGAATCAAATCCATGCCTAAACAATGGTACTTGTATCGAGCGATACG
ATGGCTACAGCTGCGATTGCCGTTGGAGCGCATTCAAAGGACCTATTTGCGCAGATGAGATTGGTGTCAACCTACGCTCC
AGTTCGATTATTCGCTACGAGTTCGAGGGTTCCTTCCGCTCCACAATTGCCGAGAACATTCGCGTCGGTTTCACCACCAC
CATTCCGAAGGGATTCCTGCTTGGCTTCTCTTCCAACCTAACTGGCGAATATTTAACTATTCAAATATCGAATTCTGGTC
ATTTGCGTTGTGTATTTGATTTCGGTTTCGAGAGGCAGGAAATCATATTCCCCAAAAAACATTTTGGCCTGGGTCAGTAC
CACGATATGCACTTTATGCGCAAGAATGGTGGATCCACAGTGGTTTTGAAGGTTGACAACTACGAGCCGGTCGAGTATAA
TTTTGATATTAAGGCCTCAGCGGATGCTCAATTCAACAATATCCAGTACATGTACATTGGCAAAAATGAATCTATGACAG
ATGGCTTTGTGGGTTGTGTGTCGCGAGTTCAATTCGATGATATCTATCCACTGAAGCTGATGTTCCAGCAGAATCCTCCC
AAGAATGTCAAATCATTGGGCACTCAATTGACTGAAGATTTCTGCGGAGTGGAGCCAGTTACGCATCCGCCCATTGAAAT
CGAGACTAGGCCACCACCCTTGGTGGATGAGGAGAAGTTGCGCAAGGCATACAATGAAGTTGACTCTGTGCTGTTGGCAT
GTCTGCTTGTGATTCTCTTCCTGCTGCTGATCCTTATGTTCTTCCTTATCGGTCGCTATTTGCACCGACACAAAGGCGAC
TACCTGACGCACGAGGATCAGGGTGCCGATGGAGCTGACGATCCAGACGATGCTGTCCTGCACTCAACTACTGGCCATCA
AGTCAGGAAGCGAACAGAGATCTTTATCTAAACCGGTCGCAAGAATATGCACATAAATCGTTTTTAAATCGCCTTAGGCA
CGCAACAGCGACCACTCAGACACACCAAAAGATAAAAAAAAAATAGATGAGAACGAAGTGAAAATCAAATAGACACGGAA
AAGGCTCACCACGCATAACAGACAGAAAATACTTTTCCATGCACTGCCAATTGTTTTGCGCAAGTAAATGCTAAGATTTG
ATTTATAATTGGTTGGCTACATTTTGCACTTATGTTTTTTTTTTTAGTCTTTGGTTCTGTCTGACGGCATTATTGAAATT
TCTTCTATCACAGAATA***CCGTCCATTTTTAGTTAATTTTA***TGAACTTTTTGTTTGCAAAATTGAGTTAAAAACTTATTTA
AGGGAGCTGCCCGAGTAGCTCAACAAAAGCCACCTATTTTATGTATTTCTATTGCGCGAGTATGTAAGAACGAACATTCC
ATTATTTTTAATATGTTTTCTCGCTTTACGTTTAATTTAAAAATGTCTTCTACTTTACACACAAAAGAACATGAAAATCA
TTTTGTGGCTAAACAAAATCGGCTACTTTAGTAACACAAAGCATTAAAAACAAATTGTATTTTTGGAAAATGCATTTTGA
ACGTCTTGACTTGAAATTTTCAAATATACATTAGCATGTGTATATAGATATATATAGCAATAAAAAAATATATATAATTA
CAAAAAAGAGACAATGAAAAAATTAAAATTTTTATCTTTCAATAAAAGAGAACTACAAAT

56. Lac

ATTCATTCGTTTCGCGACTTTCGAACTGTGAAGTTGTCTCTCCGGCGCGTTATCTCCGTCTTGGCCAAAACTCGTGACTG
ATCGAGAGAAGAAGTCTGAAACCAGCTCTGAGCGAGAAGACAAGTGTGGAGACTGCAGTTCAGCATCCGCGTTTGCTGTG
CTCAAGAAAGAAACGGCAATAGTTGTCTTCGGTTTCTTGGAGACGTCTTCGCCGCGCTCTCACCCTATTTGGAGAAGATT
GAGATCTTGGAGCGCAGCTCTTGAGAAACACTACATATATTAAATCGCGCGCTTGCAGGGTGTGGTGCTAAAAGTCAATT
TCTAAG**ATGTGGCGGCCGAGTATCTCGAATTGCGTGTGGAGCACCCTGCTCCTGGCCATTTTTGTGCAGCAAACGCTAGC**
ACAGCGAACCCCGACCATATCCTACATCACGCAGGAGCAGATCAAGGATATCGGCGGCACAGTGGAGTTCGATTGCTCCG
TCCAGTATGCCAAAGAGTACAACGTGCTGTTCCTGAAGACGGACAGCGATCCCGTCTTCCTCTCCACGGCTCCACGCTG
GTCATCAAGGATTCGCGCTTCTCGCTGCGGTACGATCCCAACTCGTCCACCTACAAGCTGCAGATCAAGGACATTCAGGA
GACGGACGCGGGCACCTACACCTGCCAGGTGGAGTTCGCACCAGTGATCACCGTGCCGCGTCCTCGTTTGGGACAGGCTC
TGCAGTACGACATGGACTTGGAGTGCCACATTGAGGCCTATCCGCCACCGGCCATTGTGTGGACCAAGGACGACATCCAG
CTGGCCAACAACCAGCACTACAGCATCTCGCACTTCGCCACCGCCGACGAGTATACCGACTCGACGCTCCGTGTGATCAC
CGTTGAGAAGCGCCAGTACGGAGATTATGTGTGCAAGGCCACCAATCGCTTTGGAGAGGCGGAGGCGCGCGTCAATCTCT
TCGAGACGATCATTCCCGTGTGCCCACCGGCCTGTGGACAGGCGTACATCGCCGGAGCCGAGGATGTGTCCGCCACTTCG
TTCGCTCTTGTGGGCATCCTGGCGGCGTTGCTCTTCGCCAGATAAGCCAATGGGCCCACGGCCGTCGACTCCAAGCGCTT
CAGGTCCAATCCATCAACCAGCCCTCGAATGCATAAAACATATATAAAATAGATATATCGAAAACAAACATCGTGTAACC
GCGCAATAGATGTCTCAAACGATTTCGGACACACCAACCCACATCCAGTTCTCAGTTCGTTTTGTAGCTTCTGCTTCCGT
TTCCGTATAGTCCAGCCCAATTTTTGTACGTCCAATATTATGTCTTAAGTTAAGTATGATAAGTGAAGGGCCAACGATAA
CTAGACTATAATTTATTAGCACCCTAGAGCCGAACTCAAACCGAAATTAGACTCCAGCCCAGCCGTAAACGATGCATTTC
A***CCTTGATCCCGTCCGTTTTGCTT***TCTGATCTGGCACTGTATATGTTTTAAATATTTTAATCTAAGCAAGATACATCACA
AGCATTCCGATTTTGCTTCGACAATTGAGAGGATATTTTATTTTTATTTAATAATAATAAAGTATTTTCGTTACGCAAAC
CTATCAGCTCGACACACTATTCCCTAAATAATATCTCAACCCCCATAATCGAGGCAAAATCCATATTCGAATAAAAGGCA
ACAAAAGTTGTGTACATTATTAGCTCATAAGCACAATGAGAAATTCTTTTGAATAACAAGACATTTTTACCCAAATTTTT
GTGTGGGTGTGAGTAAAATCAATTTCGACTTTGTAACAAAAAAAAAAAAAAGACAAACGAAAAACTAAAATTAACAAAAAA
AAATGAATAA

57. santa-maria

ACAGTCGCATCGAAGCCGCCGGGCGAAGCGGTACCATTTTACGGCATTTCCCAGGGACTCGTGTCCACTTTTCCGGCCTC
TGTGCAAAGAAATCATCCACGTGCTCAAGCGTTCTGTCCACATCCTTATCGAATTTCAATTTCCTCGCAAGGAAAATCAA

AACGTAAAAAAAAAAACCGAGGAGCCTGTGATTTGTTTCTATTTAGCAAATAAAAGTCAACCACCATTAGGTATCCACACA
CCATCGCCCAGCGTGAGCC**ATGCCAACACAAAACAGCGCAATGTGGGGCCAAAAAAGTAATCGCAAATTAATTATCGGCA**
TTTTCGGCTTTTGCCTGGGACTATTTGGCATATTGTGCGGCATGTTCTGGGTGGACCTGTTCGATTGGATCATGCACAAG
GAAATGGCTCTGGCCCCCGACACACGTGTCTATGAAAATTGGAAGAGTCCGCCAATTGATCTCAGTCTGGACATCTACCT
GTACAACTGGACGAATCCGGAGGATTTCGGCAACCTTTCCACAAAGCCCATTCTGGAGCAGGTGGGTCCTTATCGTTTTA
TCGAGCGACCCGATAAGGTGGACATTCACTGGCATCCAGAAAATGCATCCGTCACTTATCGCAGGCGCAGCTTGTTCTAC
TTTGATGCGGCGGGCAGTAATGGCAGCCTGGACGATGAGATTACCACGCTTAATGCAGTGGCTCTGTCTGCAGCCGCCAC
AGCCAAATATTGGCCACCAGTTAAGCGCTCCCTTGTCGATGTGGGACTAAAGATGTACGGTGCCGAGATGTCTGTCCAAA
AATCGATCGATGAGCTGCTCTTCACCGGTTACAATGACGCAATGATCGACGTGGCCATGGCCATGCCCATTTTTGGTGAT
GAGGTGAAGGTTCCATTCGATAAGTTCGGTTGGTTCTATACGCGCAATGGAAGTGCCGATCTTACCGGAGTTTTTAATGT
CTTCACTGGCGCCGATCAACTGGCCAAACTGGGTCAGATGCACTCGTGGAATTACCAGGAGAACACTGGATTCTTTGATT
CCTATTGCGGTATGACCAATGGATCCGCCGGGGAGTTCCAGCCACAGCATCTGAAGCCCGGCGATAGTGTTGGTCTATTT
ACGCCCGATATGTGCCGAACTATTCCGCTGGATTATGTGGAAACTGTGGATATTGAAGGACTGGAAGGTTATAAATTCTC
AGGCGGACCACGATCTGTAGATAATGGCACCCAGTATCCTGAGAACCTTTGTTTTTGTGGTGGCCAATGTGTTCCGTCGG
GTGTGATGAACATCAGTTCCTGTCGCTTTGGATCTCCTGTTTTTATGTCCTATCCACACTTTTTCAACGCCGATCCTTAC
TACCCAGATCAGGTAGAGGGCTTGAGTCCTAACCAGAAGGATCACGAGTTTTATATGGTTGTGCAGCCAAGTACTGGTAT
TCCCCTGGAGGTGGCTGCCCGATTTCAAGTGAATATGCTCGTGGAGCCAATCCAAGGCATTAGCTTATACACTGGAATAC
CAAGGATATTTTTCCCACTCGTATGGTTCGAACAGAAAGTAAGAATCACTCCAGATATGGCAGATCAACTGAAGGTGCTG
CCCATTGTCATGCTCTCCGGACATATATTCGCCGGAATTTGCTTGATCGTAGGCATAACTCTACTCTGCTGGACTCCGGT
TCAGATTCTATTGGCTTCCTGTCGAAATCGTAGATACGATCTGAGGACCAAAACCAAAACGAATGGCCAGTATAAGAGTC
GCTCTCAATTCTCCAGTGCTGAGGAACTGAAGTCCAAGGCCTCCACTTTGGTGTGCGAAAAGAGTGTCAAAGGATCTCCG
GACAGTTCTCCGCTCCTCGAAAAAGGCCGTAAGCCAACCATTATTAAATCCCAGACGGGTGAAAGTGTGGCCACTGCATC
CACAGCCATCAGCGACAATAAGCAGGATTGAACAGAACCGCCACATTCACG***CCATTTAGATAGTTAAGCCGTCC***TAGTGC
TTTAGTCGTAGCTCATAGTTTAGTGACACTCTAAAACACCCAACTGAAAATTGCAAGCATTCCCGTGCCTATGCCAAAGA
TTAGATAGCCTTCGAAGTCGAAACGTCTGGTGGACTTCGCTTAGCTAACGAATACTCTTTTTACATTCCTTAGTTATTAG
ATATGTTTTGTATTAAAATGATTTCTTGACCCATGGCTTTCGTGTCAAATAAAATCTTCAACTAC

miR-184 MREs in long non-coding RNAs

58. lincRNA.924

CGCCATCATGCGTTGGAATAGTGAGGAGCTTGCCTTTGCCGTTGAGGCCTACCTTTCAAGCCAGTGTTCGGTTATTAAAA
CACAGCGTGCAATTCGGAATCGCTTTAATTTAGCCCCGTTTGCTCTCGTCCCGGACCGCAAATCAATTGTTACGTGGGTC
ACTACATTCAGGCAAACTGCAAGTGCGACAAAGAACTGGAGTCCCTCGACCGATCGTTCTAACGTGACTATCTCAATTAG
AGGTGATTTGGAATGGCCGACTCTCCCGATCTGTCCCCTCGTGATTTTTGAAATC***CCGTGTTTGTGTGGACCGTCCAA***GA
ACCCTACAAGATTTGAAAACCAACATCCAAAAAGAAATTGCCAACATAACACCTGCTATGCTAACCAGAGTAATGACAAA
CGCCAGAAATCGGCTTACGCAATGTATGATGAATGGGGGACGTCACCTATAAGATTTCATCTTCAAAACAATATAAATAA
AAACTTTAGACATGTACCTACATTATAAAAATAAATAAATATTTTCCGATGCCTACAATAGTTTTC

59. lincRNA.218

GTTTAATTTAATCGCTTCATATGTTGTTTAAAGCCAAGTATTTAAAAAATATATAAAAAAAATTAAAACTAAATTAAAAA
TCAAGCACCGGCATTTTTGCTTGAGTGCTTGACCTTGTGTGCGATCTACCGCTGAGTGTTGGCCGGGCTTGATTGCATGG
CCTTCGTCTTCGTCTTGGTTTTGGGCTTAGTCTTCATCTCCATCGCCGTCCATCGGCATCGTCCATCTTGGCCGGAACAT
TTTGTGTGGCTTACTCAGTTTCTGATTAACAATCAATCAATTTCGCGTGCGCCATCTACTGCTGCTCCGATGAATGAATC
GACTTTCGGCCTCGCATGCGTCACTTAAATTCAGACAGCACCAACCGCAGCCATAAAGGTTATCGAGGGAGGAAACCATC
GGGCCACTTAGTCTTCGCCAGGCTCCAAAAGTTGCCATCAATC***AACGCGGAAAGTCCGTCCAAAGG***ATAAGCAAATGACG
GGATGCAAGTGAGTCCTCCAAAGGGACGACAGGTGCCAGGTGGCGGGATGTAAGGGGCAAGACCCACCTCAACCCAAATG
CCGCTTATTTATGCAGAGCGCGCGGGATGGGCGAAGCCGCAAAGTGATTTTAAATGGGACCCATACACCAAAACACTGAA
ACACCGAATCACCGAAGCTCTGAAACCATGAATCCGGAGTCACAGGCCGTCAAAGCTCTGTTGATGTAGTCGTTCTCGGG
ATGTTGTCCATGGGAGCTCGGCATACTGGGTGAGCTGCCGTTGCCGGGCCATTAAAACACGTTGTCTAAAAAGAAAACAA
AGTGCAGCCACATGGTTAACTTTTCCATGCGATTGGAGAACACAGTTAATTGAAAAGAGTAGCAAAGACAGAAAAAAAGG
GATATGTATTAATTTACGGCTACGATAAACCCAGATTAG

60. XLOC_001425

TTATATTTTATTTAATTTTTTATTTTATTTTACTTCTTTTTTTCCAGTGCCTTGCCCTTTCGTTGGATCGTTTTCACTTT
GCCCAAACCGCAAGCCGGGAAACTTTCGCACCTGTGTCGAATGACCAAAAAGCAGAGGCAAGGAGCCTGCAAGTTAGACC
ACAACTTCCTCGTCGCAGGACGATGTGAAAAAGCCGATGTCTAACCCTTTTTTTTTTTGCTTAATTGCGGTGCCAGATC***A***
TAGCCATCCGTCCGTCCGTTGGTGAAACAAAGTAAAACGACAGGAGTCCTGCAATCCGGATCCTGTGAATTCGAGCCGGA
GTCAATGGTTGTATATCGCTTGCATAAATTATCGTAACTGTAACAGTCGCGCATGTTTCAGTTCATTAGGGGACACTTTG
CGACCTTAACCCTTCTATTCCATGGACTGAACTACGACGAGCTACATGTTCCTCTGTCCATATGCAAAATAATGCCGCAT
CATTAGTGCGTCGGTCGAC

61. lincRNA.678

GCCGTACATGATAGTACAATACCATTAGATCGCATATCTACTTACAAGAAACTAATAAGACTTTGTATTTTATTATCTCT
TACAACTTTCCTACTATAGCTAAAGGTTAAACTTTTAAAGTAAGGATGGTGTTTGCAATACGCATATAAATAGGCGAGCT
GGCCAGTGTACAAGGAGCCCTCTTCGAGGGGTCCGCTGATGAGGGTCCGCGAATGAGCTGTGGCTGGAAGCCAGCGCATC
AGGCTCGTCGGTTTGGGGTTTCGTTAAATGCAAAGTAGTGTGACACATGCAAAGTTTCAGGTTCCCAATGGTCGGGGGAG
GTGGCAAACAATGTGACCGCATTTTAGTCACAGTCACACTCGGGCACACAGACGTTACATTTAATTCAGAGATTTCCGAG
CCGAGCCGCGTGCCTCCACGTGAAGTGCAAGATGAGTGCGGGGTGGAAGTGGAAACGGTTAGTGCGAAAGAGGCAACTCG
GGGAGGACTCCTCCGTAACTGGATGCTGGATGTCGGTTGCTGGTTGTGTGCACTGCAGCGGGCATAATTGAGTTTGTTTC
AAATGCTTTTTACTAAGCACAACCATCAAACGCAAATACACACTGGAAAACGATTTACGAAAGCTGGACATTTGCAAGTT
GGGTCTATGGGCATTACATGTACTGTCGTATTAGTCCATATTAGTCGCTCCTTTAATGAGTTTCATCAGAGTCGTCTTAA
GTTACCAAGTAATAATCTATACTATTAAAACACAGTAACTTGCCTATCAAAATGTATCTTCAACATACTATGAGATTACA
TACATGTACAATATGAAATTTTTTGATCAATTCGAGACAGGTTGACACACTGCTTGTACTCTTCCGCTGGTTTCTCCCAG
TGTATTTCTGCTGCTTCTGATGTTTCTCTTGCTGCTGGCATCTCTGGTCTTGTGGGTCGTTTCGGTTGCCGCTTGTTGGC
TCTGCTCTTGGCACTTTTCGACATCAGCAACGTTGCCGTTGTCGTTGTCGTTGTTGTTGCCAGTCACTGGTTGGCATCGG
CAACGGAACAAACACAAATACACACACTCGAACATCCGCTCACTACGGGGTGATGGTGGAGCGAGACGGGGGTAGGGAAA
CACGCATGTCGCCGATAAGTGTGTTTGTCGTGCCATATATAAAATTATTACTTTCGACATGCAACTGCAACAGAAACAAC
GACTTGGGCCTCGCTTCTCCAACTCACATCGCACATCTCGCATTTCTGGGCAGGAAGAGCCGCTTTGCATCCGATGGATG
CCGCCTGACCGAATGACAGATACGCTGTCATTGAGTTGTTTTCCACACTCAGAAAAAGATTTGTGTGTT***ATCTGTCCTCA***
TTATAAAACAGGTTTCCCCACTTAGTGCTTCGATTGACGATTGATATCACACAATCAACACATTTTCCTAGAAAATTTTA
ATTCATTATAATGTTATCGATTTAAGGAAAAATATTCCCCACGCGTCATAGTTTTGACCACTTTCTGGGAATTTCCAGTT
TTATGAATTTGTTCGTTCCTAT

62. lincRNA.903

CACGTACACAAATCGCGCGAGATCGAGCATTGCATATAGTATAGTGTAGTATAGTATATCGCATTGACTTGGTAGACGGG
CAGGCAATAAATTAAATTTTCTCTCGGCTGGCGAAAAAGCCAAACGCTGAAAAACGTGGAAATGCAGCCCTAATCGAAGG
CGAAGTGGGTTTCCCCCGGATTCAGCGATCTCTCCGCTTTTCACCGTTTCCCCAGCGAGGCACAAATGAAGCGCGCCGTG
TGCCACACTTTTCCACCGAAAGTCGAAATGCGTTTCCAGTTTATTTTCAAAATGTTCAATTTCAATAATTTTTTAATGGC
CTCAACGCACGCACGCACGTCCAGGCAACTCTATAAGGCCCTGCTCCCTACGATCCCCAACCCACACAACCGATCCGTTT
CGATCCCATCCCATCCGATCCGAGATGAACTCAACCGATCTGAACTGAGCCGTTTGGCCAATTAGTCGAGATGTTTGCAC
AAATGCCAGACAAATGCAGACGAGTGCCGGCGGCATATAAAATTTCAAGCTCATCGCGTCCGATGGGCGCCATTGTGCAG
ATCATGATCCTAATGGGGTTGATCTGTTTAAAACACCGGAAACAAAAAGGGTTACATCTTGCTCAAAAGACCTCTTTTTT
AGTCATTCACATTATAATCTCTAAATAGAAACGTAAACAATTTGGAAAAAATTGCTGAACAGTGCAGATTTGTATCTGCG
ATCGGAATGCGCCTCGATTATTTGTTTGTACACGGGCTTCAAAGGAGCGTGCCCAGTTTTATATCCCATTGCTAGATGCA
TTCGCTTTGACATCTCAAAGCAACATAAACCGGCGAATCGGGCAGGGAAAACACCTTTGTGCCGACACCTATAAAAATGT
CCGATCTTTACGATCGTTTAGCCCCACCACGTCGAATGGCAGACCGGGTTTATATGGTCGGGCGACTTCCACGAATTCCA
CGCCGGAAATTATGAGGTCGGCCTTTTGAGGCGACAACAATGTTGCCAAACATTAAAATATGAAAATTCACTTAAAAACG
TAAACAACACGGACTCGTCT***CCGTTCTCCGTTCTCTCTGGGCT***CCCCATTTCGACATAAAATCATCCACTGAAAATTGAA
AACTGAAAAACAGAATGGCAGCAAAAAAAAAAAAGCAAAATCCGAGTGTCCAAGGCGGCAAACGCTTTACGATCGATCGT
TAAATAAATTGGCAAATAAATGTAAATGAGGCATGGTCAGAACTGAACGGCCGAACCCGCCCCCATCCCACGACTGCATC
CATCAATGGCGATTTTGGCGGAACAATCCGCCGGACATTTGGGCAGGTCAGCCAACCAAATGGATCCTCGAACGACGCTG
TGGAAATGTGAATTATAACTGAATTTGCATATCATGCAAGTGTGGGCATTCGCATAAATCACAGCTCTCAATGGGTTTCT
TTGAAGAATTCAGTCAGTCAGTCAGATGATGATGGATAAATAGATGCCTTATGGGGCCAAGGATCGATGTACTACTGACT
GCTGAGCTGATTGGGTGGAGGTGATGGCAAGGTTTAGATTTTGTAGATAGATAGTTGTGATTAAAAGTGAATTTTGATTG
ATTGTTGATAATATTTTGTGTAATAAACGGAAGTGATA

63. lincRNA.677

GGCGGTACGATACACATACATATCAGCAGTATCTGGCCTGGTTGAAAACGGCGGCCCACACGGACAGATCTCGTTCATTT
CATCTTCGCTGCTGATAACCAAACAGCCGCGTATACGACGAGACAGGAACGTGGG***CTGCGTTCTGTCCGTCCGTTTGT***CC
GCTTGTCCGCTTGT***CCGACCGTCCGTCCGGCTGTCAG***TCCGTCTTTGGAAGCCCCATCTGATGTGCGAAAATAAATTCCC
CCAATGAATTGGACAGTCGACGGGGACACACCCAGAAGAAAAACACTGGCGGAAAAAGAAATCTGAAAACAATTTGAAAG
TGTTTGAACTTATATTGACTTTGACTGCTGCTTACTAAGCTATGAGATGGATTTCTAGAAGACGGTCGACGACAGGGTAA
GTGGCTTCTTTCTTGAGCTCCGACTGTTTTAATTGCACTTCGCTTAATTTGTCATTAGCCTCTGACTTTGGCCTCCAACT
CCAAAGGGCAAGAGTTGGGGAAAGGCAGCGCCAGAGTGCCGGAGTCATTCATCTTCTGTCATTTATGTTTTCGCTGGTCT
GCGGACTGGTCTATTGTATCTGGCAGGCACTTTGTCCAGCGGCGTTTTTGCAGCTTTCCTTTCAGTATCCCTGCATTGTT
CGATTTTAGCGGGGCAATTAGTCAGGCCAAGTAAATGATTTGCCCAGCGAACTTATCCGATCCGGGTCCGCTGCTCCTGA
CAACCCTCCCGACCACATGGTTCGCAGTGTGTATCTGGCAGATACTTACGGGTAATGCGGATGTACACGTCTG

64. CR42874

TGCATTGCATTCGATGGGAAATGCAAGATGACTATGCGATACTCTTGCAATCCTGGAACTTGACCCGATGGCTTATCTTC
TCGCCCTCCTGTTAGTCAACTACATTGATATCCCAGCTCTTGGGACGACTTCTCACCGACATGGCGTTGATAATGAGCCA
GCTGCTCAATCGGAATCGAAAACAATGCTCGTCAGTGTTTTGGTTTCGATTACGGTCTACAGTTCGCATTCGGATTCGTA
TTCGCATTCGCATTCTCGTTTT***CTGCTCTCCGTTCTTCTGAACAA***AGAATAGCCTGATAAGACGCCAAAAACAACTGAAA
CATCAGAAACAGCAGAAATAGGCGAAGTGGTTGGCAGTGGGTAGTGGGTGGTATGGGTGGCAGCAGCTGAAGCCAAAACA
CACTCACACACACACACACACTCACTGAAATTCTCCGGCCATATGGCCATATGCAAATAATGCTGGCGGGTGGAAAAGCG
TGGGGGCGGTTATAAGAGGTTGGTGGGTGGTGCATCAGATAGAATTATCCGCAGTGCGCTAGCACGGGGATATTTAAGCA

AAGAAATCGCTTTATTACATTCACTTTGCGCCACGAATCGCTTGAAATGCAATTCCGCCAGTGATTCGGTGTCGGATTTG
GGTTTCCTTTTCGGAGTGTTCATTTTTTGTGGCCTTAAAGACAGTTTGATCCGACTTGTGTGCATCTGAATGGTCGCATA
ACTTGGGGATTCATCAACATGGTTGGGAGCTATTTAAGAAGGTATAACAATAGAGCTACTAAAAAAACAACAATCCTCTA
TAGATCCCCTTTAAGCTTTGATAATAGCACCAATCTTATAAAAAAAAACTAGTCAGTTGATCAGACTTTTATATGGCCAC
ATAATCCACAATCCTAGGCAGTGACTTTAGCTTTCACCTTCCGGTTGGGGCTTCTGCGAGTCAGCTCTGCGAGGAAGGAG
TCCGCTGGCTGCGATGTTTCACTTGATTTACATATGAATCCGCATAAAGCACGGCAGTCCGAAACTGCAAAGGAATGTGG
GCCAAGCCCCCAGTCCATCCTCTTAACCCGCCGACGCCGC

65. lincRNA.484 (splicing site mis-annotated, excluded in the analysis)
CTAAAATGACTTTTCAATCATTTCTAAAATATCATTGATTCTAAATCGTATACGTATCTTGGATCATCTGGGGACATTTG
AGGATCCTACCTTAATCCCTGCTCGCATTTCCATTACCCGTCTCCGGTCATTAGTCATTCGGCAGGCAGATATGCAATT**T**
CCGgtaattcgtaactcgactagctttgcataaactctcaaccatatttccg***cttattcaccagTCCGGTCTAAG***TGCCT
CTGACCCGATCCCGATCCCGATCGCGATCCTGAGGACAACGACGACGACGACCCTCGACGAGTGTGACTCAGGCAATTAG
CGGAGGAAGTGAGTCGAGGAGTTTGGGACAGCAGACGGATTTGTGTCATTAGCGCACTCGCGCAGCTCGCCACTTGTGTC
CG

66. lincRNA.490
CAGCATTTAAGGATTGCCGTTTTCGATTCAAATTATCTGAACAAAGCAATGTCTCAAGTGGCAAGGGTAAGGCACGTAAA
TAAAAACGAGGAATAAAATCCGGGAAAAAACCCCATGCACACATATATACGATATGCGAAATGAAAGTGAAGTGAGCGCA
GGATAATGGCGACCAGAGCGGGACGGAAGGCCAAAATGAGTGAGAGAGAGAGAGAGCGATCTGGAGTAAGGGTAATGAGT
GTGCATAATGCAGATGCAGATGCA***GGATCCCCGTCCAGGATCCCAGG***AGCAGACCAATCCAGCCCATCTCCAGCACTCAT
TGAACAAGCGGGCTCAATCTGCCATGCCAAGATGCACTTGGGTAGTAGTACACTGGAA

67. lincRNA.623
CGAATCTAGTATACCTGTTTAAGCAATGAAAACGTTTCTACTGCTCCAACTGTATATGTACATGCTGCTTGGGCCTAATA
ATTAACCTTGCTGATAACCCTCATCAAATCA***CCAAACTGCCGTCCACACAATTC***CATGGTGCTCCATTATCAAATCCCTG
TTGGATGTCCGGTGCCTGAATCAACACAATTCAAAAGCCCACCCAGAAGTCGAAACCCGTTTTCGGCACCATCGTTCATT
CATCATCGCCGCCTGGCACTTGGCAAATTTACACAAGCCAGGTAAACGGATAGACAGAAGGACGGAGCCAGCCAGAGCCG
AAATACAATTAAAACCCAATCTAAACTCAAATTTTAAATCACAAAAGTTATCAGTTACCTTTCGGCCAACAGCAGAAGCA
GCAGCAGTAGCAGCCGCACGTAAACTTTGGTCAGAATCTGTAAGTTTGCAGCTGAATCTTGGTCAAAAGATACGGCTACG
ACTCCCATACACACGCACCTTTGGCAAACACACTCAATTATAGATACAGACAGACAGTGAGACCCACAAACTTCCGTGGA
AAACTTCTCGGGCACTTTGAAAATGAAGCGAATCCTGGCCAGACAGACGGGCCGTTTCCCAAGTTCGATTTCTGGCATTT
GCTGTGCTCGGCAAAATGCATTTGCCATTCCAAACTAGTTCCAATTGATTAAGTTTCTGGTTAATGGATTGTTTACATAC
CTAAGCAGGCCGACTCACAGAAGTGCAACTCTGCAACTTTTGGCAATCACCACTAAATGGTAATC

68. lincRNA.786
CTGGGAGGCGTCTCACCTTGCCTTCCTGACCTGGGCTTGGCCTGGGCTTGTGGCCAGGGTTCTGGCCCTGGTTTTGCCCT
GGCCGACTATCAGTGGGTCAGTGTCATCATCCGAGCCGCACTGCTGAGCATCAAATGGCCGTTGGCCAATAAAGCCAAAC
ACTTGGCCAGGGTGTGGGGAAGTCTCGACTGGCGACCAGGTCTCTCTCCGCATCATCTTTTGGGCCGGGCTCTAGATTCG
AATGCGCCTGAAGTTGGAGATGGGGCAGATGGAGGTGCTCACGTCACTTTTCTTGTTTGATAAGGATCCGCAATCGAGAA
CGAAGACAGGGCGAAGGCGTACGGTCGAATATATTGGAATTTTATTATGCATGACAGCTCCAAAAAAAGAAGAAACAACA
ATGGACACGACGCCAACAACAAGGAGCGGCTGGGTCTCCTCATCATTTTTCCCTCATTTCTGTTCGGCTTTGATTATTAT
TTTGTGTATGGAGAACGATTCTTGAATATCGATAAGGTGCAAAGGCGGAACGGCTGCTTTGTTGTTGGATCGGCATCGGT
TCTTGTTCTGCATGTTTTGTGCATGATTATGTAACATTTTTTCGTGGCTGAC***CCGTCCAAGCGTTCCTCGAAAAGG***AAAC
ATCGATTTATATTTTGACAAATGATTTACCAATGAGTTGACAGTTTTATTCGGCTTATTTTCCACTTTTTTTTATGGAAA
CGGTTAATAAAAGAAACAACGAGGAGGGAACATGTATT

69. lincRNA.626
CTTTTACACAATAGTATTGTGTGATTTATTAGTCAGAAGATGGCAATGAGCTTTTCATGGAATCCATATACGGCGAAACA
AATTAAAGTTTTGTTTGCACAAAATCTTTAAATTCGGGATTTCTACCTTTTGTAAAACGTACACATATCACTCAGTTTTT
TCTTATTGAAGTTCAGATTTATTTGGTAATGATCGGACGGATGGCCGAATTAACACAGCTTCATGTTTACCATGAAAGTT
GCCGACTCTATAAAGTATATTTATTCTTGATCGGAATTA***CCAGTCGATGTCCGTCCCGTCTA***GAAAGTTAAGGTGAAGAA
TGTAGATTTTAGTACTGTCAATCCCACAGAACGCCCAAACCTACCAAAGCGACCACACTTGAAAAAAAATATTTGAAATT
ATGCAATATTTTTTATTGTTTGTTTGTTGGTCACGCTAACAAACAGCAACTGCAAGTTGTCACTCTCAATTCCTAAAAAT
CATTTGATATTTTTCTTTTATTATTTTTTACCAATATATAAATAGGTAAAGTTATATCAAGAGAACTTTTAAAATTCTCC
CTGCCCACTATATGTGTGTTTATATGTATACCTTAGGCCATATGTTTTTTTTTTTTTATTTTCTTATGTACGAATCCAGT
TCTAAGGCCGATATTATTTTAACCTTAATAGTTCAAGTTTAATAAAATTTGAACAATAAGACAAACGAACAATTTTCATA
TGAAAATATAGTTAAAAAGAATGCACTGAATGTTTCGAGACATTTAGTTTGGCACAAACATTTTGCAAATGCGTACAGGA
TGAAAATTTTCAGTAAACAAAACAGCTTCTAAATCGAGTGACAGTTGAGTAAGATTTAGTGACTAGATGAAATGTCTTCA
ACGTTTCGCAGAGTATACCTATATATATATACAACTTTAAGCCACATCCAAATGACCACAAAGTATCTTGGCCATTATCAAA
ACTGTACCAGCTGCCTGGTAAAATTAAGCCGAAAACTTTTCCATCAATCGCAGACGATCCGAGCACAAGCGACCAGAGAG
ACAAGTCAATTCATTTCCCCTAACAAAAAATCAGTACACCTTTAAAAACTCATATACAAATACATTAGCTTTTTAAATAT
TTTGATATCGTTTCGAATTAGTTATTTCTATCATTAATTAATAATTTTGCCTTCCTTCCTTTAGCTGTGTAATGAGTATC
TGAAAGCCGAGTAGCTTGTAACTGAAAATAATGATGGTTCGCTGCCGATGCTGCGCTGCCTCCGCTGACCGCTGGCTGCT

GCATCCACTGCAGAGACGAAGGATGTGGATAGGGCATAGGCACACATGCATACGCCTACCTGGACACAGGTAGCTCGGCC
TCCGCAGGTAAGCAAGTCTTTGGCCCGGCAAACACTGCCGCAGATATGTATGTGCGTCCATACAGATTCCTCTGCTCGTT
ACTTGTTGCAAACAAGCGCTGAGGACTGAGGAGGGGGACAGCACCACAGACTCAACCGGCTGCCCCTGCCCCATGCTGG
GCACCACCGGCAACATTTAGCAGCGTCATGATCATCAGGGCCCAGGCACTGGGCACTAGGGACTCATGACCAAAGCCAGC
CGGACTGGTTAGGGATTTGGCCCGAGATTGGTTTGGCGCTGCAGAGGAAAGATGCAACATCGGTCAGCTTCAGGGCTGCA
GCATCATGGTATAAAAAGGAGACTAGTTATCAATTCGGGATATATGTTTCGCTGCTTTTGTTTGTTAATACAAATTAAAA
TAAAATATATCTTAAAAATATTGCTGTTAAAATATTTTATTAAAGTTTTCACGTACAACAAAAGTCGGTAATTTATTTAA
TAACCTAAATACTTGCAAAGTAATTTATTTAGCGTTGCTATTCT

70. CR43314

CAGGCAAGTCGCAAAGTGGAACGCAGCGGCGAACGAGCTAAGAAAGTCAGTTGAAAACAGTGGAAACGCGGTGCCAGTCA
CTTTTTTTCATTGCACATAGAGAGAGGGCCCTGTATACTCAGCAGCCTACCACCAACAATCAACCAACCAACCGACCAGC
CCAACCAACATACCTGTCGTGTTGGCCACGTTTGTGGCGATCCGATCAGTGCACCGTCAGCCAGCGACGCGCACACACCG
AACCACACAATCCAGCAGTCTGGGAAACGAACACCACTGTATATACTATCAAGCGAAGTGTTAGAGATAAACTGATAATA
CAACGGAGCCGAGAAAAGAAAAGCACTAACGTGGCGTTAACTCTTTTAACTAAAAAACACTCACTCACTCAACCAAAAAA
AAAAAAAAACGTCAGTAAAGACAATAAACAAAGTAAAACAAAAACGAGTCAGCGGCAAAGTGTCAATAAAACTCTCGAAT
TCATTAACACAACTCAAAACGTTCTATTAGTAAGCACTTGCAAATTTAAACGAAACAAAAAAAAAAAAATCTTGACAGAGA
GAGAGAGAGACAGAGAGCGATAAAAGGAGAGGGAACATTAGACAGAAATTAGCGTGGCCAACAGTCAAAAGGTCTAATAG
AAGCGCGTGTATACGAGTCAAAATGCCCGTATACACCTCGTCGAGACCCACAAGCCATGATCGTAAAAACCACATCAACA
AATGTTCGAAACGAGATAAAAAAAAACAACAACCGAGAATATATGAAAAATACAGAAAAAAAAAGATACAAAAACCCAGACG
AGACCGCTGAGACATGTCCTTGGCCTGTAAAAGGGCAG**//(11079bp)//**GCGAAGTGTAGCAGAGCAATTGTCAATTG
CACCACCCTATAAAAGAATGCACAGGAAGAAATTCTATATTTTCAAACTATTTTATACATTAAAAATCCAATGAAAGTAA
AAAATGTATAATGTAATCAAAGTTAAAATGTACAGCTAGTTTATATATCAGGTTCCCATACCATCTTAAAATTTTAGTAG
TTTCGGGACAAAAAATGGGTAAATCAACTGTGGCAACCTATTGAGGCAAGTTTTTGTTGCTCCTGTTTCCTGTCGTTACA
TGAGATGCACGTTGTAAGCGGATAGGCTGTGAGGGTTGTTCAGAAATTCACTTAATAATAATATTGCATTATATATTCAA
TGTGAAAGCTTTAACTGAAATGATTATCTATGAAACATTTAAAGAGTATTACATTCAATATGTGATATACAATTTAAATT
CAATACTCACCAATCTGCCCTCGCTTCGTACCCATTGATACTTATGCCAATTA***CCATTCTCCGTTCGCCTCTTTGC***AGAC
AAATGCCATGGACTCGCTTACAATTTGGTCTCGTCTTGTCAATCAATTGGGATCGAGTCGGGGTCTAGAACTGGAAATGG
CCAAGGATGACCGGCGACCGTTTCCTGTTTGGGTTGGCAAGTTGGTAAGTTAATACTCGGACTAGGCTCTAACTTAATTA
GCCGCACGACAGACTGAAGACTAAAGACTGAATGCCGAAAGCACACGACCCCGACCTCTGGTGACCTCAAAGCTGACGGA
GGCGACTAATTTATCAAAATGGCTAGAAAAACTGAAATAACTCTACGCATAATTAAATCCAGCTAAAAACGAAAAATGAG
TGAAACACCGCGATACTCAAATGCGTTTTTAAACCGTTGCGCAACGCCTATTTCTAACAGTTTTAAATTTTGTTGAGACT
TTTCTTTGGTGTTTTGTTCGAATTTTTCTTTTGCTTAATTGCCGCCTATTAATCC**//(5705)//**TTCCCTTCTGGCCAA
GTTCCGCTCGAGCACATTTATGCGGAAGAAGAGGGGTCTTTTTAATGATGACGCTTCCTGTTGGCCGGCCGCACATATAT
TTATGAGACACCTTGGCGTTGATAAGTCCCAGACGAAGCTTCAATTGAAATTTGTACTTAATTAAGTCTGGTATGGCGAA
CCGGCGTTTCTTTGATCATCGACTAAGAAGGAATTCTGAGAATTCGTCAAGTAGCCTACACTTGGGAGCAATTATTCACT
TAGGCTTAAAAACAATATTTTTTAAACGCGGGAAAATAGAATTTACAAATATTT

71. lincRNA.118

GTTTTGGTATGCTTTTATTTGCTTACTTTATAAGATAGTATAGTTTAATGGAACATACTTTCTTTAGATTTACATTTAAG
AGAGGTGGGTATCATATTACTTGTAGAGTGTATACTTTGTGGATTCATATTTGTAGTTCGTCACATTCCATTGAACGACT
TTCGGGCCGCTTCAATTCATTTGCGGCATAGTATTATACAAATAACGCAAAATATTTGGTATGCGAGTTAAATATTGAGT
GCTATTATTGTTCGTCTG***CCGCGAAGTCTGCCCGTCCGTCC***AA**CCGTCC**AA**CCGTCC**AA**CCGTCC**AACTGT***CCATCTGTC***
CATCTGTCCAAATTACCAGTTCAAGCTGGTTAAGTTTTCGGTCACGGACGTTTGGAGCCA*GTTTTGGCTAGATT**CTAGTA***
TCCGTCCGTCGAGCGAACATAACAGCATCATTTTGCGCAGCATGGTTCGCTGACTTAATGAACTCAACACGAGACGATCG
ACGATGGAGCTTGAAGATCATCACAGCTCAGTGGTGGTCATATCGGATATCATGGATGTTGGATGTTCGGTCGTTTCAAG
TGGATCGGATGCGATGTGCGTGCGGCTAATTGATGGGGTGCACATCAATTGAAGTTTGTCACTTTTCATTTGGGCCCAAG
ACGCGGAATATTTCCAGTCCAAGTCGCTGTCAGCAGATTGGAATTCTCTGCCAACTCCGGTTATCTGCGCCCAGATGTCA
CCTGGATGATCTCGTGTCTGGTCTGGACAAAGTGTGCCATCGATAAGGCCGACAAAGTTTAACTGATAAACATGGTCTAC
ATACATACATACTTACATACTCTTTGGGCTTCAAATGGAGAGCACTCGGCAAAGGATTATATTTCCGGCGATAGTGATTG
AGATAATGCATGTGCCAAATCTTAACGGACGCACAATAATCCCCGCCATAAGTACGATTCATTGAGACACCAATTGAGCA
CTTTCCAAGTAAAACCGAAACAGATCGCATAACATTCCACGAATAATACGTTTAAGTATCCCCTTGAAATGTATATACAT
ATATCGGCA
